# Targeted Degradation of SOS1 Exhibits Potent Anticancer Activity and Overcomes Resistance in KRAS-Mutant Tumors and BCR–ABL–Positive Leukemia

**DOI:** 10.1158/0008-5472.CAN-24-1093

**Published:** 2024-10-22

**Authors:** Ziwei Luo, Chencen Lin, Chuwei Yu, Changxian Yuan, Wenyong Wu, Xiaowei Xu, Renhong Sun, Yan Jia, Yafang Wang, Jie Shen, Dingyan Wang, Sinan Wang, Hualiang Jiang, Biao Jiang, Xiaobao Yang, Chengying Xie

**Affiliations:** 1 School of Life Science and Technology, ShanghaiTech University, Shanghai, China.; 2 Shanghai Institute for Advanced Immunochemical Studies, ShanghaiTech University, Shanghai, China.; 3 School of Physical Science and Technology, ShanghaiTech University, Shanghai, China.; 4 Lingang Laboratory, Shanghai, China.; 5 Department of Hematology, Shanghai Jiao Tong University School of Medicine Affiliated Shanghai General Hospital, Shanghai, China.; 6 Gluetacs Therapeutics (Shanghai) Co., Ltd., Shanghai, China.; 7 Department of Pharmacy, The SATCM Third Grade Laboratory of Traditional Chinese Medicine Preparations, Shuguang Hospital Affiliated to Shanghai University of Traditional Chinese Medicine, Shanghai, China.; 8 School of Biomedical Engineering & State Key Laboratory of Advanced Medical Materials and Devices, ShanghaiTech University, Shanghai, China.; 9 Drug Discovery and Development Center, Shanghai Institute of Materia Medica, Chinese Academy of Sciences, Shanghai, China.

## Abstract

Son of sevenless homolog 1 (SOS1) is an essential guanine nucleotide exchange factor for RAS that also plays a critical role in the activation of the small GTPase RAC mediated by BCR–ABL in leukemogenesis. Despite this, small-molecule inhibitors targeting SOS1 have shown limited efficacy in clinical trials for KRAS-mutant cancers, and their potential as a therapeutic approach for chronic myeloid leukemia (CML) remains largely unexplored. In this study, we developed a potent SOS1 proteolysis targeting chimera (PROTAC) SIAIS562055, which was designed by connecting a CRBN ligand to an analog of the SOS1 inhibitor BI-3406. SIAIS562055 exhibited sustained degradation of SOS1 and inhibition of downstream ERK pathways, resulting in superior antiproliferative activity compared with small-molecule inhibitors. SIAIS562055 also potentiated the activity of both KRAS inhibitors in KRAS-mutant cancers and ABL inhibitors in BCR–ABL–positive CML. In KRAS-mutant xenografts, SIAIS562055 displayed promising antitumor potency as a monotherapy and enhanced ERK inhibition and tumor regression when combined with KRAS inhibitors, overcoming acquired resistance. In CML cells, SIAIS562055 promoted the active uptake of BCR–ABL inhibitors by upregulating the carnitine/organic cation transporter SLC22A4. SIAIS562055 and BCR–ABL inhibitors synergistically enhanced inhibition of ABL phosphorylation and downstream signaling, demonstrating robust antitumor activities in both mouse xenografts and primary samples from patients with CML. In summary, this study suggests that PROTAC-mediated SOS1 degradation represents an effective therapeutic strategy for treating not only KRAS-mutant cancers but also BCR–ABL–harboring leukemia.

**Significance:**

The PROTAC SIAIS562055 sustainably degrades SOS1 and inhibits downstream ERK signaling, showing strong antiproliferative activity and synergistic effects with KRAS inhibitors in KRAS-mutant cancers and BCR–ABL inhibitors in chronic myeloid leukemia.

## Graphical Abstract

**Figure:**
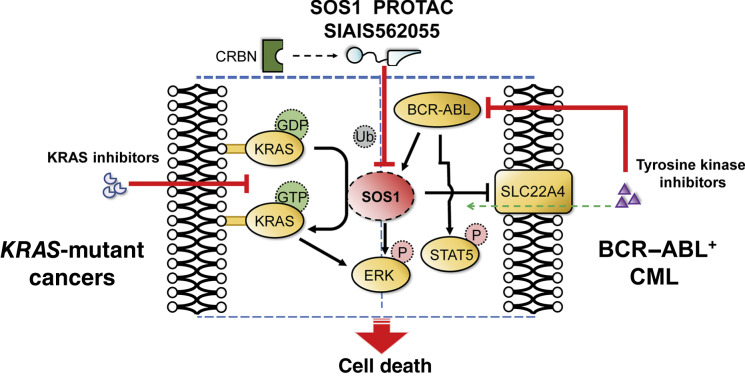

## Introduction

*KRAS* is a member of the *RAS* gene family, which also includes two other isoforms: *HRAS* and *NRAS* (1). Among these three genes, *KRAS* exhibits the highest mutation frequency in pancreatic ductal adenocarcinoma and is correlated with non–small cell lung carcinoma (NSCLC) and colorectal carcinoma (2–4). Mutant KRAS, which serves as a driver in almost 11% to 14% of cancers, remains constitutively activated and leads to the sustained activation of downstream signaling (5, 6). KRAS contains several mutation types; for instance, G12D is the most common mutation in pancreatic ductal adenocarcinoma (7), whereas G12C is prevalent in NSCLC (8). For a long time, KRAS has been considered an unmanageable target, with no effective targeted drugs available. Recently, covalent KRAS^G12C^ inhibitors, such as sotorasib (AMG510) and adagrasib (MRTX849), have been successfully developed and approved by the FDA, with several more in the pipeline (9–11). However, the therapeutic benefits are limited by the intrinsic or acquired resistance to KRAS^G12C^ inhibitors in clinical settings (12). MRTX1133, a noncovalent KRAS^G12D^ inhibitor, has demonstrated effective inhibition of KRAS-dependent signal transduction, leading to regression of KRAS^G12D^-mutant tumors in preclinical studies (13). Nevertheless, there are currently no effective drugs in clinical trials for other mutation sites, underscoring the significance of targeting KRAS-dependent signaling pathways for the treatment of KRAS-mutant cancers.

Chronic myeloid leukemia (CML) is a malignancy of the hematopoietic system characterized by Philadelphia chromosome (BCR–ABL1; refs. 14, 15). Fortunately, imatinib, a tyrosine kinase inhibitor (TKI) also known as a BCR–ABL inhibitor, revolutionizes CML treatment as a groundbreaking targeted drug (16). However, clinical practice has revealed that resistance to imatinib inevitably occurs due to several mutations in BCR–ABL kinase that reduce the drug binding affinity (17). Subsequently, second- and third-generation ABL inhibitors, such as nilotinib, olverembatinib, and asciminib, have been developed to provide a more powerful inhibition of BCR–ABL and alleviate imatinib resistance in patients with CML with these mutations (18–22). However, some patients develop resistance with unidentified resistant mutations that underlie significantly heterogeneous mechanisms. BCR–ABL–independent TKI resistance varies considerably among patients, accounting for up to 50% of cases (23). These pathways, molecules or niche-related factors that are independent of BCR–ABL contribute to the proliferation and survival of leukemic stem cells in CML, thereby promoting drug resistance (24, 25). Therefore, there is an urgent need to develop new therapeutic approaches for treating patients with TKI-resistant CML, especially those driven by BCR–ABL–independent mechanisms.

Son of sevenless homolog 1 (SOS1) is an essential guanine nucleotide exchange factor (GEF) in KRAS-driven tumors (26). Targeting SOS1 has emerged as a promising strategy for the treatment of KRAS-mutant cancers. Several SOS1 inhibitors, such as BI-3406 and its analog BI-1701963, have shown efficacy in preclinical studies and clinical trials (27, 28). However, due to concerns including challenging pharmacokinetic (PK) characteristics, safety issues, and limited monotherapy efficacy, phase I trials of BI-1701963, either as a monotherapy or in combination with BI-3011441 (NCT04835714) or irinotecan (NCT04627142) for treating patients with KRAS mutations, have been terminated. Interestingly, SOS1 also functions as a downstream node protein of BCR–ABL, underscoring its critical role in CML pathogenesis (29–32). Although the molecular mechanisms of RAS GEF catalysis have been uncovered, the process by which SOS1 acquires RAC GEF activity and the physiologic and pathologic implications of this activity remain poorly understood. Genetic interference with SOS1 has been shown to significantly reduce RAC-GTP levels, inhibit BCR–ABL–induced proliferation and transformation *in vitro*, and delay leukemogenesis *in vivo* (30). To date, limited research has been reported apart from BAY-293, a SOS1/RAS inhibitor, which is documented to enhance imatinib sensitivity and impede the proliferation of BCR–ABL–independent TKI-resistant CML cells (33). These results indicate that SOS1 is a potentially valuable target for the treatment of both KRAS-mutant cancers and BCR–ABL–positive (BCR–ABL^+^) CML, thereby necessitating the development of novel strategies to effectively target SOS1.

Proteolysis targeting chimera (PROTAC) provides a novel method for targeting specific proteins and has been widely investigated for oncotherapy (34). There are three parts of a PROTAC: a ligand that binds to the protein of interest, a ligand that binds to a certain E3 ligase, and a linker connecting them (35). PROTACs can efficiently promote the ubiquitination of protein of interest, and the ubiquitinated protein is subsequently degraded via the 26S proteasome (36). Recent advancements in targeting protein degradation aim to make “undruggable” targets accessible, addressing challenges such as drug resistance and gene mutations. The PROTAC strategy has been reported to successfully degrade KRAS (37, 38), BCR–ABL, and STAT5, which is an important downstream signaling protein in CML cells (39–43). Additionally, SOS1 PROTACs based on SOS1 inhibitors or agonists have been developed, providing a new avenue for the treatment of KRAS-mutant cancers (44, 45). Further investigations are warranted to explore the potential of SOS1 PROTACs in suppressing BCR–ABL^+^ CML and to elucidate the underlying mechanisms.

In this study, we designed and synthesized a series of SOS1 PROTACs based on the ligand of CRBN and small-molecule SOS1 inhibitors (BI-3406 analogs). It was identified that the compound SIAIS562055 sustainably degraded SOS1 levels and inhibit downstream effectors, exhibiting potent antitumor activity in both KRAS-mutant cancers and BCR–ABL^+^ CML. Moreover, we demonstrated that SIAIS562055 alone induced robust antitumor efficacy and exhibited synergistic activity when combined with KRAS inhibitors in KRAS-mutant cancers, including those with acquired resistance to KRAS inhibitors. Furthermore, SIAIS562055 promoted the uptake of imatinib and showed synergistic lethal effects with TKIs, leading to tumor regression in CML xenograft models without additional toxicity and significantly suppressed cell viability in primary CML samples. These results indicate that targeting SOS1 for degradation may be an effective therapeutic strategy for both KRAS-mutant tumors and BCR–ABL–harboring leukemia.

## Materials and Methods

### Compounds

The chemical structures and syntheses of all PROTACs used for screening, including SIAIS562055 and SIAIS562092 (negative control), are shown in the Supplementary Materials. BI-3406 (HY-125817), MRTX849 (HY-130149), AMG510 (HY-114277), MRTX1133 (HY-134813), imatinib (HY-15463), nilotinib (HY-10159), olverembatinib (HY-15666), and lenalidomide (HY-A0003) were purchased from MedChem Express.

### Cell line and cell culture

The leukemia cell lines K562 (RRID: CVCL_K562) and KU812 (RRID: CVCL_0379); pancreatic cancer cell lines MIA PaCa-2 (RRID: CVCL_0428), HPAF-II (RRID: CVCL_0313), and BxPC-3 (RRID: CVCL_0186); colon cancer cell lines SW620 (RRID: CVCL_0547) and GP2d (RRID: CVCL_2450); and NSCLC cell line NCI-H358 (RRID: CVCL_1559) were purchased from the ATCC. These cells were cultured in medium as recommended by ATCC and maintained in a 37°C incubator. Cell lines were cytogenetically tested and authenticated using the short tandem repeat method as ATCC profiles. The cancer cell lines were used within 25 passages. Mouse parental Ba/F3 cells were cultured in RPMI-1640 medium (Thermo Fisher Scientific) supplemented with 10% FBS (Thermo Fisher Scientific). Additionally, 10% WEHI-3–conditioned medium was added to provide IL-3, except for Ba/F3 cells that were transformed with plasmids expressing mutant KRAS. All cell lines were confirmed to be negative for *Mycoplasma* contamination by the MycoBlue Mycoplasma Detector (Vazyme, D101-01).

### Modeling of ternary complex structure

The PROTAC-Model was used to predict the ternary complex structure mediated by SIAIS562055, with all parameters configured using their default values. The workflow began with the acquisition of crystal structures of protein complexes and their preparation for docking. The coordinates of SOS1 in complex with BI68BS [Protein Data Bank (PDB): 6SFR] and of CRBN in complex with lenalidomide (PDB: 4TZ4) were obtained from the RCSB PDB (28, 46, 47). These structures were prepared using a protein preparation wizard in Maestro, which is a part of the Schrödinger suite (release 2020-3). Docking analysis was conducted using the four-step PROTAC-Model process, which included docking, filtering, refinement, and rescoring. Intermolecular interactions were analyzed using Schrödinger software.

### Cell viability

Cells were inoculated onto 96-well plates and treated with compounds in serial dilutions for 3 or 5 days. Cell viability was assessed using sulforhodamine B (230162, Sigma-Aldrich) assay and methyl thiazolyl tetrazolium (M5655, Sigma-Aldrich) assay for adherent and suspended cells, respectively, in accordance with previously described methods (48). The cell viability of primary patient samples was tested using the CellTiter-Glo Luminescent Assay kit (G7573, Promega) according to the manufacturer’s instructions. Three-dimensional cultivation was conducted in 96-well ultralow attachment plates (7007, Corning). The optical density (OD) values were obtained using a SPARK microplate reader (Tecan). IC_50_ was calculated using GraphPad Prism 8.0.2 (nonlinear regression analysis with four parameters). Drug synergy was evaluated with combination index (CI) values using CalcuSyn Demo Version 2.0 software. CI > 1 indicates antagonism, CI < 1 indicates synergy, and CI = 1 indicates additivity.

### Colony formation

NCI-H358 cells were seeded into plates at a suitable density. SIAIS562055, either alone or in combination with the KRAS^G12C^ inhibitors MRTX849 or AMG510, was diluted and added to the cells for 7 days. Following treatment, all the plates were fixed with paraformaldehyde and stained with crystal violet. Data analysis was performed using the ImageJ software.

### Western blot

After treatment, the cells were harvested with SDS lysis buffer, and SDS-PAGE gels were used to split the proteins in the lysate solutions. Subsequently, all proteins were transferred onto polyvinylidene fluoride membranes (10600023, Cytiva) and incubated overnight at 4°C with following primary antibodies: ERK 1/2 (9170, Cell Signaling Technology, RRID: AB_10695739), phospho-ERK 1/2 (4370, Cell Signaling Technology, RRID: AB_2315112), cleaved caspase-3 (9664, Cell Signaling Technology, RRID: AB_10831820), β-tubulin (56739, Cell Signaling Technology, RRID: AB_2799519), α-tubulin (2125, Cell Signaling Technology, RRID: AB_2619646), SOS1 (12409, Cell Signaling Technology, RRID: AB_2797902), STAT5 (94205, Cell Signaling Technology, RRID: AB_2737403), phospho-STAT5 (9351, Cell Signaling Technology, RRID: AB_2315225), eRF3a/GSPT1 (CL488-68217, Proteintech, RRID: AB_3084444), IKZF3 (19055-1-AP, Proteintech, RRID: AB_10643381), IKZF1 (66966-1-Ig, Proteintech, RRID: AB_2882289), β-actin (66009-1-lg, Proteintech), GAPDH (60004-1-lg, Proteintech, RRID: AB_2919223), c-ABL (A22082, ABclonal), phospho-c-ABL-Y412 (AP1060, ABclonal), and SLC22A4 (A10490, ABclonal, RRID: AB_2758039). Images were taken after the addition of enhanced chemiluminescence substrate (32106, Thermo Fisher Scientific) and analyzed using ImageJ for relative quantifications.

### Protein expression and purification

The human protein SOS1 (564-1049) was produced in ER2566 competent cells and subsequently purified through affinity chromatography, followed by molecular sieve chromatography. The SOS1 protein was harvested with buffer containing 10 mmol/L HEPES, pH 7.5, 150 mmol/L NaCl, and 10 mmol/L β-mercaptoethanol. All proteins were stored at −80°C before use.

### Surface plasmon resonance assay

Biacore 8K instrument (GE Healthcare) was used for surface plasmon resonance (SPR) experiments with buffer (10 mmol/L HEPES, pH 7.5, 150 mmol/L NaCl) at 25°C. The Series S Sensor Chip CM5 (BR100530, Cytiva) was pretreated with an Amine Coupling Kit (BR100050, Cytiva) before protein immobilization. SIAIS562055 was continuously diluted in running buffer and injected into the instrument. Data were recorded, and the equilibrium KD values were analyzed by fitting the dose–response curve using the Biacore 8 K Insight Evaluation software (GE Healthcare).

### Homogeneous time-resolved fluorescence assay

A homogeneous time-resolved fluorescence (HTRF) assay was employed to quantify the interaction between KRAS and SOS1 proteins. The impact of the compound was evaluated using the KRAS^G12C^/SOS1 Binding Kit (63ADK000CB16PEG, Cisbio) and KRAS^G12D^/SOS1 Binding Kit (63ADK000CB17PEG, Cisbio), following the manufacturer’s instructions. Fluorescence at 620 and 665 nm were detected using a SPARK microplate reader (Tecan) and calculated as OD (665 nmol/L)/OD (620 nmol/L) × 10^4^. The data were further analyzed using GraphPad Prism 8.0.2.

### GTP protease-linked immunosorbent assay 

Cells were initially grown in a medium with 10% FBS for 24 hours, followed by serum starvation in a medium containing 0.5% FBS or BSA with or without 2 μmol/L SIAIS562055 for an additional 24 hours. Subsequently, BxPC-3 and MIA PaCa-2 cells were stimulated with 2 ng/mL EGF for an additional 2 minutes. Lysates were collected to detect the level of RAS-GTP by GTP protease-linked immunosorbent (G-LISA) RAS Activation Assay Biochem Kit (BK131, Cytoskeleton) following the manufacturer’s instructions. The levels of RAS-GTP and RAC-GTP in K562 cells were tested by G-LISA RAS Activation Assay Biochem Kit and RAC1 G-LISA Activation Assay Kit (BK128, Cytoskeleton), respectively. The absorbance at 490 nm was quantified using the GraphPad Prism 8.0.2, and the levels of RAS/RAC-GTP were normalized to the absorbance of the loading control.

### Cell apoptosis assay

Cells were collected after treatment with the compounds at specified concentrations and durations. Subsequently, quantitative detection was performed using an apoptosis detection kit (C1062L, Beyotime), following the manufacturer’s instructions. Fluorescence data were assayed using CytoFlex (Beckman Coulter) and analyzed using the FlowJo V10 software.

### siRNA-mediated knockdown assay

The siRNAs targeting SOS1 or SLC22A4, along with their respective control siRNAs, were synthesized by Tsingke Biotechnology. These siRNAs were transfected into cells plated at 60% to 80% confluency using Lipofectamine RNAiMAX (13778150, Thermo Fisher Scientific) following the manufacturer’s instructions. The siSOS1 sequence was as follows: 5′-GGC​AGA​AAU​UCG​ACA​AUA​U (dT)-3′. The siSLC22A4 sequence was as follows: 5′-CUCGGUGCUUACAACAGAA(dT)-3′.

### RT-qPCR

After treatment, total RNA was extracted with RNAiso Plus (9109, Takara), and cDNA was obtained using a cDNA Synthesis Kit (6210, Takara) at 42°C. For the RT-qPCR assay, the AB QuantStudio 7 Real-Time PCR System (Life Technologies) was used. The primer sets used were as follows: For GAPDH, the forward primer was GGA​GCG​AGA​TCC​CTC​CAA​AAT, and the reverse primer was GGC​TGT​TGT​CAT​ACT​TCT​CAT​GG; for SLC22A4, the forward primer was TGG​TAG​CCT​TCA​TAC​TAG​GAA​CA, and the reverse primer was TGG​CAG​CAG​CAT​ATA​GCC​AAC.

### Imatinib uptake assay

Protein levels in different treatment groups were quantified by BCA assay. The supernatant and mobile phase were mixed and incubated overnight at 4°C. A solution of 4-bromobenzophenone was added to the samples as an internal standard. The mixture was then applied to an Oasis HLB extraction cartridge (913737, J&K Scientific) that had been activated by methanol and water (1:1, v/v). The cartridge was washed and eluted with methanol (49). After that, the eluent was removed, and residues were dissolved. High-performance liquid chromatography analysis was performed on an ELITE Supersil Series Column (4.6 mm inside diameter () × 150 mm length) with acetonitrile in the mobile phase and 50 mmol/L ammonium acetate at a flow rate of 0.5 mL/minutes on the instrument (UltiMate 3000, Thermo Fisher Scientific). The relative content of imatinib was calculated by normalizing it to the respective protein concentration.

### PK study

Compound SIAIS562055 was dissolved in DMSO and Solutol S in saline (5:5:90, v/v/v) and administered to 7-week-old ICR male mice (Huafukang Biotechnology) intravenously (i.v., 2 mg/kg), intraperitoneally (i.p., 10 mg/kg), or orally (10 mg/kg). Blood samples were collected at different time points and were diluted with 50% acetonitrile. Subsequently, the concentration of SIAIS562055 was analyzed and quantified through the LC-MS/MS system.

### Xenograft tumor growth and treatment

Female, 6 to 7-week-old BALB/c mice were purchased from Huafukang Biotechnology Co., Ltd., raised in constant temperature and isopiestic pressure rooms with 12 hours of light per day, and fed with recommended food and water. Animal experiments were carried out following the guidelines of the Institutional Animal Care and Use Committee of Shanghai Institute of Materia Medica, Chinese Academy of Sciences, and Lingang Laboratory. Cancer cells, ranging from 3.0 × 10^7^ to 7.0 × 10^7^ cells/mL, were suspended in NaCl solution, containing an appropriate volume of Matrigel (354248, Corning) and transplanted into the right flank of the mice, subcutaneously. All mice were raised till the tumor volumes reached approximately 100 mm^3^ and were randomly assigned to either the vehicle or treated groups (*n* = 5/group). The treated groups were administered as follows: SIAIS562055 (i.p., daily) or BI-3406 (orally, twice daily) for MIA PaCa-2 xenografts; SIAIS562055 (i.p., daily), MRTX1133 (i.p., daily), or their combination for GP2d xenografts; SIAIS562055 (i.p., daily), MRTX849 (orally., daily), or a combination of both MIA PaCa-2 and MIA PaCa-2/R xenografts; SIAIS562055 (i.p., daily), imatinib (orally, daily), or their combination for K562 xenografts. Body weights and tumor sizes of mice were recorded every 3 days. Tumor volumes were calculated as follows: ½ × length × width^2^. On the final day of the experiment, the mice were euthanized and tumor samples were prepared for Western blot analysis.

### Patient samples

None of the participants in this study had undergone preoperative chemotherapy or radiotherapy. Written informed consent was obtained from each individual, and the study received approval from the Ethics Committee of Shanghai Jiao Tong University School of Medicine Affiliated Shanghai General Hospital. The procedure was performed in accordance with the guidelines of the 1975 Declaration of Helsinki. Mononuclear cells from patient peripheral blood samples were isolated by Lympholyte‐H (Cedarlane). Mononuclear cells were then treated with Ammonium-Chloride-Potassium lysis buffer (A1049201, Thermo Fisher Scientific) to eliminate any remaining red blood cells. For cell viability and Western blot assays, the obtained cells were cultured in Iscove's Modified Dulbecco's Medium supplemented with 20% FBS and 50 μmol/L β-mercaptoethanol.

### Statistical analysis

Gray levels were obtained through ImageJ (NIH). Schrödinger was applied for modeling ternary complex structure. Biacore Insight Evaluation (GE Healthcare) was used for analyzing the SPR data. CI values were calculated by CalcuSyn Demo Version 2.0. Statistical analyses were performed using the GraphPad Prism software 8.0.2 (GraphPad Software). Data *in vitro* were shown as mean ± SD, *n* = 3, whereas data *in vivo* were shown as mean ± SEM, *n* = 5. Significance analysis was performed using a two-tailed unpaired Student *t* test. The notations for the significance levels are as follows: *, *P* < 0.05; **, *P* < 0.01; ***, *P* < 0.001; and ns indicates no significance.

### Ethics approval

The animal procedures were approved by Shanghai Institute of Materia Medica, Chinese Academy of Sciences, and Lingang Laboratory and followed the guidelines of the Institutional Animal Care and Use Committee and guidelines of Shanghai Institute of Materia Medica, Chinese Academy of Sciences, and Lingang Laboratory. Written informed consent was obtained from each patient with CML, and the study was approved by the Ethics Committee of Shanghai Jiao Tong University School of Medicine Affiliated Shanghai General Hospital.

### Data availability

The data of correlation analysis in this study were obtained from GEPIA2 at http://gepia2.cancer-pku.cn. All raw data are available upon request from the corresponding author.

## Results

### SIAIS562055 is a selective SOS1 PROTAC

**Figure 1.**
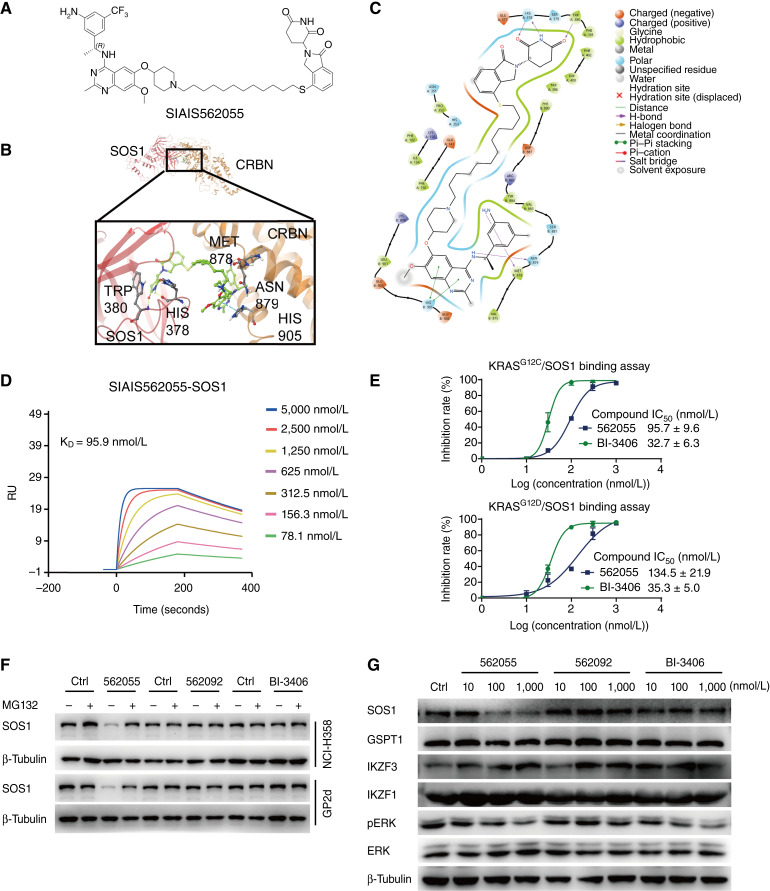
SIAIS562055 selectively degrades SOS1. **A,** Chemical structure of SIAIS562055 (562055 for short). **B,** Ternary complex of SIAIS562055, E3 ligase CRBN, and SOS1 determined by computer simulation. **C,** Visual inspection and interaction fingerprint analysis of the ternary complex. **D,** SPR assay was used to test the binding affinity of SIAIS562055 with SOS1. **E,** HTRF assay of the SOS1 and KRAS interaction after treatment with SIAIS562055 or BI-3406 at different concentrations. Data are shown as mean ± SD, *n* = 3. **F,** Effects of SOS1 by SIAIS562055 (1,000 nmol/L), SIAIS562092 (562092 for short; 1,000 nmol/L), or BI-3406 (1,000 nmol/L) for 24 hours with or without pretreatment of MG132 (1,000 nmol/L) for 1 hour in NCI-H358 and GP2d cells. **G,** Western blot analysis of SOS1, pERK, GSPT1, and IKZF1/3 in NCI-H358 cells treated with SIAIS562055, SIAIS562092, or BI-3406 for 24 hours. Data are representative of three independent replicates.

In this study, we developed a series of PROTACs targeting SOS1, utilizing analogs of a known SOS1 inhibitor, BI-3406, and a CRBN ligand as a foundation. Preliminary screening of SOS1 PROTAC candidates was performed by analyzing cell proliferation inhibition and SOS1 degradation in K562 cells (Supplementary Table S1). Subsequently, six candidates were selected to assess the degradation of SOS1 in MIA PaCa-2 (KRAS^G12C^), HPAF-II (KRAS^G12D^), and K562 cells at concentrations of 10, 100, and 1,000 nmol/L (Supplementary Fig. S1). Among the tested compounds, SIAIS562055 (Fig. 1A), which exhibited the strongest degradation of SOS1 and inhibition of cell proliferation, was selected for further examination. To structurally inspect the ternary complex formed by SIAIS562055, SOS1, and CRBN, we employed a PROTAC-Model, developed by Bian and colleagues (45), to perform structural modeling. An overview of the predicted ternary complex involving CRBN (brown), SOS1 (magenta), and SIAIS562055 (green), with details focusing on the key residues was shown in the expanded view (Fig. 1B). Specifically, the relative positioning of SOS1 and CRBN aligned consistently with the earlier research conducted by Weng and colleagues (50), underscoring the reliability of the protein–protein docking results. Visual inspection and interaction fingerprint analysis of the ternary complex revealed that, aside from the hydrogen bond interactions with His378 and Trp380 in the CRBN domain and Met878 and Asn879 in the SOS1 domain, the π–π stacking between the quinazoline core of SIAIS562055 and His905 was also crucial (Fig. 1C). The BI-3406 analog BI68BS and other SOS1 inhibitors share similar interaction patterns (45). Collectively, these findings provide structural support for the formation of the ternary complex.

To explore the direct binding affinity between SIAIS562055 and SOS1 protein, we conducted an SPR assay. The purified SOS1 protein was immobilized onto a CM5 chip coated with carboxymethyl glucan until saturation. SIAIS562055 directly bound to SOS1 in a concentration-dependent manner with a KD of 95.9 nmol/L, indicating a high affinity between SOS1 and SIAIS562055 (Fig. 1D). Subsequently, inhibition of the interaction between SOS1 and mutant KRAS by SIAIS562055 was detected using an HTRF assay. SIAIS562055 effectively blocked the binding of KRAS^G12C^ or KRAS^G12D^ to SOS1, with the IC_50_ values of 95.7 and 134.5 nmol/L, respectively (Fig. 1E). To elucidate the specificity of SIAIS562055 for protein degradation among all cellular proteins, we conducted label-free–based proteomics analysis using MIA PaCa-2 cells treated with SIAIS562055. Significant degradation of SOS1 in MIA PaCa-2 cells was observed after a 16-hour treatment with SIAIS562055 (1 μmol/L), whereas GSPT1 and IKZF1/3 were not detected, despite their CRBN dependence (Supplementary Fig. S2A; refs. 51–53). Additionally, ANTKMT (also known as FAM173A), a mitochondrial lysine (K)–specific methyltransferase (54), and TNF receptor–associated factor 6, a ubiquitin ligase bridging the RAS and NF-κB pathways in lung cancers (55), were also significantly downregulated. However, the interplay between SOS1 remains unclear.

Further investigation showed that SIAIS562055 induced SOS1 degradation, which was significantly reversed by MG132, a proteasome inhibitor, or lenalidomide, a CRBN ligand, in both KRAS-mutant and KRAS–wild type (WT) cell lines. In contrast, neither SIAIS562092, a negative control, nor BI-3406 had such an effect (Fig. 1F; Supplementary Figs. S2B and S3A). Notably, SIAIS562055 had a negligible effect on some known CRBN substrates, including GSPT1 and IKZF1/3, in KRAS^G12C^ NCI-H358 cells, indicating that SIAIS562055 is a specific PROTAC targeting SOS1 (Fig. 1G; Supplementary Fig. S3B and S3C). In summary, these results indicate that SIAIS562055 is a selective SOS1 PROTAC with a potent ability to degrade SOS1 through the CRBN E3 ubiquitin ligase and ubiquitin–proteasome system.

### SIAIS562055 inhibits the proliferation of KRAS-mutant cells and exhibits synergistic effects with KRAS inhibitors

**Figure 2.**
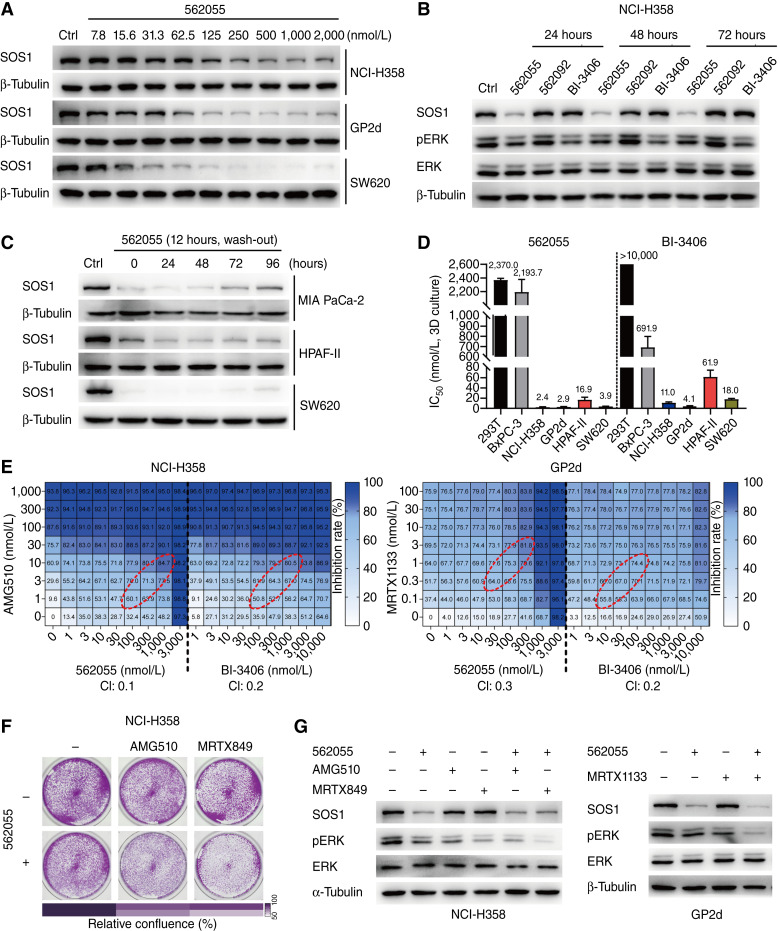
SIAIS562055 sensitizes KRAS-mutant cancer cells to KRAS inhibition *in vitro*. **A,** Western blot analysis of SOS1 in NCI-H358 (G12C), GP2d (G12D), and SW620 (G12V) cells treated with a concentration gradient of SIAIS562055 for 24 hours. **B,** Western blot analysis of SOS1 and pERK in NCI-H358 cells after the treatment of SIAIS562055 (1,000 nmol/L), SIAIS562092 (1,000 nmol/L), and BI-3406 (1,000 nmol/L) at different time points. **C,** Western blot analysis of SOS1 in MIA PaCa-2 (G12C), HPAF-II (G12D), and SW620 (G12V) cells after treatment with SIAIS562055 (1,000 nmol/L) for 12 hours, followed by wash-out and incubation with fresh media for another 0, 24, 48, 72, and 96 hours. **D,** Antiproliferative effects of SIAIS562055 and BI-3406 for 120 hours on KRAS-WT (293T, BxPC-3) and KRAS-mutant (NCI-H358, GP2d, HPAF-II, and SW620) cells. Histogram showed the IC_50_ values of compounds assessed in three-dimensional (3D) cultured cells. Data are shown as mean ± SD, *n* = 3. **E,** Inhibition rates of cell proliferation in NCI-H358 and GP2d cells treated with SIAIS562055 and KRAS inhibitors, alone or in combination, at different concentrations for 72 hours. CI values were calculated based on the data in red oval frames. **F,** Colony formation of NCI-H358 cells after treatment with SIAIS562055 (800 nmol/L), KRAS^G12C^ inhibitors (AMG510 or MRTX849, 5 nmol/L), or their combination for 7 days. **G,** Western blot analysis of SOS1 and pERK in NCI-H358 and GP2d cells after treatment with SIAIS562055 (1,000 nmol/L) combined with AMG510 (5 nmol/L), MRTX849 (5 nmol/L), or MRTX1133 (0.8 nmol/L) for 24 hours. Data are shown representatively from three independent repetitions.

Because SOS1 plays a pivotal role in overseeing the GDP–GTP cycle of KRAS, SOS1 PROTACs may exhibit antiproliferative activity against KRAS-driven tumors. We initially evaluated the degradation of SOS1 mediated by SIAIS562055 in several KRAS-mutant and KRAS-WT cell lines. Our results showed that SIAIS562055 induced concentration-dependent degradation of SOS1 across all tested cell lines (Fig. 2A; Supplementary Fig. S4A and S4B). Notably, neither SIAIS562092 nor BI-3406 caused SOS1 protein degradation, whereas SIAIS562055 maintained low levels of SOS1 for at least 72 hours with or without wash-out in NCI-H358 cells (Fig. 2B; Supplementary Fig. S4C). Similar effects were observed following treatment with SIAIS562055 in other KRAS-mutant and KRAS-WT cell lines (Fig. 2C; Supplementary Fig. S4D). Moreover, through an anchoring-independent three-dimensional growth inhibition assay, we found that SIAIS562055 significantly impeded the growth of KRAS-mutant cancer cells, while having a minimal effect on KRAS-WT cells (Fig. 2D). The IC_50_ values for NCI-H358, GP2d, HPAF-II, and SW620 were determined to be 2.4, 2.9, 16.9, and 3.9 nmol/L respectively, demonstrating superior efficacy compared with BI-3406 (Fig. 2D). To determine whether the effect of SIAIS560255 on cell proliferation depends on its impact on RAS activation, we performed G-LISA assays to evaluate the effect of SIAIS562055 on cellular RAS-GTP in both the KRAS-mutant and KRAS-WT cell lines. The results demonstrated that SIAIS560255 effectively suppressed RAS-GTP levels upon EGF stimulation in SOS1/KRAS-dependent MIA PaCa-2 (KRAS^G12C^) cells while exhibiting a negligible effect on SOS1/KRAS-independent BxPC-3 (KRAS-WT) cells (Supplementary Fig. S4E). Collectively, these data revealed that SIAIS560255 exerts significant inhibitory effects on KRAS-driven cancer cells.

A previous study has reported that concurrent inhibition of SOS1 and KRAS synergistically suppresses the proliferation of KRAS-mutant cancer cells (28). Consistently, our results showed enhanced cytotoxicity of SIAIS562055 combined with the KRAS^G12C^ inhibitor AMG510 in NCI-H358 cells, as evidenced by a CI value of 0.1 (Fig. 2E). Moreover, the combination of SIAIS562055 and MRTX1133, a known KRAS^G12D^ inhibitor, also yielded a strong synergistic antiproliferative effect on GP2d cells, with a CI value of 0.3 (Fig. 2E). The synergism between SIAIS562055 and KRAS^G12C^ inhibitors was verified by colony formation assays (Fig. 2F). In addition, SIAIS562055 treatment moderately inhibited ERK phosphorylation, which was enhanced in combination with AMG510 or MRTX849 (Fig. 2G). Similarly, cotreatment with SIAIS562055 and MRTX1133 demonstrated a synergistic inhibitory effect on ERK phosphorylation in GP2d cells (Fig. 2G). Taken together, these data suggest that SIAIS562055-induced degradation of SOS1 leads to the suppression of downstream signaling, which effectively inhibits the proliferation of KRAS-mutant cancer cells and shows synergistic effects when combined with KRAS inhibitors.

### SIAIS562055 induces pronounced tumor regression in combination with KRAS inhibitors

PK data were obtained to investigate the *in vivo* antitumor effects of SIAIS562055. As shown in Table 1, SIAIS562055 exhibited limited oral absorption and drug exposure after oral administration. However, i.p. or i.v. administration of SIAIS562055 significantly improved drug exposure and bioavailability. i.p. administration resulted in an acceptable PK profile with maximum concentration (*C*_*max*_ = 1,800 ng/mL) and high exposure (AUC_0–∞_ = 4,404 hours × ng/mL) in the samples.

**Table 1.** PK of SIAIS562055 after i.v., oral, or i.p. administration.

Parameter	SIAIS562055		
	i.v. (2 mg/kg)	Oral (10 mg/kg)	i.p. (10 mg/kg)
*C*_*max*_ (ng/mL)	3,280	30.1	1,800
*C*_0_ (ng/mL)	4,992	–	–
T_max_ (hours)	0.1	1.0	0.5
T_1/2_ (hours)	22.7	8.9	10.6
CL (mL/hours/kg)	750	–	–
MRT_0–t_ (hours)	4.5	6.2	5.9
MRT_0–inf_ (hours)	22.6	13.2	12.6
V_ss_ (mL/kg)	14,846	–	–
V_z_ (mL/kg)	21,178	–	–
AUC_0–t_ (ng/mL∙hours)	1,935	145	3,550
AUC_0–inf_ (ng/mL∙hours)	2,751	191	4,404
F (%)	–	1.3	32.5

Abbreviations: –, not applicable; AUC, area under the concentration–time curve; C, plasma concentration; CL, clearance; F, bioavailability; MRT, mean residence time; T_max_, time to maximum plasma concentration; T_1/2_, half-life; V_z_, apparent volume of distribution; V_ss_, apparent volume of distribution at steady state.

**Figure 3.**
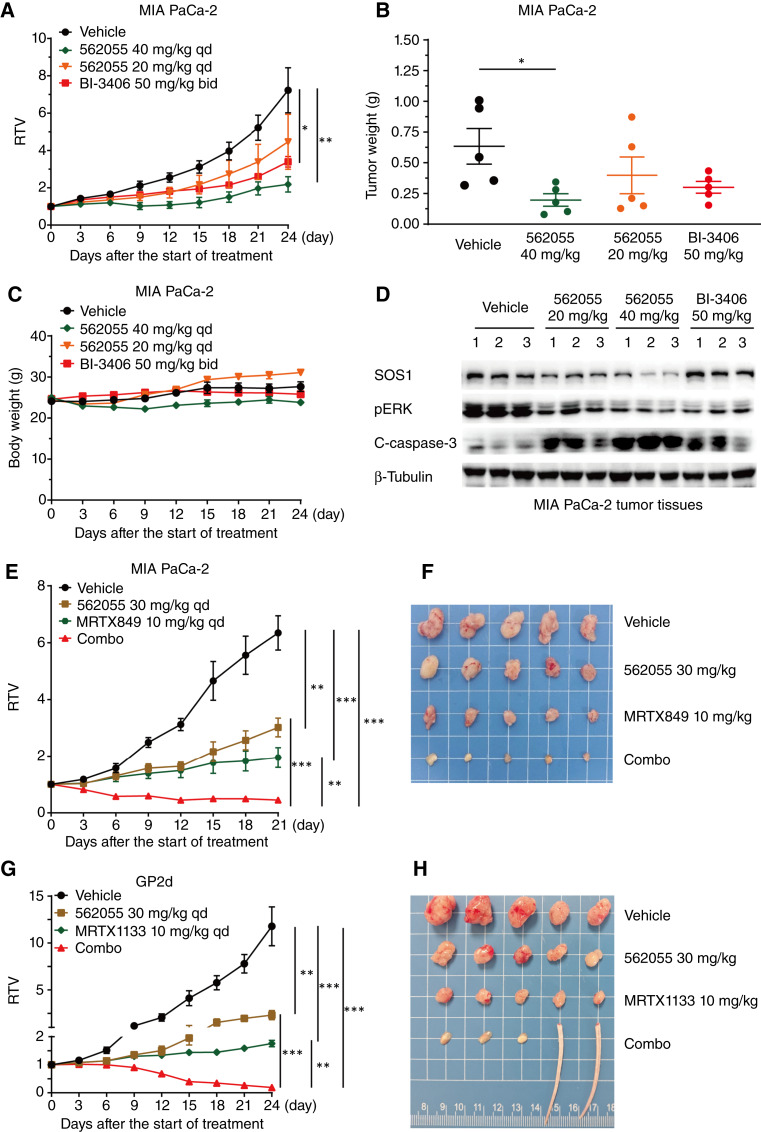
SIAIS562055 alone or coadministered with KRAS inhibitors suppresses tumor growth of KRAS-mutant xenografts *in vivo*. **A-C,** MIA PaCa-2–xenografted mice received vehicle, SIAIS562055 (20 or 40 mg/kg/day), or BI-3406 [50 mg/kg, twice a day (bid)], then relative tumor volumes (**A**), tumor weights (**B**), and mouse body weights (**C**) were determined at indicated times. **D,** Western blot analysis of SOS1, pERK, and cleaved caspase-3 in MIA PaCa-2 tumor tissues after treatment with SIAI562055 or BI-3406. **E** and **F,** MIA PaCa-2 xenograft-bearing mice received vehicle, SIAIS562055, and MRTX849, alone or in combination; the relative tumor volumes (**E**) and photos of tumors (**F**) are shown. **G** and **H,** GP2d xenograft-bearing mice received vehicle, SIAIS562055, and MRTX1133, alone or in combination; the relative tumor volumes (**G**) and photos of tumors (**H**) are shown. Data are presented as mean ± SEM, *n* = 5. Statistical significance was assessed using two-tailed unpaired Student *t* test. *, *P* < 0.05; **, *P* < 0.01; ***, *P* < 0.001. qd, once a day.

Considering its potent activity *in vitro* and favorable PK properties *in vivo*, SIAIS562055 was further investigated to examine its efficacy in a KRAS^G12C^-mutant pancreatic cancer cell xenotransplantation model. SIAIS562055 dose-dependently inhibited tumor growth in MIA PaCa-2 xenografts, showing good tolerability (Fig. 3A–C). At the end of the 24-day experimental period, daily injections of 20 and 40 mg/kg SIAIS562055 inhibited tumor growth by 45.9% and 81.3%, respectively, whereas twice daily injections of 50 mg/kg BI-3406 inhibited tumor growth by 62.0% (Fig. 3A). Furthermore, the effects of SIAIS562055 on SOS1 degradation and pERK inhibition in MIA PaCa-2 tumor tissues correlated with the *in vitro* results, as confirmed by the significant suppression of SOS1/KRAS/ERK signaling and induction of caspase-3 cleavage upon treatment with SIAIS562055 (Fig. 3D; Supplementary Fig. S5). Notably, cotreatment with SIAIS562055 (30 mg/kg) and MRTX849 (10 mg/kg) synergistically suppressed the tumor growth of MIA PaCa-2 xenograft models, achieving a tumor growth inhibition (TGI) of 110.1% and partial tumor regression (PR) of 100% (Fig. 3E and F; Supplementary Fig. S6A). Similarly, SIAIS562055 (30 mg/kg) monotherapy resulted in a TGI of KRAS^G12D^-mutant GP2d xenografts of 80.7%, whereas the inhibitory effect was further enhanced when combined with MRTX1133 (10 mg/kg), achieving a PR of 100% (Fig. 3G and H; Supplementary Fig. S6B). Importantly, these cotreatment regimens were well tolerated, with no obvious weight loss in mice in both MIA PaCa-2 and GP2d xenograft models (Supplementary Fig. S6C and S6D). These findings collectively suggest that SIAIS562055 effectively suppressed KRAS-mutant cancers both *in vitro* and *in vivo* and showed synergistic antitumor effects with KRAS inhibitors.

### SIAIS562055 counteracts resistance to KRAS inhibitors

**Figure 4.**
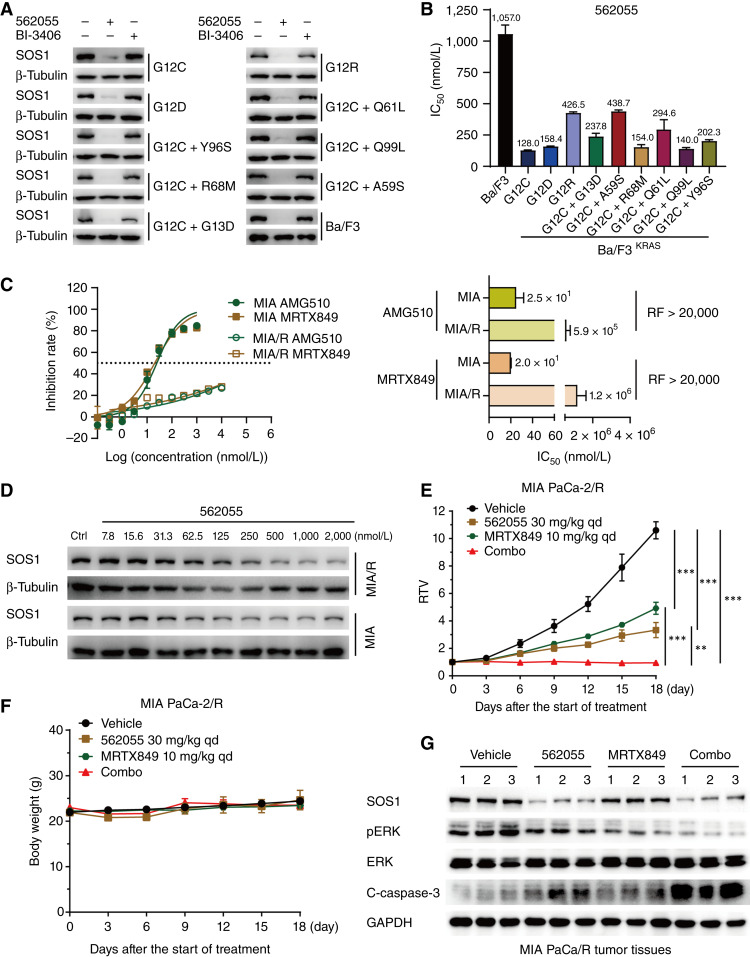
SIAIS562055 combined with KRAS inhibitors overcomes drug resistance. **A,** Western blot analysis of SOS1 in Ba/F3 cells transformed with or without KRAS mutation after treatment with SIAIS562055 (1,000 nmol/L) or BI-3406 (1,000 nmol/L) for 24 hours. **B,** Antiproliferative effects of SIAIS562055 on Ba/F3 cells transformed with or without KRAS mutations for 72 hours. Histogram shows the IC_50_ values of compounds in two-dimensional (2D) cultured cells. **C,** Cell proliferative inhibition of MIA PaCa-2 and MIA PaCa-2/R cells treated with AMG510 and MRTX849 for 72 hours. Histogram shows the IC_50_ values of compounds and resistance factors (RF) in 2D cultured cells. **D,** Western blot analysis of SOS1 by SIAIS562055 in MIA PaCa-2 and MIA PaCa-2/R cells for 72 hours. MIA PaCa-2/R xenograft-bearing mice received vehicle, SIAIS562055, and MRTX849, alone or in combination, then, the relative tumor volumes (**E**) and mouse body weights (**F**) were tested. qd, once a day. **G,** Western blot analysis of SOS1, pERK, and cleaved caspase-3 in MIA PaCa-2/R xenograft tumor tissues. Data about relative tumor volumes and body weights are presented as mean ± SEM, *n* = 5, whereas other statistical data are presented as mean ± SD, *n* = 3. **, *P* < 0.01; ***, *P* < 0.001.

Although KRAS^G12C^ inhibitors have exhibited promising efficacy in clinical trials, most patients develop acquired resistance due to genetic heterogeneity after monotherapy, secondary activation mutations of KRAS, or various compensatory mechanisms (12). In this study, we constructed Ba/F3 cell lines expressing different KRAS mutations (56) including secondary mutations that confer resistance to KRAS^G12C^ inhibitors (57). SIAIS562055 significantly reduced SOS1 levels (Fig. 4A) and specifically inhibited the proliferation of cells expressing various KRAS mutants (Fig. 4B). The IC_50_ values of SIAIS562055 in KRAS-mutant Ba/F3 cells ranged from 128.0 to 438.7 nmol/L, compared with that of parental cells (1057 nmol/L), indicating the relative selectivity for SIAIS562055 in KRAS-driven cells (Fig. 4B). To further investigate the potential effectiveness of SIAIS562055 in counteracting KRAS inhibitor resistance, we generated MIA PaCa-2/R cells by exposing parental cells to sequentially increasing concentrations of AMG510, which also displayed resistance to MRTX849, with a resistance factor of more than 20,000 (Fig. 4C). SIAIS562055 reduced SOS1 levels in a concentration-dependent manner in MIA PaCa-2/R cells, similar to its effect on the parental MIA PaCa-2 cells (Fig. 4D; Supplementary Fig. S7A). Notably, SIAIS562055 monotherapy inhibited MIA PaCa-2/R tumor growth by 76.3% (*P* < 0.001) and demonstrated even greater antitumor efficacy, with a TGI of 100.5%, when combined with the KRAS inhibitor MRTX849 (Fig. 4E). This cotreatment induced tumor regression in mice (PR = 60%; Fig. 4E) and was generally well-tolerated (Fig. 4F). Furthermore, in MIA PaCa-2/R tumor tissues, the co-administration of SIAIS562055 with MRTX849 resulted in significant degradation of SOS1, leading to a more pronounced reduction of ERK phosphorylation and enhanced cleavage of caspase-3 (Fig. 4G; Supplementary Fig. S7B and S7C). Taken together, SIAIS562055 exhibited synergistic effects with KRAS inhibitors and overcame resistance in KRAS-mutant cancer cells, making it a promising therapeutic candidate.

### SIAIS562055 induces SOS1 degradation and suppresses the proliferation of BCR–ABL^+^ CML cells

**Figure 5.**
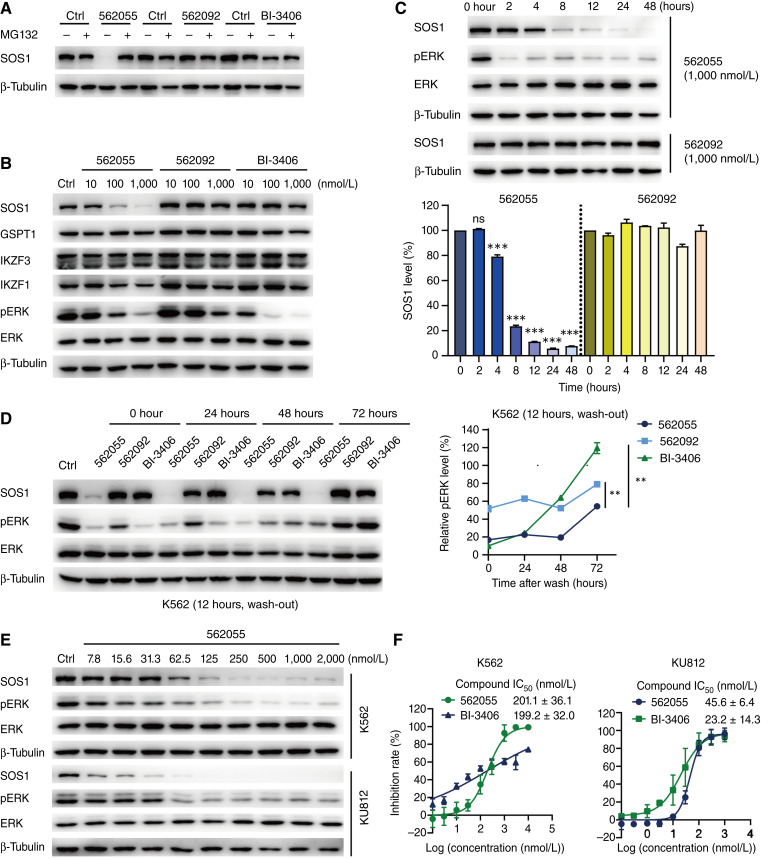
SIAIS562055 inhibits the proliferation of CML cells *in vitro*. **A,** Western blot analysis of SOS1 in K562 cells treated with SIAIS562055 (1,000 nmol/L), SIAIS562092 (1,000 nmol/L), or BI-3406 (1,000 nmol/L) for 24 hours, with or without the pretreatment of MG132 (1,000 nmol/L) for 1 hour. **B,** Western blot analysis of SOS1, pERK, GSPT1, and IKZF3 in K562 cells treated with SIAIS562055, SIAIS562092, or BI-3406 for 24 hours. **C,** Western blot analysis of SOS1 and pERK in K562 cells treated with a time gradient of SIAIS562055 or SIAIS562092. Histogram shows the relative SOS1 levels. **D,** Western blot analysis of SOS1 and pERK in K562 cells treated with SIAIS562055 (1,000 nmol/L), SIAIS562092 (1,000 nmol/L), or BI-3406 (1,000 nmol/L) for 12 hours, followed by wash-out and incubation with fresh media for another 0, 24, 48, and 72 hours. Line chart shows the relative pERK levels. **E,** Western blot analysis of SOS1 and pERK in K562 and KU812 cells treated with SIAIS562055 for 24 hours in a concentration gradient. **F,** Antiproliferative effects in K562 and KU812 cells after treatment with SIAIS562055 for 72 hours. Line charts show the IC_50_ values of two-dimensional culture. Data are shown as mean ± SD, *n* = 3. **, *P* < 0.01; ***, *P* < 0.001; ns, not significant.

It has been reported that SOS1, in addition to acting as a GEF for Ras protein, also contributes to BCR–ABL–mediated leukemogenesis in CML (58). In our study, we delved deeper into the impact of SIAIS562055 on SOS1 and downstream effectors in the BCR–ABL^+^ CML cell lines K562 and KU812. As expected, SIAIS562055 induced SOS1 degradation, which was almost completely reversed by the proteasome inhibitor MG132 in K562 cells, whereas neither SIAIS562092 nor BI-3406 caused SOS1 degradation (Fig. 5A). Consistent with the findings in KRAS-mutant cells, SIAIS562055 had little or no impact on the expression of GSPT1, IKZF3, and IKZF1 (51–53) in K562 cells (Fig. 5B). Besides, treatment with SIAIS562055 continued to degrade SOS1 protein and inhibit ERK phosphorylation for 48 hours (Fig. 5C). Additionally, the inhibitory activity persisted for at least 72 hours even after wash-out, whereas ERK phosphorylation was downregulated by BI-3406 or SIAIS562092 and returned to control levels 72 hours after drug removal (Fig. 5D). Similarly, SIAIS562055 concentration-dependently reduced the SOS1 and ERK signal in K562 and KU812 cells, with the DC_50_ values of 62.5 and 8.4 nmol/L, respectively (Fig. 5E; Supplementary Fig. S8A). As expected, SIAIS562055 inhibited the proliferation of K562 and KU812 cells, with IC_50_ values of 201.1 and 45.6 nmol/L, respectively (Fig. 5F). Subsequently, we tested whether the cytotoxicity of SIAIS562055 was attributed to its inhibition on the activation of RAC and RAS. SIAIS562055 treatment markedly reduced the levels of RAC-GTP and RAS-GTP in K562 cells (Supplementary Fig. S8B). Therefore, SIAIS562055 diminished SOS1 and its downstream signaling, leading to suppression of BCR–ABL–driven CML cell proliferation.

### SIAIS562055 enhances the sensitivity of CML cell to BCR–ABL inhibitors by SLC22A4-mediated cellular uptake transport

**Figure 6.**
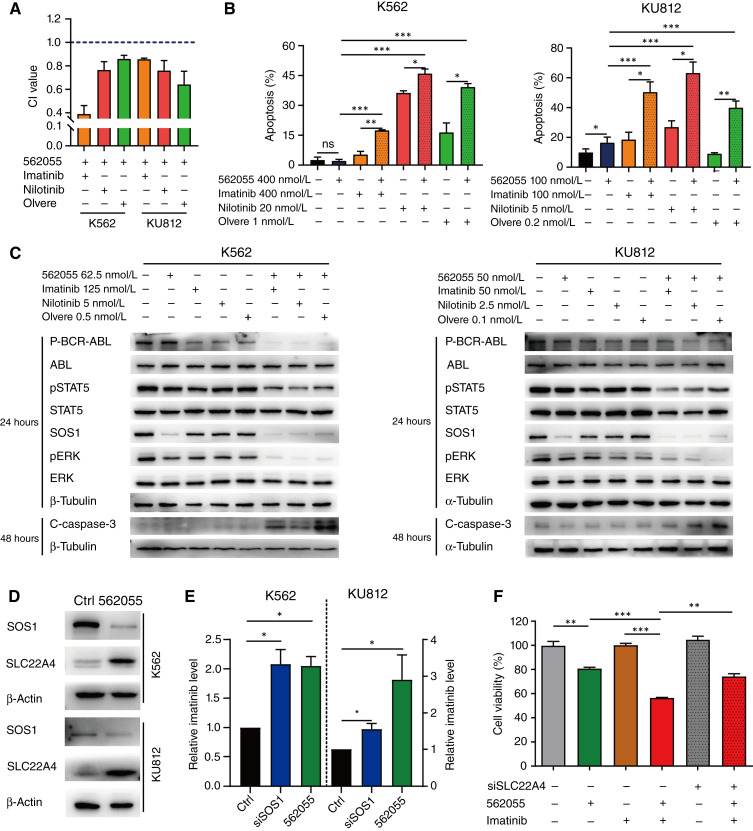
SIAIS562055 sensitizes CML cell lines to TKIs *in vitro*. **A,** SIAIS562055 and TKIs had synergistic effects on the proliferation of K562 and KU812 cells after treatment of 72 hours. Histogram shows the CI values. **B,** Cell apoptosis (Annexin V^+^) of the K562 and KU812 cells treated with SIAIS562055, TKIs such as imatinib, nilotinib, or olverembatinib (olvere), alone or in combination, for 48 hours. **C,** Western blot analysis of P-BCR–ABL, pSTAT5, SOS1, pERK, and cleaved caspase-3 in K562 and KU812 cells treated with SIAIS562055 or TKIs, alone or in combination. **D,** Western blot analysis of SLC22A4 in K562 and KU812 cells treated with SIAIS562055 (62.5 and 50 nmol/L, respectively) for 24 hours. **E,** High-performance liquid chromatography analysis examined the relative intracellular concentrations of imatinib (10 μmol/L in the media) in K562 and KU812 cells after being treated with SIAIS562055 (1,000 nmol/L, 24 hours) or SOS1 siRNAs (50 nmol/L, 48 hours). **F,** Cell viability of K562 treated with SLC22A4 siRNAs (25 nmol/L), SIAIS562055 (125 nmol/L), or imatinib (25 nmol/L), alone or in combination, for 72 hours was assessed by MTT assay. Data are presented as mean ± SD, *n* = 3. *, *P* < 0.05; **, *P* < 0.01; ***, *P* < 0.001; ns, not significant.

Given previous studies reporting the impact of SOS1 on the sensitivity of CML to the BCR–ABL inhibitor imatinib (30, 33), it would be interesting to explore whether targeting SOS1 by SIAIS562055 synergizes with TKIs to inhibit the proliferation of CML cells. Our results indicated that SIAIS562055, in combination with various BCR–ABL inhibitors, including imatinib, nilotinib, and olverembatinib, exhibited synergistic cytotoxicity in both K562 and KU812 cells, with CI values less than 1 (Fig. 6A; Supplementary Fig. S9). Additionally, flow cytometry results revealed that SIAIS562055 alone had limited proapoptotic effects on K562 and KU812 cells, whereas its combination with ABL inhibitors greatly promoted cell apoptosis (Fig. 6B). Western blot analyses further revealed that this cotreatment enhanced the suppression of ERK and STAT5 phosphorylation and induced caspases-3 cleavage in both K562 and KU812 cells (Fig. 6C; Supplementary Fig. S10). It has been reported that siRNA-mediated SOS1 depletion increases the level of the carrier protein SLC22A4, which is associated with imatinib uptake (33, 59–61). Consistently, SIAIS562055 caused SOS1 degradation and upregulated SLC22A4 as well (Fig. 6D). We speculated that this elevated level of SLC22A4 promotes the uptake of imatinib in CML cells, thereby enhancing the cellular response to imatinib treatment in CML. To confirm this, we used high-performance liquid chromatography to evaluate imatinib content in K562 and KU812 cells after treatment with SIAIS562055. The results indicated that in both cell lines, the cellular uptake of imatinib was prominently increased upon SIAIS562055 treatment (Fig. 6E). SOS1 knockdown also improved cellular imatinib absorption, consistent with previous reports (Fig. 6E; Supplementary Fig. S11A; ref. 33). Moreover, SLC22A4 knockdown partially reversed the synergistic effect between SIAIS562055 and imatinib on K562 cells (Fig. 6F; Supplementary Fig. S11B). Together, our results illustrated that SIAIS562055 and BCR–ABL inhibitors had synergistic antitumor effects on CML cells, which was associated with the upregulation of SLC22A4 caused by SIAIS562055.

### Combination of SIAIS562055 and imatinib exerts synergistic antitumor efficacy in mouse model and primary samples from patients with CML

**Figure 7.**
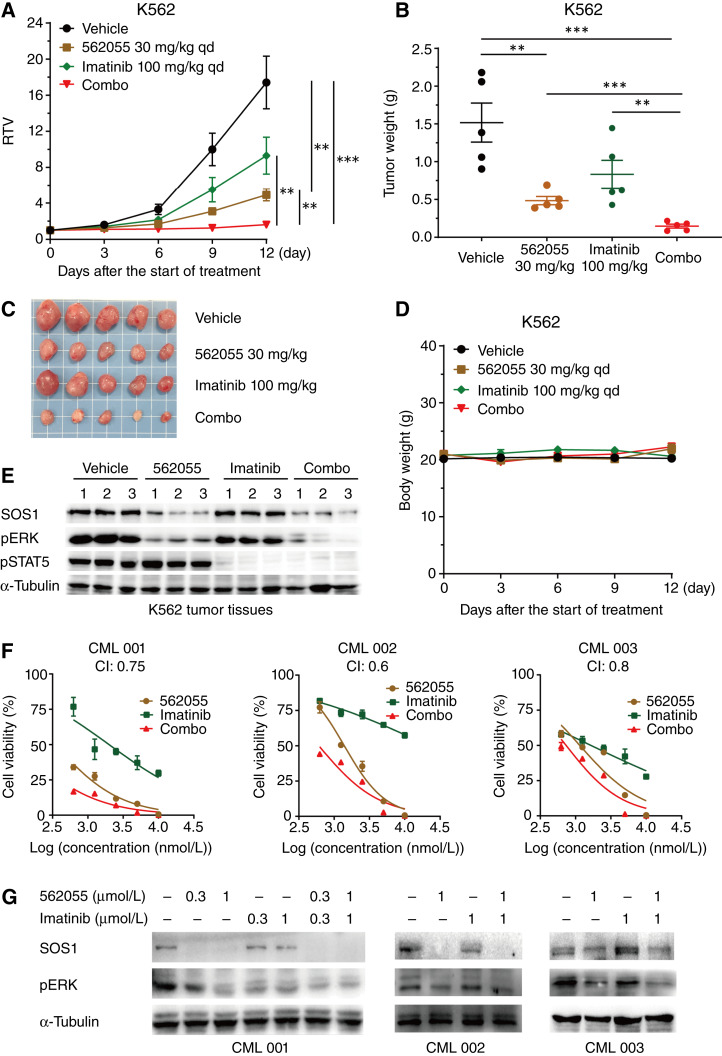
Combination of SIAIS562055 and TKIs synergistically inhibits the growth of CML xenografts *in vivo* and enhances cell death in primary samples from patients with CML. **A–D,** K562-xenografted mice received vehicle, SIAIS562055 (30 mg/kg/day), imatinib (100 mg/kg/day), or their combination; then, relative tumor volumes (**A**), tumor weights (**B**), photo of tumors (**C**), and mice body weights (**D**) were determined at indicated times. Data are presented as mean ± SEM, *n* = 5. **E,** Western blot analysis of SOS1, pERK, and pSTAT5 in K562 xenograft tumors after treatment with vehicle, SIAIS562055, imatinib, or their combination. Data are shown as mean ± SEM, *n* = 5. **F,** Cell viability of three primary BCR–ABL^+^ samples from patients with CML treated with varying doses of SIAIS562055 or imatinib, alone or in combination, for 72 hours was assessed by CellTiter-Glo assay. Data are presented as mean ± SD, *n* = 3. **G,** Western blot analysis of the indicated proteins in primary samples from patients with CML under 24-hour exposure to SIAIS562055 or imatinib, alone or in combination. Statistical significance was assessed using two-tailed unpaired Student *t* test. *, *P* < 0.05; **, *P* < 0.01; ***, *P* < 0.001. qd, once a day.

Based on the synergistic effects *in vitro*, we further investigated the antitumor efficacy of the combined treatment in the K562 xenograft model. SIAIS562055 (30 mg/kg) alone inhibited tumor growth by 76.7%, whereas imatinib (100 mg/kg) monotherapy exhibited a moderate TGI of 50.9% (Fig. 7A). Compared with the single-drug groups, cotreatment resulted in enhanced antitumor efficacy with a TGI of 96.3% and lighter tumor weights at the experimental endpoint (Fig. 7A–C), inducing tumor regression in mice (PR = 40%). Notably, the combination therapy did not induce significant changes in body weight or other signs of toxicity in nude mice (Fig. 7D). Moreover, SIAIS562055 and imatinib synergistically downregulated ERK and STAT5 activation in K562 xenograft tumors (Fig. 7E; Supplementary Fig. S12A). These data demonstrate that SIAIS562055 combined with imatinib enhanced the antitumor activity of the latter, further highlighting the beneficial advantages of PROTAC-based SOS1 degradation in CML therapy.

Using the GEPIA2 database (62), we analyzed the relationship between the expression of SOS1 and ABL or ERK (encoded by *MAPK1*) in CML. The results indicated a positive correlation between the expression of SOS1 and ABL1 or MAPK1 (*R* = 0.4 and 0.7, respectively) with *P* values less than 0.01 (Supplementary Fig. S12B). To further assess the possible clinical benefit of the combination strategy, we investigated the efficacy of the combination treatment in three patient-derived BCR–ABL^+^ CML samples obtained from diagnosis without prior treatment (patient information provided in Supplementary Table S2). CML 001 with T315I was detected in the blast phase (BP) and CML 002 was in the accelerated phase (AP). It has been acknowledged that patients progressing to AP and BP have a relatively poor prognosis, and the presence of T315I mutation is associated with imatinib resistance (63). Corroborating our findings, the CellTiter-Glo luminescent cell viability assay indicated that SIAIS562055 and imatinib c-treatment exerted a strong synergistic effect in inhibiting the proliferation of all three primary cells from patients with CML (CI, 0.8, 0.6, and 0.8, respectively; Fig. 7F). Moreover, SIAIS562055 potently reduced the level of SOS1 in these cells and significantly downregulated ERK phosphorylation when combined with imatinib (Fig. 7G). In summary, we provided evidence and rationale supporting preclinical assessment of the novel combinational targeted therapy for human CML and posited SOS1 PROTAC SIAIS562055 as a promising candidate for enhancing the therapeutic efficacy of BCR–ABL inhibitors.

## Discussion

SOS1 plays a pivotal role as a GEF for KRAS, binding to and activating GDP-bound KRAS proteins at their catalytic sites, facilitating the exchange of GDP for GTP (12). Consequently, inhibiting SOS1 activity in loading KRAS leads to indirect blockage of KRAS signaling. Several studies have also revealed that SOS1 is capable of activating GTPase RAC, a process critical for BCR–ABL–mediated leukemogenesis and malignant transformation (30, 33, 58). These findings imply that SOS1 is a promising target for cancer treatment, with several inhibitors being developed (28, 64). Nevertheless, BI-1701963, the first SOS1 inhibitor undergoing phase I clinical trials, has revealed some concerns, such as safety issues and limited monotherapy efficacy. The development of novel approaches to target SOS1 with improved pharmacologic attributes is a highly intriguing area.

PROTAC is an emerging protein degradation technology that induces degradation of specific proteins via the ubiquitin–proteasome system. This process is achieved by recruiting target proteins to E3 ligase for ubiquitination and eventual degradation by the 26S proteasome (36). Notably, PROTACs have been developed against many cancer targets, providing promising opportunities for the treatment of otherwise “undruggable” or “incurable” cancers (34). It has also been reported that SOS1 PROTAC exhibits potent antitumor activity in KRAS-mutant cancers (45, 65). In this study, we designed and synthesized a series of SOS1 PROTACs by uniquely connecting a CRBN ligand to BI-3406 analogs. Systematic modifications to the composition and linker length resulted in the discovery of SIAIS562055, which demonstrated potent SOS1-directed degradation activity. Targeting SOS1 has been shown to indirectly block KRAS signaling pathways, such as MEK/ERK and PI3K/AKT (57, 66, 67). Compared with the small-molecule SOS1 inhibitor BI-3406, SIAIS562055 led to more sustained and potent suppression of downstream signaling in KRAS-mutant cancers. Accordingly, the administration of SIAIS562055 once a day exhibited more potent antitumor activity than BI-3406 twice daily in KRAS-driven tumors. These findings indicate the potential of an intermittent dosing regimen in clinical applications for SIAIS562055, aiming to ensure reduced toxicity and increased potency compared with small-molecular inhibitors.

Exposing KRAS^G12C^-mutant cells to G12C inhibitors initially inhibits downstream signaling; however, long-term monotherapy results in feedback activation and compensatory pathways (12). Continuous suppression of the RAS pathway and improved effectiveness of KRAS inhibitors in KRAS^G12C^-mutant cancer models were achieved through vertical pathway inhibition (68). SOS1 inhibitors have been shown to sensitize cells to KRAS^G12C^ inhibitors in preclinical studies, and the combination therapy is currently under clinical investigation (28). Our study showed that cotreatment with SIAIS562055 and KRAS inhibitors further reduced ERK phosphorylation and inhibited cell proliferation. SIAIS562055 combined with either KRAS^G12C^ inhibitor MRTX849 or KRAS^G12D^ inhibitor MRTX1133 caused significant tumor regression, achieving a PR rate of 100%, without any noticeable weight loss in all mice. These findings suggest that SIAIS562055, either in monotherapy or in combination with KRAS inhibitors, can serve as a promising alternative with a favorable safety profile for the treatment of various KRAS-driven cancers.

In addition, drug resistance to KRAS inhibitors is an unavoidable challenge that significantly compromises therapeutic efficacy in clinical practice. Mechanisms include low allele frequency of hotspot mutations in small G proteins and secondary mutations in RAS or RAF, which lead to activation of MAPK pathway intermediates (12, 69). In this study, we revealed that SIAIS562055 effectively reduced SOS1 levels and selectively inhibited cell proliferation in a range of Ba/F3 cell lines expressing various KRAS mutants or G12C secondary mutations that confer acquired resistance (56), suggesting the potential application of SIAIS562055 for treating resistant patients with secondary mutations in clinical settings. Moreover, SOS1 inhibition-based combination therapy may be an effective approach for overcoming resistance (70, 71). Importantly, SIAIS562055-mediated SOS1 degradation overcame acquired resistance to KRAS inhibitors, leading to tumor regression in mouse xenograft models when combined with KRAS inhibitors. To sum up, SOS1 PROTAC may play a significant role in sensitizing KRAS-mutant cancers to KRAS-targeted therapeutics and in overcoming resistance.

BCR–ABL–driven leukemia is one of the most common hematologic neoplasms. It has been reported that SOS1 functions as a downstream node protein of BCR–ABL, participating in RAC–GEF catalysis. ABL-mediated SOS1 phosphorylation promotes RAC activation, contributing to leukemogenic effects driven by BCR–ABL, thereby underscoring the noteworthy role of SOS1 in CML progression (32, 58). Accordingly, our results showed that SIAIS562055 treatment markedly reduced the levels of RAC-GTP and RAS–GTP in BCR–ABL^+^ CML cells, continuously degraded SOS1, inhibited ERK phosphorylation, and retarded cell proliferation. Similar to the findings in KRAS-mutant cancers, the proteasome inhibitor MG132 rescued the loss of SOS1 upon SIAIS562055 treatment. SIAIS562055 did not affect the expression of CRBN neo-substrates GSPT1 and IKZF1/3, suggesting that SIAIS562055 specifically induced SOS1 reduction through proteasome-dependent degradation. Moreover, SIAIS562055 synergized with ABL inhibitors to enhance the downregulation of ERK and STAT5 phosphorylation, resulting in stronger antiproliferative effects and increased cell apoptosis. Importantly, cotreatment with SIAIS562055 and imatinib led to tumor regression in K562 xenograft models, without any observable drug-induced body weight loss.

Belonging to the carnitine/organic cation transporter family, SLC22A4 has been reported to facilitate the active uptake of imatinib into cells (59–61). Targeting SOS1 overcomes resistance to imatinib independent of BCR–ABL, through SLC22A4-mediated imatinib uptake in CML (33). In line with this, our study revealed that both SOS1 degradation by SIAIS562055 and SOS knockdown by siRNAs enhanced the intracellular content of imatinib along with the enhanced expression of SLC22A4 in BCR–ABL^+^ CML cells, indicating that downregulation of SOS1 has the potential to enhance the efficacy of imatinib in cases where TKI resistance is not solely linked to the BCR–ABL fusion gene. It is worth noticing that about 50% of resistance in CML is not related to BCR–ABL but to intrinsic factors such as CML stemness, which contributes to poor prognosis (23). Overall, the depletion of SOS1 by SIAIS562055 increased SLC22A4 expression and facilitated the uptake of BCR–ABL inhibitors in CML, providing support for the combination therapy to achieve better anticancer efficacy and overcome resistance. However, our study did not rule out the potential contribution of other factors in sensitizing CML cells to TKIs via SOS1 inhibition, necessitating further research to explore the underlying molecular mechanisms.

Patients with CML may progress through three distinct disease phases: chronic phase, AP, and BP (72). The use of TKIs for the treatment of CML has set a precedent for the development of cancer therapies. However, individuals with AP and BP CML, especially in the latter phase with the T315I mutation, still face a relatively poor prognosis despite TKI treatment, and available therapeutic options are severely limited (73). Remarkably, SIAIS562055 effectively degraded SOS1 protein, inhibited ERK phosphorylation, and exhibited antiproliferative activity in primary CML samples spanning three distinct disease phases, despite doses higher than those required for imatinib therapy in cell lines. Furthermore, combining SIAIS562055 with imatinib exhibited synergistic antitumor efficacy in primary samples from patients with, including those carrying the T315I mutation. These results favor that targeting SOS1 by PROTAC is a promising strategy for CML treatment, and novel combination therapies that combine SOS1 inhibition with TKIs will expand the therapeutic arsenal in advanced CML. However, further research and clinical trials are essential to validate these findings and to explore the clinical applications of SIAIS562055 in overcoming TKI resistance in CML.

In summary, we designed and synthesized a series of SOS1 PROTACs to develop effective antitumor drugs. Among them, SIAIS562055 emerged as a potent SOS1 PROTAC, exhibiting efficacy against not only KRAS-mutant cancer but also BCR–ABL–driven CML, both *in vitro* and *in vivo*, with evidence of robust and durable SOS1 degradation and downstream signal inhibition. The synthetic lethal interaction between SIAIS562055 and KRAS inhibitors overcame resistance and promoted tumor regression through enhanced ERK inhibition in KRAS-mutant cancers. Additionally, SIAIS562055 enhanced the entry of BCR–ABL inhibitors into CML cells through the upregulation of SLC22A4, followed by the inhibition of ABL phosphorylation and suppression of downstream signaling, which increased the sensitivity of the cells to BCR–ABL inhibitors. Our findings suggest that SOS1 PROTACs may potentially serve as a valuable option for exploring the biological functions of SOS1 and as a basis for developing novel antitumor drugs targeting KRAS-mutant cancers and BCR–ABL–driven leukemia. Future optimization of SOS1 affinity, linker types, and E3 ligands are crucial to ensure drug efficacy, improve bioavailability, and enhance tissue penetration in the development of SOS1-targeted therapy.

## Supplementary Material

Supplementary Data Supplementary figures and tables
