# Supplementary material for: Targeted Degradation of SOS1 Exhibits Potent Anticancer Activity and Overcomes Resistance in KRAS-Mutant Tumors and BCR–ABL–Positive Leukemia
Source: Cancer Res. 2024 Oct 22;85(1):101–17. doi: 10.1158/0008-5472.CAN-24-1093 (PMC11694061; doi:10.1158/0008-5472.CAN-24-1093)
Supplement: Supplementary Data — Supplementary figures and tables [file can-24-1093_supplementary_data_suppsd.pdf]

Supplementary Data for

Original Article

Targeted Degradation of SOS1 Exhibits Potent Anticancer Activity and Overcomes Resistance in KRAS-Mutant Tumors and BCR-ABL-Positive Leukemia

Table of contents

1. Supplementary tables and figures

Table S1-S2

Figure S1-S12

2. Chemistry experimental procedures and data

1. Supporting table and figures

Table S1. Screening of SOS1-PROTAC candidates for cell proliferation inhibition and SOS1 degradation in K562 cells.

Compound	Chemistry code	Linker (C_n)	IC_{50} (nM)	Degradation of SOS1 (%)	
				1.0 μM	0.1 μM
SIAIS562068	A1	C_2	2430.0 ± 946.8	29.4 ± 2.4	-19.2 ± 9.6
SIAIS561127	A2	C_3	2211.0 ± 299.1	10.8 ± 1.4	0.9 ± 5.7
SIAIS562069	A3	C_4	1223.0 ± 73.5	43.5 ± 2.2	16.7 ± 1.3
SIAIS562058	A4	C_5	1034.0 ± 85.0	51.9 ± 1.4	35.9 ± 1.7
SIAIS561122	A5	C_6	982.8 ± 59.7	41.8 ± 4.8	6.8 ± 0.5
SIAIS561128	A6	C_7	568.4 ± 0.9	42.8 ± 0.4	6.6 ± 2.0
SIAIS561123	A7	C_8	572.3 ± 34.4	11.1 ± 4.5	12.2 ± 0.3
SIAIS561124	A8	C_9	68.9 ± 17.9	38.8 ± 2.9	33.8 ± 5.5
SIAIS562054	A9	C_{10}	540.5 ± 152.0	72.1 ± 1.2	71.5 ± 0.7
SIAIS562023	A10	C_{11}	417.1 ± 83.9	85.7 ± 0.2	48.2 ± 2.0
SIAIS562055	A11	C_{12}	201.1 ± 36.1	95.8 ± 0.3	84.3 ± 0.5
SIAIS562092	A11-NC	C_{12}	936.7 ± 106.6	7.0 ± 5.1	N/A
BI-3406	N/A	N/A	199.2 ± 32.0	-1.1 ± 1.8	N/A
SIAIS561131	B1	C_1	17836.0 ± 3898.0	-2.3 ± 9.5	12.3 ± 6.5
SIAIS561132	B2	C_2	112.1 ± 4.2	-3.8 ± 4.2	-14.7 ± 10.2
SIAIS561133	B3	C_3	1674.0 ± 17.7	-4.0 ± 11.7	-26.7 ± 1.7
SIAIS561134	B4	C_4	3600.0 ± 102.5	14.2 ± 11.1	5.2 ± 12.8
SIAIS561135	B5	C_5	1850.0 ± 455.4	12.7 ± 6.3	11.3 ± 4.8
SIAIS561136	B6	C_6	2529.0 ± 260.9	49.3 ± 2.2	18.2 ± 6.2
SIAIS561139	B7	C_7	2748.0 ± 359.9	7.7 ± 4.3	-15.2 ± 2.0
SIAIS562080	B8	C_8	4952.0 ± 412.2	5.5 ± 3.2	-46.9 ± 11.0

Compound	Chemistry code	Linker (C_n)	IC_{50} (nM)	Degradation of SOS1 (%)	
				1.0 μM	0.1 μM
SIAIS561137	B9	C_9	3061.0 ± 1096.0	15.7 ± 3.0	3.6 ± 3.2
SIAIS561138	B10	C_{10}	8968.0 ± 146.4	11.0 ± 0.6	-14.6 ± 1.1
SIAIS562083	B11	C_{11}	4134.0 ± 340.1	35.5 ± 1.0	-5.4 ± 2.2
SIAIS561091	C1	C_2	1204.0 ± 65.8	27.0 ± 7.5	36.2 ± 5.7
SIAIS561084	C2	C_3	1313.0 ± 176.1	-19.5 ± 2.2	7.3 ± 8.5
SIAIS562056	C3	C_4	2242.0 ± 101.8	27.4 ±14.6	-22.3 ± 0.3
SIAIS561071	C4	C_5	1751.0 ± 306.2	24.8 ± 24.5	20.3 ± 19.4
SIAIS561082	C5	C_6	998.4 ± 323.3	44.1 ± 13.7	17.7 ± 20.6
SIAIS561089	C6	C_7	1848.0 ± 120.2	43.0 ± 14.5	28.5 ± 8.3
SIAIS561090	C7	C_8	1130.0 ± 91.2	-5.3 ± 1.0	-19.2 ± 1.8
SIAIS561085	C8	C_9	1566.0 ± 413.0	36.2 ± 4.9	12.5 ± 1.3
SIAIS562017	C9	C_{10}	2677.0 ± 274.4	39.2 ± 0.2	33.7 ± 4.1
SIAIS562019	C10	C_{11}	846.3 ± 157.3	38.7 ± 1.1	4.6 ± 1.8
SIAIS562020	C11	C_{12}	822.3 ±83.0	86.4 ± 0.4	30.4 ± 0.3
SIAIS561092	D1	C_1	7285.0 ± 1008.0	8.1 ± 2.3	-26.5 ± 8.3
SIAIS561093	D2	C_2	16412.0 ± 2444.0	24.7 ± 4.4	19.9 ± 1.8
SIAIS561094	D3	C_3	7134.0 ± 224.2	35.3 ± 0.6	32.9 ± 0.4
SIAIS561086	D4	C_4	7689.0 ± 91.2	33.5 ± 4.7	22.7 ± 1.7
SIAIS561087	D5	C_5	6506.0 ± 514.1	39.9 ± 0.9	53.5 ± 0.6
SIAIS561095	D6	C_6	5230.0 ± 1391.0	3.9 ± 4.7	26.7 ± 3.0
SIAIS561096	D7	C_7	1551.0 ± 71.4	49.7 ± 0.9	57.2 ± 0.7
SIAIS562079	D8	C_8	7083.0 ± 215.7	49.7 ± 14.0	44.4 ± 6.5
SIAIS561097	D9	C_9	15113.0 ± 2045.0	-20.9 ± 9.0	-10.2 ± 5.5
SIAIS561098	D10	C_{10}	11482.0 ± 514.8	17.0 ± 2.2	1.1 ± 1.0
SIAIS562082	D11	C_{11}	4676.0 ± 1207.0	71.0 ± 1.6	21.3 ± 0.5

Notes: C_n represents the count of carbon atom in the linkers. SIAIS562068 to SIAIS562055, SIAIS561131 to SIAIS562083, SIAIS561091 to SIAIS562020, and SIAIS561092 to SIAIS562082 share similar linkers, respectively.

Table S2 Characteristics of clinical and biological aspects in primary CML patient samples.

CML Patient	Age	Gender	Disease phase	BCR-ABL transcript
001	16	Male	Blast phase	T315I
002	20	Male	Accelerated phase	P210
003	48	Male	Chronic phase	P210

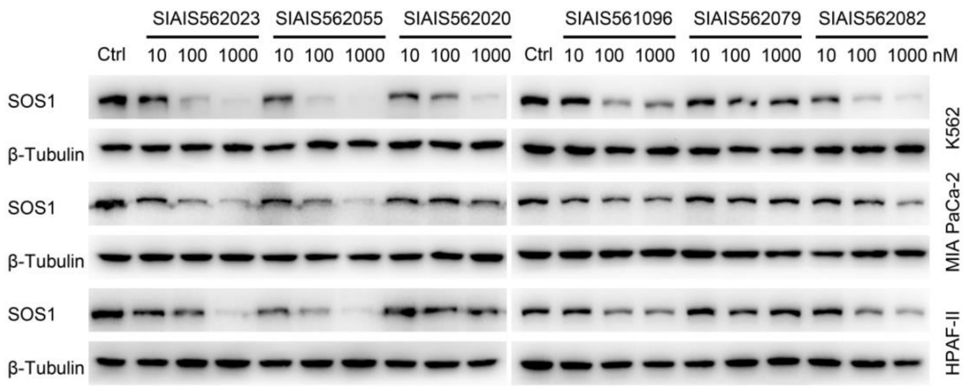

Figure S1.

The degradation of SOS1 by PROTACs in K562, MIA PaCa-2 and HPAF-II cells after 24 h of treatment.

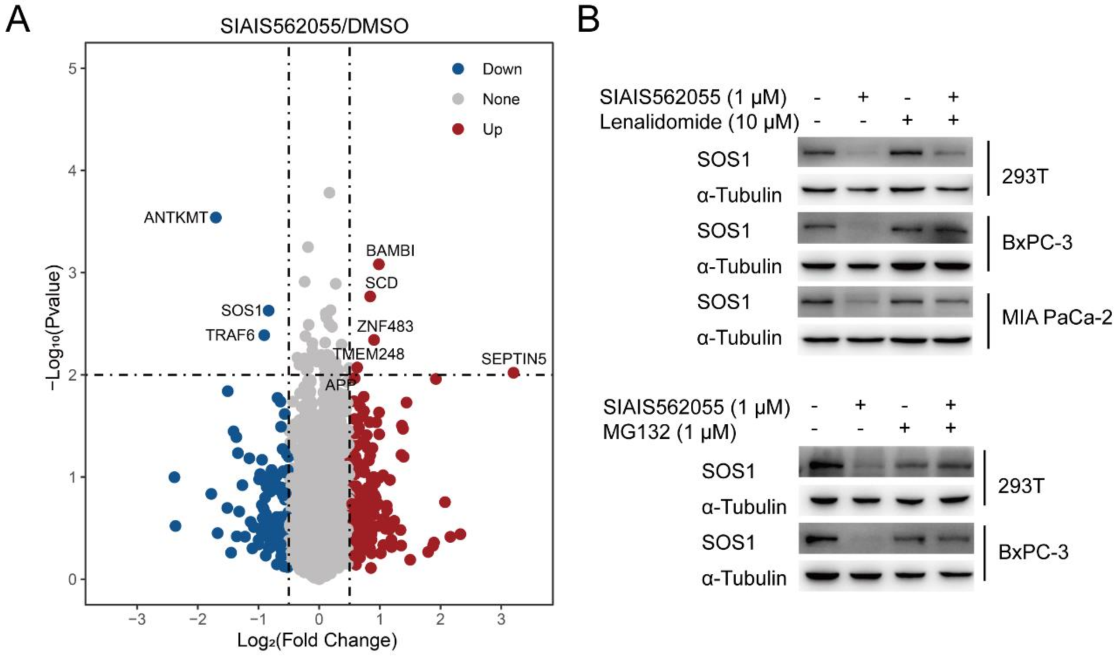

Figure S2.

SIAIS562055 degrades SOS1 selectively. **A**) Global proteomic analyses of MIA PaCa-2 cells after treatment with 1 μM SIAIS562055 for 16 h. **B**) Western blot analysis for effects of CRBN ligands and proteasome inhibitors on SOS1 degradation. 293T and BxPC-3 cells (KRAS-WT) and MIA PaCa-2 (KRAS^{G12C}) cells were pre-treated with 10 μM lenalidomide or 1 μM MG132 for 1 h, followed by treatment with 1 μM SIAIS562055 for an additional 24 h.

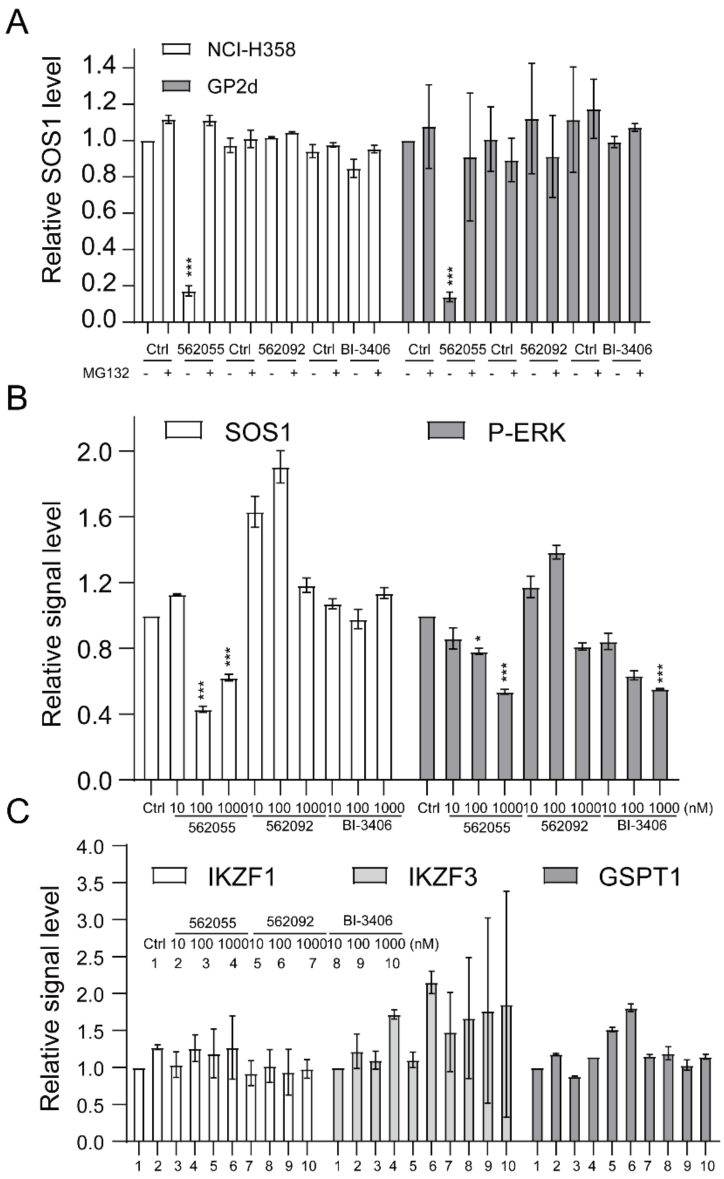

Figure S3.

The relative protein levels of SOS1, P-ERK, IKZF1/3 and GSPT1 in Fig. 1F and 1G. **A**) The relative SOS1 levels in NCI-H358 and GP2d cells treated with SIAIS562055 (562055 for short, 1000 nM), SIAIS562092 (562092 for short, 1000 nM) and BI-3406 (1000 nM) for 24 h with or without pretreatment of MG132 (1000 nM) for 1 h. **B**) The relative levels of SOS1 and P-ERK in NCI-H358 cells treated with SIAIS562055, SIAIS562092 or BI-3406 for 24 h. **C**) The relative levels of IKZF1/3 and GSPT1 in NCI-H358 cells treated with SIAIS562055, SIAIS562092 or BI-3406 for 24 h. Data are presented as mean ± SD, n = 3. Statistical significance was assessed using two-tailed unpaired Student's t test. *P < 0.05, **P < 0.01, and ***P < 0.001.

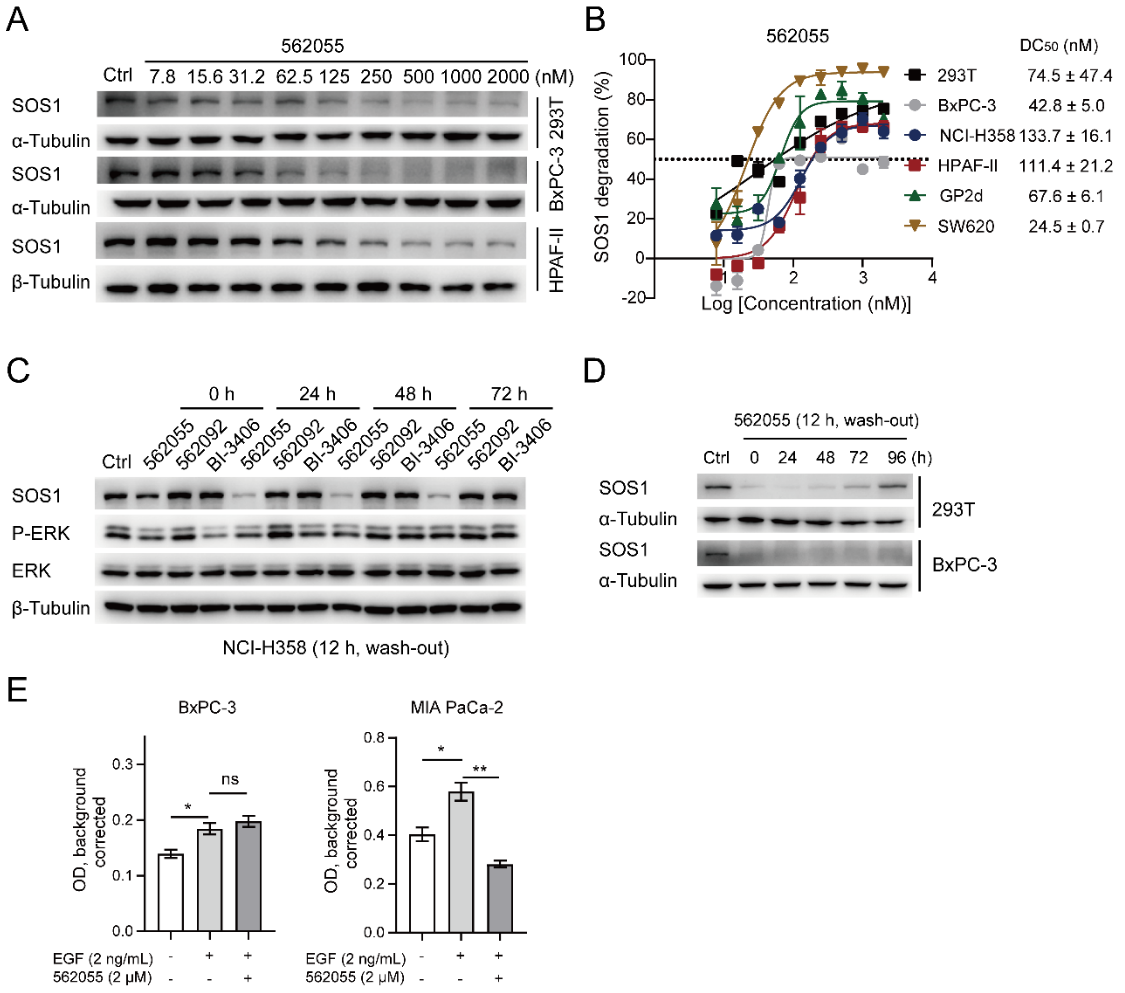

Figure S4.

The effects of SIAIS562055 on SOS1, P-ERK and RAS-GTP levels in KRAS-WT or KRAS-mutant cells. **A**) Western blot analysis of SOS1 in KRAS-WT (293T and BxPC-3) and KRAS-mutant HPAF-II cells treated with a concentration gradient of SIAIS562055 for 24 h. **B**) Degradation of SOS1 by SIAIS562055 in KRAS-WT cells (BxPC-3 and 293T) and KRAS-mutant cells (NCI-H358, GP2d, HPAF-II, and SW620) for 24 h. Data were mean ± SD, n = 3. **C**) Wash out experiments to examine the persistent effects on SOS1 degradation in NCI-H358. Western blot analysis of SOS1 and P-ERK in NCI-H358 cells treated with SIAIS562055, SIAIS562092, or BI-3406 for 12 h, followed by wash-out periods of 0, 24, 48 and 72 h. **D**) Western blot analysis of SOS1 in 293T and BxPC-3 cells after treatment with SIAIS562055 (1000 nM) for 12 h, followed by wash-out periods of 0, 24, 48, 72 and 96 h. **E**) Effects of SIAIS562055 on RAS-GTP levels. BxPC-3 (KRAS WT) and MIA PaCa-2 (KRAS G12C) cells were starved in media with 0.5% FBS with or without 2 μM SIAIS562055 for 24 h, then treated with 2 ng/mL EGF for an additional 2 minutes followed by the detection of RAS-GTP levels through GLISA. Data are presented as mean ± SD, n = 3. Statistical

significance was assessed using two-tailed unpaired Student's t test. *P < 0.05, **P < 0.01, and ***P < 0.001.

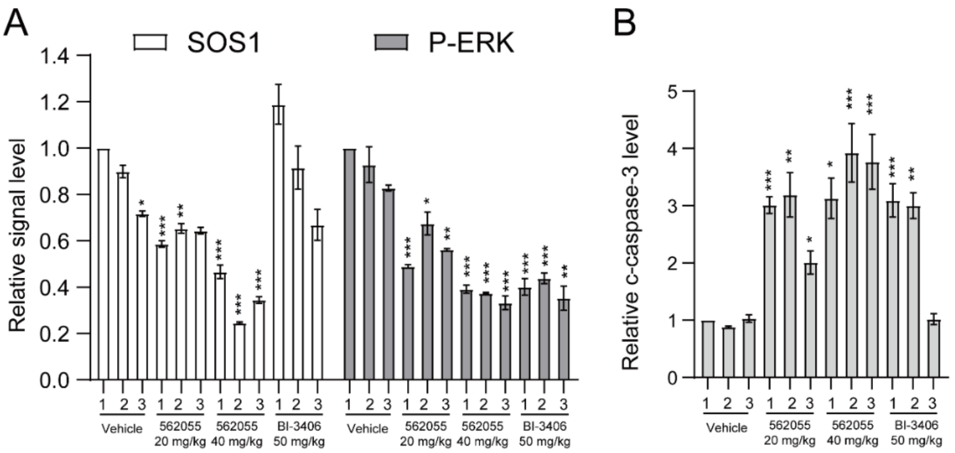

Figure S5.

Relative protein levels of SOS1, P-ERK and cleaved caspase-3 in Fig. 3D. Relative levels of SOS1 and P-ERK (**A**), and cleaved caspase-3 (**B**) in MIA PaCa-2 tumor tissues after treatment with SIAIS562055 (20 mg/kg or 40 mg/kg) and BI-3406 (50 mg/kg). Data are presented as mean ± SD, n = 3. Statistical significance was assessed using two-tailed unpaired Student's t test. *P < 0.05, **P < 0.01, and ***P < 0.001.

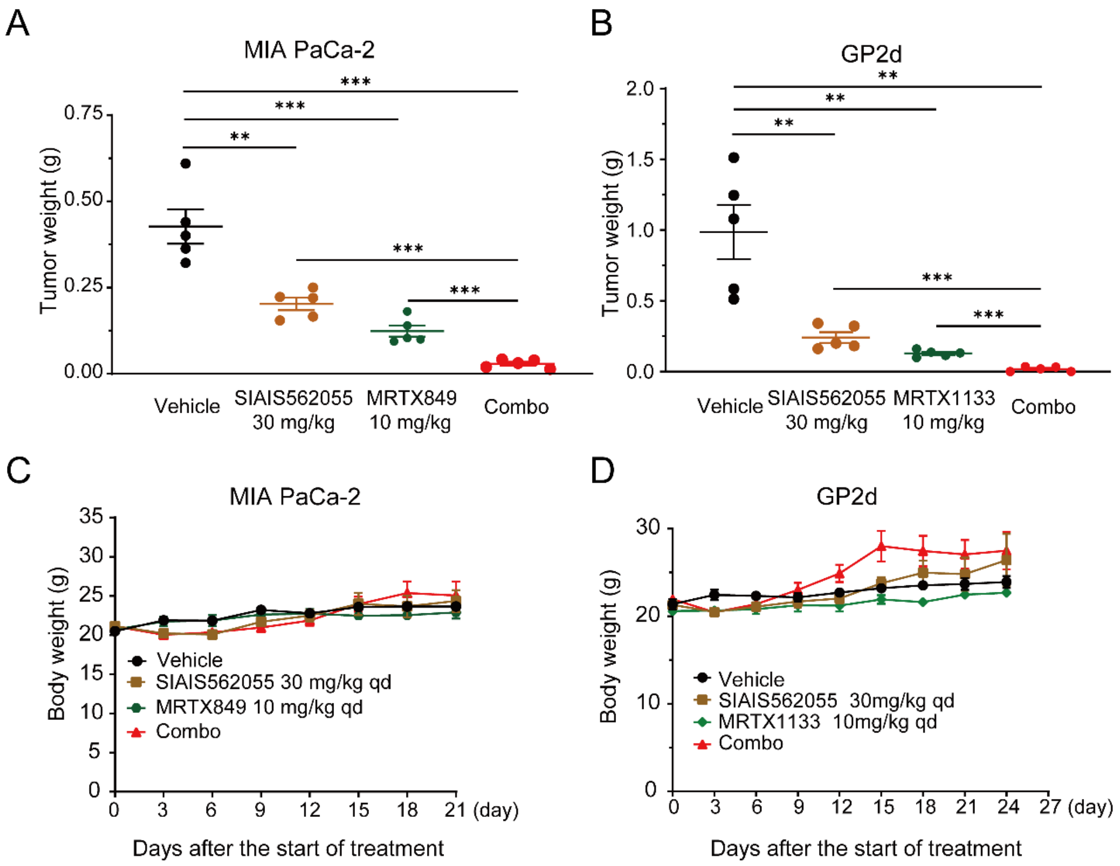

Figure S6.

Combination of SIAIS562055 and KRAS inhibitors are efficacious and well tolerated. **A**) Tumor weights of MIA PaCa-2 xenograft-bearing mice treated with vehicle, 30 mg/kg SIAIS562055, 10 mg/kg MRTX849 alone, or the combination. **B**) Tumor weights of GP2d xenograft-bearing mice treated with vehicle, 30 mg/kg SIAIS562055, 10 mg/kg MRTX1133 alone or the combination. **C**) Body weights of mice from the MIA PaCa-2 xenograft study. **D**) Body weights of mice from the GP2d xenograft study. Data are presented as mean ± SEM, n = 5. Statistical significance was assessed using two-tailed unpaired Student's t test. *P < 0.05, **P < 0.01, and ***P < 0.001.

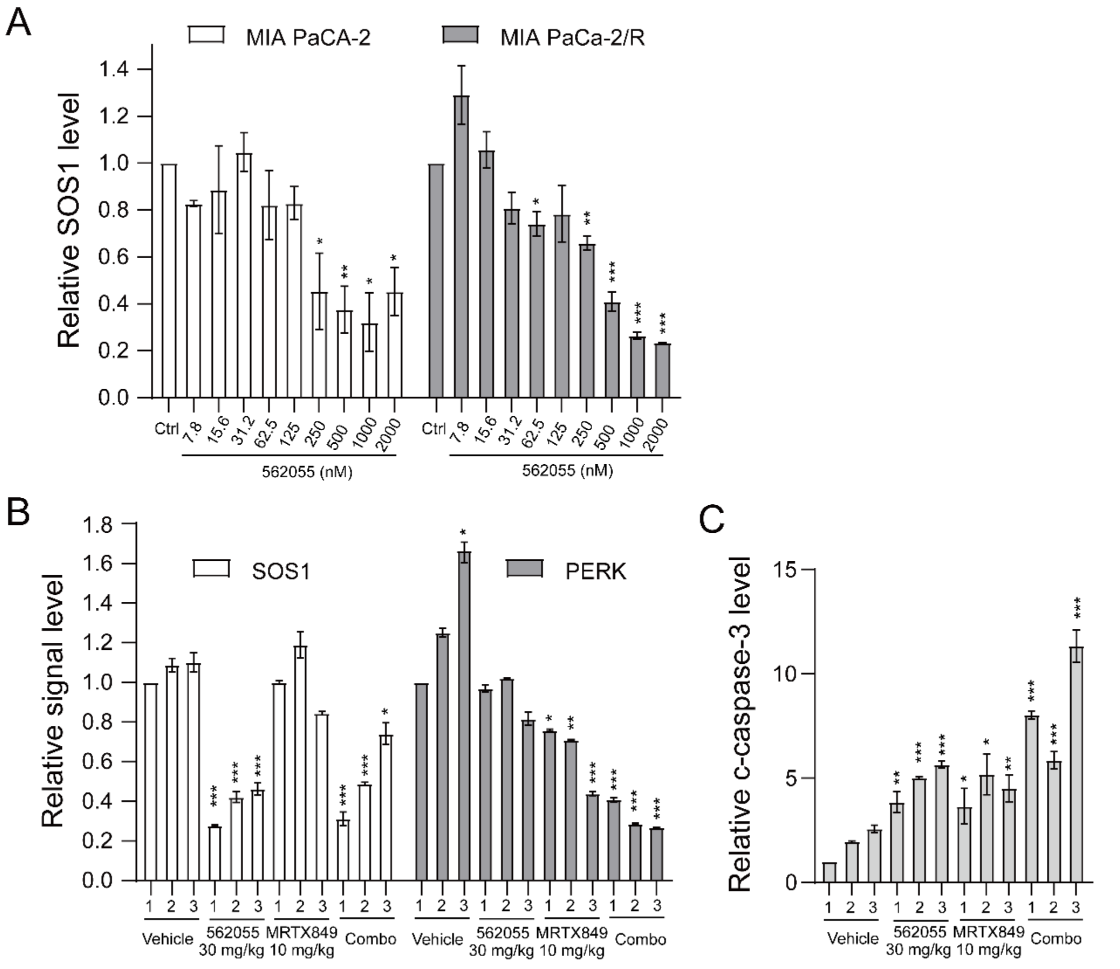

Figure S7.

Relative protein levels of SOS1, P-ERK and cleaved caspase-3 in Fig. 4D and 4G. **A**) Relative SOS1 level in MIA PaCa-2 and MIA PaCa-2/R cells after treatments of SIAIS562055 for 24 h. Relative levels of SOS1 and P-ERK (**B**) and cleaved caspase-3 (**C**) in MIA PaCa-2/R xenografts after treatment with SIAIS562055 (30 mg/kg), MRTX849 (10 mg/kg) alone, or in combination. Data are presented as mean ± SD, n = 3. Statistical significance was assessed using two-tailed unpaired Student's t test. *P < 0.05, **P < 0.01, and ***P < 0.001.

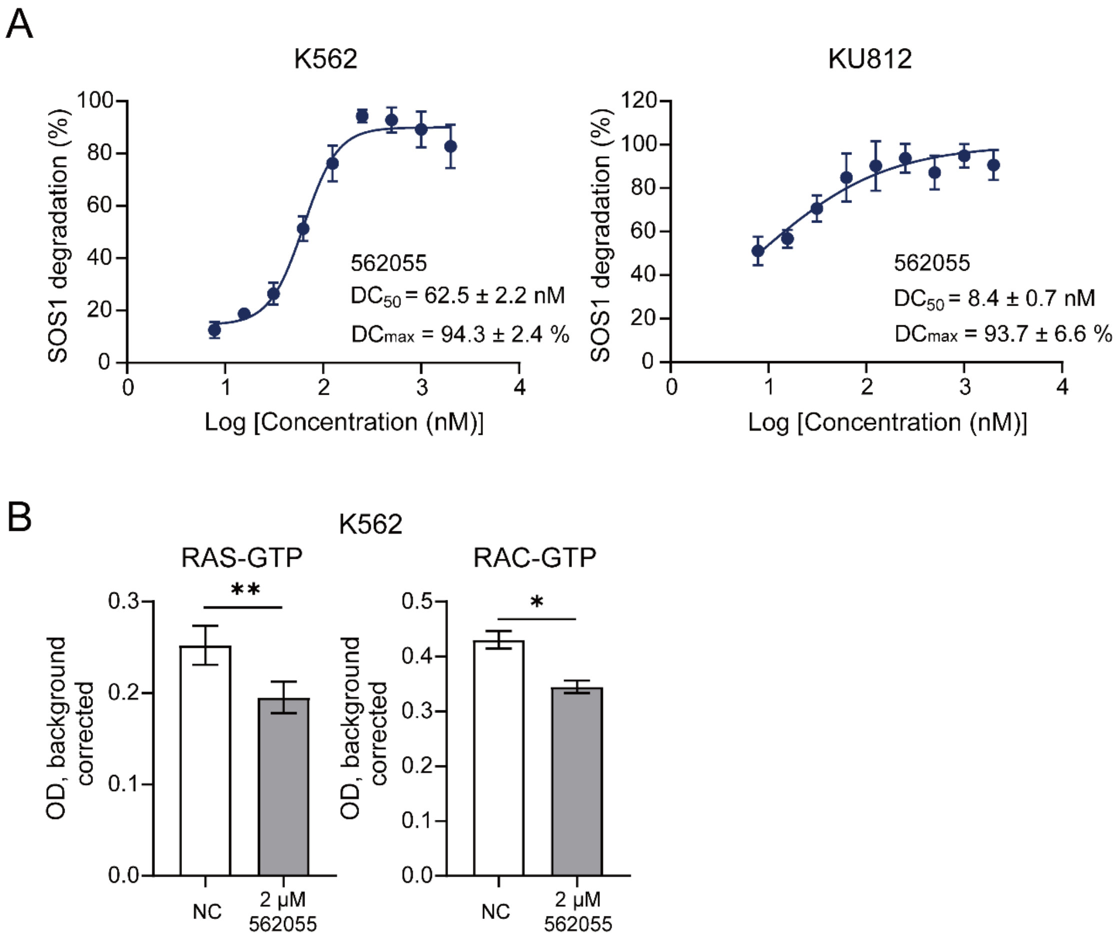

Figure S8.

The effects of SIAIS562055 on SOS1 and RAS/RAC-GTP levels in CML cells. **A**) Degradation of SOS1 by SIAIS562055 in K562 and KU812 cells. Line charts showed the relative SOS1 levels. **B**) K562 cells were serum-starved in media containing 0.5% BSA with or without 2 μM SIAIS562055 for 24 h, followed by the detection of RAS-GTP and RAC-GTP levels using GLISA. Data are presented as mean ± SD, n = 3. Statistical significance was assessed using two-tailed unpaired Student's t test. *P < 0.05, **P < 0.01, and ***P < 0.001.

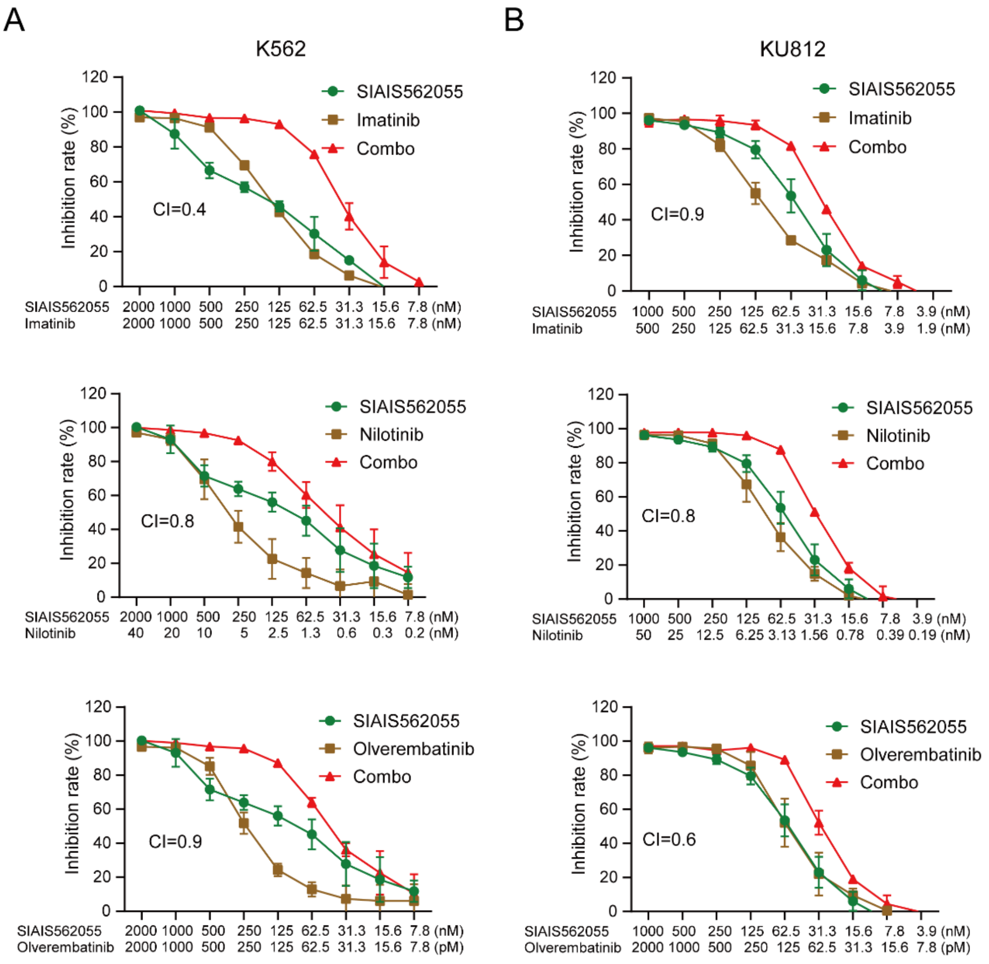

Figure S9.

SIAIS562055 sensitizes CML cells to TKIs *in vitro*. Line charts showed the inhibition rates of SIAIS562055, TKIs, or the combinations on K562 (**A**) or KU812 cells (**B**) after treatments of 72 h, respectively. Data are shown as mean ± SD, n = 3.

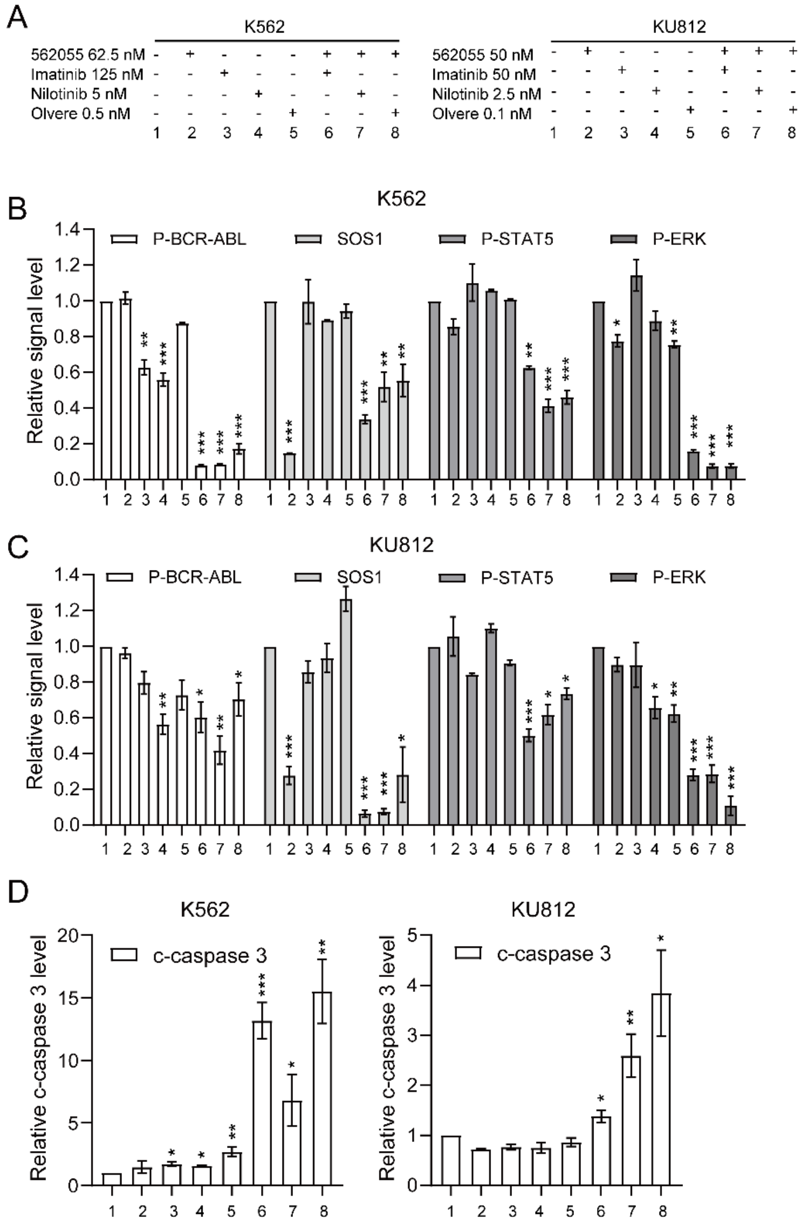

Figure S10.

Relative protein levels of P-BCR-ABL, SOS1, P-STAT5, P-ERK and cleaved caspase-3 in Fig. 6C. **A**) Compounds were used at specified concentrations in K562 or KU812 cells. **B**) Relative levels of P-BCR-ABL, SOS1, P-STAT5 and P-ERK in K562 cells after treatments described in (A) for 24 h. **C**) Relative levels of P-BCR-ABL, SOS1, P-STAT5 and P-ERK in KU812 cells after treatments described in (A) for 24 h. **D**) Relative cleaved caspase-3 level in K562 and KU812 cells after treatments described in (A) for 48 h. Data are presented as mean ± SD, n = 3. Statistical significance was assessed using two-tailed unpaired Student's t test. $^{*}P < 0.05$, $^{**}P < 0.01$, and $^{***}P < 0.001$.

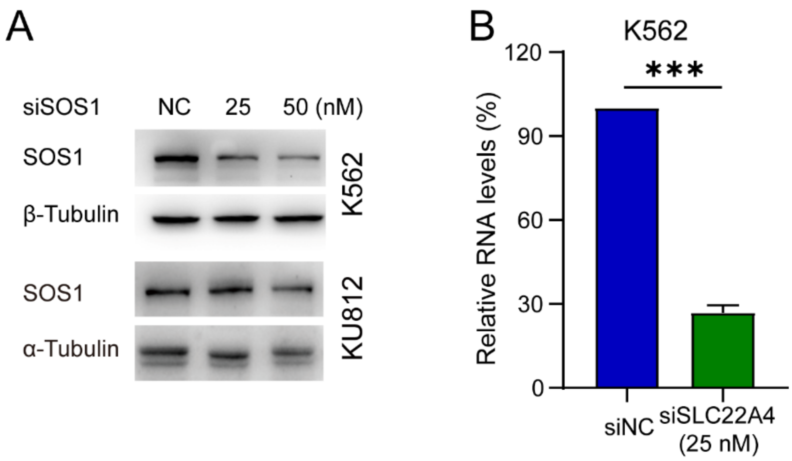

Figure S11.

siRNA-mediated depletion of SOS1 or SLC22A4 in CML cell lines. **A**) The efficacy of siRNA-mediated SOS1 knockdown in K562 and KU812 cells. **B**) The efficacy of siRNA-mediated SLC22A4 knockdown in K562 cells. Samples were collected after 48 h of treatments. Data are presented as mean ± SD, n = 3. Statistical significance was assessed using two-tailed unpaired Student's t test. *P < 0.05, **P < 0.01, and ***P < 0.001.

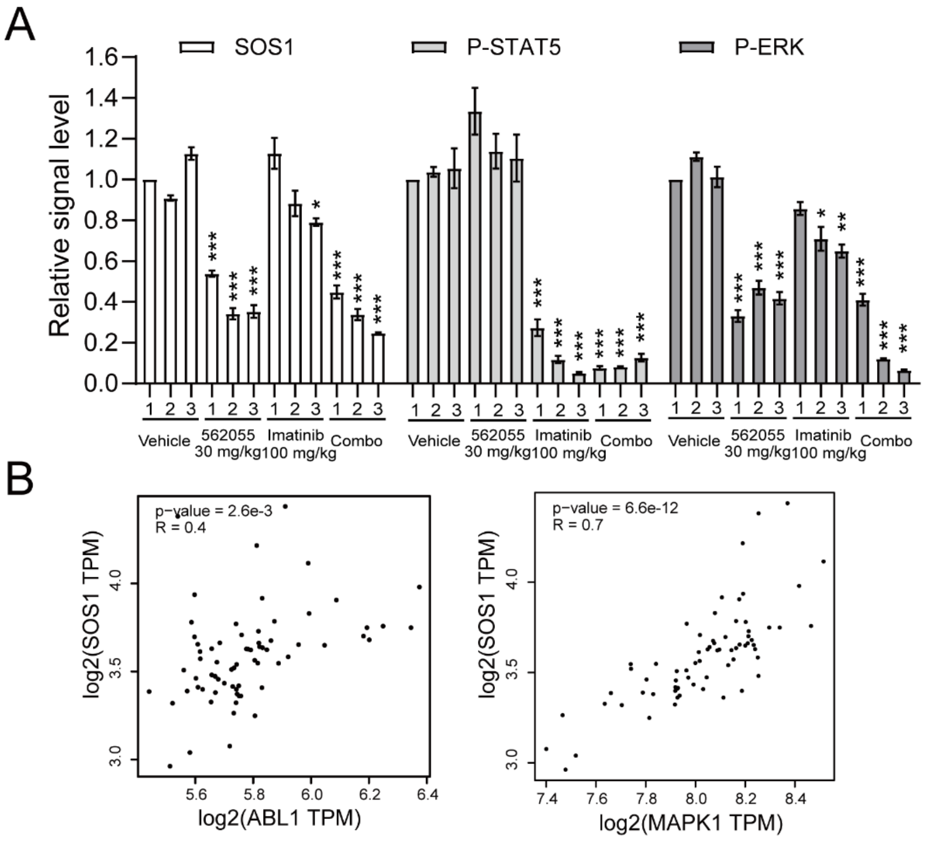

Figure S12.

Relative protein levels of SOS1, P-STAT5 and P-ERK in Fig. 7D and relationships of SOS1 with the expression of ABL1 and MAPK1 in CML. **A**) Relative SOS1, P-STAT5 and P-ERK levels in K562 xenografts after treatments with SIAIS562055 (30 mg/kg), imatinib (100 mg/kg), alone or in combination. Data are presented as mean ± SD, n = 3. Statistical significance was assessed using two-tailed unpaired Student's t test. *P < 0.05, **P < 0.01, and ***P < 0.001. **B**) The expression of SOS1 positively correlates with the expression of ABL1 or MAPK1 in CML.

2. Chemistry experimental procedures and data

2.1 General information

The reactions for the synthesis of the BI-3406 analogues are outlined in Scheme 1. The staring materials were commercially available. As shown in Scheme 1, the compound **S2-1** was synthesized by nucleophilic substitution between **S1-1** and 6-hydroxy-7-methoxy-2-methylquinazolin-4(3H)-one in the presence of Cs_2CO_3 in N,N-dimethylformamide (DMF) at 100℃ for 12 h. Then the intermediate **S3-1** was obtained by reaction of **S2-1** with 2,4,6-triisopropylbenzenesulfonyl chloride and 4-dimethylaminopyridine (DMAP) in dichloromethane (DCM) at room temperature for 12 h. A substitution reaction was performed with the (R)-3-(1-aminoethyl)-5-(trifluoromethyl)aniline in the present of triethylamine (TEA) in dimethylsulfoxide (DMSO) at 90℃ for 12 h to give compound **S4-1**. The final compound **S5-1** was obtained after removal of the Boc protecting group with triflfluoroacetic acid (TFA). Furthermore, the compound **S5-2** was synthesized from a similar route.

Scheme 1 Synthesis of BI-3406 analogues.

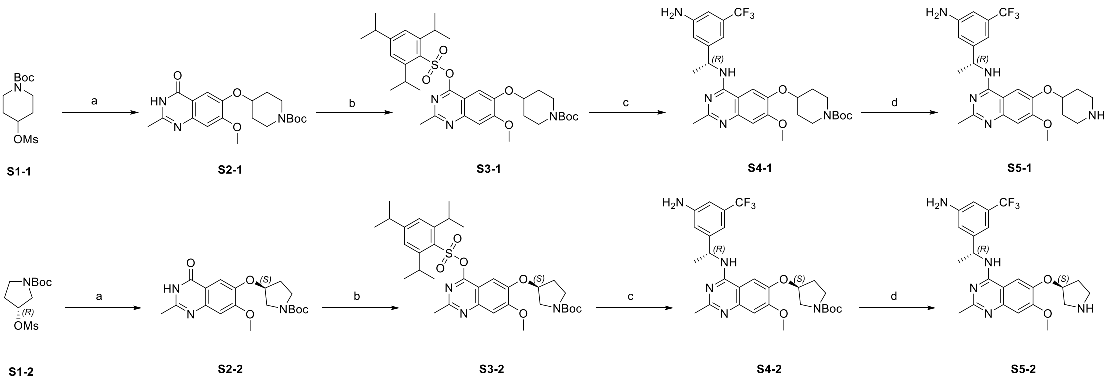

Reagents and conditions: (a) 6-hydroxy-7-methoxy-2-methylquinazolin-4(3H)-one,Cs_2CO_3, N,N-dimethylformamide (DMF), 100 ℃ , 12h; (b) 2,4,6-triisopropylbenzenesulfonyl chloride, 4-dimethylaminopyridine (DMAP), Triethylamine (TEA), dichloromethane (DCM), rt, 12h; (c) (R)-3-(1-aminoethyl)-5-(trifluoromethyl)aniline, TEA, dimethyl sulfoxide (DMSO), 90℃, 12h.; (d) trifluoroacetic acid (TFA), DCM, rt, 1-2h.

Linker synthesis

Lenalidomide derivatives were synthesized according to patent WO2019196812 and outlined in Scheme 2. Firstly, lenalidomide was transformed into **S8** through thiolation reaction and subsequent debenzylation reaction. Then lenalidomide-based linkers **S9** were prepared by nucleophilic substitution reaction between **S8** and alkyl dibromides of different chain lengths, and **S10** were prepared by nucleophilic substitution reaction of **S8** with different chain length brominated carboxylate esters and subsequent hydrolysis reaction.

Scheme 2 Synthesis of lenalidomide derivatives.

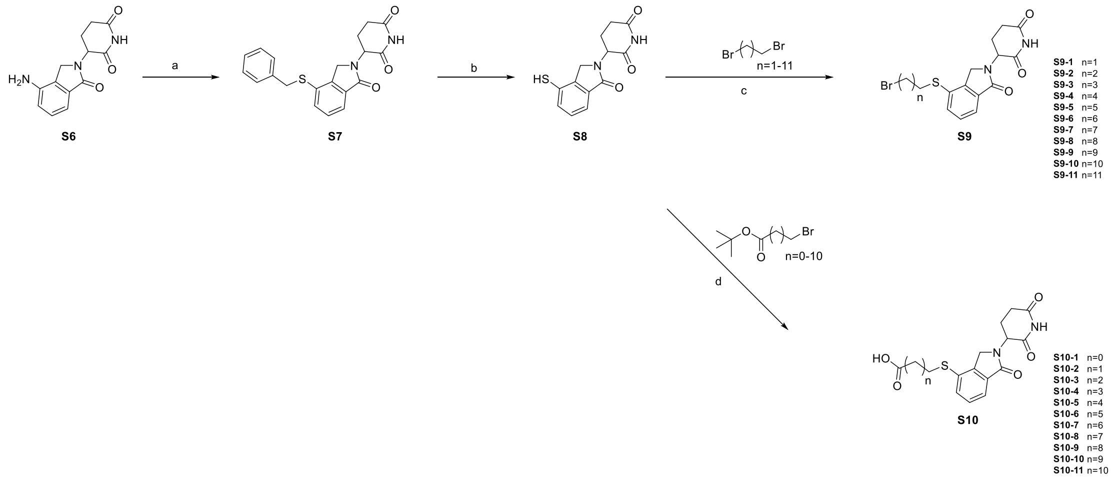

Reagents and conditions: (a) BnCl, $Na_2S_2O_3 \cdot 5H_2O$, $CuSO_4 \cdot 5H_2O$, Bipy, ^{t}BuONO, MeOH, H_2O, 80 ℃, 8 h; (b) $AlCl_3$, Toluene, 35 ℃, 12 h; (c) K_2CO_3, DMF, RT, 1 h; (d) i: tert-butyl 2-bromoacetate, K_2CO_3, DMF, 25 ℃, 2 h; ii: TFA, DCM, 25 ℃, 3 h.

PROTAC synthesis

Lastly, compounds **S5-1** and **S5-2** underwent alkylation reactions with Br-substituted linkers **S9** under condition a, or condensed with various acid-substituted linkers **S10** or under condition b to generate the corresponding SOS1 PROTACs **A1-A11**, **B1-B11**, **C1-C11**, **D1-D11** (Scheme 3) and Negative Control **(A11-NC)** (Scheme 4). All the synthesized compounds have been confirmed by NMR and mass spectrometry.

Scheme 3 Synthesis of SOS1 PROTACs based on BI-3406 analogues.

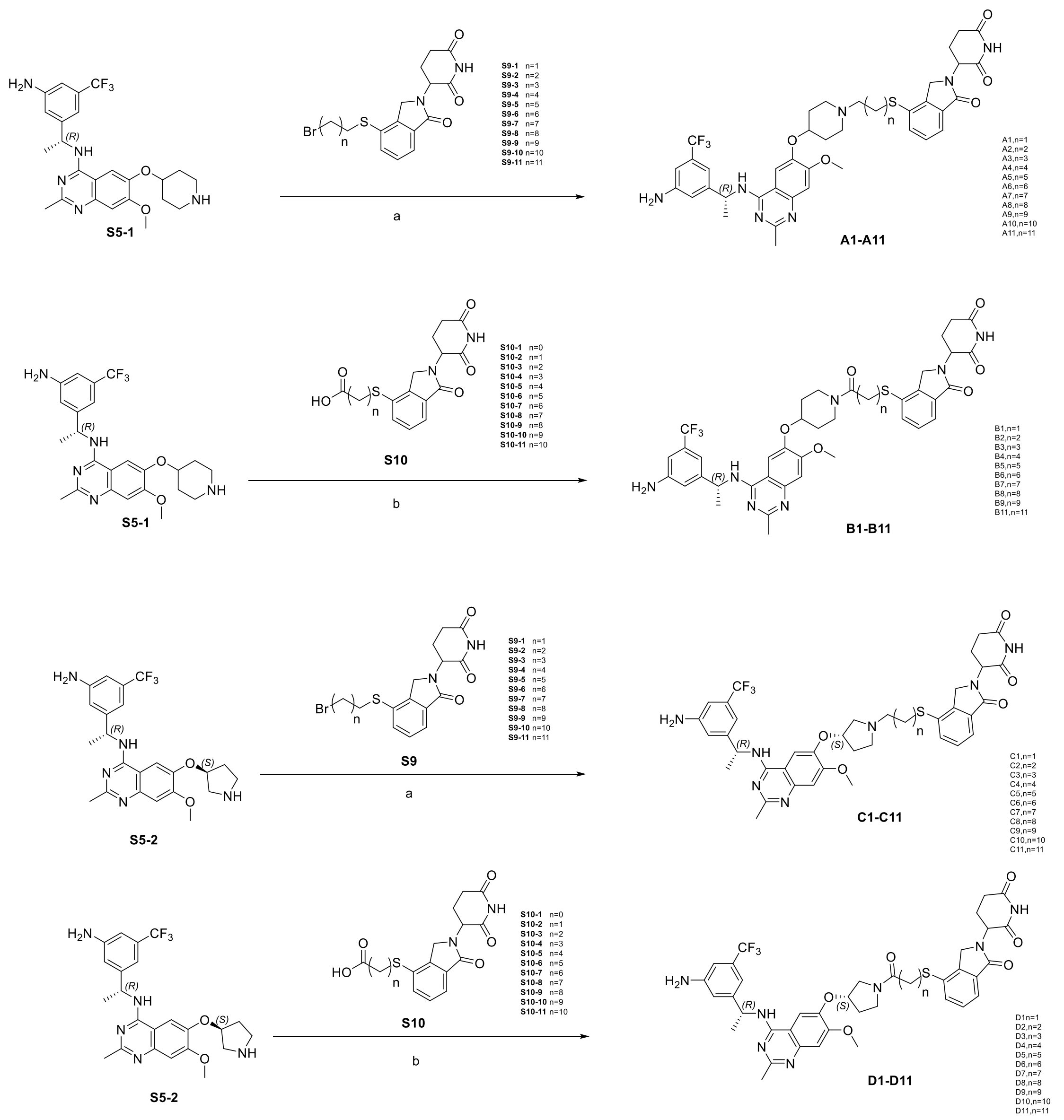

Reagents and conditions: (a) DIPEA, NaI, DMF, 80℃, 12h; (b) HATU, DIPEA, DMF, rt, 12h.

Scheme 4 Synthesis of Negative Control A11-NC.

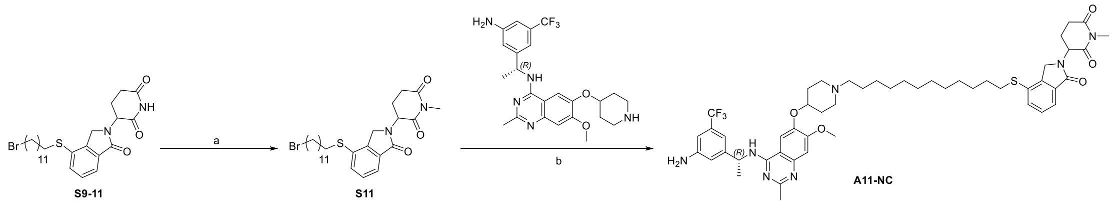

Reagents and conditions: (a) MeI, K_2CO_3, DMF, rt, 12h; (b) : DIPEA, NaI, DMF, 80℃, 12h.

2.2 Chemistry

All chemicals were obtained from commercial suppliers (Adamas and Alfa, bidepharm, ChemShuttle), and used without further purification. Flash chromatography was carried out on silica gel (200–300 mesh). All new compounds were characterized by ^{1}H NMR/^{13}C NMR, and HRMS. ^{1}H and ^{13}C NMR spectra were recorded on Bruker AVANCE III 500 MHZ (operating at 500 MHz for ^{1}H and 126 MHz for ^{13}C NMR), and chemical shifts were reported in ppm relative to the residual d_6-DMSO (2.50 ppm 1H), CD_3OD (3.31 ppm 1H) and coupling constants (J) are given in Hz. Multiplicities of signals are described as follows: s = singlet, d = doublet, t = triplet, q = quartet, m = multiple. High-Resolution Mass spectra were recorded on AB Triple 4600 spectrometer with CH_3CN and 0.01% TFA H_2O as solvent. All the final compounds were all purified by C18 reverse phase preparative HPLC column with solvent A (0.05% HCl in H_2O) and solvent B (CH_3CN) as eluents.

tert-butyl 4-((7-methoxy-2-methyl-4-oxo-3,4-dihydroquinazolin-6-yl)oxy)piperidine-1-carboxylate (S2-1)

The compound **S1-1** (745 mg, 2.66 mmol), 6-hydroxy-7-methoxy-2-methylquinazolin-4(3H)-one (500 mg, 2.42mmol), and 1.58 g Cs_2CO_3 were added to the 100 ml round bottle, followed by 5 mL anhydrous DMF to trigger the reaction. Then the reaction was stirred at 100 ℃ for 12 h. After that, the solvent was removed under the reduced pressure. Then the mixture was quenched with water (20 mL), extracted with DCM (3×30 mL), washed with water and brine, dried over anhydrous Na_2SO_4, and concentrated in vacuo and the residue was subjected to column purification (DCM/MeOH = 20:1) to obtain the intermediate compound **S2-1**. White solid, 580 mg (yield 71%). **^{1}H NMR** (500 MHz, CD_3OD) δ 7.70 (d, J = 28.8 Hz, 1H), 7.14 (d, J = 21.0 Hz, 1H), 4.74 (tt, J = 7.2, 3.5 Hz, 1H), 4.03 (d, J = 5.6 Hz, 3H), 3.73 (s, 1H), 3.48 – 3.35 (m, 2H), 3.27 (ddd, J = 12.8, 6.5, 4.1 Hz, 1H), 2.67 (d, J = 10.7 Hz, 3H), 2.22 (ddt, J = 13.1, 9.1, 3.6 Hz, 1H), 2.16 – 2.06 (m, 1H), 2.00 (ddt, J = 14.0, 7.7, 3.8 Hz, 1H), 1.78 – 1.71 (m, 1H), 1.47 (s, 9H). HRMS (ESI): calcd for $C_{20}H_{27}N_3O_5$ $[M+H]^+$, 390.2023 found, 390.2021.

tert-butyl 4-((7-methoxy-2-methyl-4-(((2,4,6-triisopropylphenyl)sulfonyl)oxy)quinazolin-6-yl)oxy)piperidine-1-carboxylate (S3-1)

The compound **S2-1** (500 mg, 1.28 mmol), 2,4,6-triisopropylbenzenesulfonyl chloride (466 mg, 1.54 mmol), DMAP (15.7 mg, 0.128mmol), and TEA (389 mg, 3.84mmol) were added to the 50 ml round bottle with 10 ml DCM. Then the reaction was stirred at room temperature for 12 h. After the reaction was completed, the mixture was condensed to remove the extra DCM and the residue was subjected to column purification (PE/EA = 1.5:1) to obtain the intermediate compound **S3-1**. White solid, 340 mg (yield 50%). **^{1}H NMR** (500 MHz, d_6-DMSO) δ 7.57 (s, 1H), 7.11 (s, 1H), 6.95 (s, 2H), 4.77 – 4.71 (m, 1H), 4.59 – 4.53 (m, 2H), 3.92 (s, 3H), 3.67 (d, *J* = 5.3 Hz, 2H), 3.20 (s, 2H), 2.83 – 2.76 (m, 1H), 2.52 (s, 3H), 1.96 – 1.90 (m, 2H), 1.58 – 1.51 (m, 2H), 1.41 (s, 9H), 1.16 (d, *J* = 6.9 Hz, 6H), 1.10 (d, *J* = 6.8 Hz, 12H). HRMS (ESI): calcd for $C_{35}H_{49}N_3O_7S$ $[M+H]^+$, 656.3364 found, 656.3368.

(R)-N-(1-(3-amino-5-(trifluoromethyl)phenyl)ethyl)-7-methoxy-2-methyl-6-(piperidin-4-yloxy)quinazolin-4-amine (S5-1)

The compound **S3-1** (500 mg, 0.76 mmol), (R)-3-(1-aminoethyl)-5-(trifluoromethyl)aniline (274 mg, 1.14 mmol), and TEA (0.4 mL) were added to a 50 mL round bottle with 5 mL DMSO. The solution was stirred at 90 ℃ for 12 h. When the reaction was completed, the mixture was poured into 30 mL H_2O and then extracted with 30 mL EA three times, washed with 30 mL water three times, saturated with 30 mL salt solution, dried over Na_2SO_4, and concentrated in vacuo and the residue was subjected to column purification (DCM/MeOH = 20:1) to obtain the intermediate compound **S4-1**. The intermediate **S4-1** was then added to a 25 mL single bottle with TFA solution (2mL TFA in 10 mL DCM) and stirred at room temperature overnight. Then the reaction mixture was condensed under the vacuum and purified by preparative HPLC (10% to 90% acetonitrile/0.05% HCl in H_2O) to obtain the compound **S5-1**. White solid, 200 mg (yield 75%). **^{1}H NMR** (500 MHz, CD_3OD) δ 8.31 (s, 1H), 7.57 – 7.54 (m, 2H), 7.25 (s, 1H), 7.11 (s, 1H), 5.85 (q, *J* = 7.0 Hz, 1H), 5.05 (dt, *J* = 6.9, 3.6 Hz, 1H), 4.04 (s, 3H), 3.49 – 3.43 (m, 2H), 3.38 – 3.32 (m, 2H), 2.63 (s, 3H),

2.29 (q, J = 6.7 Hz, 2H), 2.14 – 2.09 (m, 2H), 1.80 (d, J = 7.1 Hz, 3H). HRMS (ESI): calcd for $C_{24}H_{28}F_3N_5O_2$ $[M+H]^+$, 476.2268 found, 476.2265.

tert-butyl (S)-3-((7-methoxy-2-methyl-4-oxo-3,4-dihydroquinazolin-6-yl)oxy)pyrrolidine-1-carboxylate (S2-2)

The compound **S1-2** (643 mg, 2.42 mmol), 6-hydroxy-7-methoxy-2-methylquinazolin-4(3H)-one (500 mg, 2.42 mmol), and Cs_2CO_3 (1.58 g) were added to the 100 ml round bottle, followed by 5 mL anhydrous DMF to trigger the reaction. Then the reaction was stirred at 100 ℃ for 12 h. After that, the solvent was removed under the reduced pressure. Then the mixture was quenched with water (20 mL), extracted with DCM (3x30 mL), washed with water and brine, dried over anhydrous Na_2SO_4, and concentrated in vacuo and the residue was subjected to column purification (DCM/MeOH = 20:1) to obtain the intermediate compound **S2-2**. White solid, 646 mg (yield 71%). **^{1}H NMR** (500 MHz, CD_3OD) δ 7.62 (d, J = 3.5 Hz, 1H), 7.10 (d, J = 2.4 Hz, 1H), 5.21 – 5.13 (m, 1H), 4.02 (s, 3H), 3.71 – 3.59 (m, 2H), 3.58 – 3.45 (m, 2H), 2.68 (s, 3H), 2.28 – 2.19 (m, 2H), 1.47 (d, J = 9.1 Hz, 9H). HRMS (ESI): calcd for $C_{19}H_{25}N_3O_5$ $[M+H]^+$, 376.1867; found, 376.1866.

tert-butyl (S)-3-((7-methoxy-2-methyl-4-(((2,4,6-triisopropylphenyl)sulfonyl)oxy)quinazolin-6-yl)oxy)pyrrolidine-1-carboxylate (S3-2)

The compound **S2-2** (500 mg, 1.33 mmol), 2,4,6-triisopropylbenzenesulfonyl chloride (484 mg, 1.60 mmol), DMAP (16.3 mg, 0.133mmol), and TEA (404 mg, 3.99mmol) were added to the 50 ml round bottle with 10 ml DCM. Then the reaction was stirred at room temperature for 12 h. After the reaction was completed, the mixture was condensed to remove the extra DCM and the residue was subjected to column purification (PE/EA = 1.5:1) to obtain the intermediate compound **S3-2**. White solid, 602 mg (yield 70%). **^{1}H NMR** (500 MHz, d_6-DMSO) δ 7.48 (s, 1H), 7.11 (s, 1H), 6.95 (s, 2H), 5.17 (d, J = 7.6 Hz, 1H), 4.56 (p, J = 6.9 Hz, 2H), 3.92 (s, 3H), 3.43 (d, J = 7.8 Hz, 2H), 3.34 – 3.29 (m, 2H), 2.79 (p, J = 6.9 Hz, 1H), 2.51 (s, 3H), 2.22 – 2.13 (m, 1H), 2.09 (d, J = 19.4 Hz, 1H), 1.40 (d, J = 8.8 Hz, 9H), 1.16 (d, J = 7.0 Hz, 6H), 1.10 (d, J = 6.8 Hz,

12H). HRMS (ESI): calcd for $C_{34}H_{47}N_3O_7S$ $[M+H]^+$, 642.3207 found, 642.3199.

N-((R)-1-(3-amino-5-(trifluoromethyl)phenyl)ethyl)-7-methoxy-2-methyl-6-(((S)-pyrrolidin-3-yl)oxy)quinazolin-4-amine (S5-2)

The compound **S3-2** (500 mg, 0.78 mmol), (R)-3-(1-aminoethyl)-5-(trifluoromethyl)aniline (281 mg, 1.17 mmol), and TEA (0.4 mL) were added to a 50 mL round bottle with 5 mL DMSO. The solution was stirred at 90 ℃ for 12 h. When the reaction was completed, the mixture was poured into 30 mL H_2O and then extracted with 30 mL EA three times, washed with 30 mL water three times, saturated with 30 mL salt solution, dried over Na_2SO_4, and concentrated in vacuo and the residue was subjected to column purification (DCM/MeOH = 20:1) to obtain the intermediate compound **S4-2**. The intermediate**S4-2** was then added to a 25 mL single bottle with TFA solution (2mL TFA in 10 mL DCM) and stirred at room temperature overnight. Then the reaction mixture was condensed under the vacuum and purified by preparative HPLC (10% to 90% acetonitrile/0.05% HCl in H_2O) to obtain the compound **S5-2**. White solid, 287 mg (yield 80%). **^{1}H NMR** (500 MHz, CD_3OD) δ 8.32 (s, 1H), 7.66 (s, 2H), 7.33 (s, 1H), 7.13 (s, 1H), 5.87 (q, *J* = 7.0 Hz, 1H), 5.52 (s, 1H), 4.03 (s, 3H), 3.73 – 3.65 (m, 2H), 3.58 – 3.53 (m, 2H), 2.64 (s, 3H), 2.47 – 2.39 (m, 2H), 1.82 (d, *J* = 7.1 Hz, 3H). HRMS(ESI): calcd for $C_{23}H_{26}F_3N_5O_2$ $[M+H]^+$, 462.2111 found, 462.2113.

3-(4-((2-(4-((4-(((R)-1-(3-amino-5-(trifluoromethyl)phenyl)ethyl)amino)-7-methoxy-2-methylquinazolin-6-yl)oxy)piperidin-1-yl)ethyl)thio)-1-oxoisoindolin-2-yl)piperidine-2,6-dione (A1)

The compound **S5-1** (20 mg, 0.042 mmol) and compound **S9-1** (19.9 mg, 0.031 mmol) were added into a 25 mL round single bottle. Then NaI (13 mg, 0.052 mmol), K_2CO_3 (12 mg, 0.052

mmol) and DMF (2 mL) were added and the mixture was stirred at 80 ℃ for 12 h. After the reaction was finished, the mixture was filtered, followed by purification via preparative HPLC (10% to 90% acetonitrile/0.05% HCl in H2O) to obtain the compound **A1**. White solid, 8.4 mg (yield 25.7%). **^{1}H NMR** (500 MHz, CD_3OD) δ 8.31 (d, *J* = 43.9 Hz, 1H), 7.84 (dd, *J* = 7.7, 3.7 Hz, 1H), 7.76 (d, *J* = 7.5 Hz, 1H), 7.62 (t, *J* = 7.7 Hz, 1H), 7.54 (s, 1H), 7.40 (d, *J* = 8.6 Hz, 1H), 7.18 (d, *J* = 30.5 Hz, 1H), 7.10 (d, *J* = 15.5 Hz, 1H), 5.89 – 5.79 (m, 1H), 5.19 (dd, *J* = 13.3, 5.1 Hz, 1H), 5.01 (d, *J* = 3.7 Hz, 1H), 4.60 – 4.47 (m, 2H), 4.02 (d, *J* = 8.6 Hz, 3H), 3.75 (d, *J* = 12.7 Hz, 1H), 3.60 – 3.56 (m, 1H), 3.53 – 3.47 (m, 2H), 3.45 – 3.37 (m, 4H), 2.97 – 2.88 (m, 1H), 2.83 – 2.76 (m, 1H), 2.63 (d, *J* = 2.8 Hz, 3H), 2.60 – 2.44 (m, 2H), 2.35 – 2.26 (m, 2H), 2.23 – 2.18 (m, 1H), 2.06 – 1.98 (m, 1H), 1.81 (d, *J* = 7.1 Hz, 1H), 1.77 (d, *J* = 7.1 Hz, 2H). **^{13}C NMR** (126 MHz, CD_3OD) δ 174.63, 172.24, 170.85, 161.20, 160.32, 159.62, 159.06, 148.76, 148.47, 147.99, 144.71, 137.50, 134.57, 133.79, 130.87, 130.64, 124.47, 123.53, 123.49, 110.15, 107.96, 106.52, 100.61, 100.37, 72.86, 70.54, 57.39, 57.24, 57.14, 53.78, 52.54, 32.37, 28.25, 28.11, 27.84, 24.10, 21.60, 21.46. HRMS (ESI): calcd for $C_{39}H_{42}F_3N_7O_5S$ $[M+H]^+$, 778.2993 found, 778.2997.

3-(4-((3-(4-((4-(((R)-1-(3-amino-5-(trifluoromethyl)phenyl)ethyl)amino)-7-methoxy-2-methylquinazolin-6-yl)oxy)piperidin-1-yl)propyl)thio)-1-oxoisoindolin-2-yl)piperidine-2,6-dione (A2)

The compound **A2** was obtained from the nucleophilic substitution of **S5-1** and **S9-2** according to the similar process of **A1**. White solid, 4.2 mg (yield 12.6%). **^{1}H NMR** (500 MHz, CD_3OD) δ 8.27 (d, *J* = 46.9 Hz, 1H), 7.78 – 7.74 (m, 1H), 7.73 – 7.69 (m, 1H), 7.61 – 7.56 (m, 1H), 7.38 – 7.32 (m, 1H), 7.23 (d, *J* = 14.4 Hz, 1H), 7.08 (d, *J* = 18.1 Hz, 1H), 7.01 (d, 1H), 5.86 – 5.76 (m, 1H), 5.22 – 5.15 (m, 1H), 5.01 – 4.97 (m, 1H), 4.56 – 4.42 (m, 2H), 4.02 (d, *J* = 13.4 Hz, 3H), 3.71 – 3.64 (m, 1H), 3.54 – 3.49 (m, 1H), 3.38 – 3.32 (m, 4H), 3.27 – 3.13 (m, 2H), 2.96 – 2.87 (m, 1H), 2.83 – 2.75 (m, 1H), 2.63 (d, *J* = 4.6 Hz, 3H), 2.60 – 2.43 (m, 2H), 2.34 – 2.29 (m, 1H), 2.24 – 2.09 (m, 4H), 2.04 – 1.93 (m, 1H), 1.77 (dd, *J* = 22.4, 7.1 Hz, 3H). **^{13}C NMR** (126 MHz, CD_3OD) δ 174.63, 172.25, 171.01, 161.17, 160.28, 159.58, 158.99, 148.76, 148.42, 147.79, 144.00, 133.72, 133.43, 132.32, 130.62, 123.10, 122.69, 122.62, 110.11, 107.88, 106.51, 100.62,

100.35, 72.93, 70.55, 57.29, 56.97, 56.21, 53.76, 52.65, 52.56, 32.37, 28.23, 28.10, 25.31, 25.10, 24.09, 21.61, 21.47. HRMS (ESI): calcd for $C_{40}H_{44}F_3N_7O_5S$ $[M+H]^+$, 792.3149 found, 792.3148.

3-(4-((4-(4-((4-(((R)-1-(3-amino-5-(trifluoromethyl)phenyl)ethyl)amino)-7-methoxy-2-methylquinazolin-6-yl)oxy)piperidin-1-yl)butyl)thio)-1-oxoisoindolin-2-yl)piperidine-2,6-dione (A3)

The compound **A3** was obtained from the nucleophilic substitution of **S5-1** and **S9-3** according to the similar process of **A1**. White solid, 6.1 mg (yield 18.0%). **^{1}H NMR** (500 MHz, CD_3OD) δ 8.29 (d, *J* = 46.4 Hz, 1H), 7.74 – 7.65 (m, 2H), 7.55 (t, *J* = 7.6 Hz, 1H), 7.40 (d, *J* = 11.1 Hz, 1H), 7.29 (d, *J* = 13.4 Hz, 1H), 7.13 – 7.04 (m, 2H), 5.86 – 5.78 (m, 1H), 5.22 – 5.14 (m, 1H), 5.03 – 4.99 (m, 1H), 4.47 (q, *J* = 17.3 Hz, 2H), 4.03 (d, *J* = 14.8 Hz, 3H), 3.72 – 3.64 (m, 1H), 3.53 – 3.47 (m, 1H), 3.37 – 3.33 (m, 1H), 3.22 – 3.11 (m, 4H), 2.96 – 2.86 (m, 1H), 2.83 – 2.75 (m, 1H), 2.63 (d, *J* = 3.8 Hz, 3H), 2.60 – 2.42 (m, 2H), 2.34 – 2.29 (m, 1H), 2.26 – 2.16 (m, 2H), 2.05 – 1.89 (m, 3H), 1.80 (d, *J* = 7.1 Hz, 1H), 1.76 (d, *J* = 7.0 Hz, 3H), 1.75 – 1.68 (m, 1H). **^{13}C NMR** (126 MHz, CD_3OD) δ 174.61, 172.25, 171.08, 161.17, 160.30, 159.61, 159.01, 148.44, 148.29, 148.02, 143.72, 133.42, 133.36, 133.08, 130.48, 123.93, 122.22, 122.18, 110.18, 107.88, 106.52, 100.63, 100.37, 73.01, 70.65, 57.72, 57.46, 57.26, 56.94, 53.76, 52.64, 52.56, 33.14, 32.39, 29.46, 28.08, 27.30, 24.32, 24.08, 22.33, 21.50. HRMS (ESI): calcd for $C_{41}H_{46}F_3N_7O_5S$ $[M+H]^+$, 806.3306 found, 806.3308.

3-(4-((5-(4-((4-(((R)-1-(3-amino-5-(trifluoromethyl)phenyl)ethyl)amino)-7-methoxy-2-methylquinazolin-6-yl)oxy)piperidin-1-yl)pentyl)thio)-1-oxoisoindolin-2-yl)piperidine-2,6-dione (A4)

The compound **A4** was obtained from the nucleophilic substitution of **S5-1** and **S9-4** according to the similar process of **A1**. White solid, 5.5 mg (yield 17.0%). **^{1}H NMR** (500 MHz, CD_3OD) δ 8.30 (d, *J* = 44.4 Hz, 1H), 7.69 – 7.64 (m, 2H), 7.58 – 7.49 (m, 2H), 7.38 (d, *J* = 9.8 Hz, 1H), 7.19

– 7.07 (m, 2H), 5.89 – 5.79 (m, 1H), 5.21 – 5.15 (m, 1H), 5.03 – 4.99 (m, 1H), 4.50 – 4.40 (m, 2H), 4.03 (d, *J* = 14.6 Hz, 3H), 3.73 – 3.64 (m, 2H), 3.53 – 3.49 (m, 1H), 3.37 – 3.34 (m, 1H), 3.18 – 3.10 (m, 4H), 2.95 – 2.87 (m, 1H), 2.79 (ddt, *J* = 17.7, 4.9, 2.5 Hz, 1H), 2.63 (d, *J* = 3.1 Hz, 3H), 2.59 – 2.43 (m, 2H), 2.34 – 2.29 (m, 1H), 2.25 – 2.18 (m, 2H), 2.05 – 1.93 (m, 1H), 1.84 – 1.74 (m, 7H), 1.60 – 1.54 (m, 2H). **^{13}C NMR** (126 MHz, CD_3OD) δ 174.62, 172.27, 171.14, 161.17, 160.28, 159.59, 159.00, 148.78, 148.46, 147.61, 143.42, 137.45, 133.22, 133.16, 133.05, 132.95, 130.43, 122.02, 121.95, 110.10, 107.91, 106.50, 100.63, 100.37, 73.03, 70.66, 57.47, 57.30, 57.23, 53.74, 52.57, 36.98, 33.36, 32.38, 31.66, 29.73, 28.10, 26.53, 24.66, 24.07, 22.32, 21.45. HRMS (ESI): calcd for $C_{42}H_{48}F_3N_7O_5S$ $[M+H]^+$, 820.3462 found, 820.3458.

3-(4-((6-(4-((4-(((R)-1-(3-amino-5-(trifluoromethyl)phenyl)ethyl)amino)-7-methoxy-2-methylquinazolin-6-yl)oxy)piperidin-1-yl)hexyl)thio)-1-oxoisoindolin-2-yl)piperidine-2,6-dione (A5)

The compound **A5** was obtained from the nucleophilic substitution of **S5-1** and **S9-5** according to the similar process of **A1**. White solid, 4.5 mg (yield 25.8%). **^{1}H NMR** (500 MHz, CD_3OD) δ 8.25 (d, *J* = 29.8 Hz, 1H), 7.72 – 7.59 (m, 2H), 7.57 – 7.51 (m, 1H), 7.30 – 7.18 (m, 2H), 7.10 (d, *J* = 13.1 Hz, 1H), 6.97 (d, *J* = 21.6 Hz, 1H), 5.87 – 5.77 (m, 1H), 5.25 – 5.12 (m, 1H), 5.00 – 4.96 (m, 1H), 4.54 – 4.38 (m, 2H), 4.03 (d, *J* = 13.8 Hz, 3H), 3.63 – 3.47 (m, 2H), 3.35 – 3.33 (m, 1H), 3.30 – 3.16 (m, 1H), 3.16 – 3.06 (m, 4H), 2.96 – 2.84 (m, 1H), 2.83 – 2.76 (m, 1H), 2.64 (d, *J* = 1.4 Hz, 3H), 2.59 – 2.50 (m, 1H), 2.48 – 2.41 (m, 1H), 2.35 – 2.29 (m, 1H), 2.23 – 2.16 (m, 2H), 1.95 (dd, *J* = 21.6, 11.3 Hz, 1H), 1.78 – 1.77 (m, 1H), 1.75 (d, *J* = 7.3 Hz, 3H), 1.73 – 1.63 (m, 3H), 1.58 – 1.49 (m, 2H), 1.45 – 1.38 (m, 2H). **^{13}C NMR** (126 MHz, CD_3OD) δ 174.62, 172.25, 171.15, 161.17, 160.27, 159.58, 158.98, 148.75, 148.45, 147.47, 143.32, 137.45, 137.07, 133.85, 133.10, 132.93, 130.43, 130.38, 121.84, 110.10, 106.50, 106.46, 100.63, 100.38, 72.98, 70.67, 58.24, 57.46, 57.38, 57.23, 53.74, 52.57, 33.47, 32.38, 30.00, 28.96, 27.08, 24.90, 24.05, 22.32, 22.28, 21.53, 21.44. HRMS (ESI): calcd for $C_{43}H_{50}F_3N_7O_5S$ $[M+H]^+$, 834.3619 found, 834.3620.

3-(4-((7-(4-((4-(((R)-1-(3-amino-5-(trifluoromethyl)phenyl)ethyl)amino)-7-methoxy-2-methylquinazolin-6-yl)oxy)piperidin-1-yl)heptyl)thio)-1-oxoisoindolin-2-yl)piperidine-2,6-dione (A6)

The compound **A6** was obtained from the nucleophilic substitution of **S5-1** and **S9-6** according to the similar process of **A1**. White solid, 6.2 mg (yield 17.4%). **^{1}H NMR** (500 MHz, CD_3OD) δ 8.28 (d, *J* = 40 Hz, 1H), 7.69 – 7.61 (m, 2H), 7.56 – 7.50 (m, 1H), 7.40 (d, *J* = 11.8 Hz, 1H), 7.31 (d, *J* = 12.1 Hz, 1H), 7.13 – 7.03 (m, 2H), 5.88 – 5.78 (m, 1H), 5.23 – 5.13 (m, 1H), 5.04 – 4.96 (m, 1H), 4.51 – 4.38 (m, 2H), 4.03 (d, *J* = 14.5 Hz, 3H), 3.70 – 3.58 (m, 1H), 3.55 – 3.47 (m, 1H), 3.29 – 3.20 (m, 1H), 3.17 – 3.13 (m, 2H), 3.12 – 3.03 (m, 2H), 2.96 – 2.85 (m, 1H), 2.78 (ddd, *J* = 17.7, 4.7, 2.4 Hz, 1H), 2.64 (d, *J* = 1.8 Hz, 3H), 2.59 – 2.45 (m, 2H), 2.32 (d, *J* = 15.2 Hz, 1H), 2.23 – 2.15 (m, 2H), 2.05 – 1.93 (m, 1H), 1.79 (d, *J* = 7.0 Hz, 1H), 1.76 (d, *J* = 7.0 Hz, 3H), 1.71 – 1.67 (m, 2H), 1.53 – 1.47 (m, 2H), 1.42 – 1.36 (m, 4H), 1.35 – 1.27 (m, 4H). **^{13}C NMR** (126 MHz, CD_3OD) δ 174.64, 172.23, 171.17, 161.17, 160.84, 159.59, 159.01, 148.78, 148.46, 147.67, 143.20, 137.45, 137.08, 133.98, 133.08, 132.74, 130.37, 130.35, 121.75, 110.05, 106.51, 106.46, 100.63, 100.38, 73.03, 70.65, 58.35, 57.64, 57.25, 57.13, 53.74, 52.56, 33.54, 32.38, 30.12, 29.58, 29.28, 28.23, 28.11, 27.43, 24.99, 24.06, 22.33, 21.46. HRMS (ESI): calcd for $C_{44}H_{52}F_3N_7O_5S$ $[M+H]^+$, 848.3775 found, 848.3773.

3-(4-((8-(4-((4-(((R)-1-(3-amino-5-(trifluoromethyl)phenyl)ethyl)amino)-7-methoxy-2-methylquinazolin-6-yl)oxy)piperidin-1-yl)octyl)thio)-1-oxoisoindolin-2-yl)piperidine-2,6-dione (A7)

The compound **A7** was obtained from the nucleophilic substitution of **S5-1** and **S9-7** according to the similar process of **A1**. White solid, 11.4 mg (yield 31.5%) **^{1}H NMR** (500 MHz, CD_3OD) δ 8.33 (d, J = 39.0 Hz, 1H), 7.66 – 7.60 (m, 2H), 7.56 – 7.48 (m, 2H), 7.41 (d, J = 15.0 Hz, 1H), 7.21 – 7.08 (m, 2H), 5.89 – 5.78 (m, 1H), 5.22 – 5.12 (m, 1H), 5.05 – 4.99 (m, 1H), 4.50 – 4.36 (m, 2H), 4.03 (d, *J* = 15.2 Hz, 3H), 3.71 – 3.62 (m, 1H), 3.55 – 3.49 (m, 1H), 3.38 – 3.33 (m, 1H), 3.18 – 3.03 (m, 4H), 2.96 – 2.85 (m, 1H), 2.83 – 2.75 (m, 1H), 2.64 (d, *J* = 2.7 Hz, 3H), 2.58 –

2.45 (m, 2H), 2.36 – 2.29 (m, 1H), 2.27 – 2.16 (m, 2H), 2.07 – 1.95 (m, 1H), 1.81 (d, *J* = 7.0 Hz, 1H), 1.78 (d, *J* = 7.0 Hz, 3H), 1.70 – 1.64 (m, 2H), 1.52 – 1.45 (m, 2H), 1.38 – 1.29 (m, 8H). **^{13}C NMR** (126 MHz, CD_3OD) δ 174.63, 172.22, 171.17, 161.14, 160.26, 159.58, 158.97, 148.77, 148.44, 147.71, 143.13, 137.43, 137.05, 134.03, 133.05, 132.69, 132.66, 130.35, 121.69, 110.12, 106.51, 106.45, 100.65, 100.39, 73.04, 70.67, 58.39, 57.63, 57.47, 57.28, 53.76, 52.57, 33.56, 32.40, 30.15, 29.83, 29.37, 28.24, 28.12, 27.49, 25.30, 25.07, 24.06, 22.36, 21.50. HRMS (ESI): calcd for $C_{45}H_{54}F_3N_7O_5S$ $[M+H]^+$, 862.3932 found, 862.3935.

3-(4-((9-(4-((4-(((R)-1-(3-amino-5-(trifluoromethyl)phenyl)ethyl)amino)-7-methoxy-2-methylquinazolin-6-yl)oxy)piperidin-1-yl)nonyl)thio)-1-oxoisoindolin-2-yl)piperidine-2,6-dione (A8)

The compound **A8** was obtained from the nucleophilic substitution of **S5-1** and **S9-8** according to the similar process of **A1**. White solid, 10.3 mg (yield 27.9%) **^{1}H NMR** (500 MHz, CD_3OD) δ 8.30 (d, J = 44.5 Hz, 1H), 7.66 – 7.60 (m, 2H), 7.55 – 7.50 (m, 1H), 7.39 – 7.29 (m, 1H), 7.29 – 7.22 (m, 1H), 7.14 – 7.00 (m, 2H), 5.86 – 5.77 (m, 1H), 5.20 – 5.12 (m, 1H), 5.04 – 4.96 (m, 1H), 4.50 – 4.35 (m, 2H), 4.03 (d, *J* = 14.9 Hz, 3H), 3.72 – 3.65 (m, 1H), 3.56 – 3.50 (m, 1H), 3.38 – 3.33 (m, 1H), 3.19 – 3.13 (m, 2H), 3.10 – 3.01 (m, 2H), 2.95 – 2.85 (m, 1H), 2.82 – 2.74 (m, 1H), 2.64 (d, J = 3.3 Hz, 3H), 2.59 – 2.44 (m, 2H), 2.36 – 2.30 (m, 1H), 2.28 – 2.21 (m, 1H), 2.21 – 2.15 (m, 1H), 2.06 – 1.95 (m, 1H), 1.79 (d, *J* = 7.1 Hz, 2H), 1.76 (d, *J* = 7.1 Hz, 3H), 1.67 (p, *J* = 7.3 Hz, 2H), 1.51 – 1.43 (m, 2H), 1.40 – 1.26 (m, 9H). **^{13}C NMR** (126 MHz, CD_3OD) δ 174.64, 172.21, 171.19, 161.15, 160.22, 159.51, 158.99, 148.71, 148.45, 146.73, 143.11, 137.41, 137.09, 134.07, 133.06, 132.61, 130.33, 121.68, 119.90, 119.40, 109.90, 106.47, 100.64, 100.40, 73.13, 70.66, 58.44, 57.64, 57.46, 57.38, 53.76, 52.60, 33.55, 32.38, 30.20, 30.01, 29.94, 29.46, 28.22, 28.14, 27.61, 25.35, 25.10, 24.04, 22.33, 21.41. HRMS (ESI): calcd for $C_{46}H_{56}F_3N_7O_5S$ $[M+H]^+$, 876.4088 found, 876.4091.

3-(4-((10-(4-((4-(((R)-1-(3-amino-5-(trifluoromethyl)phenyl)ethyl)amino)-7-methoxy-2-

methylquinazolin-6-yl)oxy)piperidin-1-yl)decyl)thio)-1-oxoisoindolin-2-yl)piperidine-2,6-dione (A9)

The compound **A9** was obtained from the nucleophilic substitution of **S5-1** and **S9-9** according to the similar process of **A1**. White solid, 11.6 mg (yield 31.0%) **^{1}H NMR** (500 MHz, CD_3OD) δ 8.35 (d, *J* = 34.3 Hz, 1H), 7.61 (d, *J* = 7.4 Hz, 2H), 7.57 – 7.48 (m, 2H), 7.44 (d, *J* = 14.7 Hz, 1H), 7.22 – 7.09 (m, 2H), 5.83 (dd, *J* = 12.7, 6.7 Hz, 1H), 5.16 (dd, *J* = 11.8, 5.7 Hz, 1H), 5.05 (s, 1H), 4.50 – 4.36 (m, 2H), 4.03 (d, *J* = 17.4 Hz, 3H), 3.74 – 3.64 (m, 1H), 3.58 – 3.49 (m, 1H), 3.35 (s, 1H), 3.20 – 3.13 (m, 2H), 3.09 – 3.01 (m, 2H), 2.97 – 2.85 (m, 1H), 2.83 – 2.74 (m, 1H), 2.64 (d, *J* = 3.2 Hz, 3H), 2.58 – 2.44 (m, 2H), 2.36 – 2.24 (m, 2H), 2.22 – 2.16 (m, 1H), 2.09 – 1.97 (m, 1H), 1.82 (d, *J* = 7.0 Hz, 1H), 1.78 (d, *J* = 7.1 Hz, 3H), 1.66 (p, *J* = 7.1 Hz, 2H), 1.52 – 1.43 (m, 2H), 1.41 – 1.21 (m, 12H). **^{13}C NMR** (126 MHz, CD_3OD) δ 174.68, 172.20, 171.19, 160.76, 160.24, 159.55, 158.93, 148.76, 148.40, 147.94, 143.02, 137.41, 137.02, 134.09, 133.15, 133.02, 132.50, 130.33, 121.62, 110.18, 106.49, 106.44, 100.67, 100.41, 73.07, 70.67, 58.41, 57.63, 57.46, 57.29, 53.77, 52.58, 33.54, 32.39, 30.29, 30.18, 30.12, 30.04, 29.51, 28.10, 27.62, 25.10, 24.05, 22.35, 22.30, 21.66, 21.53. HRMS (ESI): calcd for $C_{47}H_{58}F_3N_7O_5S$ $[M+H]^+$, 890.4245 found, 890.4238.

3-(4-((11-(4-((4-(((R)-1-(3-amino-5-(trifluoromethyl)phenyl)ethyl)amino)-7-methoxy-2-methylquinazolin-6-yl)oxy)piperidin-1-yl)undecyl)thio)-1-oxoisoindolin-2-yl)piperidine-2,6-dione (A10)

The compound **A10** was obtained from the nucleophilic substitution of **S5-1** and **S9-10** according to the similar process of **A1** White solid, 12.5 mg (yield 33.3%). **^{1}H NMR** (500 MHz, CD_3OD) δ 8.22 (d, *J* = 43.1 Hz, 1H), 7.63 (d, *J* = 7.2 Hz, 2H), 7.52 (t, *J* = 7.6 Hz, 1H), 7.23 – 7.06 (m, 3H), 6.94 (d, *J* = 12.5 Hz, 1H), 5.83 – 5.76 (m, 1H), 5.19 – 5.13 (m, 1H), 4.99 – 4.96 (m, 1H), 4.48 – 4.37 (m, 2H), 4.04 (d, *J* = 13.6 Hz, 3H), 3.74 – 3.67 (m, 1H), 3.56 – 3.52 (m, 1H), 3.40 – 3.35 (m, 1H), 3.28 – 3.22 (m, 1H), 3.19 – 3.15 (m, 2H), 3.09 – 3.03 (m, 2H), 2.95 – 2.85 (m, 1H), 2.81 – 2.75 (m, 1H), 2.64 (d, *J* = 3.2 Hz, 3H), 2.58 – 2.44 (m, 2H), 2.36 – 2.31 (m, 1H), 2.24 – 2.16 (m, 2H), 2.05 – 1.95 (m, 1H), 1.77 (d, *J* = 7.1 Hz, 2H), 1.74 (d, *J* = 7.1 Hz, 3H), 1.69 – 1.64 (m, 2H),

1.48 – 1.43 (m, 2H), 1.39 – 1.30 (m, 12H). **^{13}C NMR** (126 MHz, CD_3OD) δ 174.66, 172.19, 171.21, 161.16, 160.26, 159.55, 159.01, 148.47, 147.63, 147.31, 143.06, 137.42, 137.09, 134.10, 133.06, 132.52, 130.32, 121.66, 109.95, 108.05, 106.50, 106.46, 100.63, 100.39, 73.10, 70.65, 58.44, 57.47, 57.38, 57.30, 53.75, 52.58, 33.54, 32.38, 30.41, 30.22, 30.08, 29.53, 28.24, 28.13, 27.65, 25.40, 25.14, 24.05, 22.33, 22.28, 21.60, 21.44. HRMS (ESI): calcd for $C_{48}H_{60}F_3N_7O_5S$ $[M+H]^+$, 904.4401 found, 904.4402.

3-(4-((12-(4-((4-(((R)-1-(3-amino-5-(trifluoromethyl)phenyl)ethyl)amino)-7-methoxy-2-methylquinazolin-6-yl)oxy)piperidin-1-yl)dodecyl)thio)-1-oxoisoindolin-2-yl)piperidine-2,6-dione (A11, SIAIS562055)

The compound **A11** was obtained from the nucleophilic substitution of **S5-1** and **S9-11** according to the similar process of **A1** White solid, 14.6 mg (yield 37.9%) **^{1}H NMR** (500 MHz, CD_3OD) δ 8.32 (d, *J* = 40.3 Hz, 1H), 7.60 (dd, *J* = 7.7, 3.4 Hz, 2H), 7.50 (t, *J* = 7.7 Hz, 1H), 7.42 (d, *J* = 15.6 Hz, 1H), 7.32 (d, *J* = 18.4 Hz, 1H), 7.16 – 7.05 (m, 2H), 5.86 – 5.76 (m, 1H), 5.21 – 5.12 (m, 1H), 5.07 – 4.99 (m, 1H), 4.46 – 4.36 (m, 2H), 4.03 (d, *J* = 17.2 Hz, 3H), 3.73 – 3.66 (m, 1H), 3.57 – 3.49 (m, 1H), 3.37 – 3.34 (m, 1H), 3.21 – 3.14 (m, 2H), 3.05 (t, *J* = 7.2 Hz, 2H), 2.96 – 2.88 (m, 1H), 2.89 – 2.85 (m, 1H), 2.82 – 2.75 (m, 1H), 2.64 (d, *J* = 3.7 Hz, 3H), 2.58 – 2.45 (m, 2H), 2.35 – 2.24 (m, 2H), 2.21 – 2.15 (m, 1H), 2.09 – 1.96 (m, 1H), 1.81 (d, *J* = 7.1 Hz, 2H), 1.77 (d, *J* = 7.1 Hz, 3H), 1.69 – 1.63 (m, 2H), 1.49 – 1.43 (m, 2H), 1.41 – 1.36 (m, 4H), 1.33 – 1.28 (m, 10H). **^{13}C NMR** (126 MHz, CD_3OD) δ 174.64, 172.19, 171.15, 161.10, 160.76, 160.19, 159.48, 148.70, 148.39, 147.14, 142.90, 137.37, 137.01, 134.11, 133.00, 132.30, 130.30, 121.57, 109.95, 107.91, 106.45, 106.41, 100.63, 100.38, 73.07, 70.63, 58.42, 57.40, 57.29, 57.25, 53.73, 52.71, 52.59, 33.48, 32.37, 30.53, 30.50, 30.46, 30.20, 30.12, 29.59, 28.19, 28.10, 27.65, 25.38, 25.12, 24.04, 22.34, 21.48. HRMS (ESI): calcd for $C_{49}H_{62}F_3N_7O_5S$ $[M+H]^+$, 918.4558 found, 918.4551.

3-(4-((12-(4-((4-(((R)-1-(3-amino-5-(trifluoromethyl)phenyl)ethyl)amino)-7-methoxy-2-methylquinazolin-6-yl)oxy)piperidin-1-yl)dodecyl)thio)-1-oxoisoindolin-2-yl)-1-

methylpiperidine-2,6-dione（A11-NC）

The compound **A11-NC** was obtained from the nucleophilic substitution of **S5-1** and **S11** according to the similar process of **A1**. White solid, 4.7 mg (yield 12.1%). **^{1}H NMR** (500 MHz, CD_3OD) δ 8.39 (dd, *J* = 30.7, 5.3 Hz, 1H), 7.84 (d, *J* = 11.0 Hz, 1H), 7.75 (s, 1H), 7.63 (dd, *J* = 8.1, 1.6 Hz, 2H), 7.53 (d, *J* = 7.7 Hz, 1H), 7.44 (d, *J* = 18.6 Hz, 1H), 7.12 (d, *J* = 17.5 Hz, 1H), 5.95 – 5.84 (m, 1H), 5.21 – 5.17 (m, 1H), 5.10 – 5.06 (m, 1H), 4.45 – 4.38 (m, 2H), 4.03 (d, *J* = 16.3 Hz, 3H), 3.74 – 3.68 (m, 1H), 3.56 – 3.52 (m, 1H), 3.38 – 3.35 (m, 1H), 3.21 – 3.17 (m, 2H), 3.14 (s, 3H), 3.06 (td, *J* = 7.2, 1.6 Hz, 2H), 2.97 – 2.90 (m, 2H), 2.89 – 2.86 (m, 1H), 2.63 (d, *J* = 2.1 Hz, 3H), 2.54 – 2.47 (m, 2H), 2.36 – 2.31 (m, 1H), 2.29 – 2.23 (m, 1H), 2.19 – 2.15 (m, 1H), 2.06 – 1.97 (m, 1H), 1.85 (d, *J* = 7.1 Hz, 2H), 1.82 (d, *J* = 7.1 Hz, 3H), 1.69 – 1.64 (m, 2H), 1.48 – 1.44 (m, 2H), 1.42 – 1.37 (m, 4H), 1.33 – 1.27 (m, 10H). **^{13}C NMR** (126 MHz, DMSO) δ 172.00, 170.67, 167.94, 159.38, 158.97, 158.15, 156.29, 146.50, 146.27, 145.31, 145.01, 140.96, 135.63, 135.29, 132.19, 132.04, 130.27, 129.25, 120.25, 115.93, 115.57, 107.42, 105.06, 99.47, 71.89, 56.60, 56.36, 52.18, 50.93, 50.51, 50.21, 46.74, 42.91, 31.46, 31.42, 28.99, 28.86, 28.64, 28.61, 28.57, 28.08, 28.00, 26.65, 26.17, 23.34, 23.24, 22.05, 21.69, 21.20, 21.17. HRMS (ESI): calcd for $C_{50}H_{64}F_3N_7O_5S$ $[M+H]^+$, 932.4714 found, 932.4718.

3-(4-((2-(4-((4-(((R)-1-(3-amino-5-(trifluoromethyl)phenyl)ethyl)amino)-7-methoxy-2-methylquinazolin-6-yl)oxy)piperidin-1-yl)-2-oxoethyl)thio)-1-oxoisoindolin-2-yl)piperidine-2,6-dione (B1)

The compound **S5-1** (20 mg, 0.042 mmol) and compound **S10-1** (16.6 mg, 0.046mmol) were added into a 25 mL round single bottle. Then HATU (24 mg, 0.063 mmol), DIEA (20.86μl, 0.126 mmol) and DMF (2 mL) were added and the mixture was stirred at room temperature for 12 h. After the reaction was finished, the mixture was quenched by 1 mL H_2O, followed by purification via preparative HPLC (10% to 90% acetonitrile/0.05% HCl in H_2O) to obtain the compound **B1**. White solid, 6.3 mg (yield 19.0%). **^{1}H NMR** (500 MHz, CD_3OD) δ 8.06 (dd, *J* = 6.2, 3.7 Hz, 1H), 7.80 (dd, *J* = 7.8, 2.4 Hz, 1H), 7.74 (dd, *J* = 7.8, 3.0 Hz, 1H), 7.61 (dd, *J* = 7.5, 4.7 Hz, 1H), 7.31

(d, *J* = 6.6 Hz, 2H), 7.11 – 7.07 (m, 2H), 5.82 (q, *J* = 7.0 Hz, 1H), 5.20 – 5.11 (m, 2H), 4.53 – 4.40 (m, 2H), 4.03 (d, *J* = 1.5 Hz, 3H), 3.85 – 3.76 (m, 2H), 3.65 – 3.52 (m, 2H), 3.35 – 3.32 (m, 2H), 2.96 – 2.86 (m, 2H), 2.84 – 2.76 (m, 2H), 2.63 (s, 3H), 2.59 – 2.45 (m, 2H), 2.22 – 2.15 (m, 2H), 1.75 (dd, *J* = 7.1, 2.1 Hz, 3H). **^{13}C NMR** (126 MHz, CD_3OD) δ 174.74, 172.12, 170.99, 169.12, 160.81, 160.23, 159.48, 149.20, 147.27, 143.94, 137.00, 135.93, 133.42, 131.71, 130.60, 123.57, 123.02, 121.07, 107.96, 106.47, 100.36, 74.76, 57.82, 57.65, 57.48, 57.31, 57.25, 53.73, 52.41, 44.50, 40.28, 36.50, 32.36, 31.95, 31.12, 30.96, 24.08, 22.25, 21.27. HRMS (ESI): calcd for $C_{39}H_{40}F_3N_7O_6S$ $[M+H]^+$, 792.2786 found, 792.2788.

3-(4-((3-(4-((4-(((R)-1-(3-amino-5-(trifluoromethyl)phenyl)ethyl)amino)-7-methoxy-2-methylquinazolin-6-yl)oxy)piperidin-1-yl)-3-oxopropyl)thio)-1-oxoisoindolin-2-yl)piperidine-2,6-dione (B2)

The compound **B2** was obtained from the condensation reaction of **S5-1** and **S10-2** according to the similar process of **B1**. White solid, 5.2 mg (yield 30.7%). **^{1}H NMR** (500 MHz, CD_3OD) δ 8.00 (dd, *J* = 5.6, 2.1 Hz, 1H), 7.71 (d, *J* = 7.7 Hz, 1H), 7.67 (d, *J* = 7.5 Hz, 1H), 7.55 (t, *J* = 2.3 Hz, 1H), 7.08 (s, 1H), 6.99 (t, *J* = 2.0 Hz, 1H), 6.97 (s, 1H), 6.83 (d, *J* = 2.0 Hz, 1H), 5.76 (q, *J* = 3.0 Hz, 1H), 5.22 – 5.10 (m, 2H), 4.51 – 4.41 (m, 2H), 4.01 (s, 3H), 3.86 – 3.79 (m, 1H), 3.77 – 3.65 (m, 1H), 3.63 – 3.53 (m, 1H), 3.50 – 3.40 (m, 1H), 3.38 – 3.32 (m, 2H), 2.93 – 2.86 (m, 1H), 2.82 – 2.75 (m, 3H), 2.63 (s, 3H), 2.59 – 2.49 (m, 1H), 2.22 – 2.15 (m, 1H), 2.04 – 1.92 (m, 2H), 1.85 – 1.73 (m, 2H), 1.71 (d, *J* = 7.1 Hz, 3H). **^{13}C NMR** (126 MHz, CD_3OD) δ 174.67, 171.46, 171.12, 160.78, 160.16, 159.38, 149.14, 143.68, 136.95, 133.25, 131.53, 130.50, 126.79, 122.29, 117.75, 113.50, 111.75, 107.99, 106.41, 100.36, 74.94, 64.29, 57.81, 57.65, 57.47, 57.30, 57.19, 57.13, 53.75, 52.45, 49.62, 43.60, 39.93, 33.88, 32.35, 30.74, 29.55, 24.04, 22.25, 21.23. HRMS (ESI): calcd for $C_{40}H_{42}F_3N_7O_6S$ $[M+H]^+$, 806.2942 found, 806.2941.

3-(4-((4-(4-((4-(((R)-1-(3-amino-5-(trifluoromethyl)phenyl)ethyl)amino)-7-methoxy-2-methylquinazolin-6-yl)oxy)piperidin-1-yl)-4-oxobutyl)thio)-1-oxoisoindolin-2-yl)piperidine-2,6-

dione (B3)

The compound **B3** was obtained from the condensation reaction of **S5-1** and **S10-3** according to the similar process of **B1**. White solid, 4.8 mg (yield 27.9%). **^{1}H NMR** (500 MHz, CD_3OD) δ 8.17 (d, *J* = 4.9 Hz, 1H), 7.70 – 7.66 (m, 3H), 7.61 (d, *J* = 7.3 Hz, 1H), 7.51 (td, *J* = 7.6, 3.1 Hz, 1H), 7.37 (s, 1H), 7.11 (s, 1H), 5.87 (q, *J* = 7.2 Hz, 1H), 5.14 (dd, *J* = 13.0, 4.6 Hz, 1H), 4.96 (s, 1H), 4.48 – 4.38 (m, 2H), 4.02 (s, 3H), 3.93 – 3.85 (m, 1H), 3.79 – 3.71 (m, 1H), 3.60 – 3.47 (m, 2H), 3.15 – 3.09 (m, 2H), 2.94 – 2.84 (m, 1H), 2.81 – 2.74 (m, 1H), 2.63 (s, 3H), 2.61 – 2.56 (m, 2H), 2.51 (td, *J* = 13.2, 5.7 Hz, 1H), 2.21 – 2.15 (m, 1H), 2.06 – 2.00 (m, 2H), 1.99 – 1.93 (m, 2H), 1.81 (d, *J* = 6.2 Hz, 3H), 1.78 – 1.70 (m, 2H). **^{13}C NMR** (126 MHz, CD_3OD) δ 174.66, 172.90, 172.15, 171.09, 160.68, 160.18, 159.36, 149.18, 147.74, 143.18, 136.88, 133.47, 133.13, 132.71, 130.41, 126.21, 124.06, 121.88, 121.86, 120.24, 116.42, 107.96, 107.90, 106.48, 100.33, 74.97, 57.81, 57.63, 57.46, 57.26, 53.71, 52.46, 43.77, 39.92, 32.91, 32.36, 31.13, 26.20, 24.04, 22.28, 21.44. HRMS (ESI): calcd for $C_{41}H_{44}F_3N_7O_6S$ $[M+H]^+$, 820.3099 found, 820.3103.

3-(4-((5-(4-((4-(((R)-1-(3-amino-5-(trifluoromethyl)phenyl)ethyl)amino)-7-methoxy-2-methylquinazolin-6-yl)oxy)piperidin-1-yl)-5-oxopentyl)thio)-1-oxoisoindolin-2-yl)piperidine-2,6-dione (B4)

The compound **B4** was obtained from the condensation reaction of **S5-1** and **S10-4** according to the similar process of **B1**. White solid, 5.3 mg (yield 30.3%). **^{1}H NMR** (500 MHz, CD_3OD) δ 8.10 (d, *J* = 10.0Hz, 1H), 7.63 (dd, *J* = 14.9, 7.6 Hz, 2H), 7.51 (t, *J* = 7.6 Hz, 1H), 7.33 – 7.26 (m, 2H), 7.12 – 7.04 (m, 2H), 5.81 (q, *J* = 7.0 Hz, 1H), 5.18 – 5.10 (m, 2H), 4.51 – 4.37 (m, 2H), 4.04 – 4.00 (m, 3H), 3.82 (dd, *J* = 30.9, 14.8 Hz, 2H), 3.60 – 3.48 (m, 2H), 3.15 – 3.04 (m, 2H), 2.88 (qd, *J* = 14.4, 7.0 Hz, 2H), 2.76 (ddt, *J* = 17.6, 4.6, 2.5 Hz, 2H), 2.63 (s, 3H), 2.56 – 2.48 (m, 1H), 2.45 (t, *J* = 7.1 Hz, 2H), 2.22 – 2.14 (m, 1H), 2.08 – 1.87 (m, 2H), 1.84 – 1.79 (m, 1H), 1.76 (d, *J* = 5.6 Hz, 3H), 1.75 – 1.69 (m, 3H). **^{13}C NMR** (126 MHz, CD_3OD) δ 174.66, 173.60, 172.15, 171.18, 160.76, 160.16, 159.40, 149.18, 146.66, 146.62, 143.34, 136.94, 133.70, 133.10, 132.94, 130.39, 126.67, 124.51, 121.87, 118.94, 115.07, 112.82, 108.04, 106.46, 100.39, 75.04, 57.64, 57.47,

57.30, 57.23, 53.76, 52.47, 43.84, 39.80, 33.43, 32.35, 31.24, 29.85, 25.45, 24.03, 22.28, 21.31. HRMS (ESI): calcd for $C_{42}H_{46}F_3N_7O_6S$ $[M+H]^+$, 834.3255 found, 834.3257.

3-(4-((6-(4-((4-(((R)-1-(3-amino-5-(trifluoromethyl)phenyl)ethyl)amino)-7-methoxy-2-methylquinazolin-6-yl)oxy)piperidin-1-yl)-6-oxohexyl)thio)-1-oxoisoindolin-2-yl)piperidine-2,6-dione (B5)

The compound **B5** was obtained from the condensation reaction of **S5-1** and **S10-5** according to the similar process of **B1**. White solid, 5.1 mg (yield 28.6%). **^{1}H NMR** (500 MHz, CD_3OD) δ 8.13 (d, *J* = 6.7 Hz, 1H), 7.63 – 7.57 (m, 2H), 7.50 (d, *J* = 7.4 Hz, 1H), 7.38 (d, *J* = 14.4 Hz, 2H), 7.12 (d, *J* = 11.9 Hz, 2H), 5.82 (q, *J* = 6.9 Hz, 1H), 5.18 – 5.11 (m, 2H), 4.47 – 4.37 (m, 2H), 4.01 (s, 3H), 3.92 – 3.84 (m, 1H), 3.82 – 3.72 (m, 1H), 3.59 – 3.45 (m, 4H), 3.08 – 3.02 (m, 2H), 2.92 – 2.85 (m, 1H), 2.80 – 2.72 (m, 1H), 2.63 (s, 3H), 2.57 – 2.47 (m, 1H), 2.44 – 2.36 (m, 2H), 2.22 – 2.14 (m, 1H), 2.09 – 1.96 (m, 2H), 1.77 (d, *J* = 7.0 Hz, 3H), 1.69 – 1.60 (m, 4H), 1.54 – 1.47 (m, 2H). **^{13}C NMR** (126 MHz, CD_3OD) δ 174.65, 173.84, 172.14, 171.17, 160.72, 160.15, 159.34, 149.17, 146.76, 143.25, 136.89, 133.89, 133.03, 132.85, 130.35, 126.62, 124.46, 121.78, 119.27, 115.61, 113.12, 107.98, 107.88, 106.46, 100.38, 75.03, 57.81, 57.63, 57.46, 57.25, 53.71, 52.47, 43.89, 39.85, 33.53, 32.36, 31.34, 30.01, 29.16, 26.01, 24.03, 22.29, 21.36. HRMS (ESI): calcd for $C_{43}H_{48}F_3N_7O_6S$ $[M+H]^+$, 848.3412 found, 848.3410.

3-(4-((7-(4-((4-(((R)-1-(3-amino-5-(trifluoromethyl)phenyl)ethyl)amino)-7-methoxy-2-methylquinazolin-6-yl)oxy)piperidin-1-yl)-7-oxoheptyl)thio)-1-oxoisoindolin-2-yl)piperidine-2,6-dione (B6)

The compound **B6** was obtained from the condensation reaction of **S5-1** and **S10-6** according to the similar process of **B1**. White solid, 5.8 mg (yield 32.0%). **^{1}H NMR** (500 MHz, CD_3OD) δ 8.11 (dd, *J* = 5.7, 2.8 Hz, 1H), 7.65 – 7.55 (m, 2H), 7.50 (t, *J* = 7.0 Hz, 1H), 7.25 (d, *J* = 15.5 Hz, 2H), 7.10 (s, 1H), 7.05 – 6.99 (m, 1H), 5.80 (q, *J* = 7.0 Hz, 1H), 5.19 – 5.09 (m, 1H), 4.47 – 4.38 (m,

2H), 4.02 (s, 3H), 3.91 – 3.75 (m, 2H), 3.61 – 3.47 (m, 2H), 3.10 – 3.02 (m, 2H), 2.93 – 2.82 (m, 1H), 2.81 – 2.73 (m, 1H), 2.64 (s, 3H), 2.56 – 2.48 (m, 1H), 2.40 (d, *J* = 7.7 Hz, 2H), 2.21 – 2.14 (m, 1H), 2.08 – 1.97 (m, 2H), 1.86 – 1.79 (m, 1H), 1.75 (d, *J* = 7.1 Hz, 3H), 1.71 – 1.64 (m, 2H), 1.61 – 1.56 (m, 2H), 1.52 – 1.46 (m, 2H), 1.40 – 1.27 (m, 4H). **^{13}C NMR** (126 MHz, CD_3OD) δ 174.66, 174.08, 172.14, 171.20, 160.75, 160.16, 159.36, 149.17, 146.45, 143.17, 136.92, 134.00, 133.01, 132.77, 130.33, 126.74, 124.57, 121.74, 118.27, 114.17, 112.14, 107.87, 107.79, 106.46, 100.39, 75.00, 57.64, 57.47, 57.29, 57.23, 53.71, 52.45, 43.90, 39.80, 33.92, 32.36, 31.28, 30.12, 29.78, 29.25, 26.39, 24.04, 22.29, 21.31. HRMS (ESI): calcd for $C_{44}H_{50}F_3N_7O_6S$ $[M+H]^+$, 862.3568 found, 862.3563.

3-(4-((8-(4-((4-(((R)-1-(3-amino-5-(trifluoromethyl)phenyl)ethyl)amino)-7-methoxy-2-methylquinazolin-6-yl)oxy)piperidin-1-yl)-8-oxooctyl)thio)-1-oxoisoindolin-2-yl)piperidine-2,6-dione (B7)

The compound **B7** was obtained from the condensation reaction of **S5-1** and **S10-7** according to the similar process of **B1**. White solid, 5.4 mg (yield 29.4%). **^{1}H NMR** (500 MHz, CD_3OD) δ 8.10 (d, *J* = 1.5 Hz 1H), 7.65 – 7.56 (m, 2H), 7.54 – 7.46 (m, 1H), 7.27 – 7.18 (m, 2H), 7.09 (s, 1H), 7.03 – 6.98 (m, 1H), 5.80 (q, *J* = 6.6 Hz, 1H), 5.18 – 5.12 (m, 1H), 4.49 – 4.36 (m, 2H), 4.02 (s, 3H), 3.91 – 3.75 (m, 2H), 3.62 – 3.48 (m, 2H), 3.10 – 3.02 (m, 2H), 2.94 – 2.83 (m, 1H), 2.82 – 2.74 (m, 1H), 2.63 (s, 3H), 2.58 – 2.47 (m, 1H), 2.41 (t, *J* = 7.6 Hz, 2H), 2.21 – 2.14 (m, 1H), 2.10 – 1.96 (m, 2H), 1.81 (d, *J* = 30.5 Hz, 2H), 1.74 (d, *J* = 7.1 Hz, 3H), 1.70 – 1.63 (m, 2H), 1.62 – 1.55 (m, 2H), 1.51 – 1.44 (m, 2H), 1.38 – 1.32 (m, 4H). **^{13}C NMR** (126 MHz, CD_3OD) δ 174.67, 174.15, 172.15, 171.23, 160.77, 160.16, 159.37, 149.18, 146.36, 143.18, 136.92, 134.00, 133.00, 132.73, 130.32, 126.77, 124.61, 121.72, 117.97, 113.73, 111.89, 107.86, 107.76, 106.44, 100.36, 74.97, 57.81, 57.64, 57.47, 57.21, 53.72, 52.42, 43.89, 39.79, 33.97, 33.63, 32.36, 31.16, 30.12, 29.83, 29.33, 26.46, 24.03, 22.28, 21.27. HRMS (ESI): calcd for $C_{45}H_{52}F_3N_7O_6S$ $[M+H]^+$, 876.3725 found, 876.3721.

3-(4-((9-(4-((4-(((R)-1-(3-amino-5-(trifluoromethyl)phenyl)ethyl)amino)-7-methoxy-2-methylquinazolin-6-yl)oxy)piperidin-1-yl)-9-oxononyl)thio)-1-oxoisoindolin-2-yl)piperidine-2,6-dione (B8)

The compound **B8** was obtained from the condensation reaction of **S5-1** and **S10-8** according to the similar process of **B1**. White solid, 5.6 mg (yield 30.0%). **^{1}H NMR** (500 MHz, CD_3OD) δ 8.10 (dd, 1H), 7.64 – 7.56 (m, 2H), 7.53 – 7.44 (m, 3H), 7.22 (d, *J* = 8.0 Hz, 1H), 7.07 (d, *J* = 2.8 Hz, 1H), 5.84 (q, *J* = 7.0 Hz, 1H), 5.19 – 5.11 (m, 1H), 4.94 – 4.90 (m, 1H), 4.47 – 4.37 (m, 2H), 4.02 (s, 3H), 3.83 (q, *J* = 12.8 Hz, 2H), 3.62 – 3.51 (m, 2H), 3.10 – 3.02 (m, 2H), 2.94 – 2.84 (m, 1H), 2.81 – 2.74 (m, 1H), 2.63 (s, 3H), 2.58 – 2.47 (m, 1H), 2.42 (dt, *J* = 11.8, 5.5 Hz, 2H), 2.22 – 2.14 (m, 1H), 2.09 (d, *J* = 4.1 Hz, 1H), 2.06 – 1.98 (m, 1H), 1.91 – 1.79 (m, 2H), 1.77 (d, *J* = 7.1 Hz, 3H), 1.70 – 1.64 (m, 2H), 1.62 – 1.56 (m, 2H), 1.50 – 1.44 (m, 2H), 1.37 – 1.32 (m, 6H). **^{13}C NMR** (126 MHz, CD_3OD) δ 174.69, 174.22, 172.19, 171.24, 160.82, 160.19, 159.45, 149.21, 146.31, 143.14, 136.95, 134.06, 133.02, 132.63, 130.32, 124.61, 121.69, 117.88, 113.55, 111.84, 107.86, 106.43, 100.35, 75.05, 57.82, 57.65, 57.48, 57.20, 53.71, 52.37, 43.87, 39.75, 34.04, 33.59, 32.36, 31.22, 31.12, 30.19, 29.93, 29.47, 26.55, 24.04, 22.28, 21.21. HRMS (ESI): calcd for $C_{46}H_{54}F_3N_7O_6S$ $[M+H]^+$, 890.3881 found, 890.3889.

3-(4-((10-(4-((4-(((R)-1-(3-amino-5-(trifluoromethyl)phenyl)ethyl)amino)-7-methoxy-2-methylquinazolin-6-yl)oxy)piperidin-1-yl)-10-oxodecyl)thio)-1-oxoisoindolin-2-yl)piperidine-2,6-dione (B9)

The compound **B9** was obtained from the condensation reaction of **S5-1** and **S10-9** according to the similar process of **B1**. White solid, 6.6 mg (yield 34.8%). **^{1}H NMR** (500 MHz, CD_3OD) δ 8.04 (dd, 1H), 7.59 (d, *J* = 6.9 Hz, 2H), 7.51 – 7.46 (m, 1H), 7.07 (d, *J* = 6.7 Hz, 1H), 6.99 (d, *J* = 18.7 Hz, 2H), 6.82 (s, 1H), 5.76 (q, 1H), 5.19 – 5.10 (m, 1H), 4.66 – 4.59 (m, 1H), 4.45 – 4.35 (m, 2H), 4.01 (s, 3H), 3.89 – 3.79 (m, 2H), 3.61 – 3.52 (m, 2H), 3.04 (t, *J* = 7.2 Hz, 2H), 2.94 – 2.84 (m, 1H), 2.83 – 2.74 (m, 1H), 2.63 (s, 3H), 2.57 – 2.48 (m, 1H), 2.45 – 2.39 (m, 2H), 2.21 – 2.14 (m, 1H), 2.12 –

2.06 (m, 1H), 2.04 – 1.98 (m, 1H), 1.87 – 1.83 (m, 1H), 1.81 – 1.76 (m, 1H), 1.72 (d, *J* = 7.0 Hz, 3H), 1.68 – 1.63 (m, 2H), 1.61 – 1.56 (m, 2H), 1.48 – 1.43 (m, 2H), 1.41 – 1.36 (m, 2H), 1.34 – 1.30 (m, 6H). **^{13}C NMR** (126 MHz, CD_3OD) δ 174.68, 174.21, 172.18, 171.22, 160.75, 160.14, 159.33, 149.17, 146.13, 143.04, 136.89, 134.08, 132.99, 132.48, 130.29, 126.87, 124.71, 121.64, 117.11, 112.58, 111.11, 107.88, 107.76, 106.42, 100.36, 74.98, 57.64, 57.47, 57.30, 57.20, 53.70, 52.44, 43.90, 39.78, 34.05, 33.56, 32.36, 32.20, 31.26, 30.28, 30.19, 29.99, 29.53, 26.58, 24.04, 22.28, 21.26. HRMS (ESI): calcd for $C_{47}H_{56}F_3N_7O_6S$ $[M+H]^+$, 904.4038 found, 904.4037.

3-(4-((11-(4-((4-(((R)-1-(3-amino-5-(trifluoromethyl)phenyl)ethyl)amino)-7-methoxy-2-methylquinazolin-6-yl)oxy)piperidin-1-yl)-11-oxoundecyl)thio)-1-oxoisoindolin-2-yl)piperidine-2,6-dione (B10)

The compound **B10** was obtained from the condensation reaction of **S5-1** and **S10-10** according to the similar process of **B1**. White solid, 5.8 mg (yield 30.1%). **^{1}H NMR** (500 MHz, CD_3OD) δ 8.11 (d, *J* = 4.9 Hz, 1H), 7.59 – 7.53 (m, 2H), 7.50 – 7.43 (m, 1H), 7.25 (d, *J* = 1.9 Hz, 1H), 7.22 (d, *J* = 2.7 Hz, 1H), 7.09 (s, 1H), 7.01 (d, *J* = 1.9 Hz, 1H), 5.79 (q, *J* = 7.0 Hz, 1H), 5.14 (dd, *J* = 13.3, 5.2 Hz, 1H), 4.93 – 4.91 (m, 1H), 4.44 – 4.34 (m, 2H), 4.01 (s, 3H), 3.94 – 3.86 (m, 1H), 3.86 – 3.79 (m, 1H), 3.61 – 3.53 (m, 2H), 3.02 (t, *J* = 6.5 Hz, 2H), 2.93 – 2.84 (m, 1H), 2.81 – 2.74 (m, 1H), 2.63 (s, 3H), 2.56 – 2.47 (m, 1H), 2.45 – 2.38 (m, 2H), 2.21 – 2.14 (m, 1H), 2.14 – 2.06 (m, 1H), 2.06 – 1.98 (m, 1H), 1.88 – 1.82 (m, 1H), 1.80 – 1.77 (m, 1H), 1.75 (d, *J* = 7.0 Hz, 3H), 1.67 – 1.62 (m, 2H), 1.62 – 1.57 (m, 2H), 1.48 – 1.41 (m, 2H), 1.35 – 1.28 (m, 10H). **^{13}C NMR** (126 MHz, CD_3OD) δ 174.64, 174.19, 172.18, 171.16, 160.69, 160.11, 159.30, 159.28, 149.15, 146.46, 142.88, 136.86, 134.10, 132.96, 132.27, 130.27, 126.75, 124.59, 121.55, 118.17, 113.99, 112.04, 107.90, 107.80, 106.42, 100.35, 75.03, 74.99, 57.63, 57.46, 57.29, 57.23, 53.71, 53.69, 52.48, 43.96, 39.84, 34.08, 33.50, 32.35, 30.36, 30.08, 29.59, 26.61, 24.03, 22.29, 21.35, 21.31. HRMS (ESI): calcd for $C_{48}H_{58}F_3N_7O_6S$ $[M+H]^+$, 918.4194 found, 918.4195.

3-(4-((12-(4-((4-(((R)-1-(3-amino-5-(trifluoromethyl)phenyl)ethyl)amino)-7-methoxy-2-methylquinazolin-6-yl)oxy)piperidin-1-yl)-12-oxododecyl)thio)-1-oxoisoindolin-2-yl)piperidine-2,6-dione (B11)

The compound **B11** was obtained from the condensation reaction of **S5-1** and **S10-11** according to the similar process of **B1**. White solid, 4.7 mg (yield 24.0%). **^{1}H NMR** (500 MHz, CD_3OD) δ 8.04 (dd, *J* = 5.0 Hz, 1H), 7.61 – 7.58 (m, 2H), 7.49 (q, *J* = 6.1 Hz, 1H), 7.20 – 7.14 (m, 2H), 7.07 (d, *J* = 2.9 Hz, 1H), 6.99 (s, 1H), 5.79 (q, *J* = 6.3 Hz, 1H), 5.15 (dd, *J* = 13.4, 5.1 Hz, 1H), 4.45 – 4.36 (m, 2H), 4.02 (s, 3H), 3.88 – 3.79 (m, 2H), 3.63 – 3.53 (m, 2H), 3.04 (t, *J* = 7.2 Hz, 2H), 2.94 – 2.85 (m, 1H), 2.81 – 2.75 (m, 1H), 2.63 (s, 3H), 2.57 – 2.48 (m, 1H), 2.47 – 2.39 (m, 2H), 2.21 – 2.14 (m, 1H), 2.12 – 2.05 (m, 1H), 2.04 – 1.98 (m, 1H), 1.91 – 1.83 (m, 1H), 1.82 – 1.76 (m, 1H), 1.74 (d, *J* = 7.1 Hz, 3H), 1.69 – 1.64 (m, 2H), 1.63 – 1.58 (m, 2H), 1.48 – 1.43 (m, 2H), 1.37 – 1.33 (m, 4H), 1.32 – 1.29 (m, 8H). **^{13}C NMR** (126 MHz, CD_3OD) δ 174.68, 174.23, 172.20, 171.22, 160.79, 160.16, 159.41, 149.20, 146.18, 142.97, 136.92, 134.13, 133.01, 132.38, 130.29, 126.85, 123.72, 121.61, 117.37, 112.84, 111.33, 107.92, 107.78, 106.43, 100.36, 75.01, 57.65, 57.47, 57.30, 57.20, 53.71, 52.42, 44.44, 43.88, 39.76, 33.53, 32.36, 30.44, 30.11, 29.60, 29.42, 28.48, 27.23, 26.63, 24.04, 22.28, 21.24, 19.94, -8.87. HRMS(ESI): calcd for $C_{49}H_{60}F_3N_7O_6S$ $[M+H]^+$, 932.4531 found, 932.4535.

3-(4-((2-((S)-3-((4-(((R)-1-(3-amino-5-(trifluoromethyl)phenyl)ethyl)amino)-7-methoxy-2-methylquinazolin-6-yl)oxy)pyrrolidin-1-yl)ethyl)thio)-1-oxoisoindolin-2-yl)piperidine-2,6-dione (C1)

The compound **S5-2** (20 mg, 0.04 mmol) and compound **S9-1** (19.9 mg, 0.031 mmol) were added into a 25 mL round single bottle. Then NaI (13 mg,0.052 mmol), K_2CO_3 (12 mg,0.052 mmol) and DMF (2 mL) were added and stirred at 80 ℃ for 12 h. After the reaction was finished, the mixture was filtered, followed by purification via preparative HPLC (10% to 90% acetonitrile/0.05% HCl in H_2O) to obtain the compound **C1**. White solid, 5.2 mg (yield 15.7%).

^{1}H NMR (500 MHz, CD_3OD) δ 8.19 (d, *J* = 15.8 Hz, 1H), 7.85 – 7.71 (m, 2H), 7.60 (dd, *J* = 17.4, 9.5 Hz, 1H), 7.29 (d, *J* = 12.7 Hz, 2H), 7.12 – 7.02 (m, 2H), 5.81 (q, *J* = 7.0 Hz, 1H), 5.45 (s, 1H), 5.19 (dd, *J* = 13.3, 5.2 Hz, 1H), 4.52 (q, *J* = 17.4 Hz, 2H), 4.08 – 3.90 (m, 5H), 3.64 – 3.37 (m, 7H), 2.92 (ddd, *J* = 18.5, 13.6, 5.4 Hz, 1H), 2.81 – 2.76 (m, 1H), 2.64 (s, 3H), 2.58 – 2.44 (m, 2H), 2.19 (s, 1H), 1.77 (d, *J* = 7.1 Hz, 3H). **^{13}C NMR** (126 MHz, CD_3OD) δ 174.64, 172.22, 170.80, 161.25, 160.27, 158.96, 147.80, 144.55, 144.53, 137.62, 134.45, 133.69, 133.23, 132.97, 130.82, 130.61, 126.16, 124.00, 123.40, 121.20, 117.01, 109.28, 106.37, 100.71, 78.53, 60.19, 57.64, 57.47, 57.29, 57.13, 53.81, 52.67, 32.36, 29.09, 25.21, 24.09, 22.35, 21.56. HRMS (ESI): calcd for $C_{38}H_{40}F_3N_7O_5S$ $[M+H]^+$, 764.2836 found, 764.2835.

3-(4-((3-((S)-3-((4-(((R)-1-(3-amino-5-(trifluoromethyl)phenyl)ethyl)amino)-7-methoxy-2-methylquinazolin-6-yl)oxy)pyrrolidin-1-yl)propyl)thio)-1-oxoisoindolin-2-yl)piperidine-2,6-dione (C2)

The compound **C2** was obtained from the nucleophilic substitution of **S5-2** and **S9-2** according to the similar process of **C1**. White solid, 4.8 mg (yield 14.3%). **^{1}H NMR** (500 MHz, CD_3OD) δ 8.24 (dd, *J* = 4.7, 14.6 Hz, 1H), 7.77 – 7.62 (m, 2H), 7.60 – 7.50 (m, 1H), 7.48 – 7.40 (m, 2H), 7.13 (d, *J* = 21.0 Hz, 2H), 5.83 (q, *J* = 6.9 Hz, 1H), 5.52 – 5.45 (m, 1H), 5.22 – 5.12 (m, 1H), 4.56 – 4.40 (m, 2H), 4.02 (d, *J* = 5.1 Hz, 3H), 4.01 – 3.96 (m, 1H), 3.94 – 3.82 (m, 1H), 3.70 – 3.56 (m, 1H), 3.56 – 3.40 (m, 3H), 3.39 – 3.32 (m, 1H), 3.26 – 3.13 (m, 2H), 2.97 – 2.85 (m, 1H), 2.81 – 2.75 (m, 1H), 2.64 (d, *J* = 6.0 Hz, 3H), 2.58 – 2.40 (m, 2H), 2.20 – 2.05 (m, 3H), 1.80 (d, *J* = 5.4 Hz, 3H). **^{13}C NMR** (126 MHz, CD_3OD) δ 174.64, 172.29, 171.02, 161.26, 160.30, 158.87, 148.31, 144.03, 137.61, 133.70, 133.43, 133.17, 132.29, 130.61, 125.96, 125.04, 123.80, 122.71, 122.60, 118.63, 109.01, 106.37, 100.63, 78.42, 60.27, 57.64, 57.47, 57.33, 56.43, 53.74, 52.61, 32.36, 31.30, 30.68, 26.58, 24.08, 22.32, 21.55. HRMS (ESI): calcd for $C_{39}H_{42}F_3N_7O_5S$ $[M+H]^+$, 778.2993 found, 778.2998.

3-(4-((4-((S)-3-((4-(((R)-1-(3-amino-5-(trifluoromethyl)phenyl)ethyl)amino)-7-methoxy-2-methylquinazolin-6-yl)oxy)pyrrolidin-1-yl)butyl)thio)-1-oxoisoindolin-2-yl)piperidine-2,6-dione (C3)

The compound **C3** was obtained from the nucleophilic substitution of **S5-2** and **S9-3** according to the similar process of **C1**. White solid, 5.5 mg (yield 16.0%). **^{1}H NMR** (500 MHz, CD_3OD) δ 8.29 (d, *J* = 1.9 Hz, 1H), 7.72 – 7.67 (m, 1H), 7.66 – 7.60 (m, 3H), 7.57 – 7.50 (m, 1H), 7.33 – 7.25 (m, 1H), 7.11 (d, *J* = 10.6 Hz, 1H), 5.87 (q, *J* = 7.0 Hz, 1H), 5.55 – 5.47 (m, 1H), 5.17 (ddd, *J* = 10.1, 6.4, 3.7 Hz, 1H), 4.49 (d, *J* = 17.3 Hz, 1H), 4.47 – 4.43 (m, 1H), 4.43 (d, *J* = 10.0 Hz, 1H), 4.02 (d, *J* = 16.2 Hz, 3H), 3.99 – 3.80 (m, 2H), 3.65 – 3.52 (m, 1H), 3.45 – 3.34 (m, 2H), 3.20 – 3.10 (m, 2H), 2.95 – 2.88 (m, 1H), 2.83 – 2.73 (m, 2H), 2.64 (s, 3H), 2.58 – 2.42 (m, 2H), 2.23 – 2.14 (m, 1H), 1.99 – 1.90 (m, 2H), 1.82 (d, *J* = 2.3 Hz, 3H), 1.79 – 1.72 (m, 2H). **^{13}C NMR** (126 MHz, CD_3OD) δ 174.66, 172.27, 171.07, 161.22, 160.29, 158.95, 148.29, 148.12, 143.66, 137.63, 133.36, 133.22, 133.10, 130.84, 130.47, 126.05, 124.30, 123.89, 122.21, 117.88, 109.00, 108.77, 106.39, 100.65, 78.44, 57.64, 57.47, 57.36, 57.30, 53.73, 52.63, 33.08, 32.37, 31.98, 31.28, 27.33, 25.73, 24.07, 22.33, 21.56. HRMS (ESI): calcd for $C_{40}H_{44}F_3N_7O_5S$ $[M+H]^+$, 792.3149 found, 792.3153.

3-(4-((5-((S)-3-((4-(((R)-1-(3-amino-5-(trifluoromethyl)phenyl)ethyl)amino)-7-methoxy-2-methylquinazolin-6-yl)oxy)pyrrolidin-1-yl)pentyl)thio)-1-oxoisoindolin-2-yl)piperidine-2,6-dione (C4)

The compound **C4** was obtained from the nucleophilic substitution of **S5-2** and **S9-4** according to the similar process of **C1**. White solid, 6.1 mg (yield 17.5%). **^{1}H NMR** (500 MHz, CD_3OD) δ 8.26 (d, *J* = 10.3 Hz, 1H), 7.68 – 7.62 (m, 2H), 7.58 – 7.51 (m, 3H), 7.27 – 7.23 (m, 1H), 7.12 – 7.08 (m, 1H), 5.86 (q, *J* = 7.1 Hz, 1H), 5.52 – 5.46 (m, 1H), 5.20 – 5.14 (m, 1H), 4.47 (d, *J* = 17.3 Hz, 1H), 4.45 – 4.41 (m, 1H), 4.40 (d, *J* = 9.7 Hz 1H), 4.05 – 3.96 (m, 4H), 3.93 – 3.82 (m, 1H), 3.63 – 3.52 (m, 1H), 3.46 – 3.39 (m, 1H), 3.29 – 3.23 (m, 2H), 3.18 – 3.02 (m, 3H), 2.95 – 2.87

(m, 1H), 2.83 – 2.74 (m, 2H), 2.64 (d, *J* = 2.4 Hz, 3H), 2.58 – 2.43 (m, 2H), 2.34 (dt, *J* = 14.9, 7.4 Hz, 1H), 2.22 – 2.15 (m, 1H), 1.83 – 1.80 (m, 3H), 1.79 – 1.69 (m, 4H), 1.61 – 1.55 (m, 2H). ^{13}**C NMR** (126 MHz, CD_3OD) δ 174.62, 172.31, 171.14, 161.23, 160.26, 158.90, 148.28, 147.14, 143.53, 137.57, 133.53, 133.20, 133.15, 132.82, 130.42, 126.44, 122.04, 108.82, 106.39, 100.63, 78.46, 60.13, 57.82, 57.64, 57.47, 57.30, 57.13, 56.64, 54.23, 53.84, 53.71, 52.68, 33.41, 32.37, 31.23, 29.64, 26.38, 26.29, 24.07, 22.31, 21.48. HRMS (ESI): calcd for $C_{41}H_{46}F_3N_7O_5S$ $[M+H]^+$, 806.3306 found, 806.3309.

3-(4-((6-((S)-3-((4-(((R)-1-(3-amino-5-(trifluoromethyl)phenyl)ethyl)amino)-7-methoxy-2-methylquinazolin-6-yl)oxy)pyrrolidin-1-yl)hexyl)thio)-1-oxoisoindolin-2-yl)piperidine-2,6-dione (C5)

The compound **C5** was obtained from the nucleophilic substitution of **S5-2** and **S9-5** according to the similar process of **C1**. White solid, 4.9 mg (yield 13.8%). ^{1}**H NMR** (500 MHz, CD_3OD) δ 8.22 (dd, *J* = 5.9, 3.1 Hz, 1H), 7.69 – 7.62 (m, 2H), 7.56 – 7.49 (m, 1H), 7.43 (s, 2H), 7.15 (s, 1H), 7.12 – 7.09 (m, 1H), 5.84 (q, *J* = 7.3 Hz, 1H), 5.49 – 5.43 (m, 1H), 5.19 – 5.12 (m, 1H), 4.47 (d, *J* = 6.2 Hz, 1H), 4.46 – 4.42 (m, 1H), 4.40 (d, *J* = 8.3 Hz, 1H), 4.03 (d, *J* = 4.1 Hz, 3H), 3.99 – 3.80 (m, 2H), 3.58 – 3.50 (m, 1H), 3.46 – 3.33 (m, 1H), 3.25 – 3.14 (m, 2H), 3.14 – 3.02 (m, 2H), 2.94 – 2.85 (m, 1H), 2.84 – 2.69 (m, 2H), 2.66 – 2.63 (m, 3H), 2.58 – 2.49 (m, 1H), 2.47 – 2.41 (m, 1H), 2.34 (dt, *J* = 14.4, 7.3 Hz, 1H), 2.22 – 2.15 (m, 1H), 1.84 – 1.78 (m, 3H), 1.75 – 1.66 (m, 4H), 1.57 – 1.49 (m, 2H), 1.45 – 1.38 (m, 2H). ^{13}**C NMR** (126 MHz, CD_3OD) δ 174.59, 172.32, 171.14, 161.23, 160.30, 158.95, 147.81, 143.54, 137.61, 133.83, 133.30, 133.11, 130.40, 123.23, 121.96, 116.90, 108.64, 106.40, 100.63, 100.42, 78.62, 78.44, 60.12, 57.65, 57.47, 57.41, 57.31, 53.85, 53.73, 52.64, 33.58, 32.38, 31.98, 31.28, 29.99, 28.88, 28.79, 26.94, 26.64, 24.06, 22.32, 21.49. HRMS (ESI): calcd for $C_{42}H_{48}F_3N_7O_5S$ $[M+H]^+$, 820.3462 found, 820.3458.

3-(4-((7-((S)-3-((4-(((R)-1-(3-amino-5-(trifluoromethyl)phenyl)ethyl)amino)-7-methoxy-2-methylquinazolin-6-yl)oxy)pyrrolidin-1-yl)heptyl)thio)-1-oxoisoindolin-2-yl)piperidine-2,6-dione

(C6)

The compound **C6** was obtained from the nucleophilic substitution of **S5-2** and **S9-6** according to the similar process of **C1**. White solid, 5.3 mg (yield 14.7%). **^{1}H NMR** (500 MHz, CD_3OD) δ 8.23 (d, *J* = 10.9 Hz, 1H), 7.67 – 7.60 (m, 2H), 7.55 – 7.43 (m, 3H), 7.18 (s, 1H), 7.12 – 7.08 (m, 1H), 5.84 (q, *J* = 7.0 Hz, 1H), 5.50 – 5.43 (m, 1H), 5.19 – 5.13 (m, 1H), 4.49 – 4.37 (m, 2H), 4.03 (d, *J* = 8.7 Hz, 3H), 4.02 – 3.93 (m, 1H), 3.91 – 3.80 (m, 1H), 3.61 – 3.51 (m, 1H), 3.46 – 3.35 (m, 1H), 3.24 (dt, *J* = 10.6, 5.6 Hz, 1H), 3.12 – 3.01 (m, 2H), 2.94 – 2.86 (m, 1H), 2.80 (dd, *J* = 4.7, 2.3 Hz, 1H), 2.79 – 2.74 (m, 1H), 2.64 (d, *J* = 1.6 Hz, 3H), 2.59 – 2.41 (m, 2H), 2.39 – 2.30 (m, 1H), 2.22 – 2.14 (m, 1H), 1.79 (dt, *J* = 7.2, 1.4 Hz, 3H), 1.70 (dt, *J* = 14.6, 7.6 Hz, 4H), 1.50 (s, 2H), 1.39 (q, *J* = 3.5 Hz, 4H). **^{13}C NMR** (126 MHz, CD_3OD) δ 174.61, 172.27, 171.18, 161.23, 160.27, 158.92, 148.31, 148.20, 147.30, 143.27, 137.60, 133.95, 133.08, 132.85, 130.35, 121.81, 108.81, 108.60, 106.38, 100.63, 78.45, 60.04, 57.89, 57.64, 57.47, 57.30, 57.13, 54.24, 53.81, 53.73, 52.66, 33.54, 32.37, 31.25, 30.14, 30.07, 29.44, 29.14, 27.21, 26.63, 24.05, 22.31, 21.45. HRMS (ESI): calcd for $C_{43}H_{50}F_3N_7O_5S$ $[M+H]^+$, 834.3619 found, 834.3614.

3-(4-((8-((S)-3-((4-(((R)-1-(3-amino-5-(trifluoromethyl)phenyl)ethyl)amino)-7-methoxy-2-methylquinazolin-6-yl)oxy)pyrrolidin-1-yl)octyl)thio)-1-oxoisoindolin-2-yl)piperidine-2,6-dione (C7)

The compound **C7** was obtained from the nucleophilic substitution of **S5-2** and **S9-7** according to the similar process of **C1**. White solid, 7.2 mg (yield 19.6%). **^{1}H NMR** (500 MHz, CD_3OD) δ 8.33 (d, *J* = 11.2 Hz, 1H), 7.81 – 7.71 (m, 2H), 7.66 – 7.58 (m, 2H), 7.55 – 7.49 (m, 1H), 7.41 (s, 1H), 7.17 – 7.09 (m, 1H), 5.88 (q, *J* = 7.3 Hz, 1H), 5.61 – 5.46 (m, 1H), 5.21 – 5.10 (m, 1H), 4.45 (d, *J* = 19.6 Hz, 1H), 4.43 – 4.41 (m, 1H), 4.39 (d, *J* = 17.3 Hz, 1H), 4.03 (s, 3H), 4.02 – 3.95 (m, 1H), 3.92 – 3.82 (m, 1H), 3.68 – 3.54 (m, 1H), 3.53 – 3.37 (m, 1H), 3.28 – 3.21 (m, 1H), 3.14 – 3.00 (m, 2H), 2.97 – 2.86 (m, 1H), 2.86 – 2.69 (m, 2H), 2.64 (s, 3H), 2.59 – 2.43 (m, 2H), 2.40 – 2.29 (m, 1H), 2.24 – 2.13 (m, 1H), 1.83 (d, *J* = 6.7 Hz, 3H), 1.80 – 1.72 (m, 2H), 1.70 – 1.63 (m, 2H), 1.51 – 1.44 (m, 2H), 1.37 – 1.33 (m, 4H), 1.23 – 1.21 (m, 2H). **^{13}C NMR** (126 MHz,

CD_3OD) δ 174.64, 172.24, 171.15, 161.15, 160.23, 158.88, 148.28, 147.68, 143.13, 137.54, 134.00, 133.02, 132.90, 132.71, 130.34, 126.23, 124.07, 121.69, 120.35, 116.31, 108.84, 108.68, 106.36, 100.65, 78.43, 57.88, 57.47, 57.29, 56.90, 53.78, 52.67, 33.54, 32.38, 31.98, 31.27, 30.72, 30.09, 29.82, 29.33, 27.35, 26.72, 24.03, 22.35, 21.53. HRMS (ESI): calcd for $C_{44}H_{52}F_3N_7O_5S$ $[M+H]^+$, 848.3775 found, 848.3778.

3-(4-((9-((S)-3-((4-(((R)-1-(3-amino-5-(trifluoromethyl)phenyl)ethyl)amino)-7-methoxy-2-methylquinazolin-6-yl)oxy)pyrrolidin-1-yl)nonyl)thio)-1-oxoisoindolin-2-yl)piperidine-2,6-dione (C8)

The compound **C8** was obtained from the nucleophilic substitution of **S5-2** and **S9-8** according to the similar process of **C1**. White solid, 6.6 mg (yield17.7%). **^{1}H NMR** (500 MHz, CD_3OD) δ 8.30 (d, *J* = 12.1 Hz, 1H), 7.65 – 7.57 (m, 2H), 7.56 – 7.43 (m, 3H), 7.22 – 7.09 (m, 2H), 5.83 (q, *J* = 6.8 Hz, 1H), 5.56 – 5.47 (m, 1H), 5.19 – 5.11 (m, 1H), 4.47 – 4.35 (m, 2H), 4.03 (s, 3H), 3.92 – 3.82 (m, 1H), 3.67 – 3.50 (m, 1H), 3.48 – 3.35 (m, 1H), 3.21 (q, *J* = 7.3 Hz, 3H), 3.10 – 3.01 (m, 2H), 2.96 – 2.70 (m, 3H), 2.64 (s, 3H), 2.58 – 2.43 (m, 2H), 2.40 – 2.29 (m, 1H), 2.23 – 2.14 (m, 1H), 1.80 (d, *J* = 6.4 Hz, 3H), 1.70 – 1.62 (m, 2H), 1.49 – 1.43 (m, 2H), 1.34 – 1.32 (m, 7H), 1.31 – 1.27 (m, 3H), 1.22 (d, *J* = 6.3 Hz, 1H). **^{13}C NMR** (126 MHz, CD_3OD) δ 174.62, 172.23, 171.20, 161.20, 160.25, 158.90, 148.29, 147.07, 143.13, 137.58, 134.06, 133.06, 132.77, 132.66, 130.32, 126.47, 124.31, 121.70, 120.74, 117.41, 114.38, 108.85, 108.63, 106.37, 100.64, 78.47, 57.96, 57.64, 57.47, 57.31, 53.74, 52.68, 33.55, 32.37, 31.95, 31.25, 30.18, 29.93, 29.43, 27.55, 27.44, 26.83, 24.04, 22.32, 21.46. HRMS (ESI): calcd for $C_{45}H_{54}F_3N_7O_5S$ $[M+H]^+$, 862.3932 found, 862.3929.

3-(4-((10-((S)-3-((4-(((R)-1-(3-amino-5-(trifluoromethyl)phenyl)ethyl)amino)-7-methoxy-2-methylquinazolin-6-yl)oxy)pyrrolidin-1-yl)decyl)thio)-1-oxoisoindolin-2-yl)piperidine-2,6-dione (C9)

The compound **C9** was obtained from the nucleophilic substitution of **S5-2** and **S9-9** according to the similar process of **C1**. White solid, 6.1 mg (yield 16.1%). **^{1}H NMR** (500 MHz, CD_3OD) δ 8.18 (d, *J* = 4.1 Hz, 1H), 7.66 – 7.61 (m, 2H), 7.55 – 7.49 (m, 1H), 7.31 – 7.23 (m, 2H), 7.10 (d, *J* = 4.7 Hz, 1H), 7.03 (s, 1H), 5.82 (q, *J* = 7.0 Hz, 1H), 5.48 – 5.42 (m, 1H), 5.16 (dd, *J* = 13.4, 5.1 Hz, 1H), 4.49 – 4.35 (m, 2H), 4.03 (d, *J* = 3.6 Hz, 3H), 4.02 – 3.97 (m, 1H), 3.94 – 3.82 (m, 1H), 3.60 – 3.53 (m, 1H), 3.47 – 3.40 (m, 1H), 3.27 – 3.23 (m, 1H), 3.10 – 3.02 (m, 2H), 2.95 – 2.85 (m, 1H), 2.81 – 2.78 (m, 1H), 2.78 – 2.74 (m, 1H), 2.64 (s, 3H), 2.58 – 2.42 (m, 2H), 2.41 – 2.29 (m, 1H), 2.23 – 2.14 (m, 1H), 1.77 (d, *J* = 7.1 Hz, 3H), 1.69 – 1.63 (m, 2H), 1.49 – 1.43 (m, 2H), 1.40 – 1.28 (m, 12H). **^{13}C NMR** (126 MHz, CD_3OD) δ 174.72, 172.30, 172.21, 171.20, 161.17, 160.26, 158.89, 148.30, 148.12, 143.02, 137.56, 134.05, 133.32, 132.99, 132.54, 130.33, 124.33, 123.87, 122.52, 121.65, 117.89, 108.79, 108.66, 106.38, 100.68, 78.45, 78.40, 57.64, 57.53, 57.46, 57.29, 53.75, 52.63, 33.53, 32.37, 30.28, 30.16, 30.08, 29.47, 27.55, 27.45, 26.85, 26.79, 24.04, 22.36, 21.60. HRMS (ESI): calcd for $C_{46}H_{56}F_3N_7O_5S$ $[M+H]^+$, 876.4088 found, 876.4094.

3-(4-((11-((S)-3-((4-(((R)-1-(3-amino-5-(trifluoromethyl)phenyl)ethyl)amino)-7-methoxy-2-methylquinazolin-6-yl)oxy)pyrrolidin-1-yl)undecyl)thio)-1-oxoisoindolin-2-yl)piperidine-2,6-dione (C10)

The compound **C10** was obtained from the nucleophilic substitution of **S5-2** and **S9-10** according to the similar process of **C1**. White solid, 7.3 mg (yield 19.0%). **^{1}H NMR** (500 MHz, CD_3OD) δ 8.24 (d, *J* = 7.5 Hz, 1H), 7.66 – 7.60 (m, 2H), 7.55 – 7.49 (m, 1H), 7.49 – 7.42 (m, 2H), 7.17 (s, 1H), 7.11 (d, *J* = 2.2 Hz, 1H), 5.84 (q, *J* = 7.1 Hz, 1H), 5.52 – 5.45 (m, 1H), 5.16 (dd, *J* = 13.3, 5.1 Hz, 1H), 4.47 – 4.37 (m, 2H), 4.03 (s, 3H), 4.03 – 3.97 (m, 1H), 3.94 – 3.82 (m, 1H), 3.63 – 3.53 (m, 1H), 3.47 – 3.36 (m, 1H), 3.29 – 3.24 (m, 1H), 3.10 – 3.02 (m, 2H), 2.96 – 2.85 (m, 1H), 2.81 – 2.79 (m, 1H), 2.79 – 2.74 (m, 1H), 2.64 (s, 3H), 2.58 – 2.43 (m, 2H), 2.39 – 2.31 (m, 1H), 2.22 – 2.15 (m, 1H), 1.79 (d, *J* = 2.1 Hz, 3H), 1.78 – 1.71 (m, 2H), 1.66 (p, *J* = 7.3 Hz, 2H), 1.49 – 1.43 (m, 2H), 1.37 – 1.28 (m, 11H), 1.24 – 1.20 (m, 1H). **^{13}C NMR** (126 MHz, CD_3OD) δ 174.64, 172.20, 171.21, 161.20, 160.21, 158.86, 149.38, 146.64 – 145.84 (m), 143.08, 137.58, 134.09, 133.05, 132.54, 130.31, 123.55, 121.67, 117.59, 116.78, 113.29, 111.44, 108.76, 106.35, 100.66,

78.49, 60.18, 57.64, 57.47, 57.29, 56.96, 53.73, 52.74, 33.54, 32.36, 31.90, 31.21, 30.38, 30.20, 30.10, 30.05, 29.49, 27.58, 26.80, 25.24, 24.25, 24.04, 22.31, 21.41. HRMS (ESI): calcd for $C_{47}H_{58}F_3N_7O_5S$ $[M+H]^+$, 890.4245 found, 890.4247.

3-(4-((12-((S)-3-((4-(((R)-1-(3-amino-5-(trifluoromethyl)phenyl)ethyl)amino)-7-methoxy-2-methylquinazolin-6-yl)oxy)pyrrolidin-1-yl)dodecyl)thio)-1-oxoisoindolin-2-yl)piperidine-2,6-dione (C11)

The compound **C11** was obtained from the nucleophilic substitution of **S5-2** and **S9-11** according to the similar process of **C1**, 6.7 mg (yield 17.1%). White solid. **^{1}H NMR** (500 MHz, CD_3OD) δ 8.28 (d, *J* = 12.1 Hz, 1H), 7.63 – 7.60 (m, 2H), 7.55 – 7.48 (m, 3H), 7.22 (s, 1H), 7.12 (d, *J* = 13.7 Hz, 1H), 5.84 (q, *J* = 7.4 Hz, 1H), 5.55 – 5.47 (m, 1H), 5.16 (dd, *J* = 13.4, 4.7 Hz, 1H), 4.46 – 4.37 (m, 2H), 4.03 (s, 3H), 3.95 – 3.82 (m, 1H), 3.64 – 3.57 (m, 1H), 3.56 – 3.35 (m, 2H), 3.27 – 3.17 (m, 1H), 3.07 – 3.03 (m, 2H), 2.94 – 2.86 (m, 2H), 2.80 – 2.76 (m, 1H), 2.64 (s, 3H), 2.58 – 2.45 (m, 2H), 2.39 – 2.31 (m, 1H), 2.21 – 2.16 (m, 1H), 1.81 (d, *J* = 6.7 Hz, 3H), 1.68 – 1.63 (m, 2H), 1.47 – 1.43 (m, 2H), 1.35 – 1.28 (m, 15H), 1.22 (d, *J* = 6.7 Hz, 1H). **^{13}C NMR** (126 MHz, CD_3OD) δ 174.65, 172.19, 171.18, 161.17, 160.22, 158.89, 148.26, 148.18, 147.16, 143.00, 137.56, 134.09, 133.02, 132.47, 130.31, 126.44, 124.28, 121.64, 120.90, 120.75, 117.88, 108.92, 108.66, 106.37, 100.69, 78.48, 60.23, 58.02, 57.63, 57.46, 57.39, 53.75, 52.71, 33.56, 32.38, 31.97, 31.28, 30.48, 30.42, 30.21, 30.14, 29.54, 27.60, 26.81, 25.65, 24.04, 22.35, 21.56. HRMS (ESI): calcd for $C_{48}H_{60}F_3N_7O_5S$ $[M+H]^+$, 904.4401 found, 904.4396.

3-(4-((2-((S)-3-((4-(((R)-1-(3-amino-5-(trifluoromethyl)phenyl)ethyl)amino)-7-methoxy-2-methylquinazolin-6-yl)oxy)pyrrolidin-1-yl)-2-oxoethyl)thio)-1-oxoisoindolin-2-yl)piperidine-2,6-dione (D1)

The compound **S5-2** (20 mg, 0.026 mmol) and compound **S10-1** (14.5 mg, 0.026 mmol) were added into a 25 mL round single bottle. Then HATU (24 mg,0.039 mmol), DIEA (10.9mg,0.052 mmol) and DMF (2 mL) were added and the mixture was stirred at room temperature for 12 h. After the reaction was finished, the mixture was quenched by 1 mL H_2O, followed by purification via preparative HPLC (10% to 90% acetonitrile/0.05% HCl in H_2O) to obtain the compound **D1**. White solid, 6.0 mg (yield 17.8%). **^{1}H NMR** (500 MHz, CD_3OD) δ 7.90 (dd, *J* = 19.3, 35.1 Hz, 1H), 7.82 – 7.72 (m, 2H), 7.59 – 7.46 (m, 1H), 7.07 (d, *J* = 2.7 Hz, 1H), 7.03 (d, *J* = 5.6 Hz, 2H), 6.89 (s, 1H), 5.79 (q, *J* = 7.1 Hz, 1H), 5.18 (q, *J* = 12.3 Hz, 1H), 5.13 (qd, *J* = 11.1, 4.9 Hz, 1H), 4.60 – 4.39 (m, 2H), 4.01 (s, 3H), 3.90 (d, *J* = 5.2 Hz, 1H), 3.88 – 3.81 (m, 2H), 3.81 – 3.76 (m, 2H), 3.74 – 3.69 (m, 1H), 2.93 – 2.82 (m, 1H), 2.81 – 2.69 (m, 1H), 2.64 (s, 3H), 2.53 – 2.45 (m, 1H), 2.39 – 2.23 (m, 2H), 2.18 – 2.13 (m, 1H), 1.75 – 1.71 (m, 3H). **^{13}C NMR** (126 MHz, CD_3OD) δ 174.66, 172.15, 170.94, 169.44, 160.94, 160.16, 159.13, 148.88, 147.20, 144.89, 137.07, 133.28, 132.98, 132.73, 130.52, 126.46, 124.30, 123.63, 123.38, 120.55, 117.32, 108.40, 107.74, 106.39, 100.60, 78.61, 57.81, 57.63, 57.46, 57.33, 53.63, 52.58, 46.29, 45.75, 32.30, 24.05, 22.31, 21.34. HRMS (ESI): calcd for $C_{38}H_{38}F_3N_7O_6S$ $[M+H]^+$, 778.2629 found, 778.2631.

3-(4-((3-((S)-3-((4-(((R)-1-(3-amino-5-(trifluoromethyl)phenyl)ethyl)amino)-7-methoxy-2-methylquinazolin-6-yl)oxy)pyrrolidin-1-yl)-3-oxopropyl)thio)-1-oxoisoindolin-2-yl)piperidine-2,6-dione (D2)

The compound **D2** was obtained from the condensation reaction of **S5-2** and **S10-2** according to the similar process of **D1**. White solid, 5.5 mg (yield 16.0%). **^{1}H NMR** (500 MHz, CD_3OD) δ 7.89 (dd, *J* = 5.3, 7.6 Hz, 1H), 7.76 – 7.61 (m, 2H), 7.55 – 7.48 (m, 1H), 7.19 (d, *J* = 8.7 Hz, 2H), 7.08 – 6.99 (m, 2H), 5.80 (q, *J* = 7.2 Hz, 1H), 5.25 – 5.08 (m, 2H), 4.48 – 4.32 (m, 2H), 3.99 (d, *J* = 6.2 Hz, 3H), 3.83 – 3.76 (m, 1H), 3.69 (dd, *J* = 23.1, 12.3 Hz, 2H), 3.63 – 3.56 (m, 1H), 3.41 – 3.33 (m, 2H), 2.90 – 2.85 (m, 1H), 2.78 – 2.67 (m, 3H), 2.63 (d, *J* = 2.0 Hz, 3H), 2.49 (dq, *J* = 14.1, 6.7 Hz, 1H), 2.29 (d, *J* = 24.6 Hz, 2H), 2.19 – 2.13 (m, 1H), 1.78 – 1.72 (m, 3H). **^{13}C NMR** (126 MHz, CD_3OD) δ 174.63, 172.34, 172.04, 171.07, 160.88, 160.27, 159.19, 149.11, 148.30, 143.92, 137.17, 133.55, 133.25, 132.32, 130.49, 125.97, 124.81, 123.81, 122.38, 122.00, 118.43,

107.87, 107.55, 106.42, 100.45, 78.64, 57.47, 57.33, 53.69, 52.48, 46.06, 45.24, 35.02, 32.35, 31.06, 29.22, 24.02, 22.30, 21.41. HRMS (ESI): calcd for $C_{39}H_{40}F_3N_7O_6S$ $[M+H]^+$, 792.2786 found, 792.2785.

3-(4-((4-((S)-3-((4-(((R)-1-(3-amino-5-(trifluoromethyl)phenyl)ethyl)amino)-7-methoxy-2-methylquinazolin-6-yl)oxy)pyrrolidin-1-yl)-4-oxobutyl)thio)-1-oxoisoindolin-2-yl)piperidine-2,6-dione (D3)

The compound **D3** was obtained from the condensation reaction of **S5-2** and **S10-3** according to the similar process of **D1**. White solid, 4.8 mg (yield 13.8%). **^{1}H NMR** (500 MHz, CD_3OD) δ 7.98 (dd, *J* = 5.1, 10.3 Hz, 1H), 7.70 – 7.61 (m, 2H), 7.59 – 7.50 (m, 1H), 7.23 (d, *J* = 8.6 Hz, 2H), 7.05 (d, *J* = 4.2 Hz, 2H), 5.81 (q, *J* = 7.0 Hz, 1H), 5.29 (d, *J* = 34.8 Hz, 1H), 5.17 – 5.14 (m, 1H), 4.53 – 4.40 (m, 2H), 3.98 (d, *J* = 3.0 Hz, 3H), 3.85 – 3.80 (m, 1H), 3.70 (dt, *J* = 18.5, 12.4 Hz, 3H), 3.58 – 3.44 (m, 1H), 3.35 (s, 1H), 3.18 – 3.11 (m, 2H), 2.93 – 2.86 (m, 1H), 2.82 – 2.77 (m, 1H), 2.63 (s, 3H), 2.57 – 2.52 (m, 2H), 2.38 – 2.33 (m, 1H), 2.20 – 2.16 (m, 1H), 2.02 – 1.97 (m, 2H), 1.78 – 1.73 (m, 3H). **^{13}C NMR** (126 MHz, CD_3OD) δ 174.65, 173.55, 172.18, 171.11, 160.81, 160.21, 159.09, 149.04, 148.16, 142.91, 137.08, 133.55, 133.08, 132.47, 130.36, 126.03, 124.28, 123.87, 122.33, 121.67, 117.90, 107.73, 107.58, 106.39, 100.42, 78.45, 57.46, 57.35, 53.72, 52.48, 46.11, 45.13, 33.89, 32.56, 32.37, 30.96, 25.75, 24.04, 22.32, 21.43. HRMS (ESI): calcd for $C_{40}H_{42}F_3N_7O_6S$ $[M+H]^+$, 806.2942 found, 806.2939.

3-(4-((5-((S)-3-((4-(((R)-1-(3-amino-5-(trifluoromethyl)phenyl)ethyl)amino)-7-methoxy-2-methylquinazolin-6-yl)oxy)pyrrolidin-1-yl)-5-oxopentyl)thio)-1-oxoisoindolin-2-yl)piperidine-2,6-dione (D4)

The compound **D4** was obtained from the condensation reaction of **S5-2** and **S10-4** according to the similar process of **D4**. White solid, 6.8 mg (yield 19.2%). **^{1}H NMR** (500 MHz, CD_3OD) δ 7.94 (dd, *J* = 4.8, 19.6 Hz, 1H), 7.64 – 7.58 (m, 2H), 7.52 – 7.47 (m, 1H), 7.11 (d, *J* = 3.7 Hz, 2H),

7.05 – 7.02 (m, 1H), 6.95 (s, 1H), 5.80 (q, *J* = 7.3 Hz, 1H), 5.20 – 5.15 (m, 2H), 4.45 – 4.39 (m, 2H), 4.00 – 3.97 (m, 3H), 3.86 – 3.76 (m, 2H), 3.76 – 3.64 (m, 3H), 3.58 – 3.45 (m, 1H), 3.09 – 3.04 (m, 2H), 2.92 – 2.87 (m, 1H), 2.79 – 2.75 (m, 1H), 2.66 (s, 3H), 2.64 – 2.64 (m, 1H), 2.61 (d, *J* = 2.0 Hz, 1H), 2.55 – 2.49 (m, 1H), 2.43 – 2.41 (m, 1H), 2.23 – 2.15 (m, 2H), 1.80 – 1.77 (m, 2H), 1.74 – 1.72 (m, 3H). **^{13}C NMR** (126 MHz, CD_3OD) δ 174.68, 174.26, 172.29, 171.15, 160.86, 160.23, 159.09, 149.06, 147.69, 143.33, 137.08, 133.76, 133.25, 133.00, 130.33, 124.07, 122.63, 121.90, 121.81, 120.07, 116.38, 107.71, 107.38, 106.37, 100.39, 78.41, 57.47, 57.30, 53.69, 52.45, 46.10, 45.09, 34.56, 33.48, 32.35, 30.92, 29.82, 24.85, 24.02, 22.29, 21.34. HRMS (ESI): calcd for $C_{41}H_{44}F_3N_7O_6S$ $[M+H]^+$, 820.3099 found, 820.3100.

3-(4-((6-((S)-3-((4-(((R)-1-(3-amino-5-(trifluoromethyl)phenyl)ethyl)amino)-7-methoxy-2-methylquinazolin-6-yl)oxy)pyrrolidin-1-yl)-6-oxohexyl)thio)-1-oxoisoindolin-2-yl)piperidine-2,6-dione (D5)

The compound **D5** was obtained from the condensation reaction of **S5-2** and **S10-5** according to the similar process of **D1**. White solid, 6.4 mg (yield 17.7%). **^{1}H NMR** (500 MHz, CD_3OD) δ 7.97 (dd, *J* = 5.0, 24.6 Hz, 1H), 7.64 – 7.60 (m, 2H), 7.52 – 7.48 (m, 1H), 7.18 (d, *J* = 3.7 Hz, 2H), 7.06 – 7.04 (m, 1H), 7.00 (s, 1H), 5.81 (q, *J* = 4.7 Hz,1H), 5.26 – 5.15 (m, 2H), 4.44 – 4.41 (m, 2H), 4.00 (d, *J* = 1.7 Hz, 3H), 3.87 – 3.78 (m, 2H), 3.75 – 3.66 (m, 3H), 3.56 – 3.46 (m, 1H), 3.08 – 3.03 (m, 2H), 2.93 – 2.86 (m, 1H), 2.80 – 2.76 (m, 1H), 2.66 (d, *J* = 1.2 Hz, 1H), 2.63 (dd, *J* = 2.6, 1.2 Hz, 3H), 2.56 – 2.49 (m, 1H), 2.39 – 2.36 (m, 2H), 2.32 – 2.23 (m, 2H), 2.18 (d, *J* = 13.7 Hz, 2H), 1.74 (dd, *J* = 7.1, 2.5 Hz, 3H), 1.67 (d, *J* = 11.5 Hz, 2H).**^{13}C NMR** (126 MHz, CD_3OD) δ 174.69, 174.50, 172.19, 171.15, 160.81, 160.21, 159.10, 149.08, 147.97, 143.19, 137.06, 133.91, 133.27, 133.01, 132.75, 130.33, 126.10, 123.95, 121.71, 107.61, 107.47, 106.41, 106.37, 100.39, 78.43, 57.81, 57.64, 57.46, 57.30, 53.73, 52.48, 46.13, 45.09, 34.96, 33.51, 32.36, 30.01, 29.18, 25.37, 24.02, 22.29, 21.40. HRMS (ESI): calcd for $C_{42}H_{46}F_3N_7O_6S$ $[M+H]^+$, 834.3255 found, 834.3258.

3-(4-((7-((S)-3-((4-(((R)-1-(3-amino-5-(trifluoromethyl)phenyl)ethyl)amino)-7-methoxy-2-methylquinazolin-6-yl)oxy)pyrrolidin-1-yl)-7-oxoheptyl)thio)-1-oxoisoindolin-2-yl)piperidine-2,6-dione (D6)

The compound **D6** was obtained from the condensation reaction of **S5-2** and **S10-6** according to the similar process of **D1**. White solid, 5.8 mg (yield 15.8%). **^{1}H NMR** (500 MHz, CD_3OD) δ 7.93 (dd, *J* = 5.3, 24.6 Hz, 1H), 7.61 – 7.56 (m, 2H), 7.51 – 7.46 (m, 1H), 7.05 – 7.03 (m, 2H), 7.02 (d, *J* = 1.7 Hz, 1H), 6.90 (s, 1H), 5.79 (q, *J* = 5.3 Hz, 1H), 5.20 – 5.13 (m, 2H), 4.44 – 4.37 (m, 2H), 3.99 (d, *J* = 4.7 Hz, 3H), 3.90 – 3.78 (m, 2H), 3.78 – 3.67 (m, 3H), 3.60 – 3.44 (m, 1H), 3.08 – 3.03 (m, 2H), 2.93 – 2.86 (m, 1H), 2.82 – 2.76 (m, 1H), 2.67 – 2.65 (m, 1H), 2.64 – 2.62 (m, 3H), 2.53 (d, *J* = 14.0 Hz, 1H), 2.41 – 2.31 (m, 4H), 2.24 – 2.14 (m, 2H), 1.74 – 1.71 (m, 3H), 1.69 – 1.63 (m, 4H). **^{13}C NMR** (126 MHz, CD_3OD) δ 174.69, 174.59, 172.25, 171.15, 160.83, 160.24, 159.14, 149.12, 149.04, 148.32, 143.02, 137.12, 134.01, 132.96, 132.53, 130.29, 125.98, 124.70, 123.82, 121.64, 121.60, 118.32, 107.66, 107.42, 106.36, 100.36, 78.45, 57.47, 57.30, 53.72, 52.33, 46.17, 45.08, 35.00, 33.45, 32.36, 30.01, 29.56, 29.27, 25.77, 24.03, 22.29, 21.39. HRMS (ESI): calcd for $C_{43}H_{48}F_3N_7O_6S$ $[M+H]^+$, 848.3412 found, 848.3410.

3-(4-((8-((S)-3-((4-(((R)-1-(3-amino-5-(trifluoromethyl)phenyl)ethyl)amino)-7-methoxy-2-methylquinazolin-6-yl)oxy)pyrrolidin-1-yl)-8-oxooctyl)thio)-1-oxoisoindolin-2-yl)piperidine-2,6-dione (D7)

The compound **D7** was obtained from the condensation reaction of **S5-2** and **S10-7** according to the similar process of **D1**. White solid, 6.2 mg (yield 16.6%). **^{1}H NMR** (500 MHz, CD_3OD) δ 7.97 (dd, *J* = 2.3, 14.7 Hz, 1H), 7.63 – 7.58 (m, 2H), 7.52 – 7.46 (m, 1H), 7.07 – 7.02 (m, 3H), 6.89 (d, *J* = 3.8 Hz, 1H), 5.78 (q, *J* = 6.8 Hz, 1H), 5.27 – 5.11 (m, 2H), 4.46 – 4.36 (m, 2H), 4.00 (d, *J* = 2.3 Hz, 3H), 3.89 – 3.81 (m, 1H), 3.82 – 3.78 (m, 1H), 3.77 – 3.72 (m, 1H), 3.72 – 3.68 (m, 1H), 3.07 – 3.01 (m, 2H), 2.94 – 2.84 (m, 1H), 2.81 – 2.75 (m, 1H), 2.63 (d, *J* = 2.2 Hz, 3H), 2.52 (qd, *J* = 13.3, 4.7 Hz, 1H), 2.40 – 2.34 (m, 2H), 2.33 – 2.22 (m, 2H), 2.20 – 2.15 (m, 1H), 1.74 – 1.70 (m, 3H), 1.69 – 1.59 (m, 4H), 1.52 – 1.43 (m, 2H), 1.37 – 1.34 (m, 2H), 1.33 – 1.28 (m, 2H).

^{13}C NMR (126 MHz, CD_3OD) δ 174.83, 174.75, 172.22, 171.22, 160.92, 160.15, 159.04, 150.34, 146.09, 143.08, 137.09, 133.98, 132.96, 132.60, 130.32, 126.85, 124.69, 121.67, 117.20, 114.35, 112.64, 111.16, 107.82, 107.28, 100.45, 78.40, 57.47, 57.24, 53.71, 52.41, 46.11, 45.04, 35.13, 33.55, 32.34, 30.09, 30.02, 29.77, 29.33, 28.67, 25.79, 24.00, 22.29, 21.17. HRMS (ESI): calcd for $C_{44}H_{50}F_3N_7O_6S$ $[M+H]^+$, 862.3568 found, 862.3566.

3-(4-((9-((S)-3-((4-(((R)-1-(3-amino-5-(trifluoromethyl)phenyl)ethyl)amino)-7-methoxy-2-methylquinazolin-6-yl)oxy)pyrrolidin-1-yl)-9-oxononyl)thio)-1-oxoisoindolin-2-yl)piperidine-2,6-dione (D8)

The compound **D8** was obtained from the condensation reaction of **S5-2** and **S10-8** according to the similar process of **D1**. White solid, 5.1 mg (yield 13.5%). **^{1}H NMR** (500 MHz, CD_3OD) δ 7.99 (dd, *J* = 6.2, 34.8 Hz, 1H), 7.62 – 7.58 (m, 2H), 7.49 (dd, *J* = 9.7, 5.6 Hz, 1H), 7.29 (s, 2H), 7.09 (s, 1H), 7.06 – 7.04 (m, 1H), 5.81 (q, *J* = 3.1 Hz, 1H), 5.31 – 5.21 (m, 1H), 5.16 – 5.12 (m, 1H), 4.43 – 4.38 (m, 2H), 4.00 (d, *J* = 3.2 Hz, 3H), 3.85 – 3.77 (m, 2H), 3.77 – 3.65 (m, 3H), 3.60 – 3.42 (m, 1H), 3.05 – 3.02 (m, 2H), 2.90 – 2.85 (m, 1H), 2.80 – 2.76 (m, 1H), 2.63 (d, *J* = 1.2 Hz, 3H), 2.55 – 2.49 (m, 1H), 2.39 – 2.35 (m, 3H), 2.32 – 2.25 (m, 2H), 2.22 – 2.16 (m, 2H), 1.76 – 1.73 (m, 3H), 1.68 – 1.52 (m, 8H). **^{13}C NMR** (126 MHz, CD_3OD) δ 174.81, 174.70, 172.20, 171.17, 160.84, 160.16, 159.06, 149.05, 147.28, 142.94, 137.06, 134.06, 132.96, 132.40, 130.30, 126.40, 124.24, 121.61, 121.10, 114.88, 107.77, 107.44, 106.38, 106.34, 100.42, 78.44, 57.46, 57.32, 57.30, 53.72, 52.35, 46.15, 45.06, 35.21, 33.55, 32.60, 32.36, 30.86, 30.12, 29.88, 29.46, 25.90, 24.03, 22.30, 21.34. HRMS (ESI): calcd for $C_{45}H_{52}F_3N_7O_6S$ $[M+H]^+$, 876.3725 found, 876.3727.

3-(4-((10-((S)-3-((4-(((R)-1-(3-amino-5-(trifluoromethyl)phenyl)ethyl)amino)-7-methoxy-2-methylquinazolin-6-yl)oxy)pyrrolidin-1-yl)-10-oxodecyl)thio)-1-oxoisoindolin-2-yl)piperidine-2,6-dione (D9)

The compound **D9** was obtained from the condensation reaction of **S5-2** and **S10-9** according to the similar process of **D1**. White solid, 6.8 mg (yield 17.7%). **^{1}H NMR** (500 MHz, CD_3OD) δ 7.93 (d, *J* = 1.7 Hz, 1H), 7.62 – 7.58 (m, 2H), 7.50 (d, *J* = 7.8 Hz, 1H), 7.08 (d, *J* =29.8 Hz, 2H), 7.04 (d, *J* = 2.3 Hz, 1H), 6.95 (d, *J* = 1.5 Hz, 1H), 5.79 (q, *J* = 1.2 Hz, 1H), 5.28 (d, *J* = 1.4 Hz, 1H), 5.17 – 5.13 (m, 1H), 4.43 – 4.37 (m, 2H), 4.00 (d, *J* = 3.3 Hz, 3H), 3.84 – 3.78 (m, 2H), 3.76 – 3.67 (m, 3H), 3.59 – 3.48 (m, 1H), 3.05 – 3.02 (m, 2H), 2.91 – 2.86 (m, 1H), 2.80 – 2.76 (m, 1H), 2.63 (d, *J* = 1.1 Hz, 3H), 2.38 – 2.31 (m, 4H), 1.72 (d, *J* = 7.1 Hz, 3H), 1.64 – 1.60 (m, 4H), 1.33 – 1.28 (m, 10H). **^{13}C NMR** (126 MHz, CD_3OD) δ 174.82, 174.68, 172.19, 171.19, 160.84, 160.24, 159.17, 149.04, 148.04, 143.00, 137.11, 134.09, 133.36, 132.99, 132.40, 130.29, 126.10, 123.94, 123.74, 121.61, 117.42, 107.73, 107.47, 106.38, 100.39, 78.46, 57.81, 57.64, 57.47, 57.29, 53.72, 52.45, 46.14, 45.07, 35.42, 35.23, 33.52, 32.36, 30.26, 30.18, 29.99, 29.52, 25.94, 24.03, 22.28, 21.36. HRMS (ESI): calcd for $C_{46}H_{54}F_3N_7O_6S$ $[M+H]^+$, 890.3881 found, 890.3883.

3-(4-((11-((S)-3-((4-(((R)-1-(3-amino-5-(trifluoromethyl)phenyl)ethyl)amino)-7-methoxy-2-methylquinazolin-6-yl)oxy)pyrrolidin-1-yl)-11-oxoundecyl)thio)-1-oxoisoindolin-2-yl)piperidine-2,6-dione (D10)

The compound **D10** was obtained from the condensation reaction of **S5-2** and **S10-10** according to the similar process of **D1**. White solid, 6.1 mg (yield 15.6%). **^{1}H NMR** (500 MHz, CD_3OD) δ 7.94 (d, *J* = 19.8 Hz, 1H), 7.61 (t, *J* = 6.8 Hz, 2H), 7.50 (t, *J* = 7.8 Hz, 1H), 7.05 (d, *J* = 4.6 Hz, 1H), 6.97 (d, *J* = 6.1 Hz, 2H), 6.84 (s, 1H), 5.77 (q, *J* = 5.9 Hz, 1H), 5.19 – 5.13 (m, 2H), 4.47 – 4.35 (m, 2H), 4.00 (d, *J* = 3.2 Hz, 3H), 3.84 – 3.80 (m, 1H), 3.79 – 3.70 (m, 2H), 3.71 – 3.64 (m, 1H), 3.35 (s, 2H), 3.04 (t, *J* = 7.2 Hz, 2H), 2.81 – 2.75 (m, 1H), 2.66 (s, 1H), 2.63 (s, 3H), 2.53 (dd, *J* = 13.3, 4.7 Hz, 1H), 2.39 – 2.36 (m, 2H), 2.32 – 2.25 (m, 1H), 1.71 (d, *J* = 7.1 Hz, 3H), 1.66 – 1.61 (m, 4H), 1.47 – 1.43 (m, 2H), 1.32 – 1.28 (m, 10H). **^{13}C NMR** (126 MHz, CD_3OD) δ 174.85, 174.69, 172.19, 171.21, 160.87, 160.26, 159.21, 149.02, 147.61, 143.01, 137.15, 134.09, 133.27, 133.01, 132.46, 130.30, 126.25, 124.09, 122.35, 121.63, 116.14, 107.78, 107.41, 106.36, 100.41, 78.46, 57.82, 57.64, 57.47, 57.25, 53.72, 52.30, 46.13, 45.04, 35.45, 35.25, 33.55, 32.62, 32.36, 30.36, 30.18, 30.03, 29.53, 25.96, 24.03, 22.27, 21.27. HRMS (ESI): calcd for

$C_{47}H_{56}F_3N_7O_6S$ $[M+H]^+$, 904.4038 found, 904.4041.

3-(4-((12-((S)-3-((4-(((R)-1-(3-amino-5-(trifluoromethyl)phenyl)ethyl)amino)-7-methoxy-2-methylquinazolin-6-yl)oxy)pyrrolidin-1-yl)-12-oxododecyl)thio)-1-oxoisoindolin-2-yl)piperidine-2,6-dione (D11)

The compound **D11** was obtained from the condensation reaction of **S5-2** and **S10-11** according to the similar process of **D1**. White solid, 4.6 mg (yield 11.6%). **^{1}H NMR** (500 MHz, CD_3OD) δ 8.03 (d, *J* = 20.3 Hz, 1H), 7.60 (dd, *J* = 7.6, 4.5 Hz, 2H), 7.52 – 7.48 (m, 1H), 7.34 – 7.29 (m, 2H), 7.11 – 7.06 (m, 2H), 5.81 (q, *J* = 7.0 Hz, 1H), 5.35 – 5.25 (m, 1H), 5.17 – 5.13 (m, 1H), 4.45 – 4.37 (m, 2H), 4.00 (d, *J* = 2.0 Hz, 3H), 3.84 – 3.80 (m, 1H), 3.79 – 3.67 (m, 3H), 3.05 – 3.02 (m, 2H), 2.93 – 2.86 (m, 1H), 2.81 – 2.76 (m, 1H), 2.63 (s, 3H), 2.56 – 2.50 (m, 1H), 2.39 – 2.36 (m, 2H), 2.34 – 2.29 (m, 1H), 2.28 – 2.24 (m, 1H), 2.20 – 2.16 (m, 1H), 1.75 (dd, *J* = 7.1, 3.4 Hz, 3H), 1.66 – 1.58 (m, 5H), 1.48 – 1.39 (m, 3H), 1.30 – 1.25 (m, 10H). **^{13}C NMR** (126 MHz, CD_3OD) δ 174.82, 174.69, 172.19, 171.19, 160.82, 160.21, 159.13, 149.11, 149.03, 147.82, 142.93, 137.08, 134.10, 132.99, 132.36, 130.29, 126.17, 123.99, 123.20, 121.59, 116.97, 107.72, 107.47, 106.37, 100.41, 78.44, 57.46, 57.30, 53.72, 52.52, 46.16, 45.08, 35.48, 35.27, 33.51, 32.62, 32.36, 30.46, 30.43, 30.33, 30.21, 30.10, 30.08, 29.58, 25.98, 24.04, 22.30, 21.38. HRMS (ESI): calcd for $C_{48}H_{58}F_3N_7O_6S$ $[M+H]^+$, 918.4194 found, 918.4197.

2.3 Purity spectrum and mass spectrum of the compounds

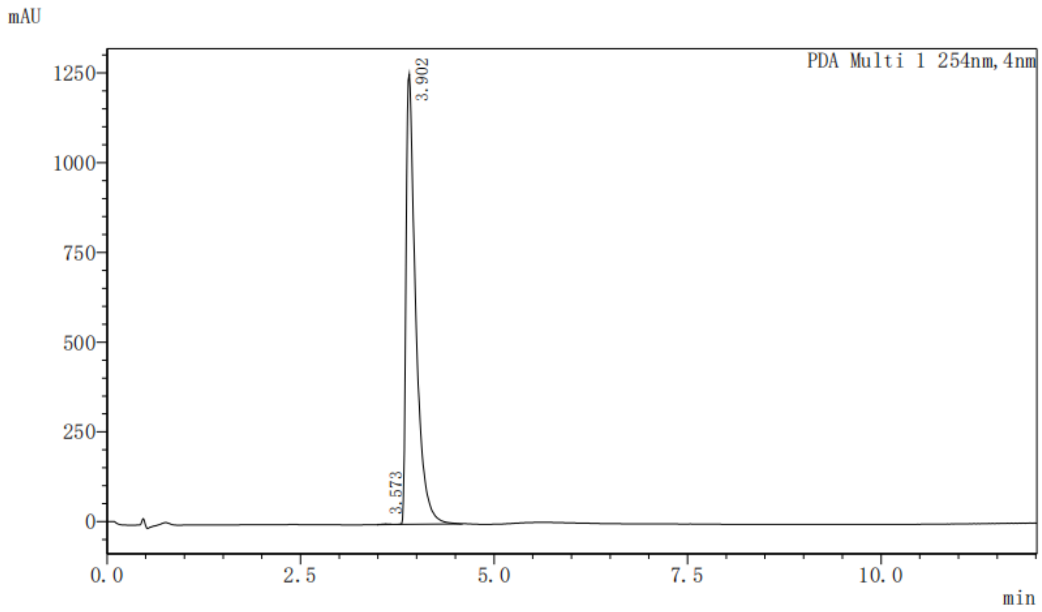

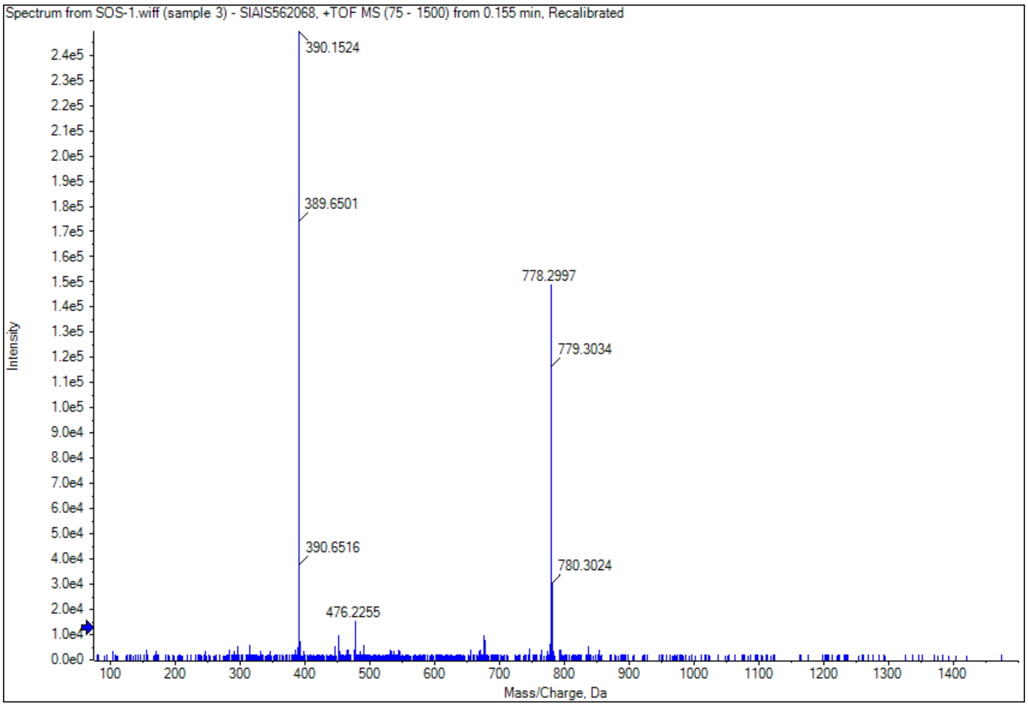

Figure S13. Purity spectrum and mass spectrum of compound **A1**.

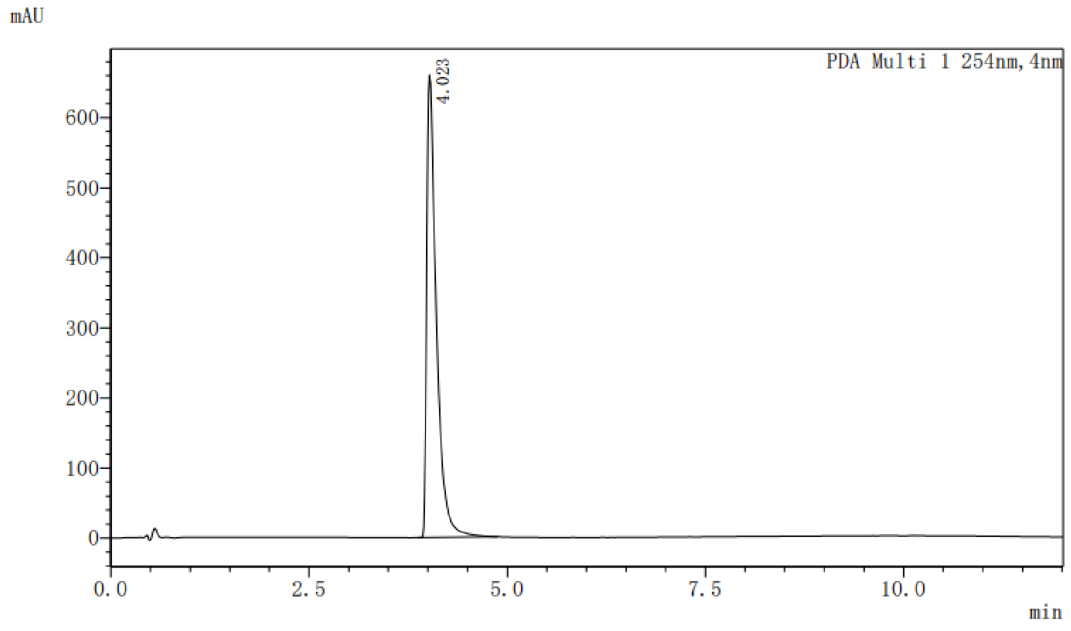

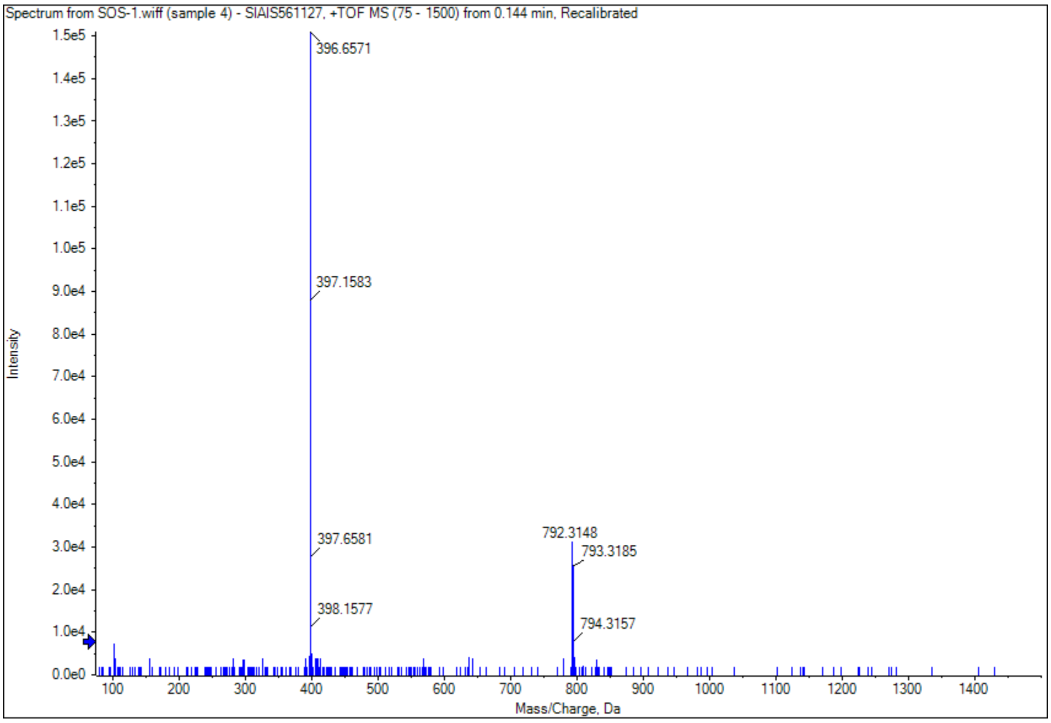

Figure S14. Purity spectrum and mass spectrum of compound **A2**.

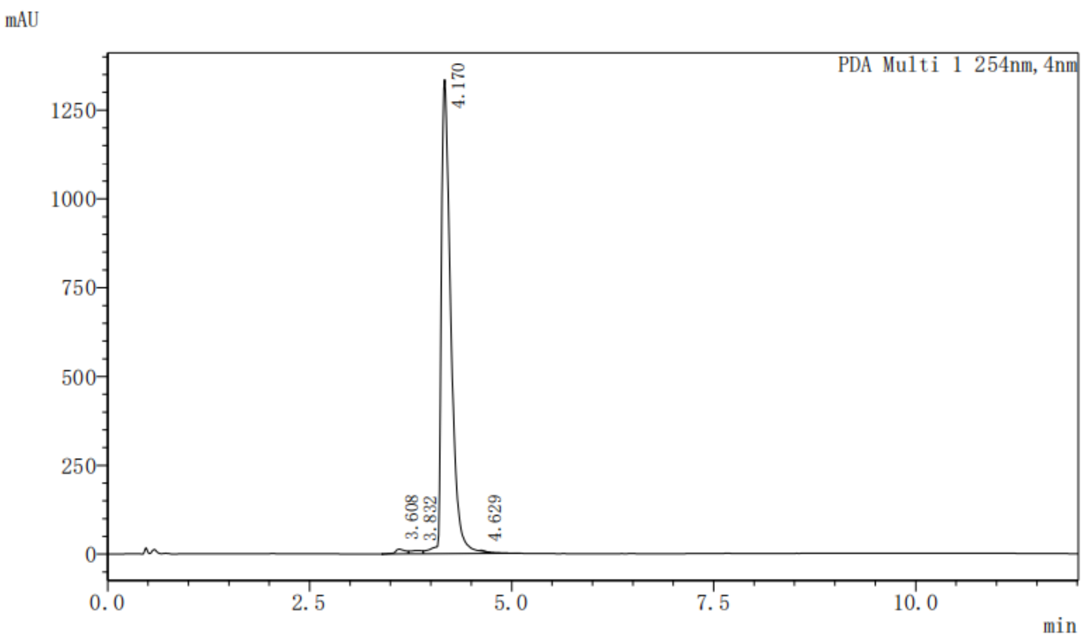

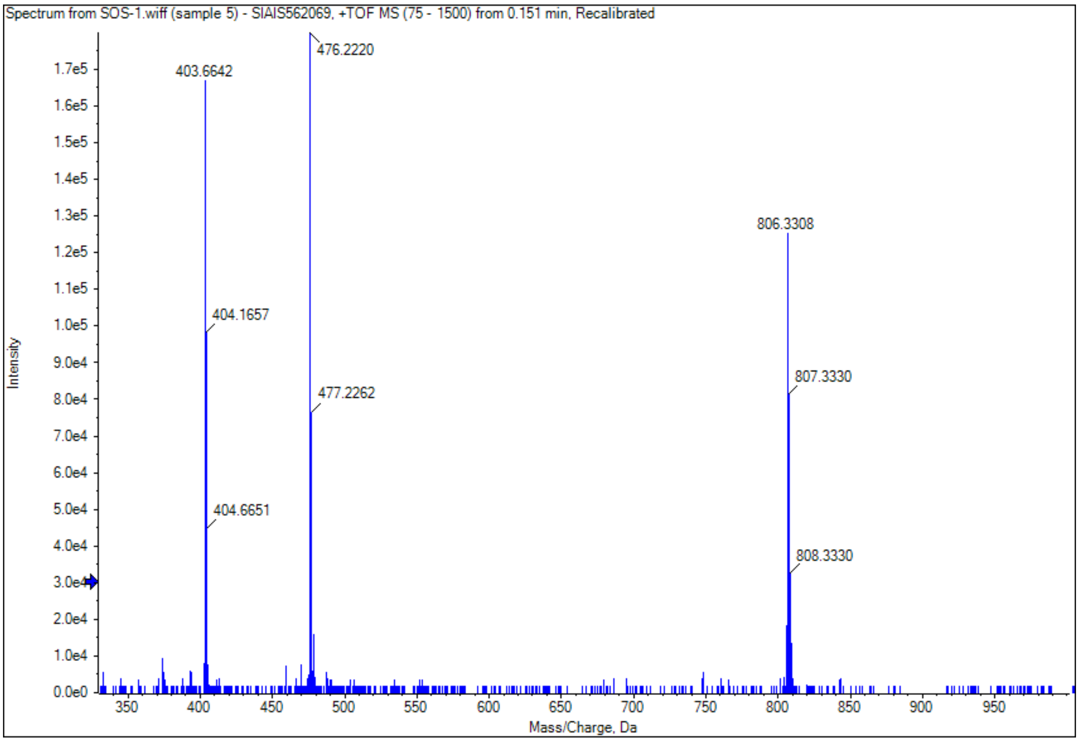

Figure S15. Purity spectrum and mass spectrum of compound **A3**.

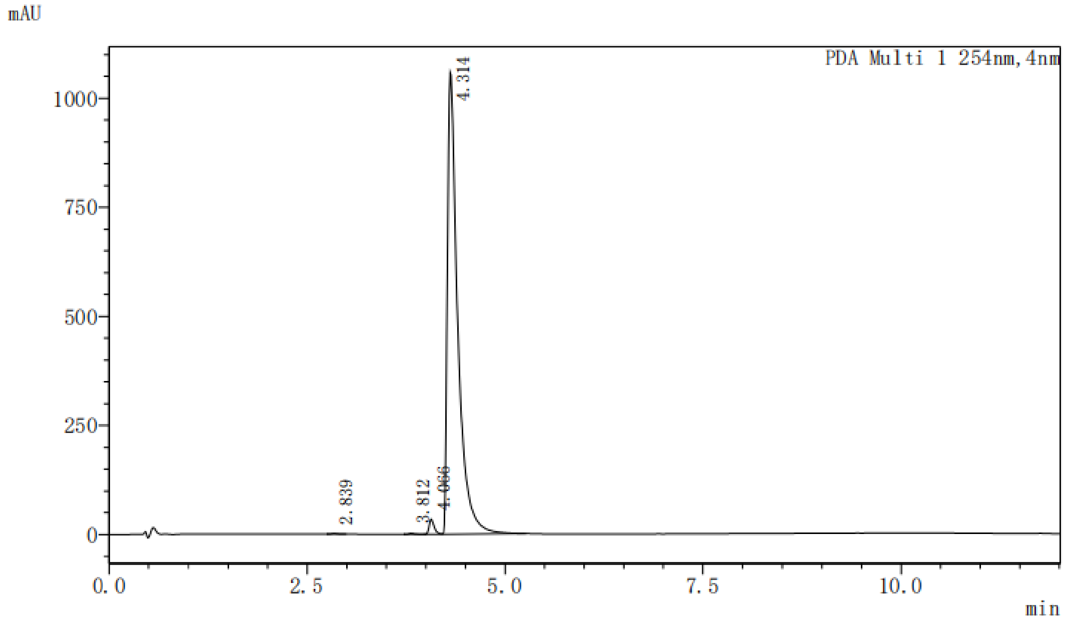

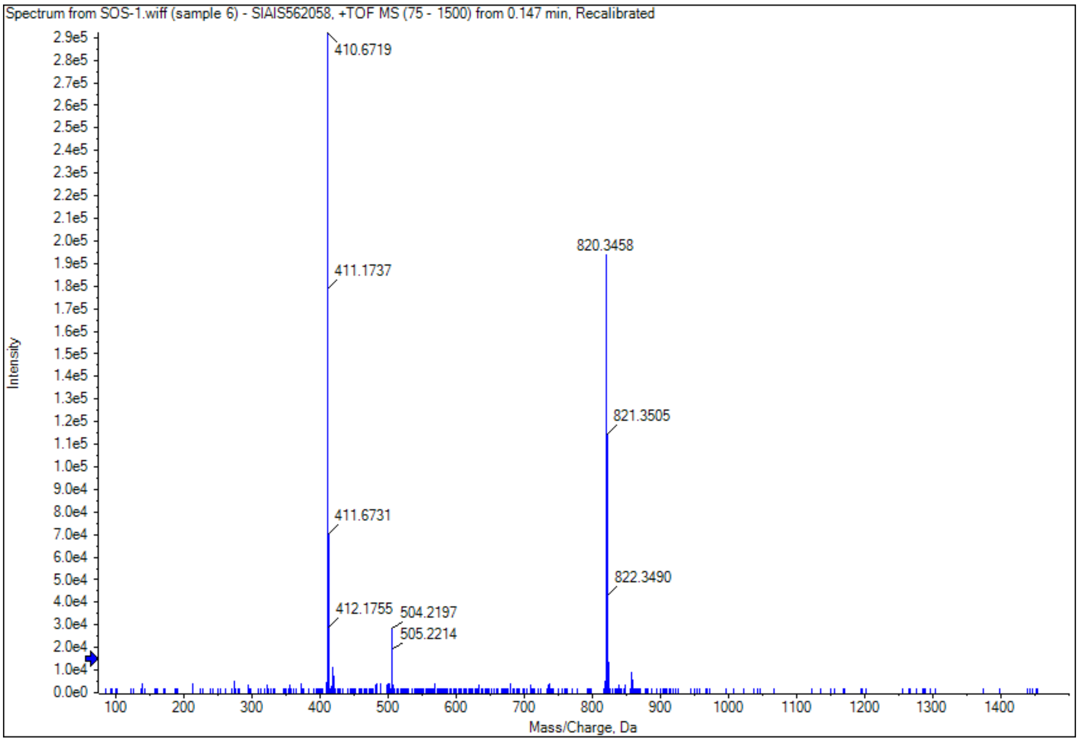

Figure S16. Purity spectrum and mass spectrum of compound **A4**.

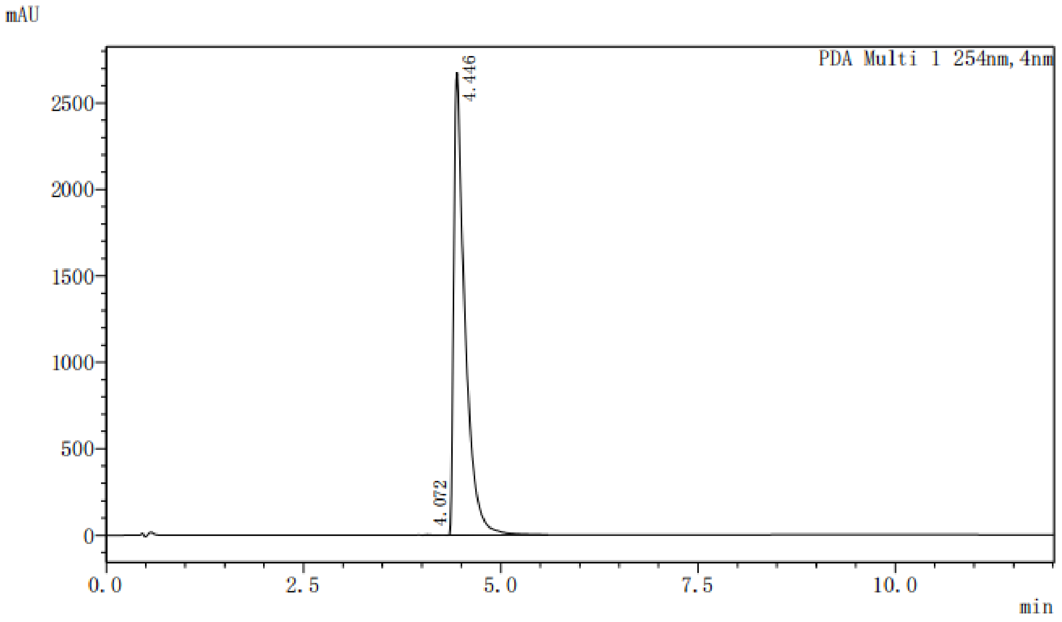

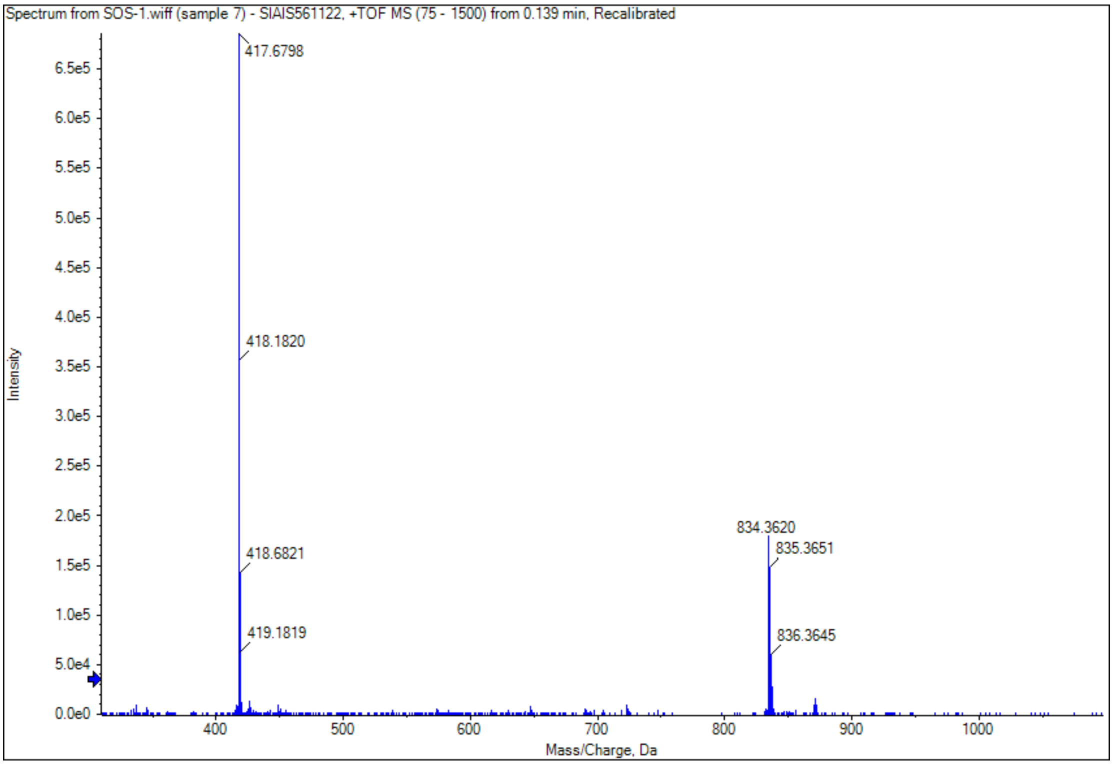

Figure S17. Purity spectrum and mass spectrum of compound **A5**.

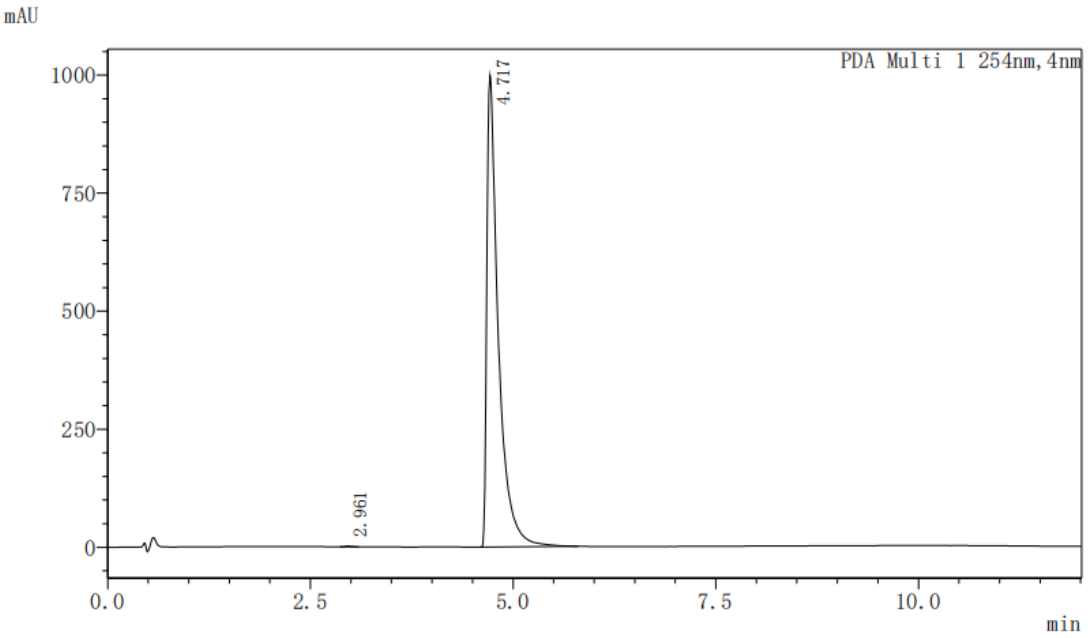

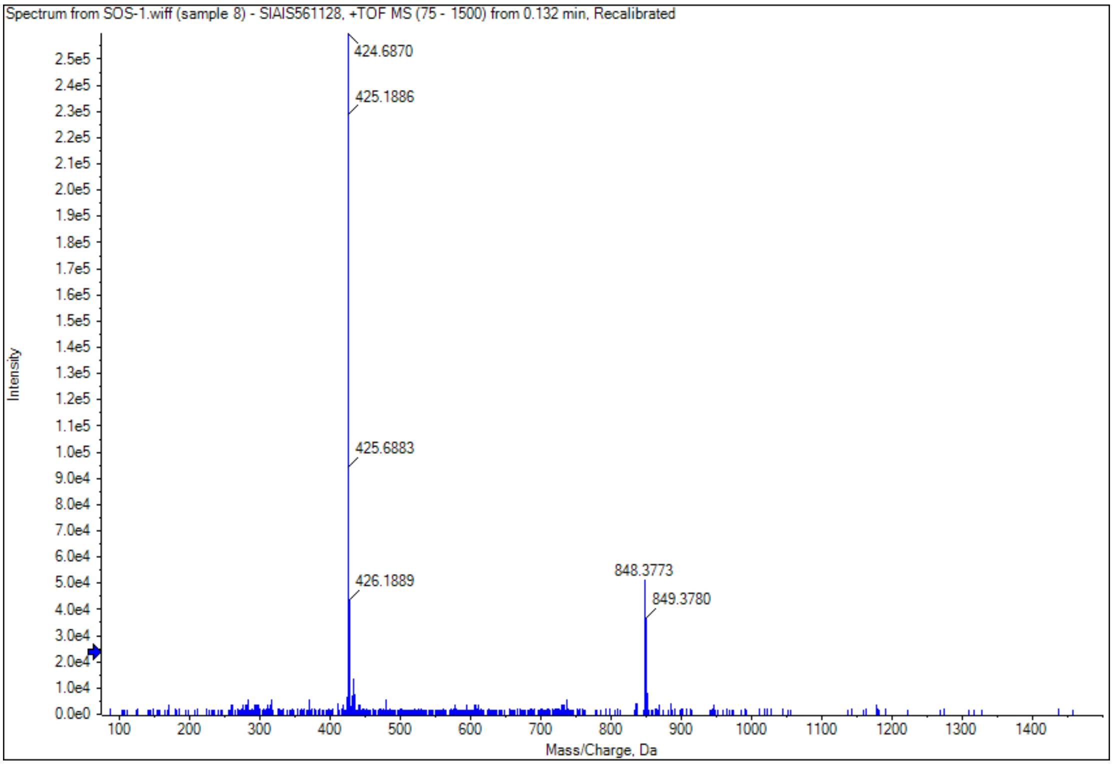

Figure S18. Purity spectrum and mass spectrum of compound **A6**.

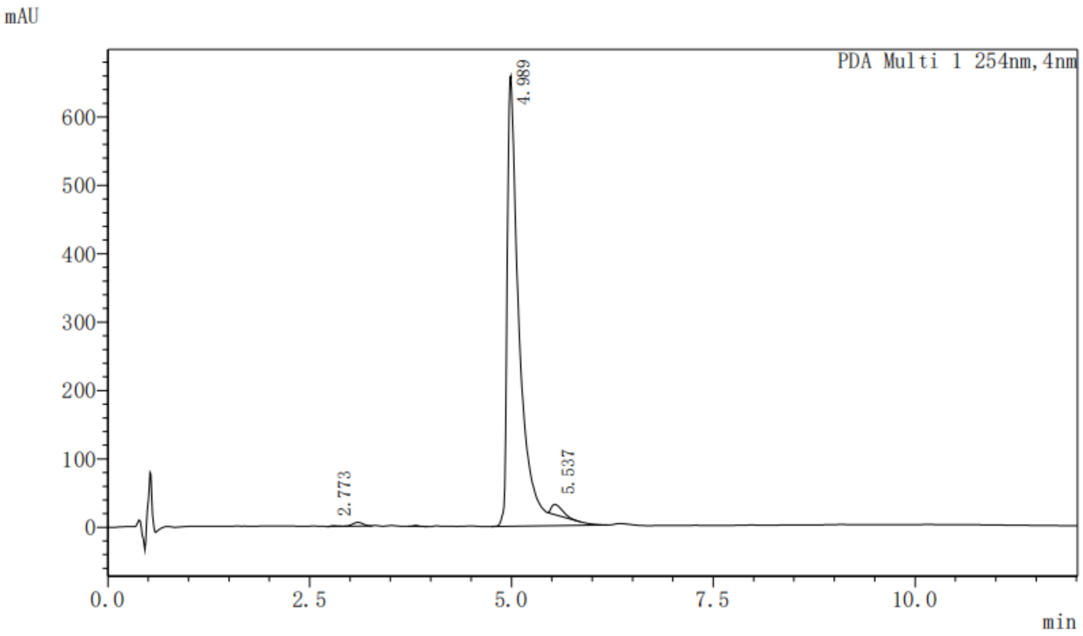

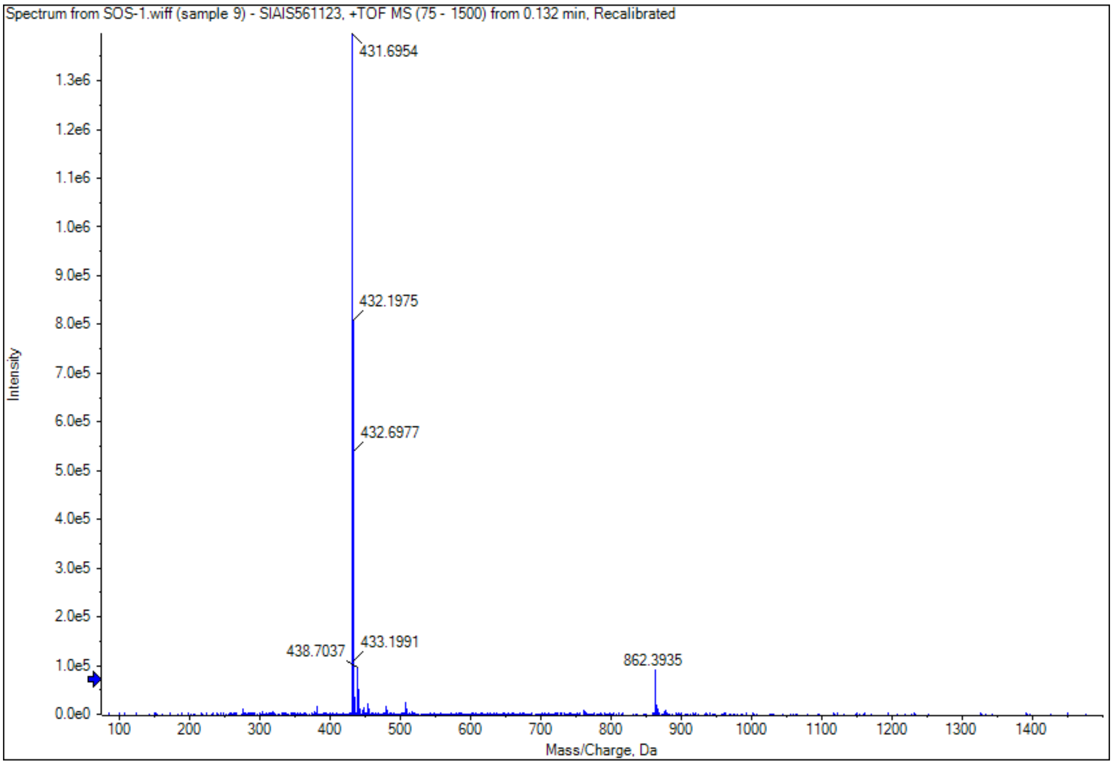

Figure S19. Purity spectrum and mass spectrum of compound **A7**.

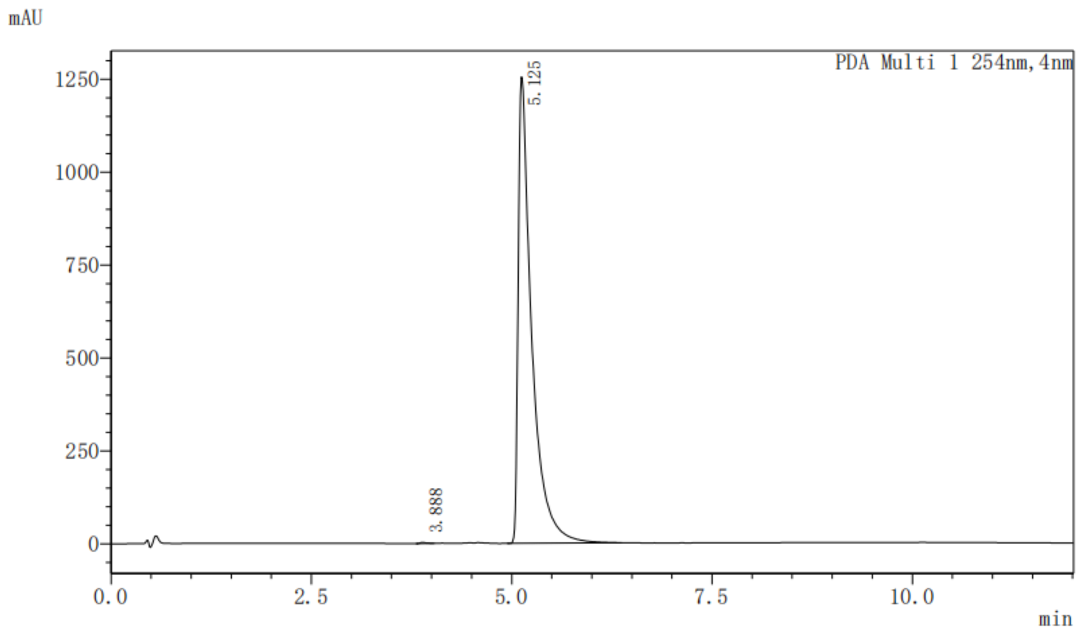

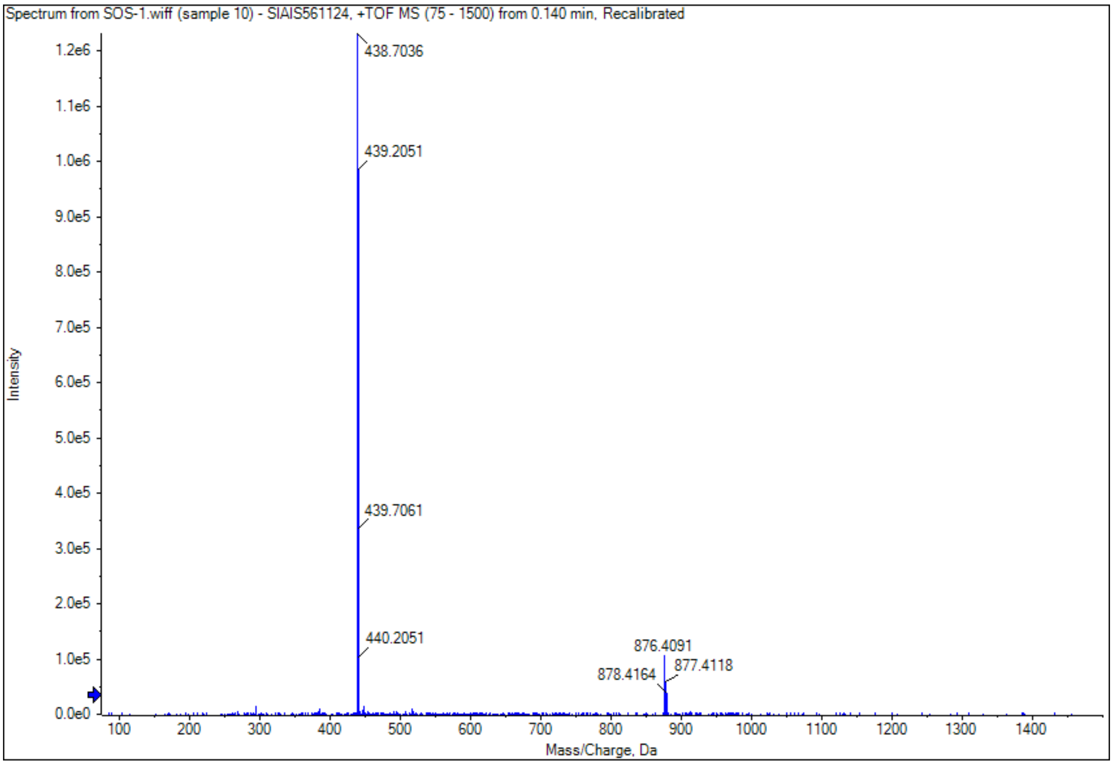

Figure S20. Purity spectrum and mass spectrum of compound **A8**.

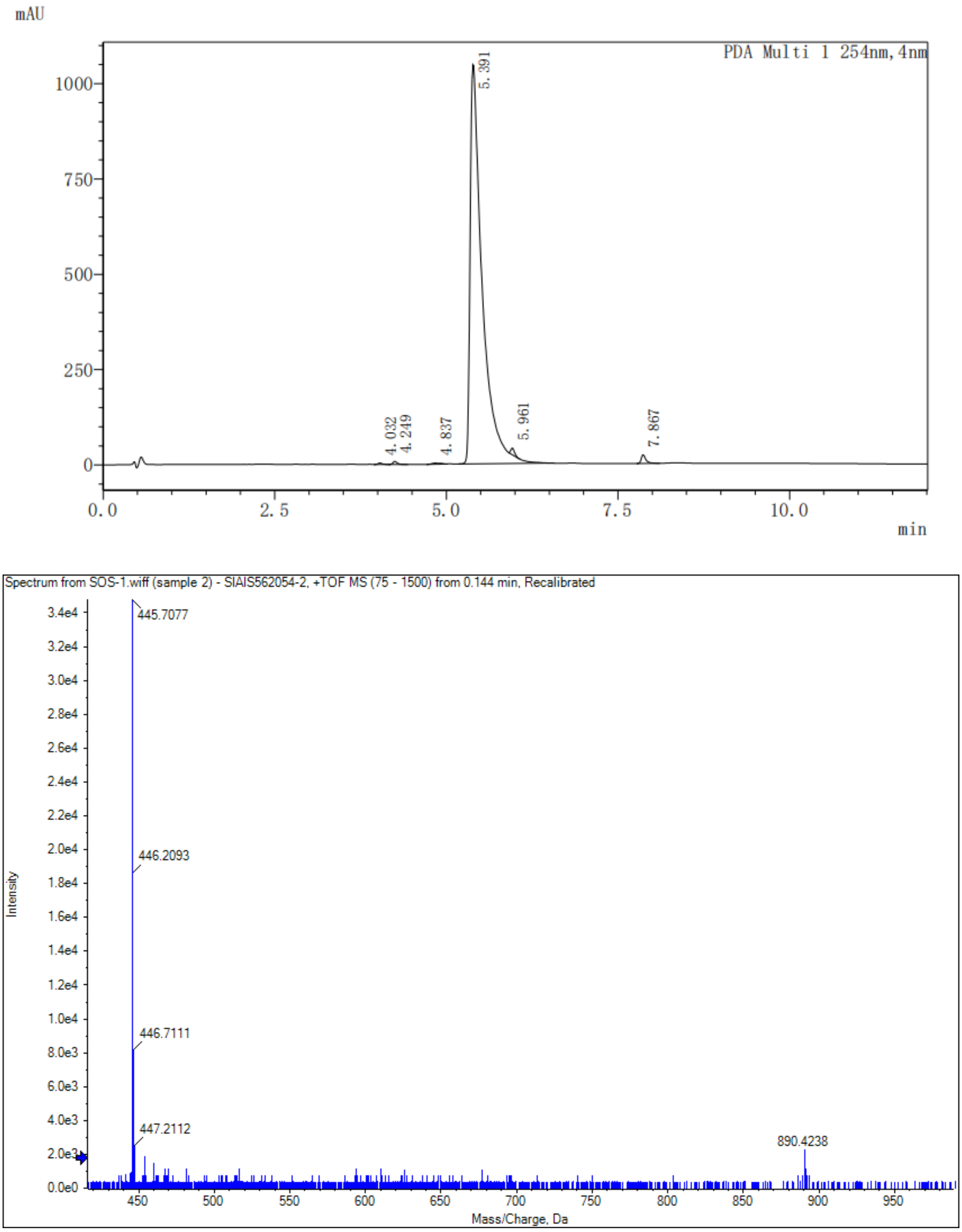

Figure S21. Purity spectrum and mass spectrum of compound **A9**.

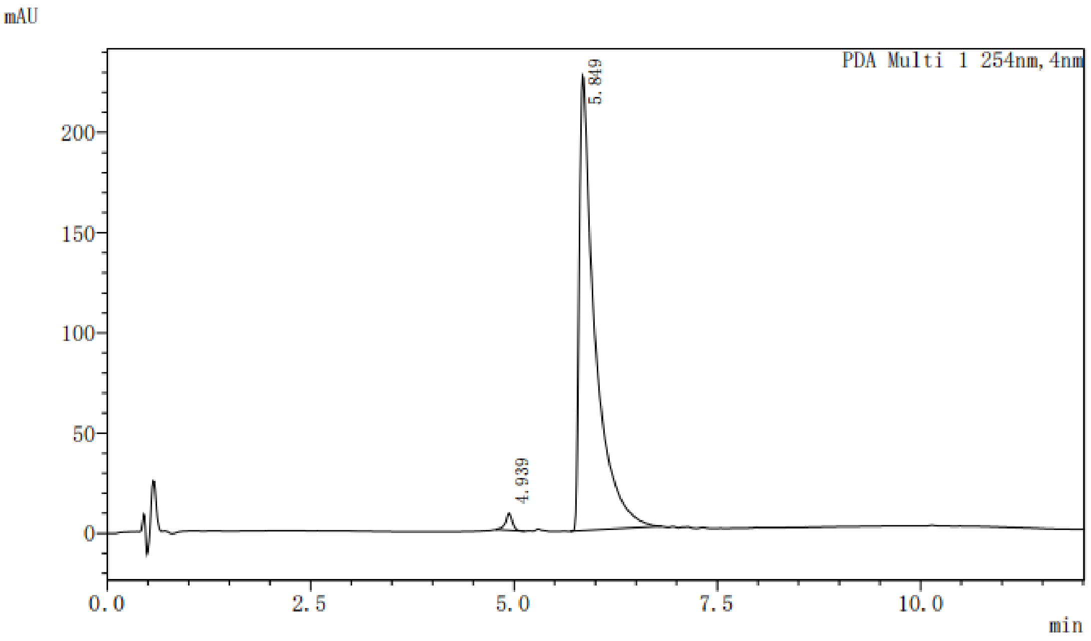

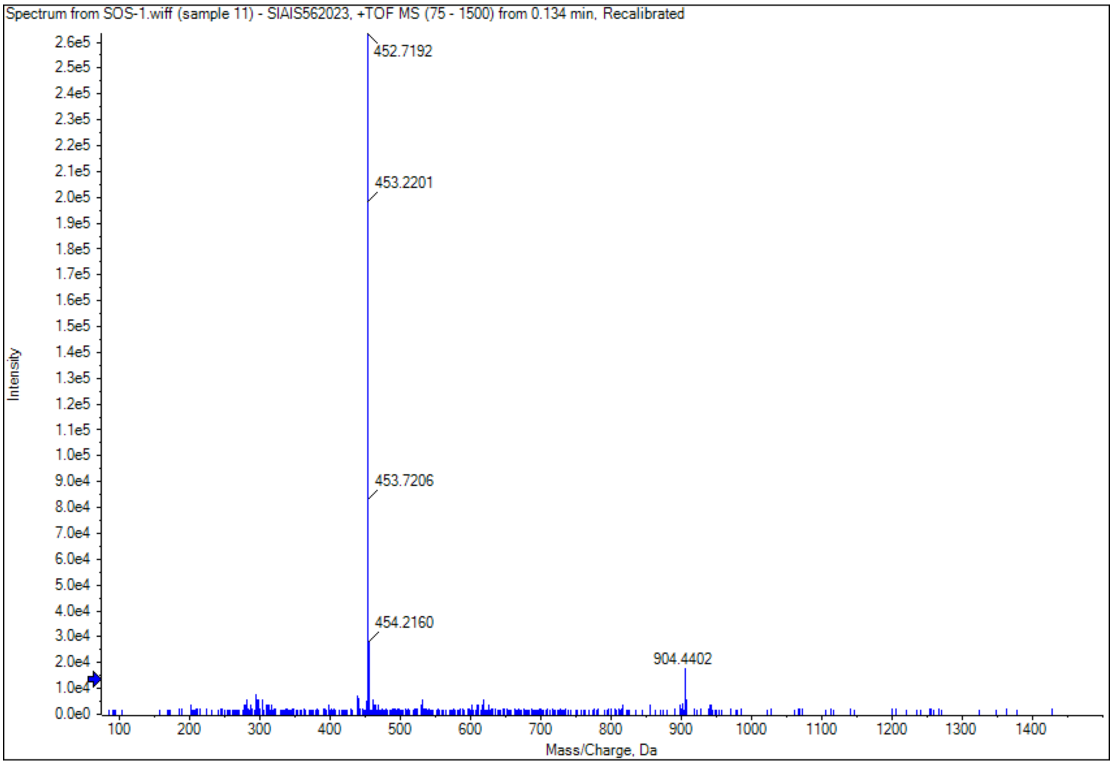

Figure S22. Purity spectrum and mass spectrum of compound **A10**.

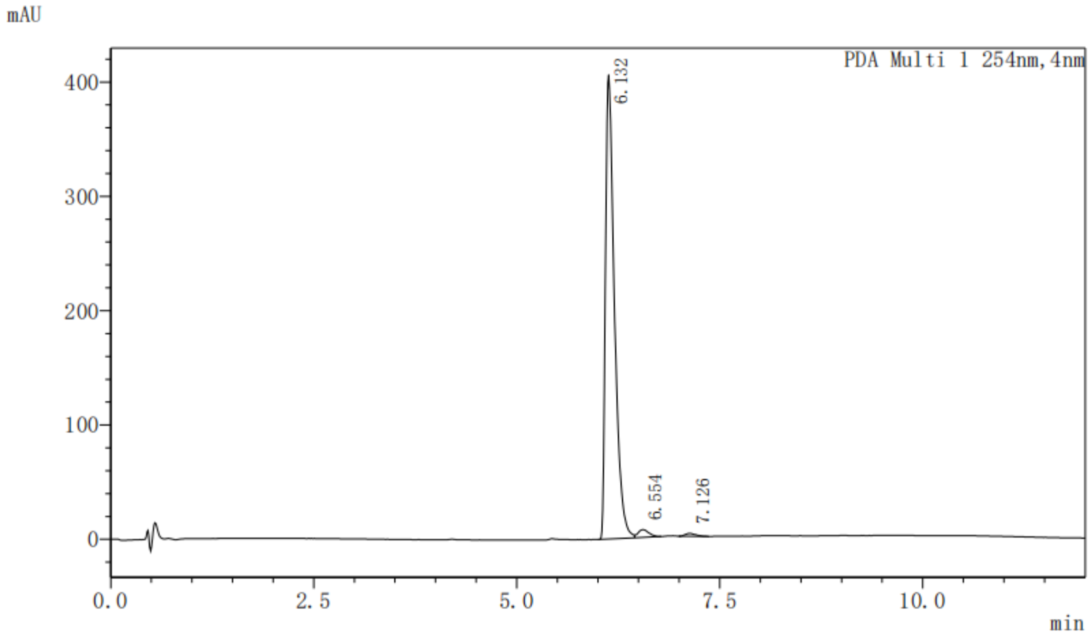

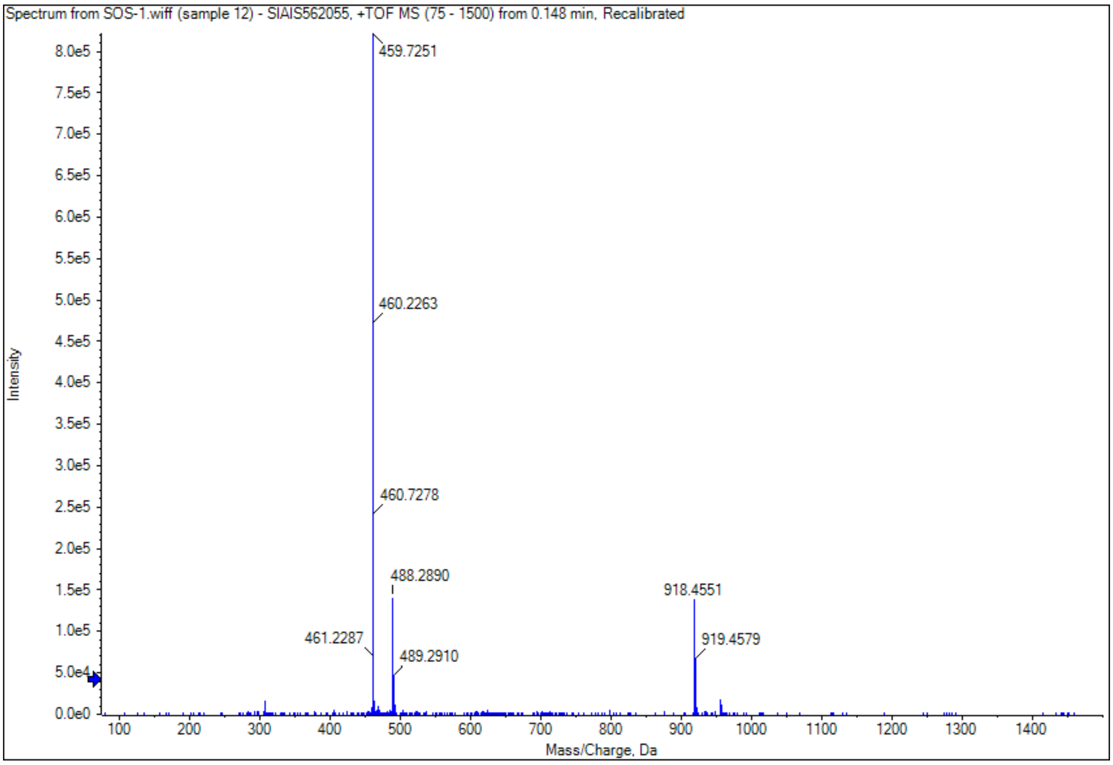

Figure S23. Purity spectrum and mass spectrum of compound **A11**.

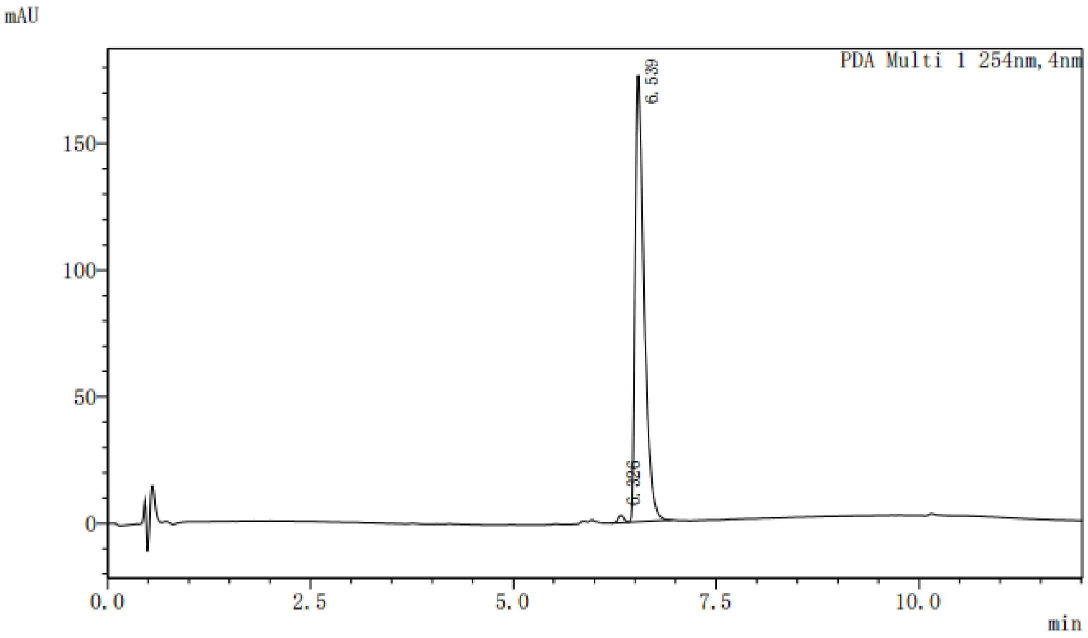

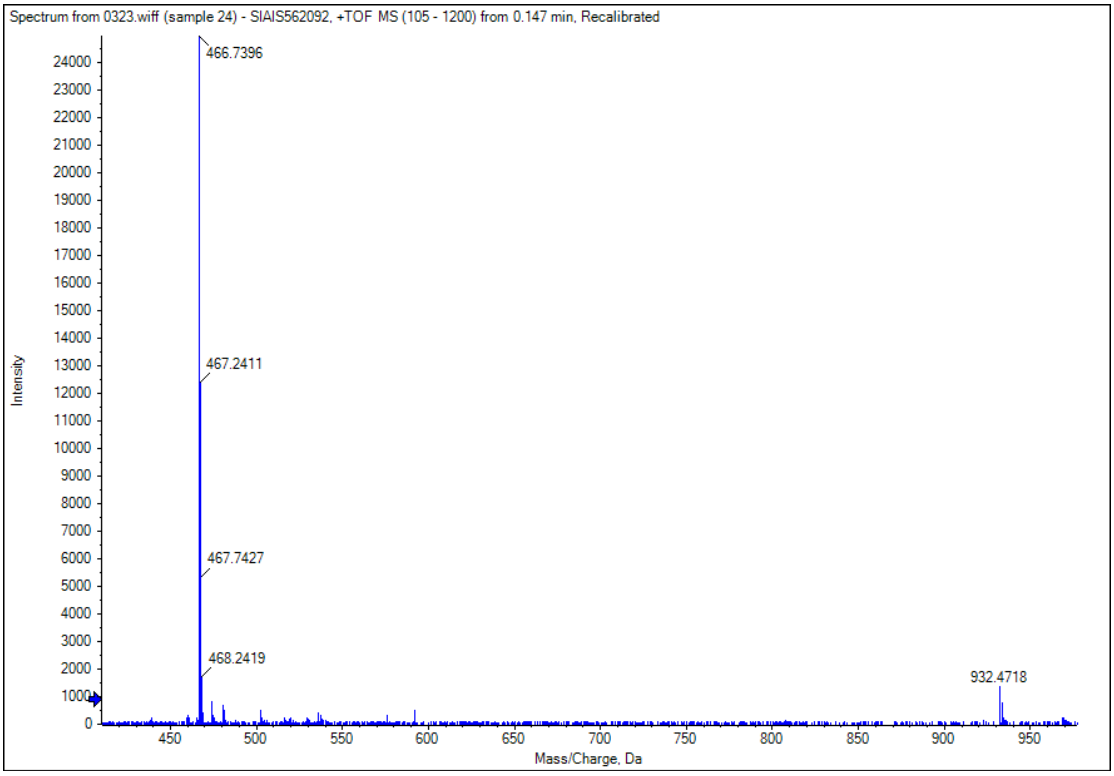

Figure S24. Purity spectrum and mass spectrum of compound **A11-NC**.

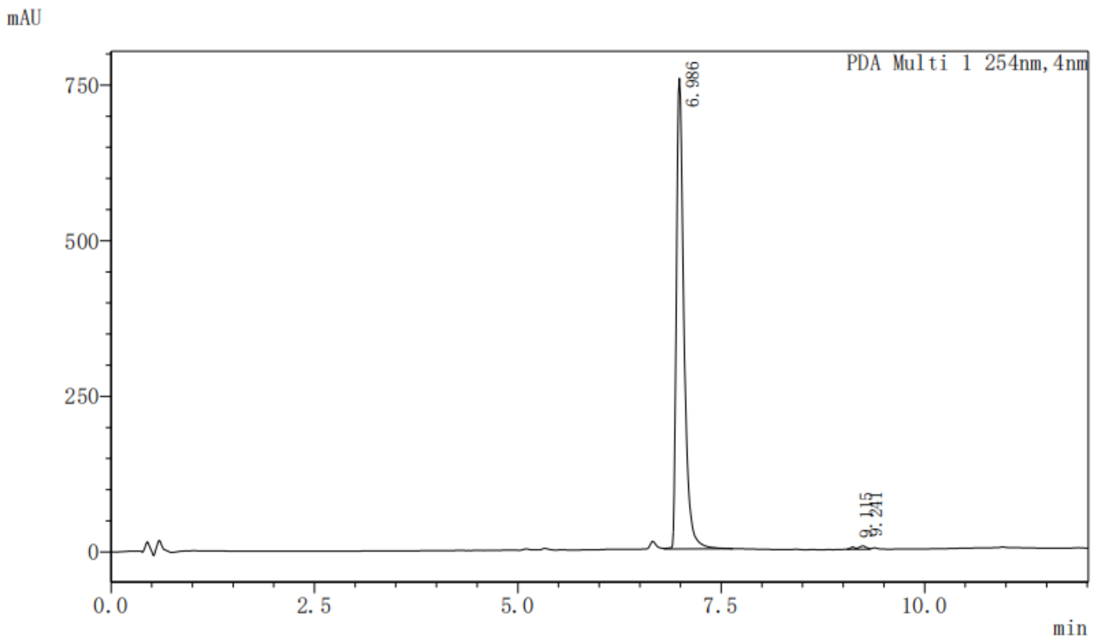

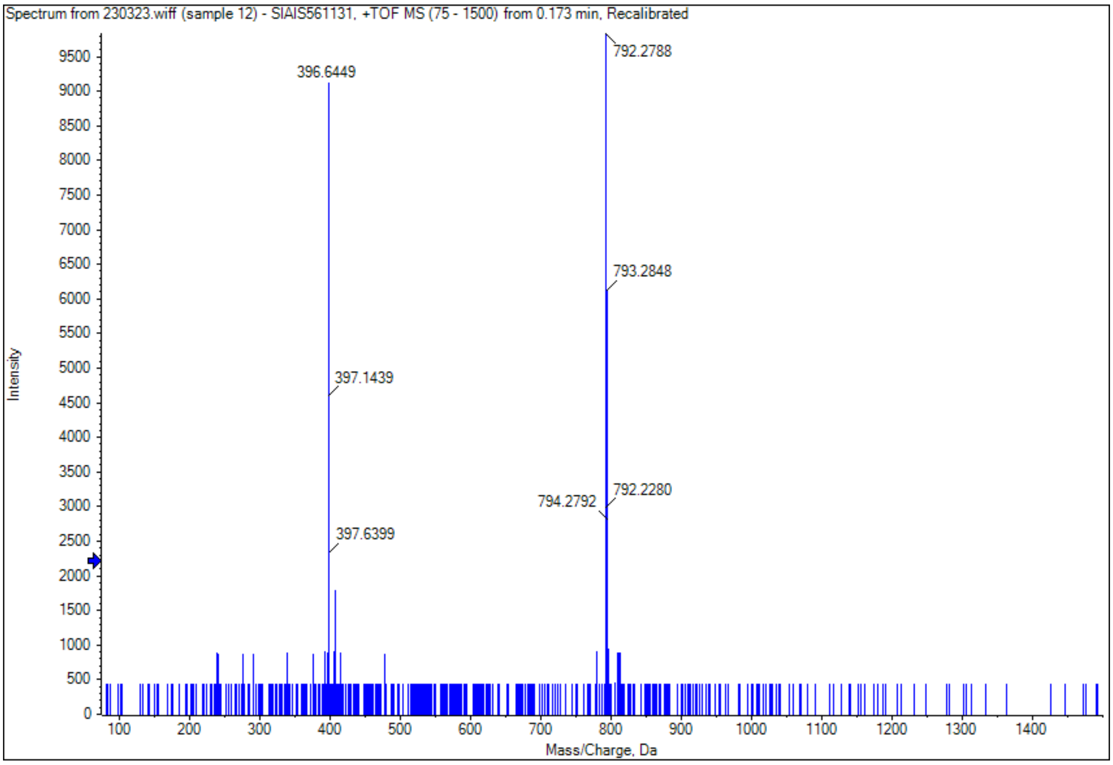

Figure S25. Purity spectrum and mass spectrum of compound **B1.**

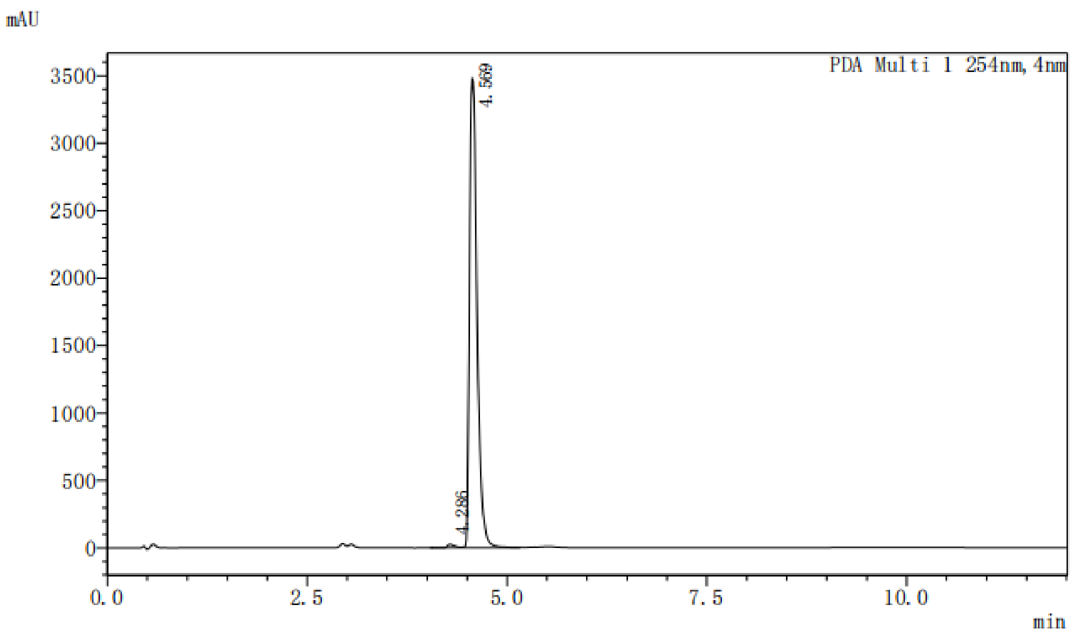

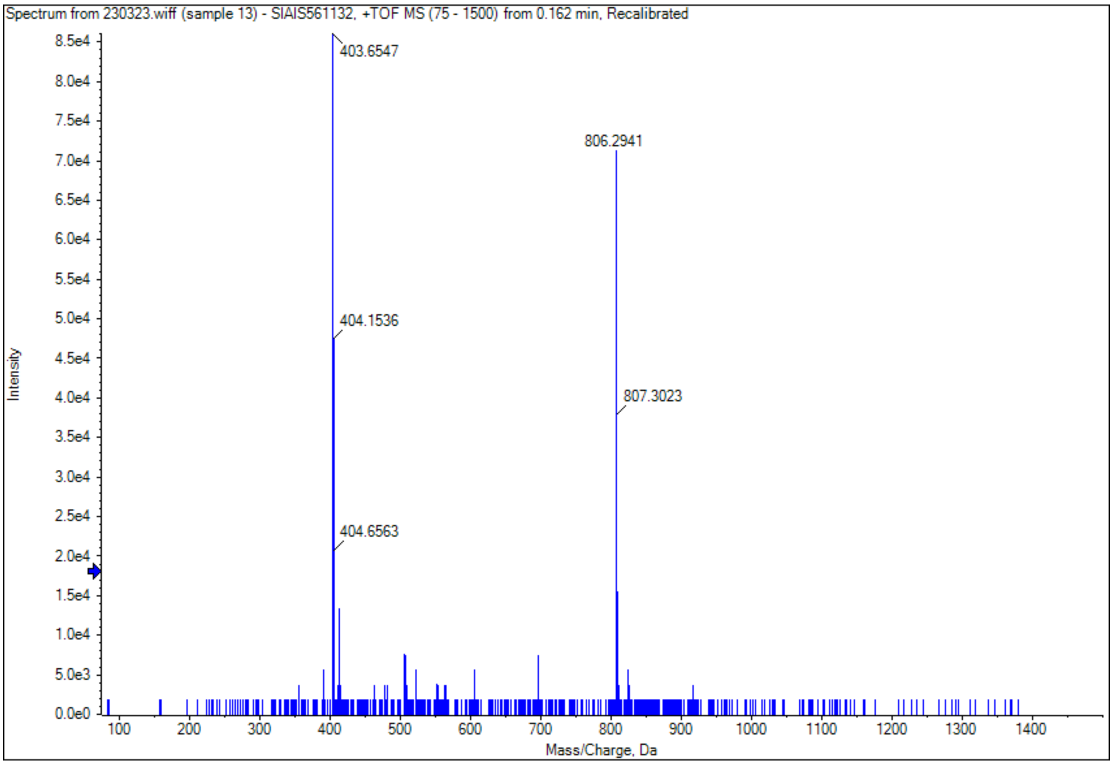

Figure S26. Purity spectrum and mass spectrum of compound **B2.**

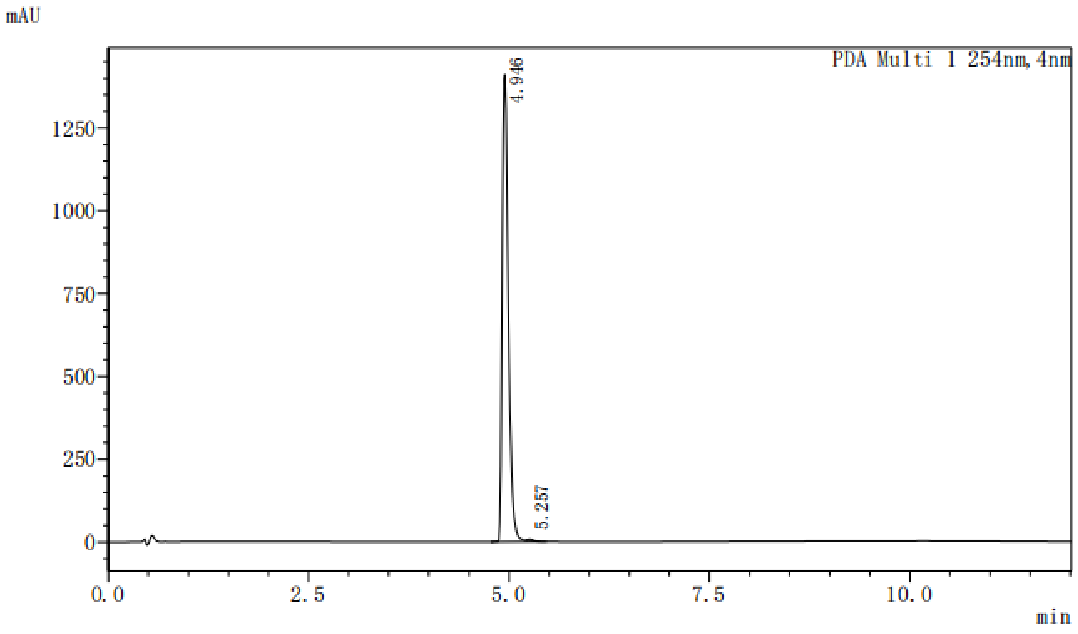

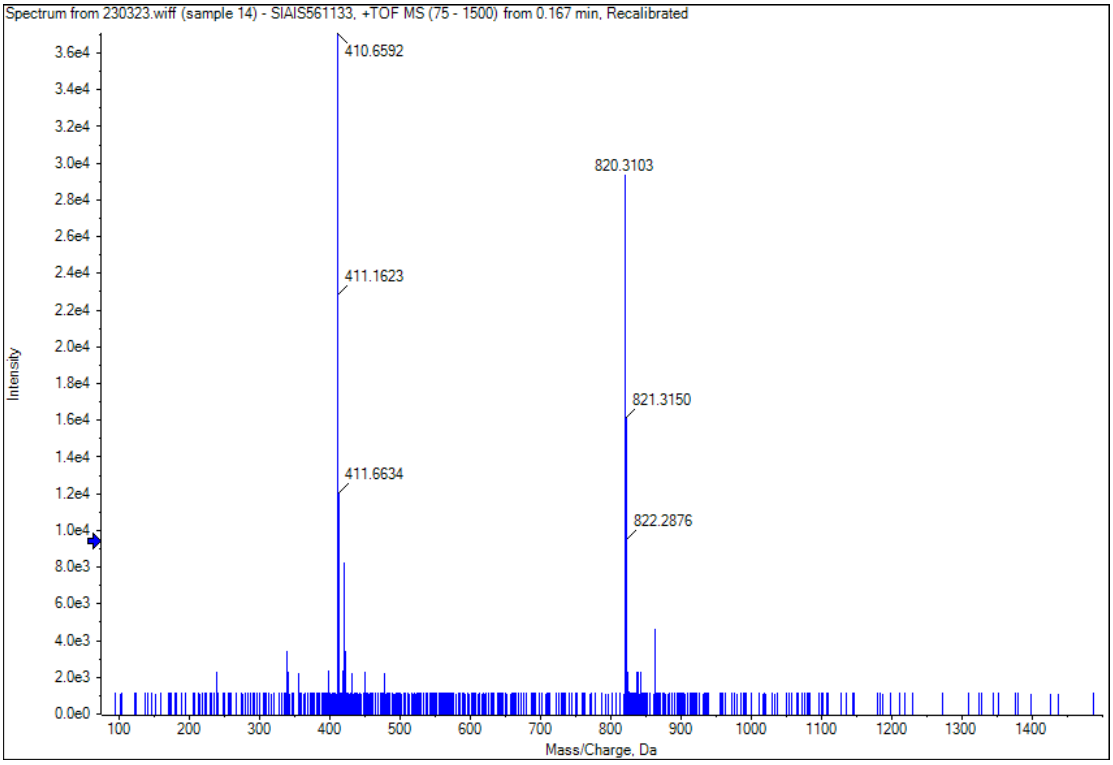

Figure S27. Purity spectrum and mass spectrum of compound **B3.**

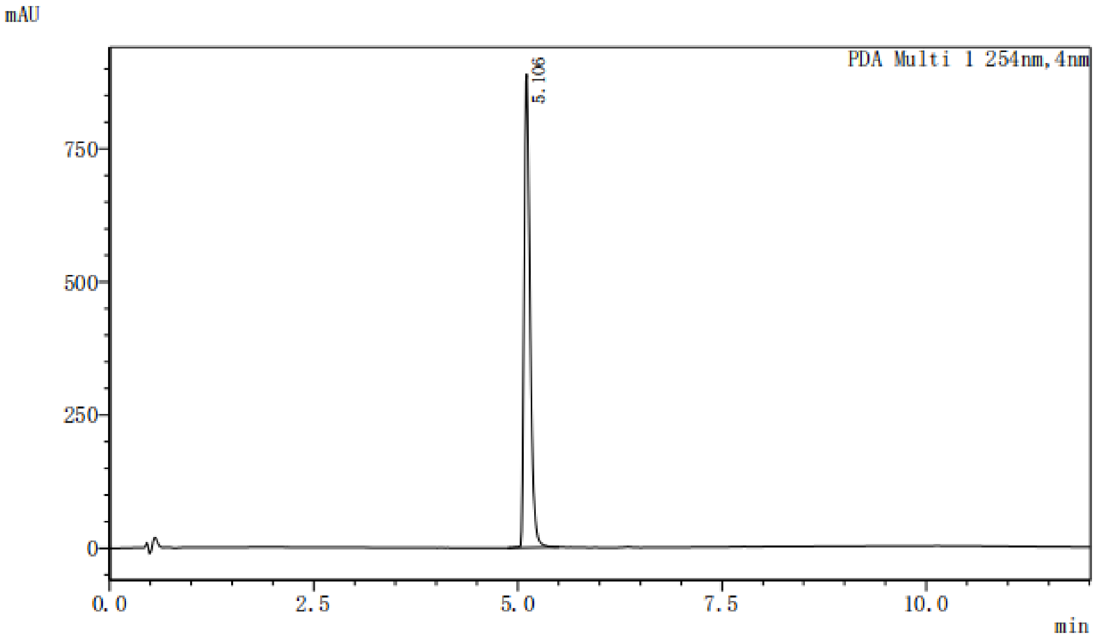

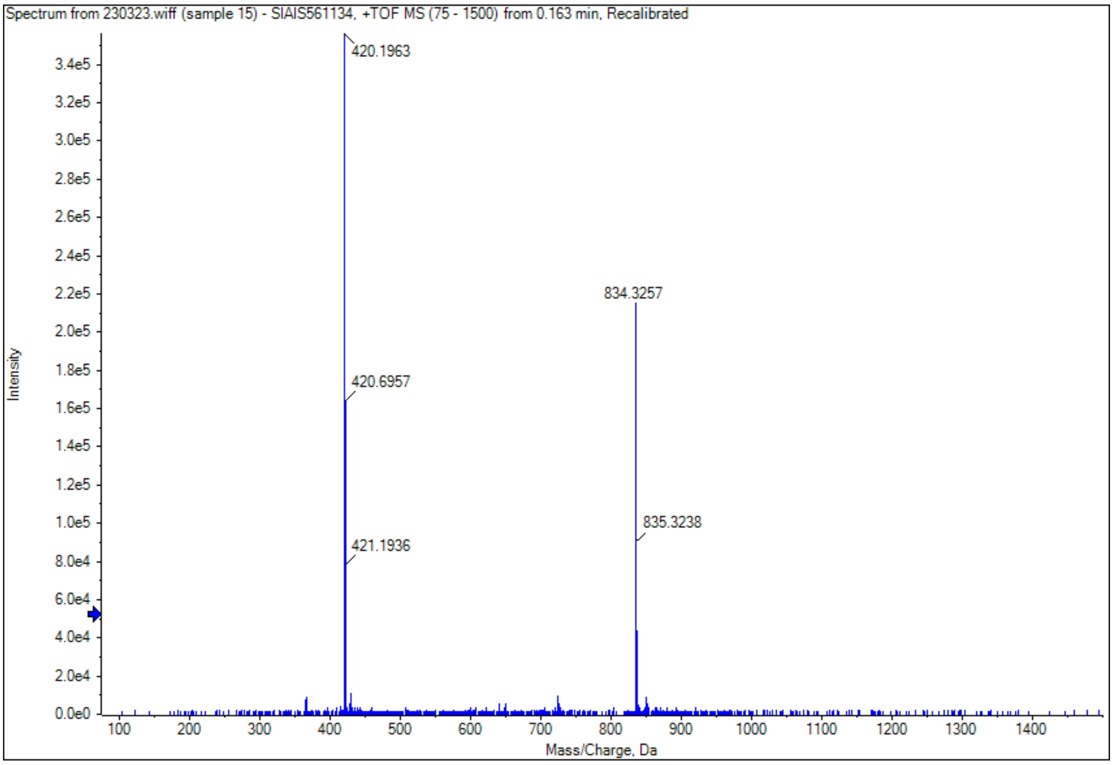

Figure S28. Purity spectrum and mass spectrum of compound **B4.**

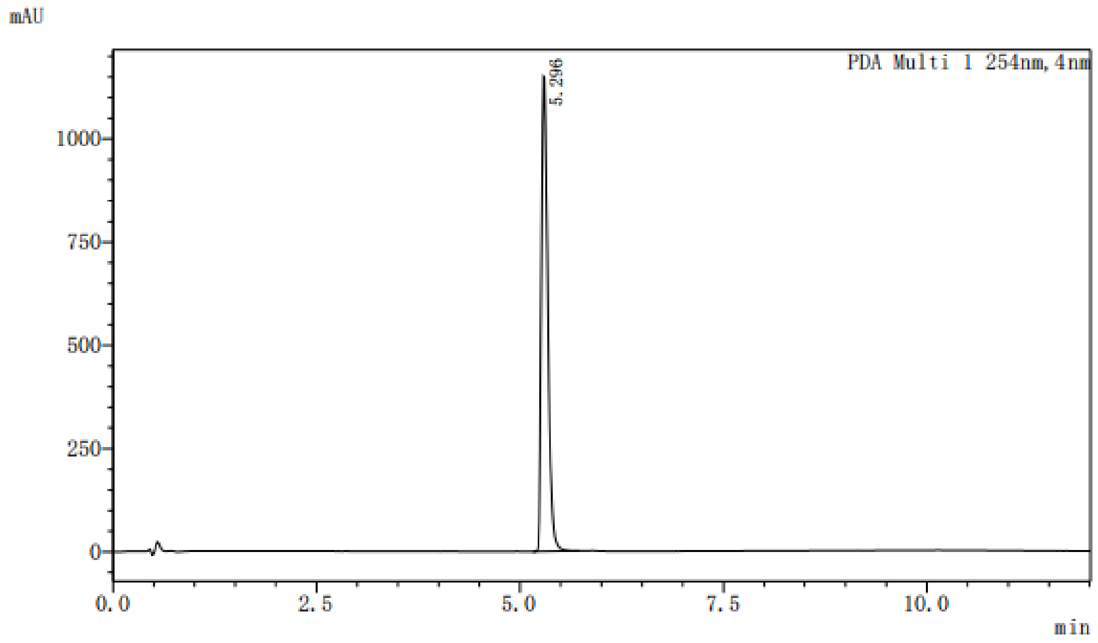

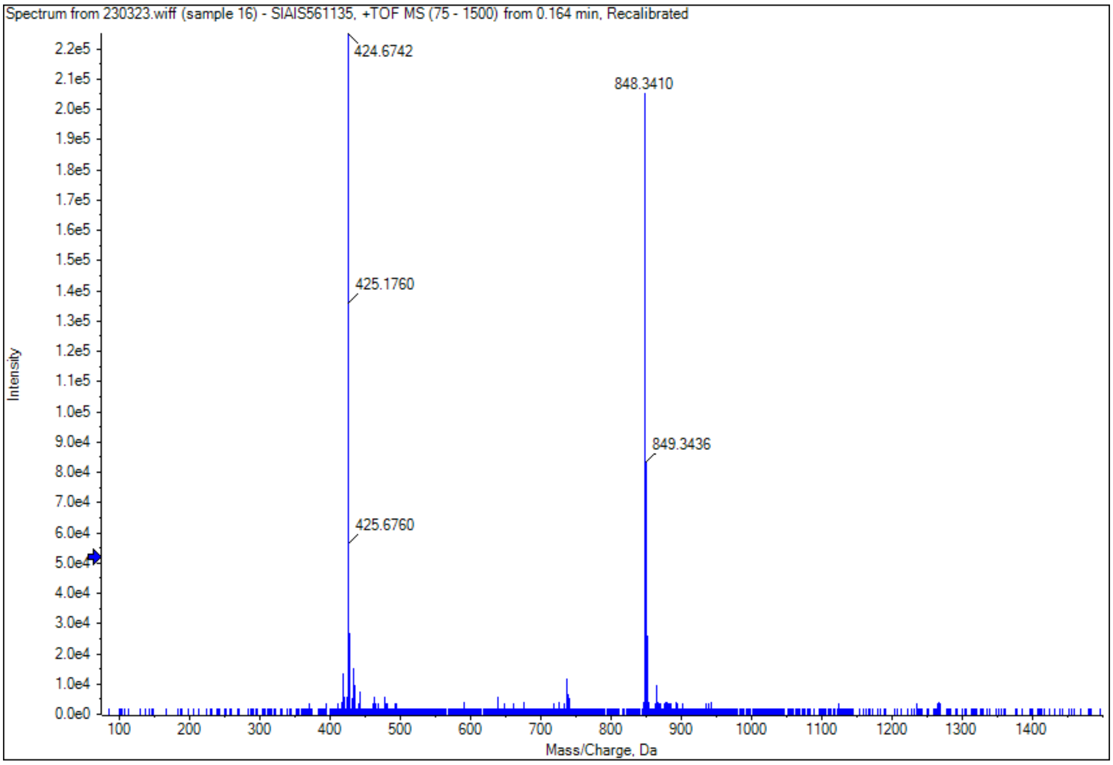

Figure S29. Purity spectrum and mass spectrum of compound **B5.**

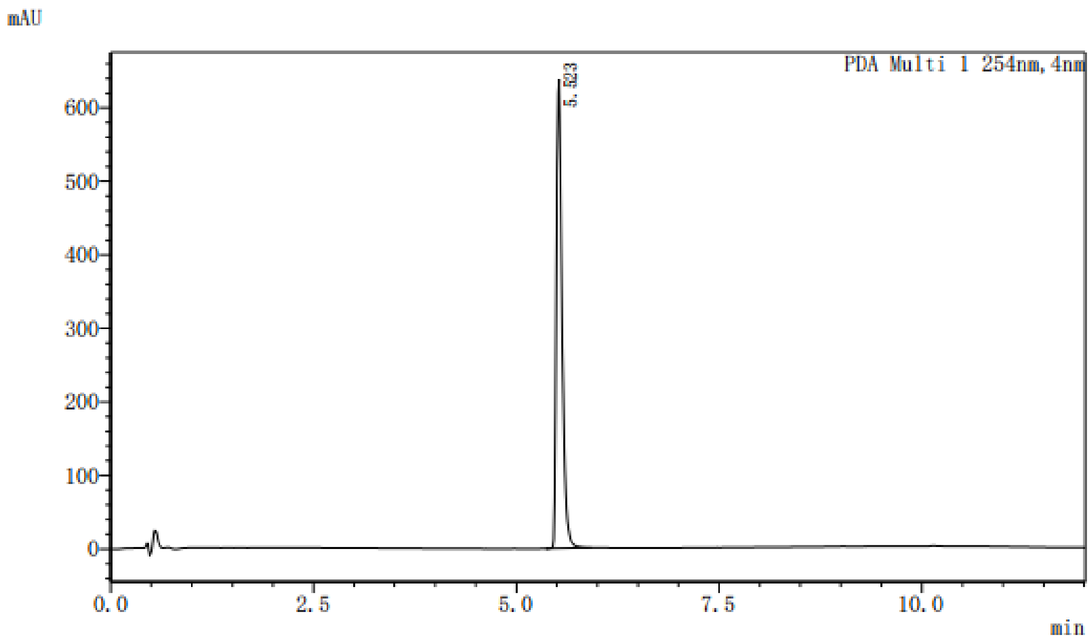

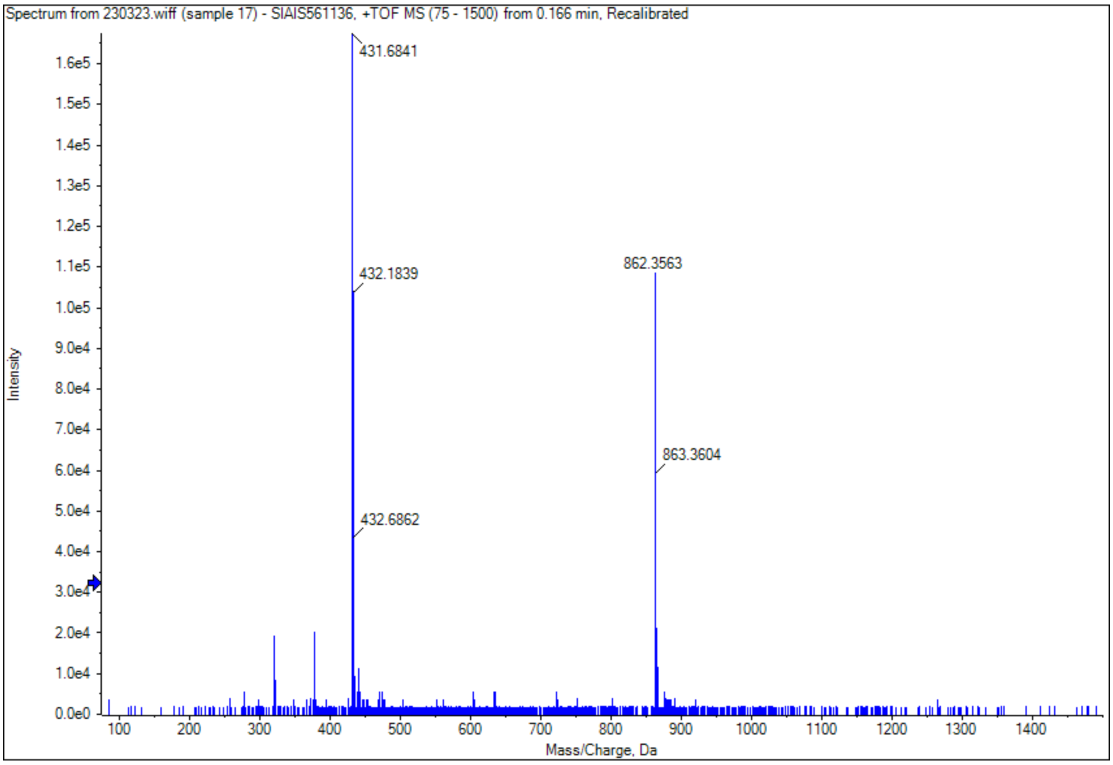

Figure S30. Purity spectrum and mass spectrum of compound **B6.**

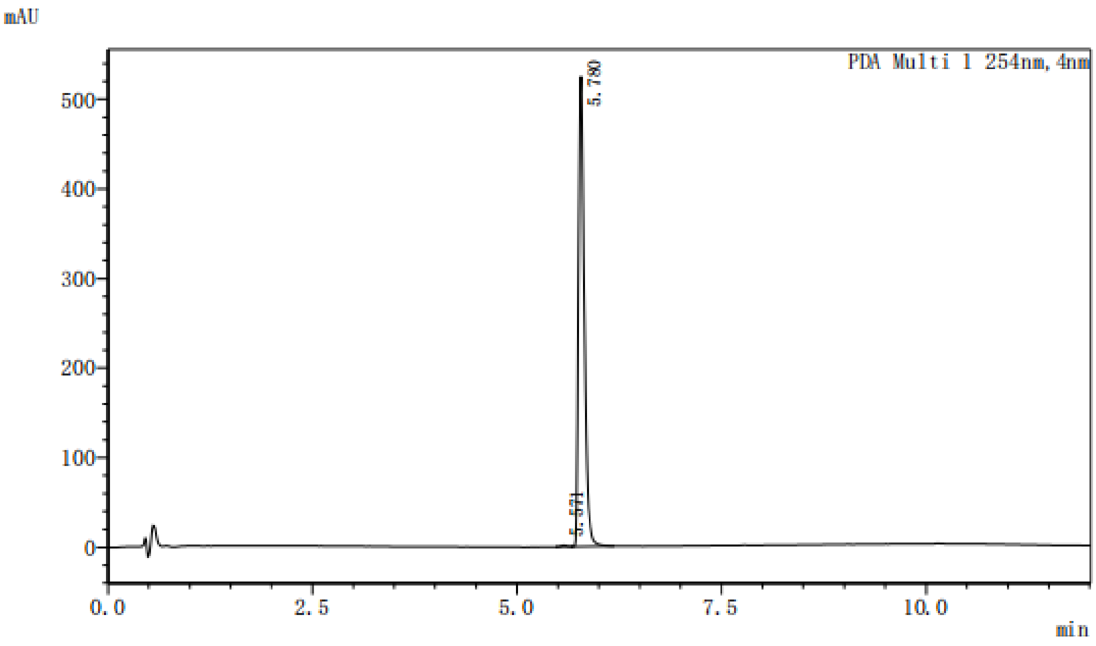

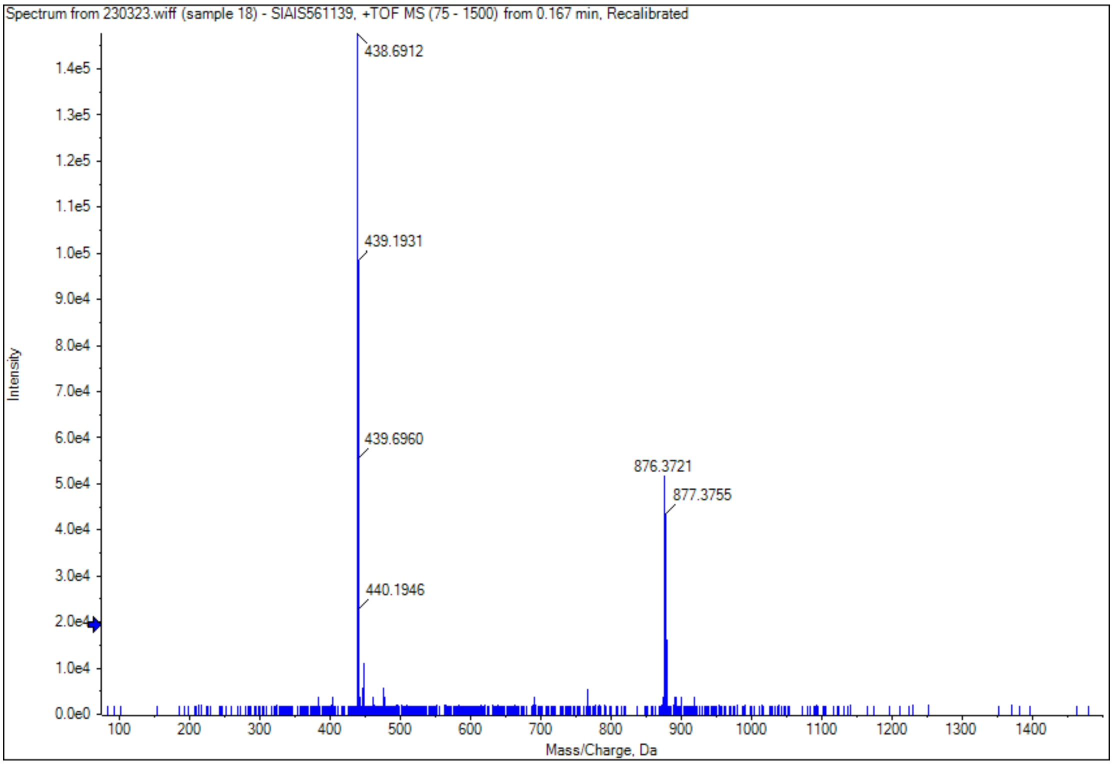

Figure S31. Purity spectrum and mass spectrum of compound **B7.**

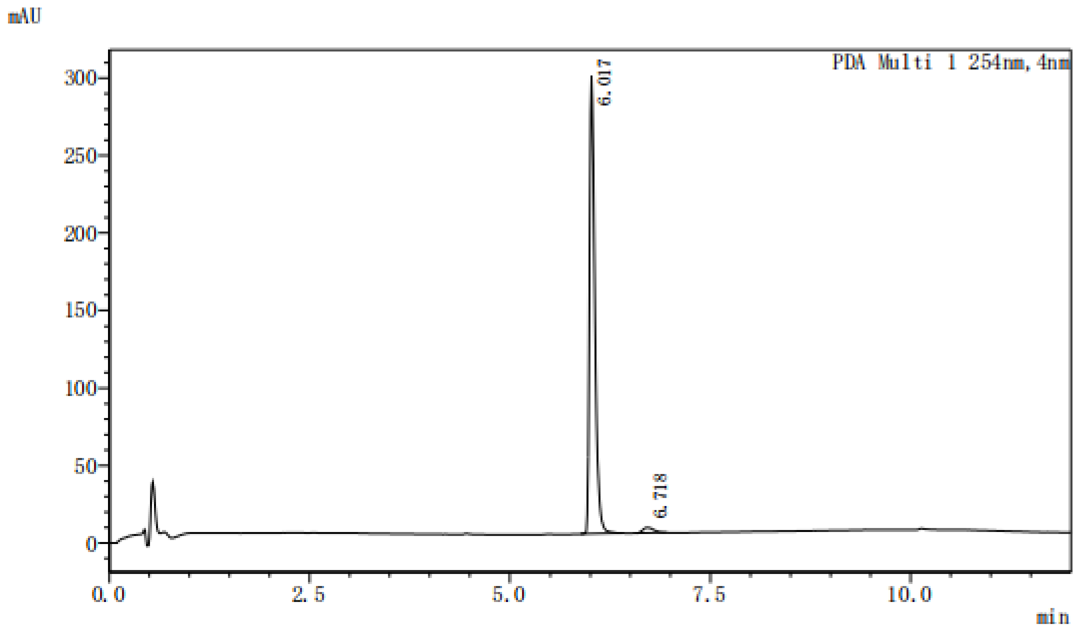

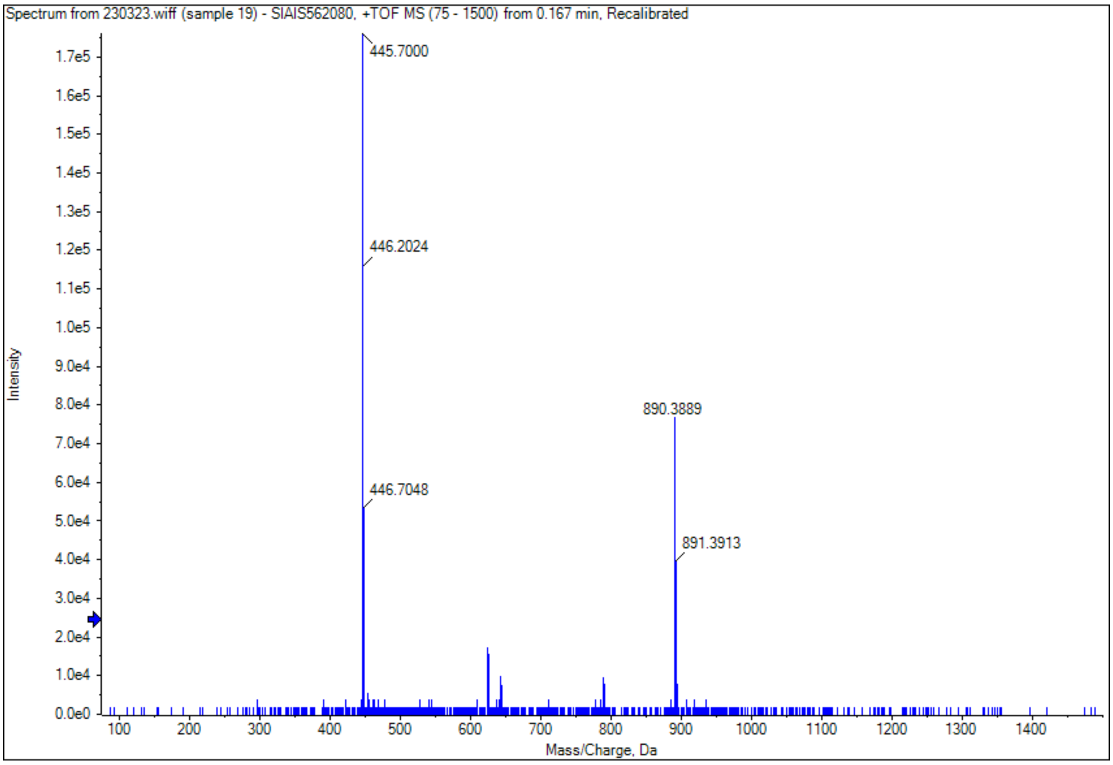

Figure S32. Purity spectrum and mass spectrum of compound **B8.**

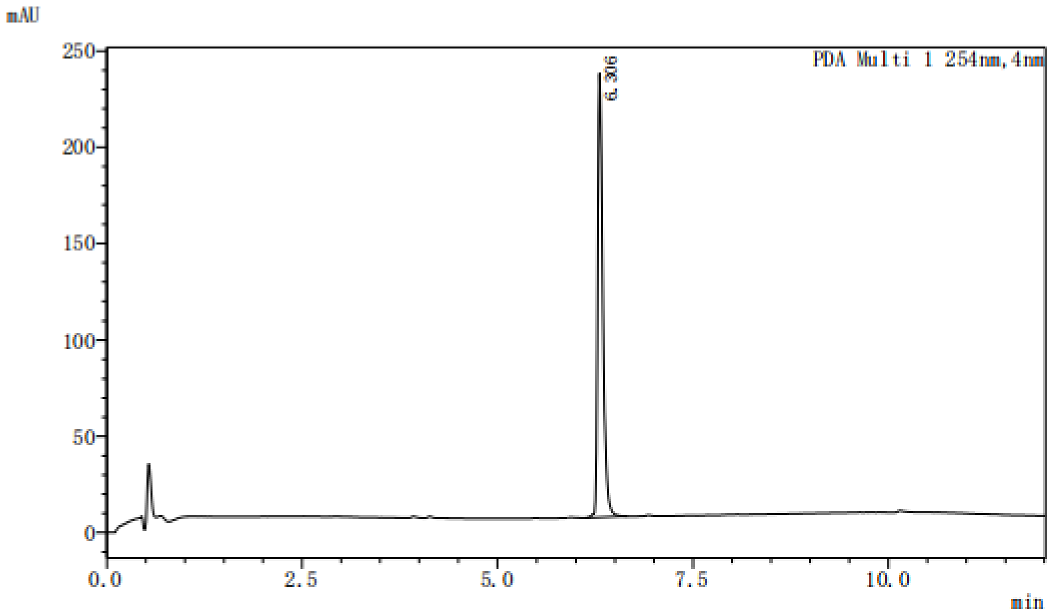

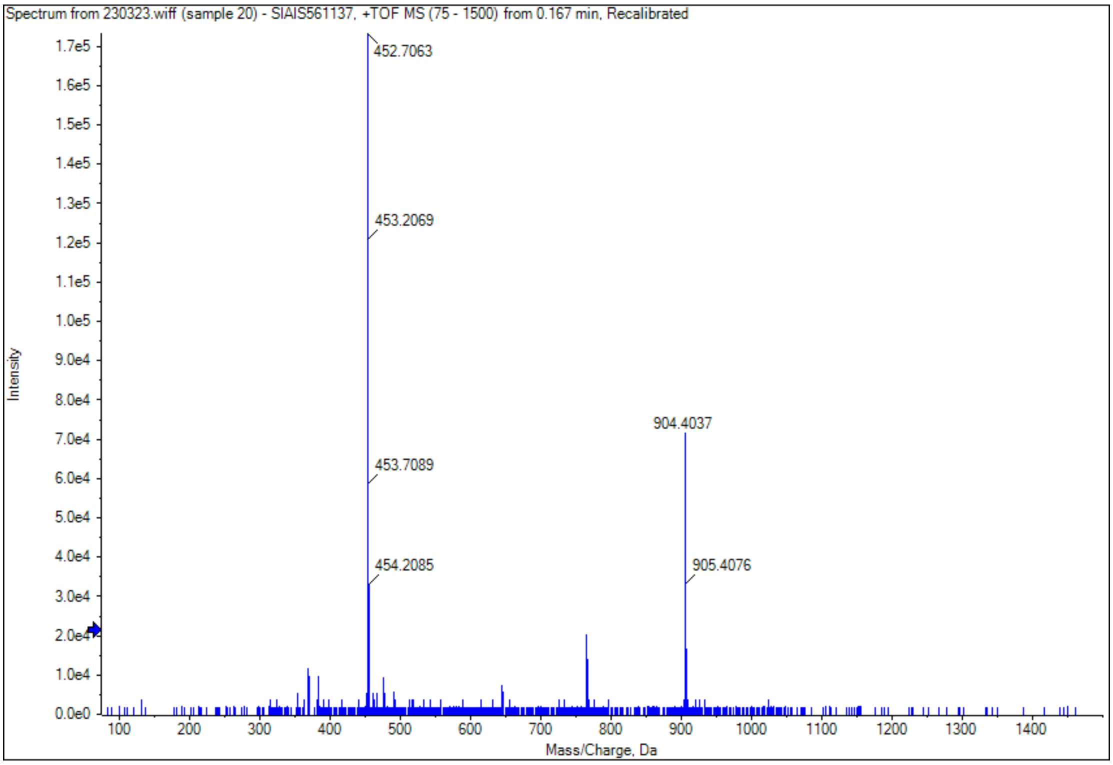

Figure S33. Purity spectrum and mass spectrum of compound **B9.**

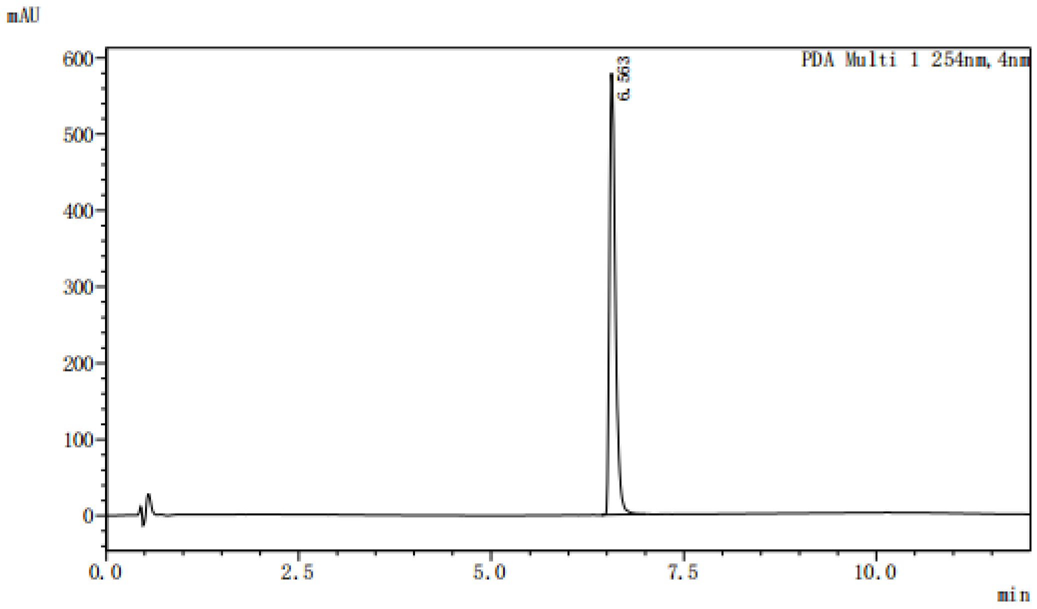

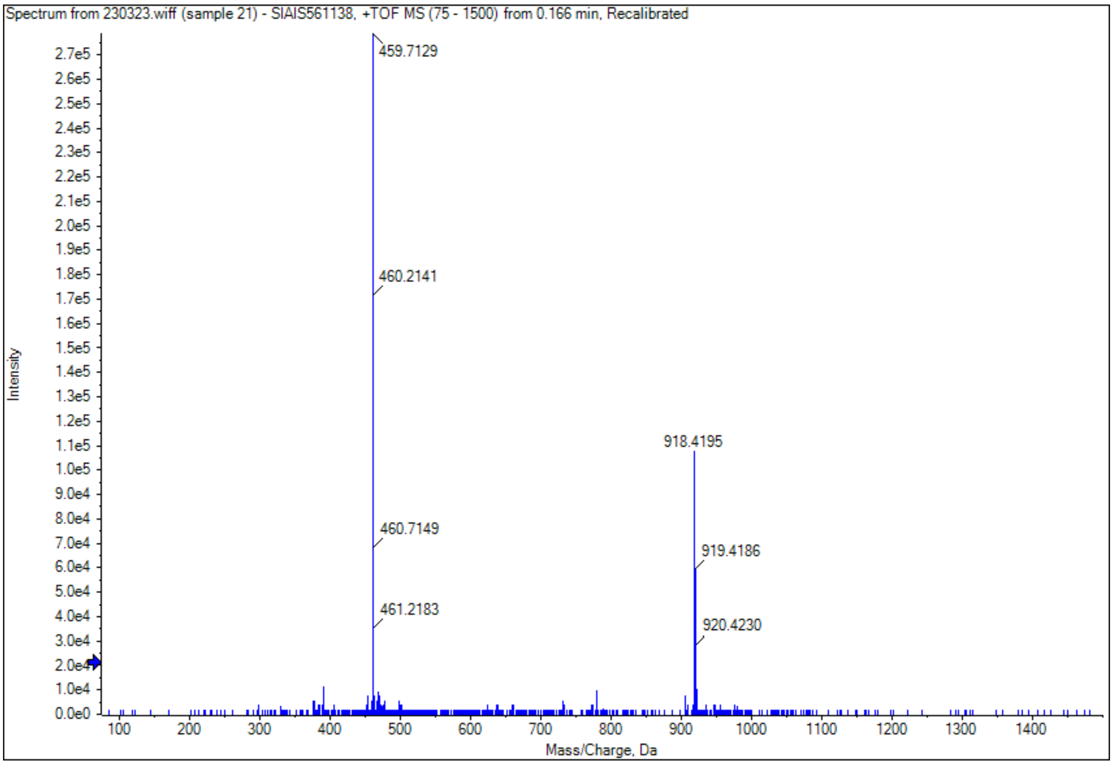

Figure S34. Purity spectrum and mass spectrum of compound **B10.**

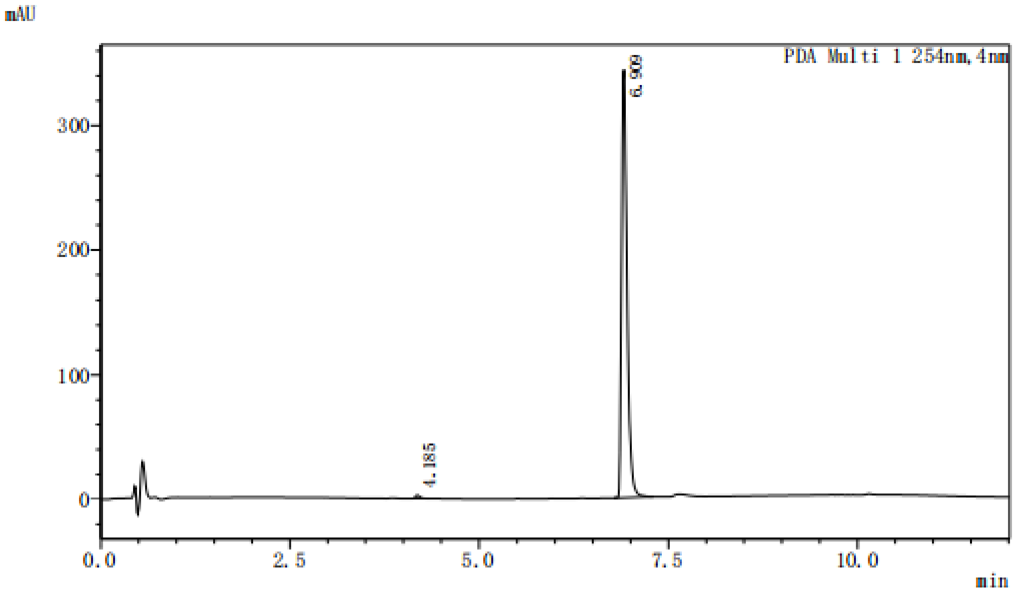

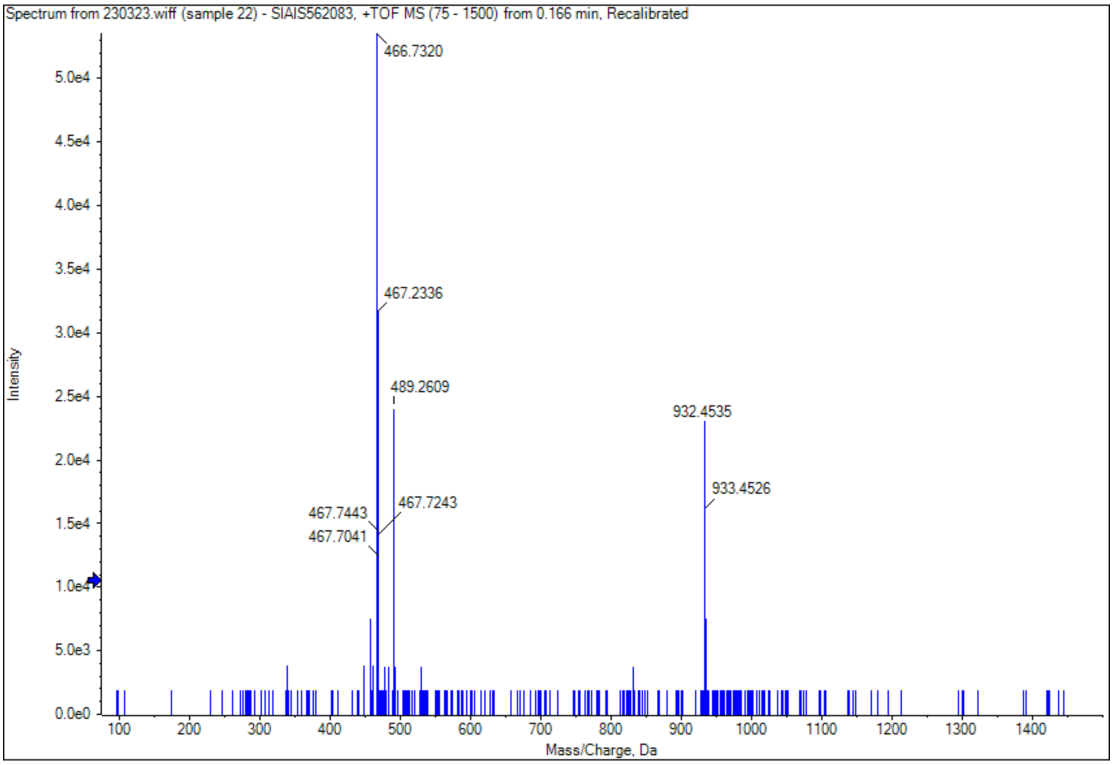

Figure S35. Purity spectrum and mass spectrum of compound **B11.**

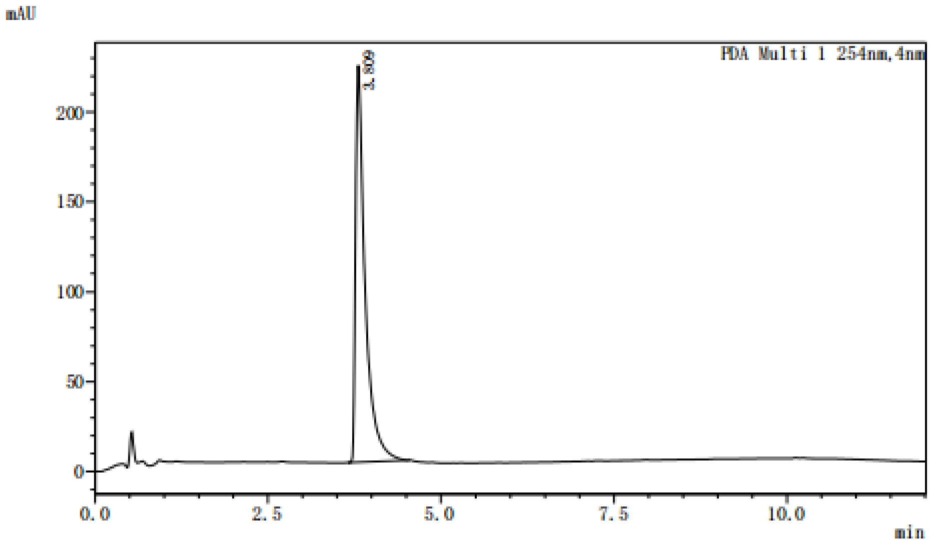

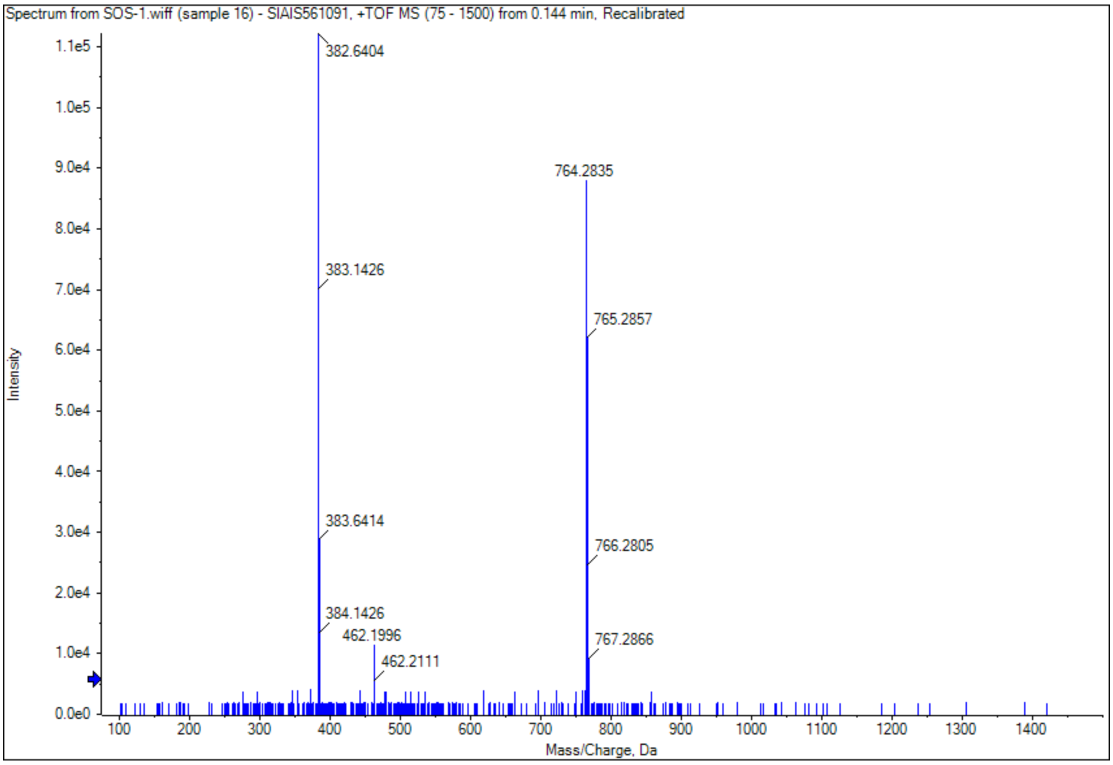

Figure S36. Purity spectrum and mass spectrum of compound **C1.**

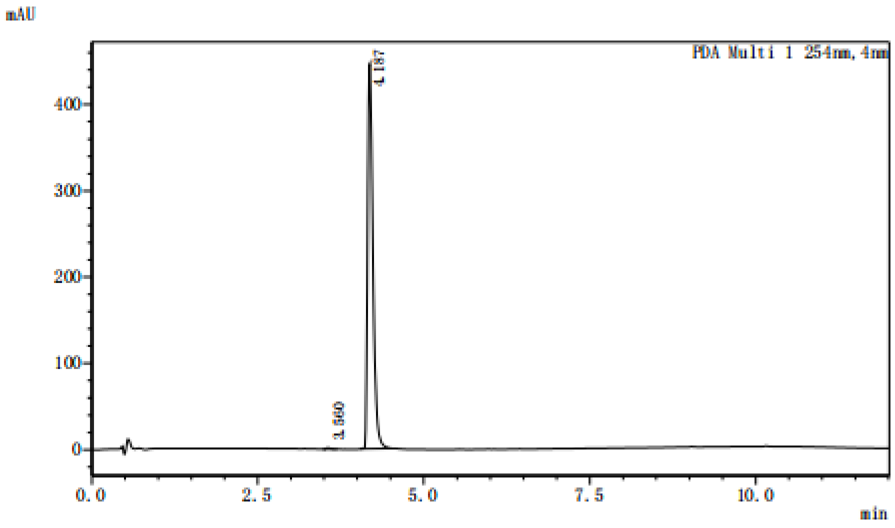

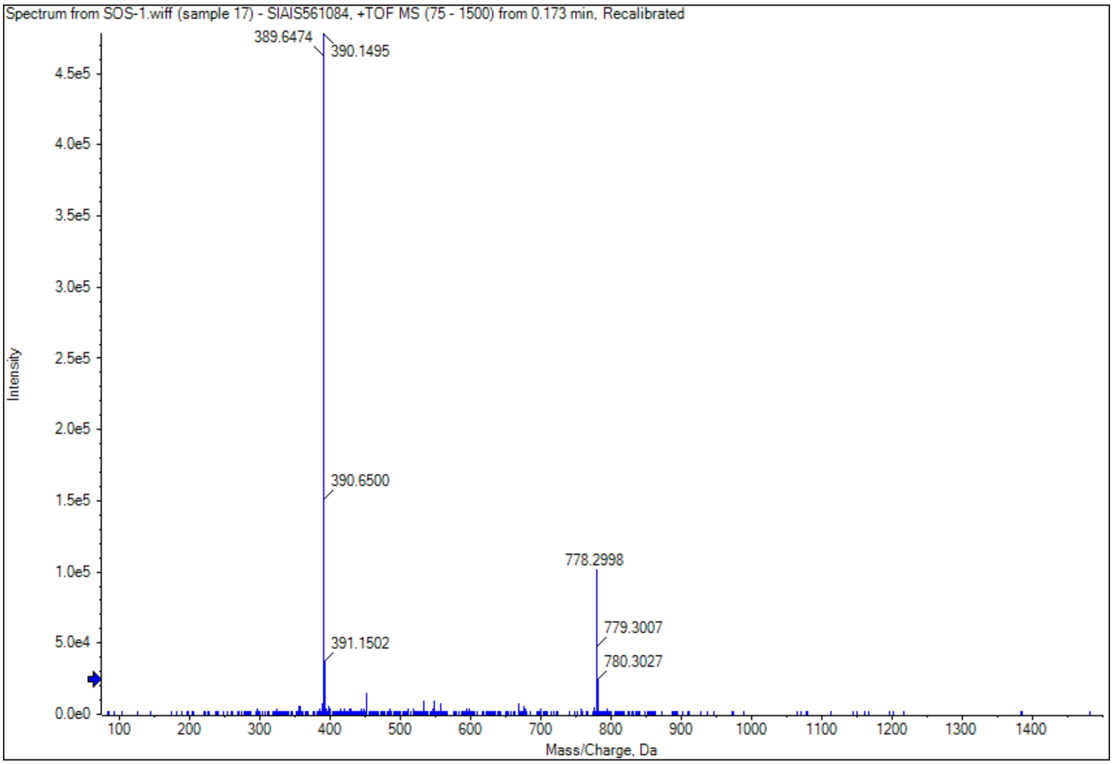

Figure S37. Purity spectrum and mass spectrum of compound **C2.**

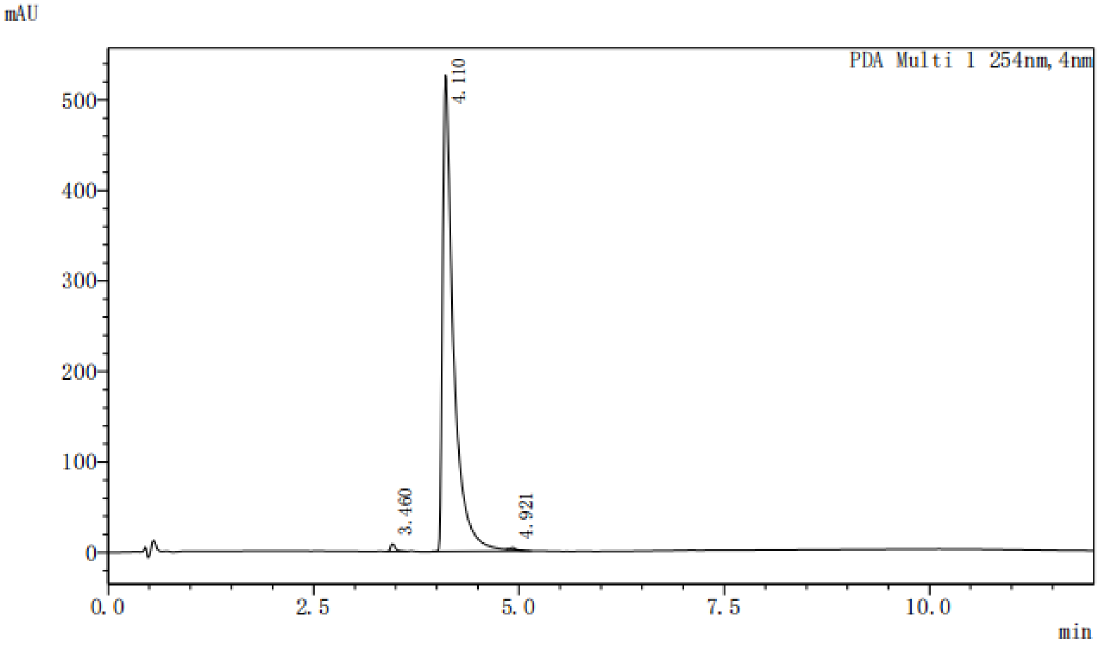

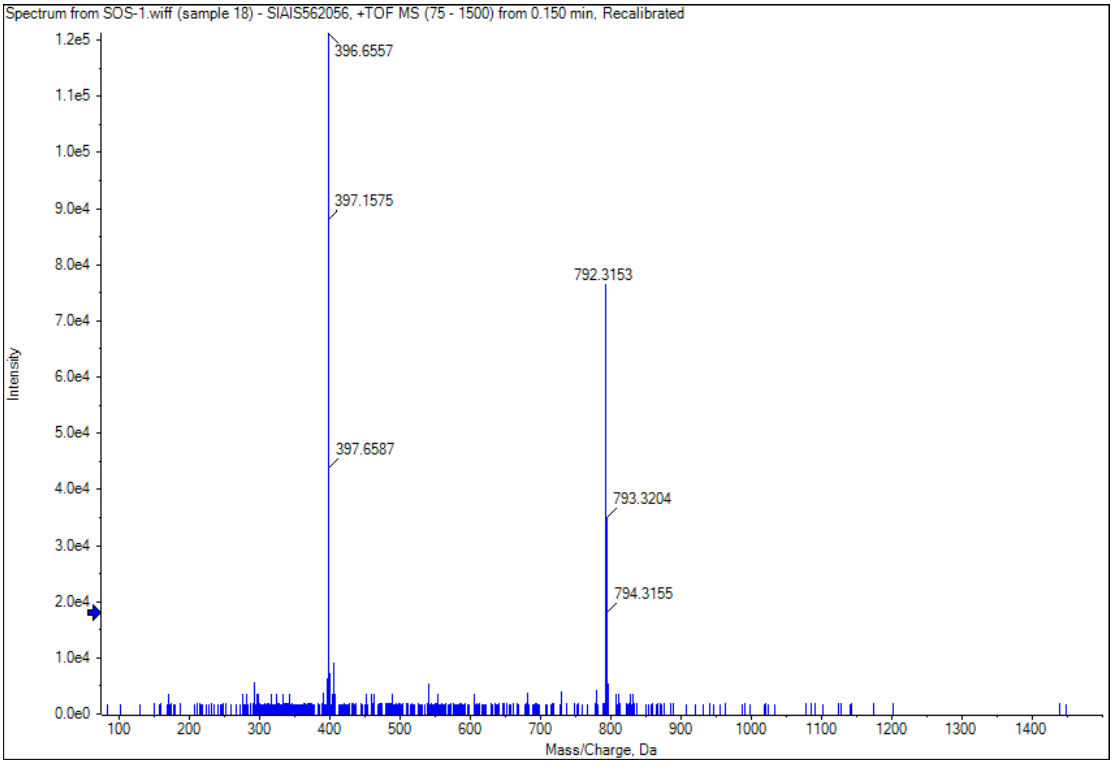

Figure S38. Purity spectrum and mass spectrum of compound **C3.**

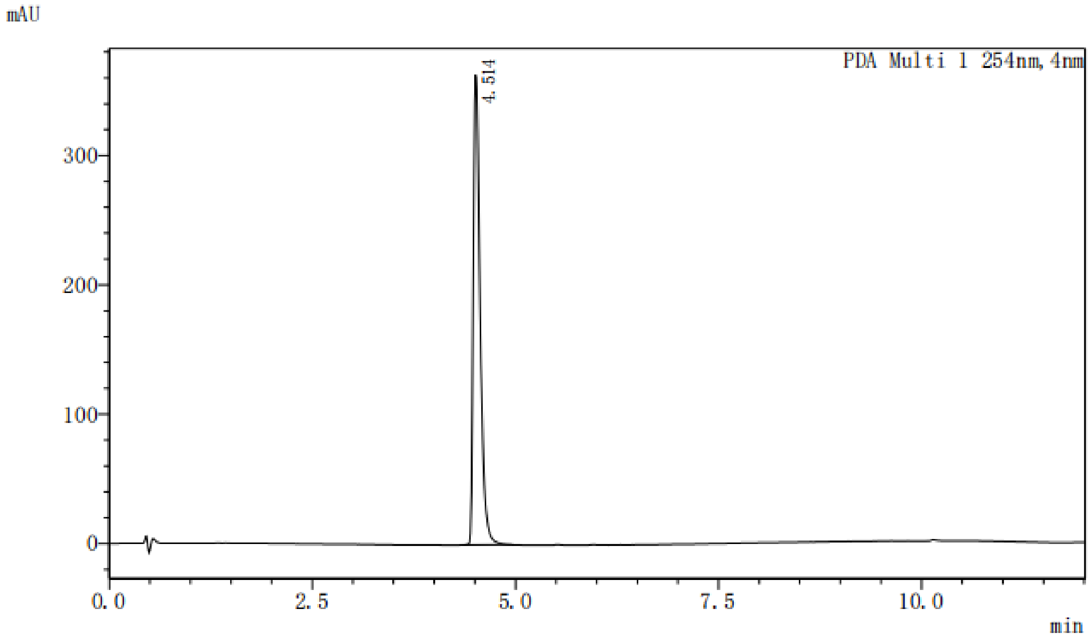

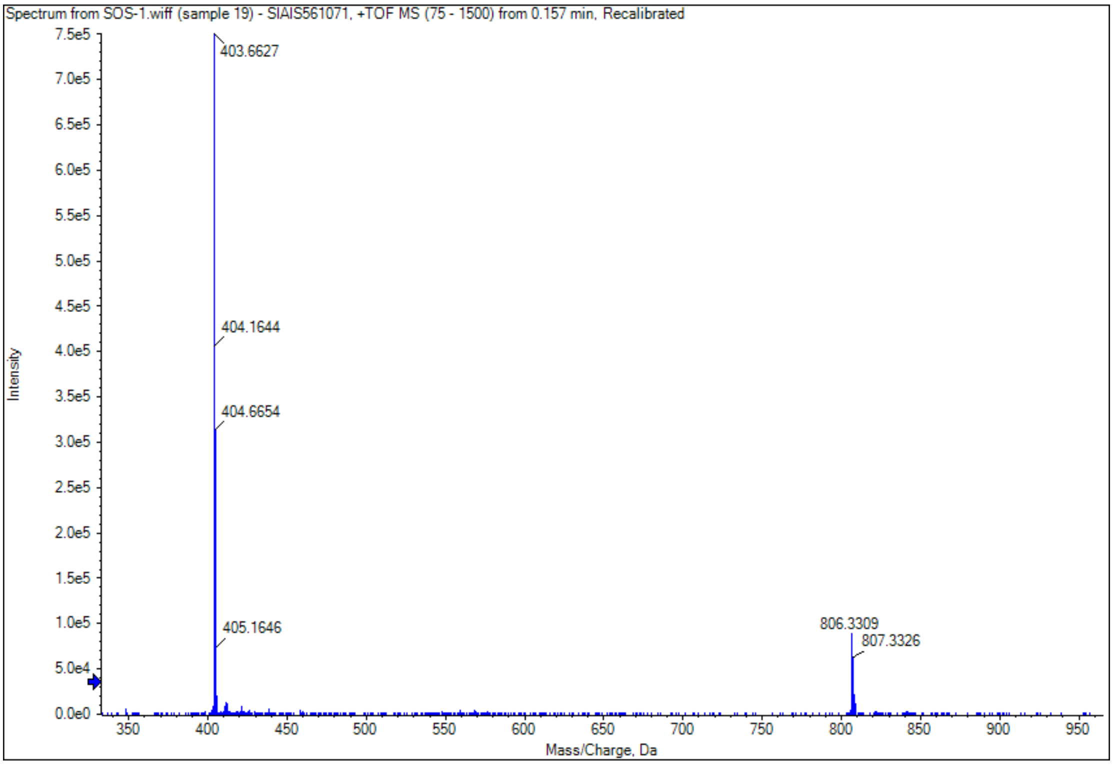

Figure S39. Purity spectrum and mass spectrum of compound **C4.**

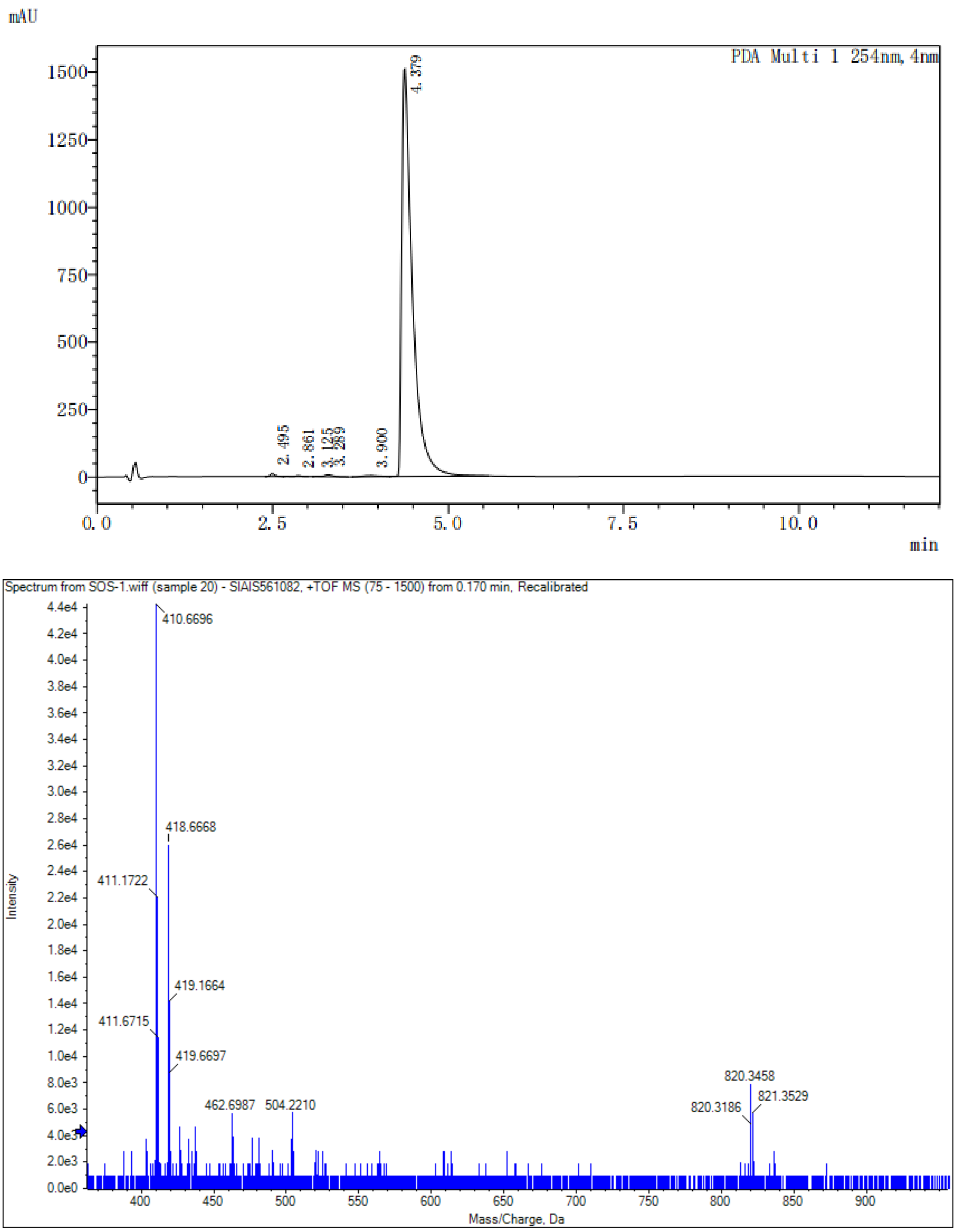

Figure S40. Purity spectrum and mass spectrum of compound **C5.**

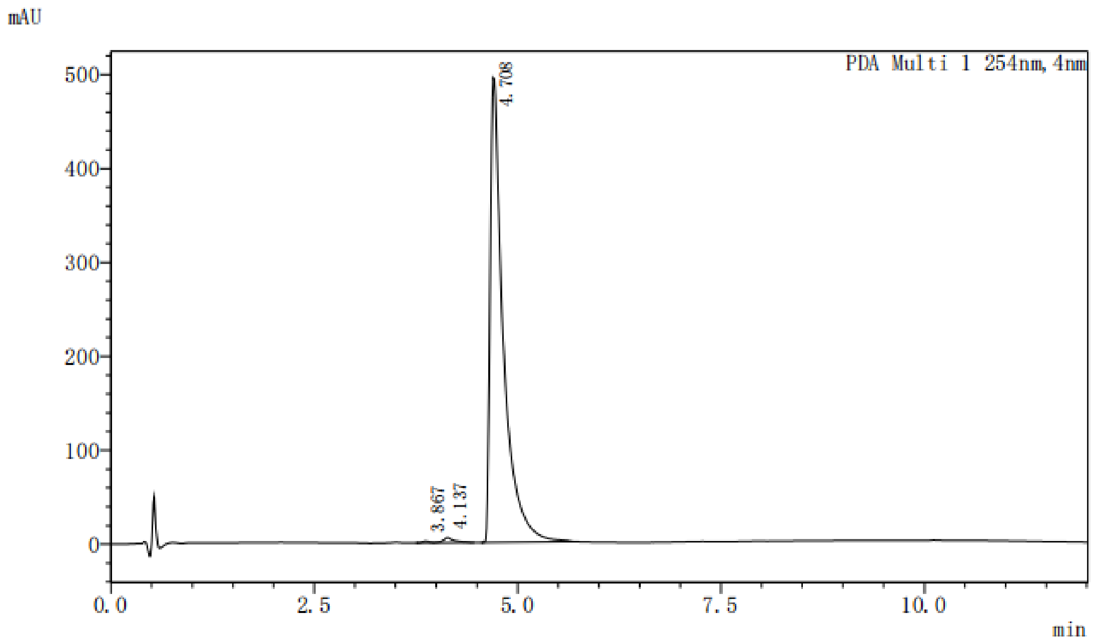

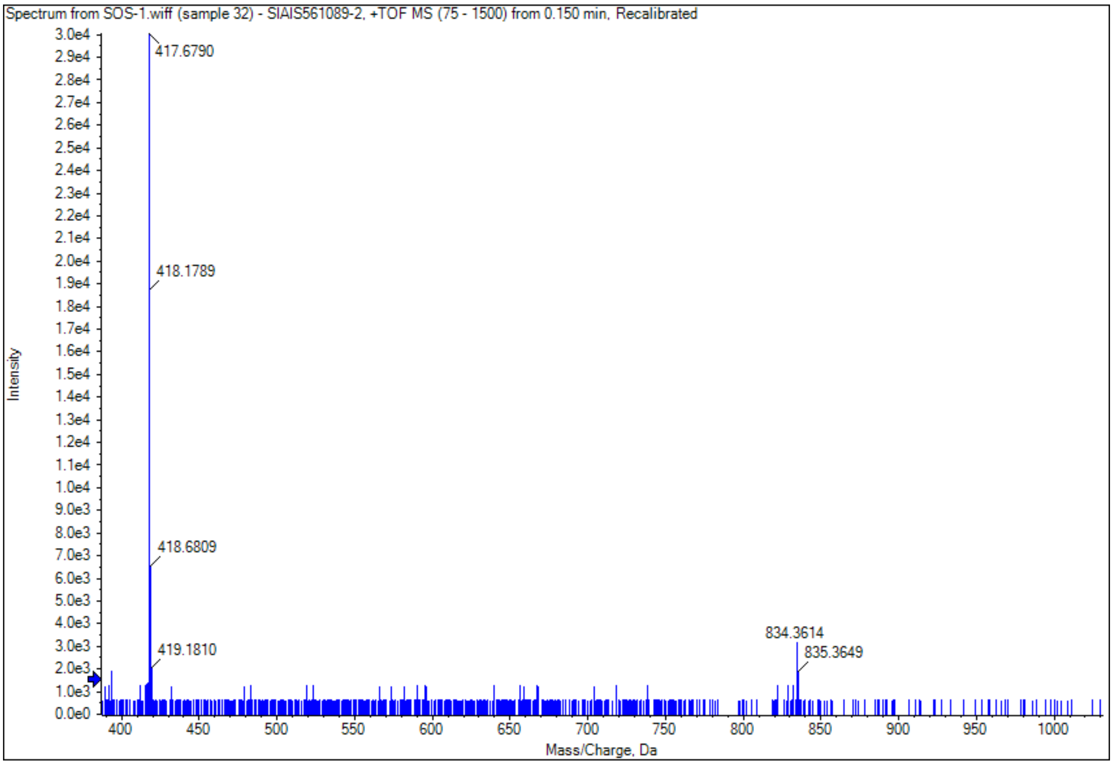

Figure S41. Purity spectrum and mass spectrum of compound **C6.**

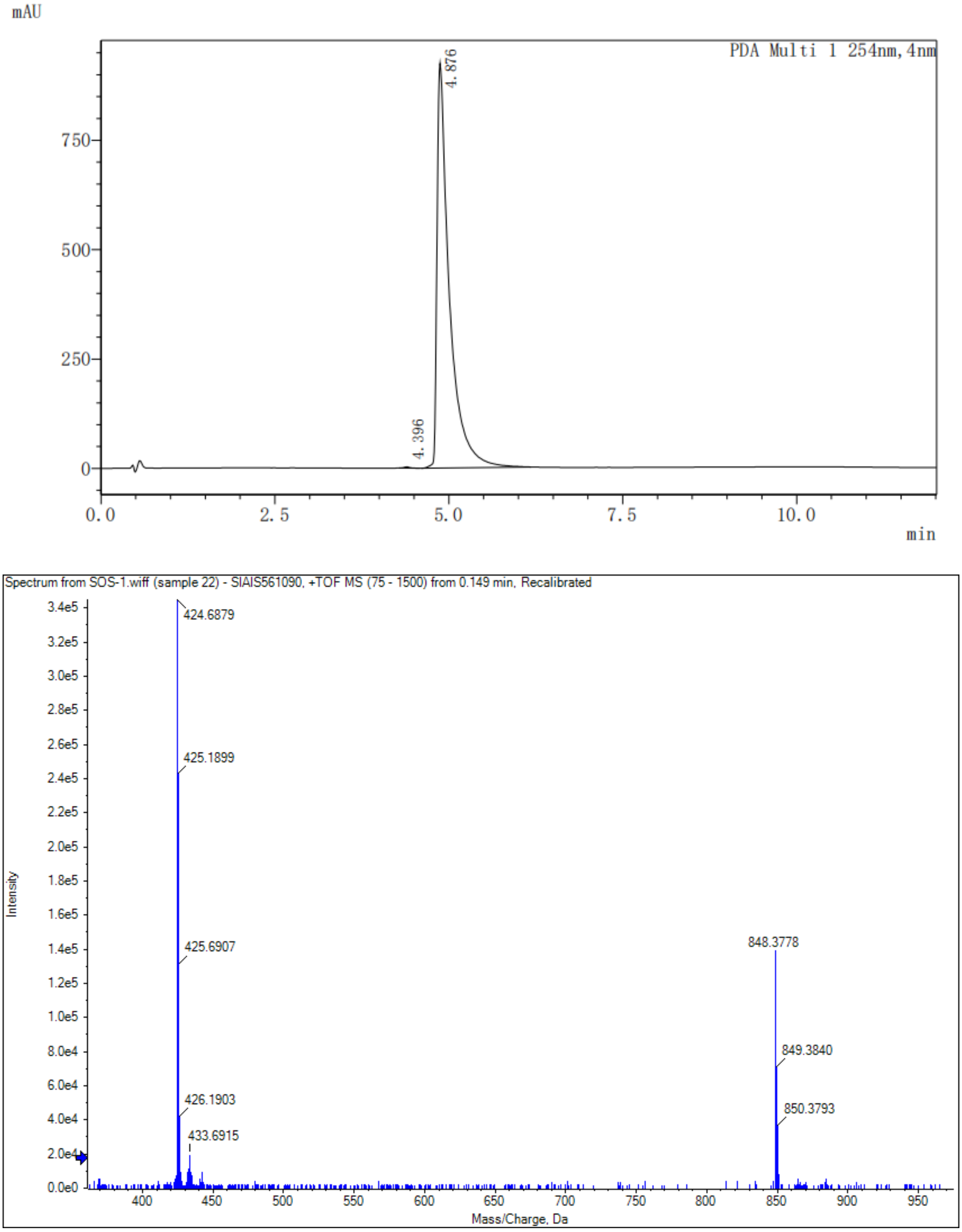

Figure S42. Purity spectrum and mass spectrum of compound **C7.**

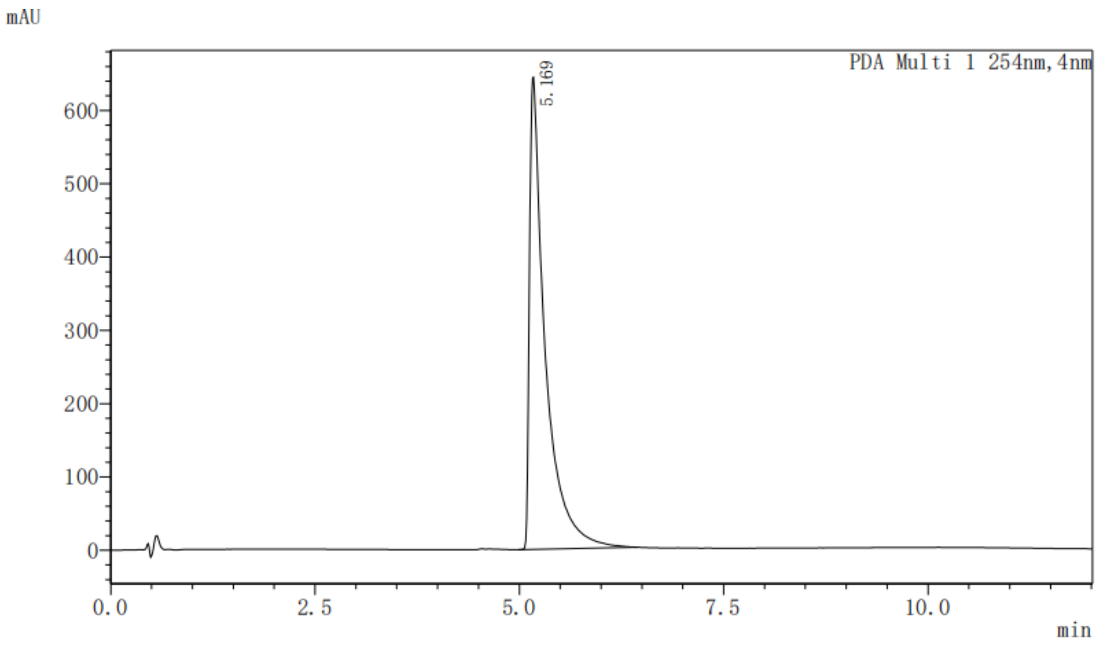

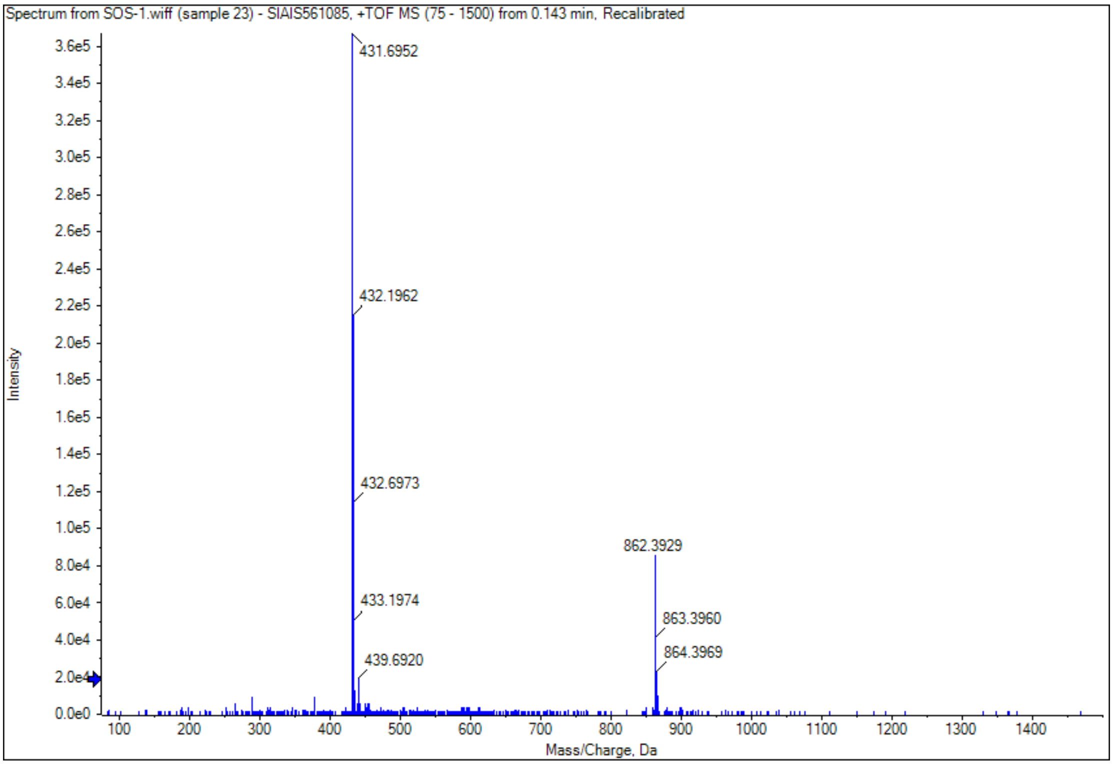

Figure S43. Purity spectrum and mass spectrum of compound **C8.**

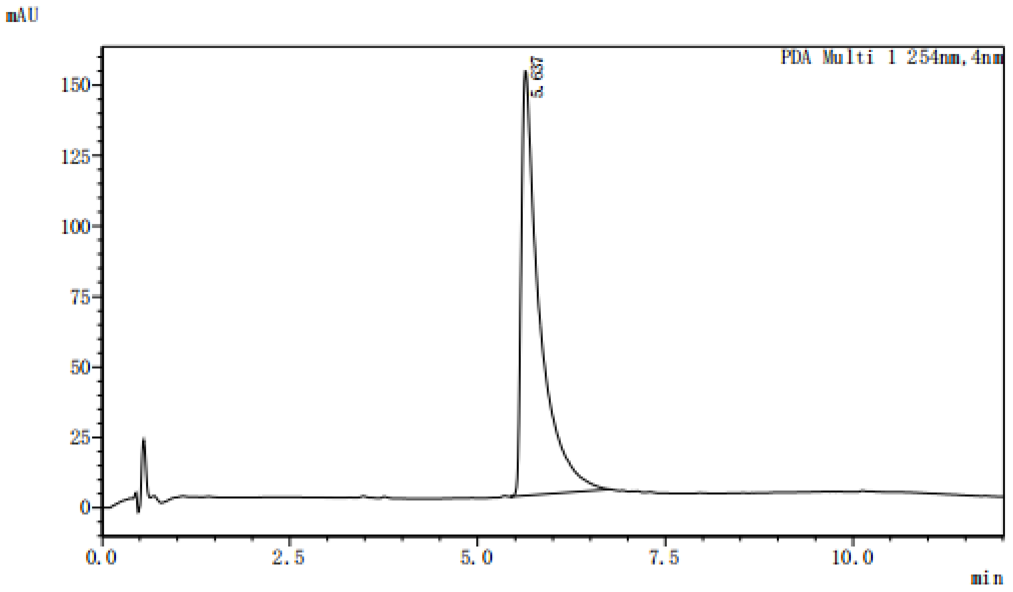

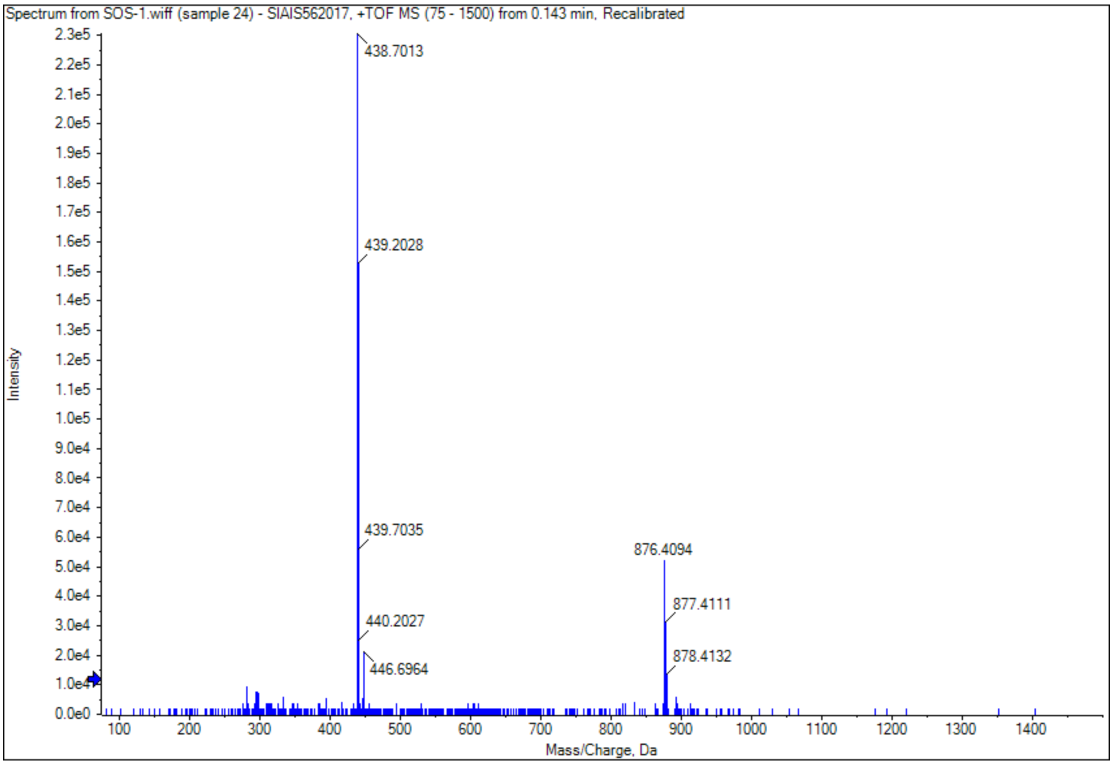

Figure S44. Purity spectrum and mass spectrum of compound **C9.**

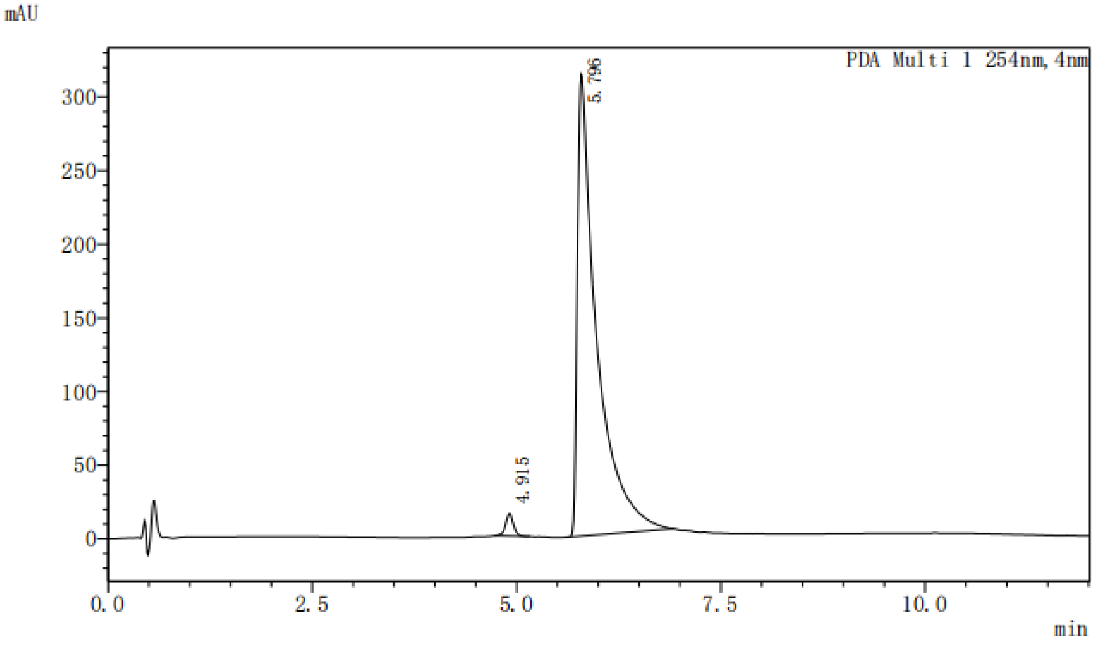

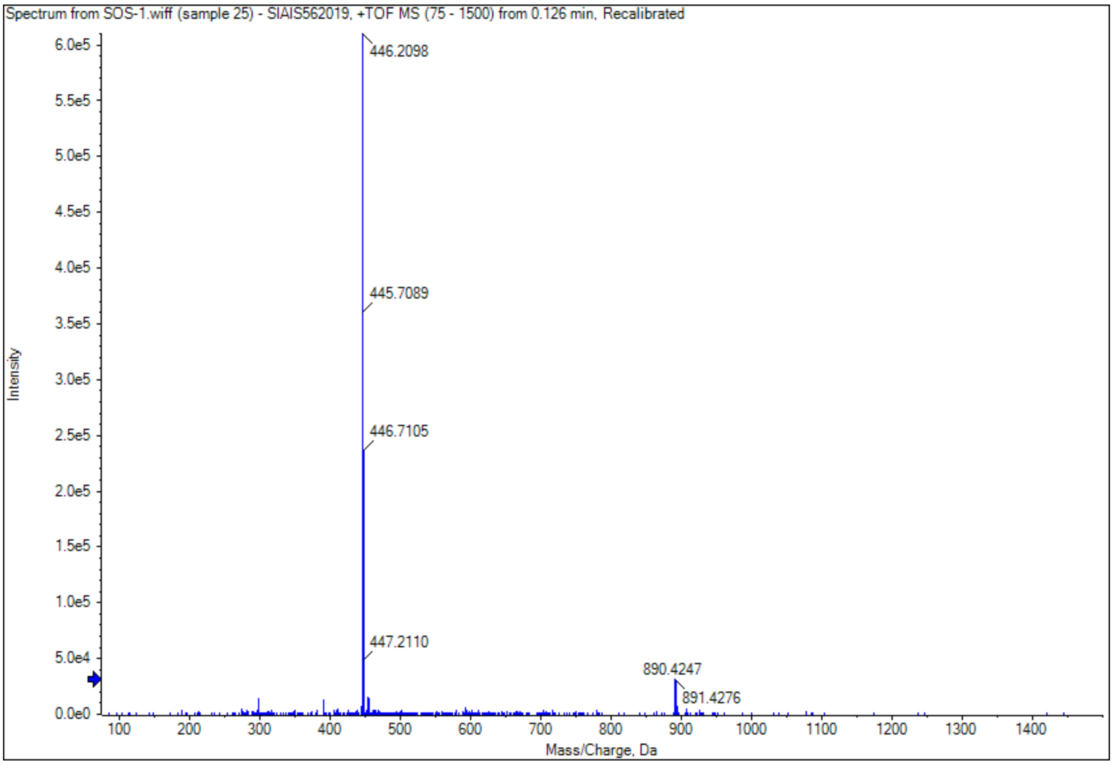

Figure S45. Purity spectrum and mass spectrum of compound **C10.**

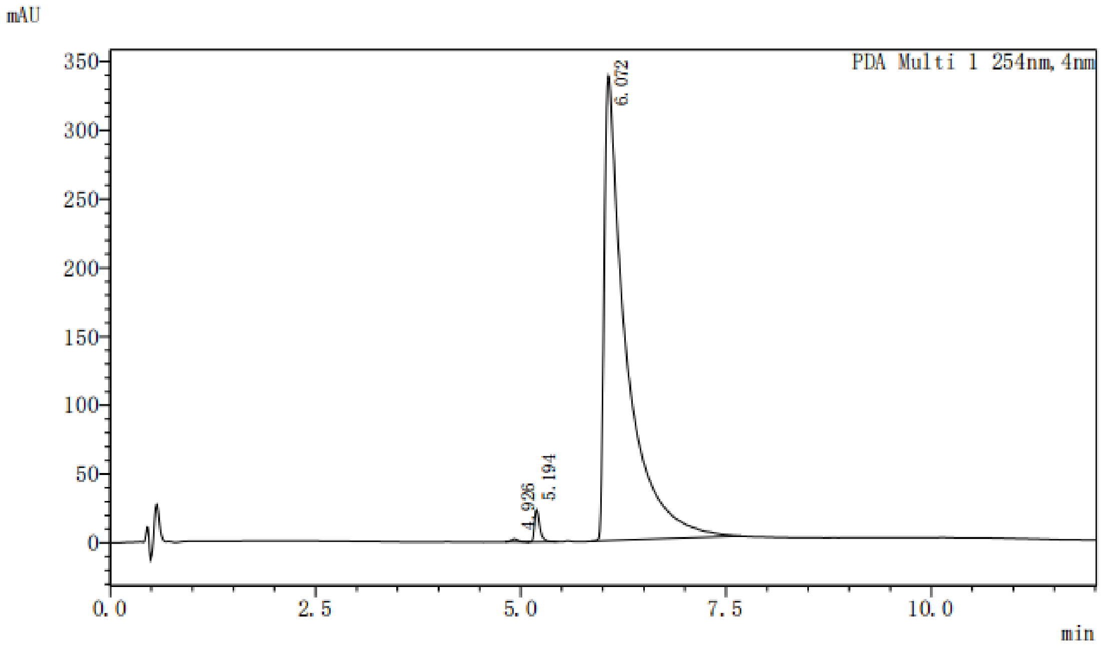

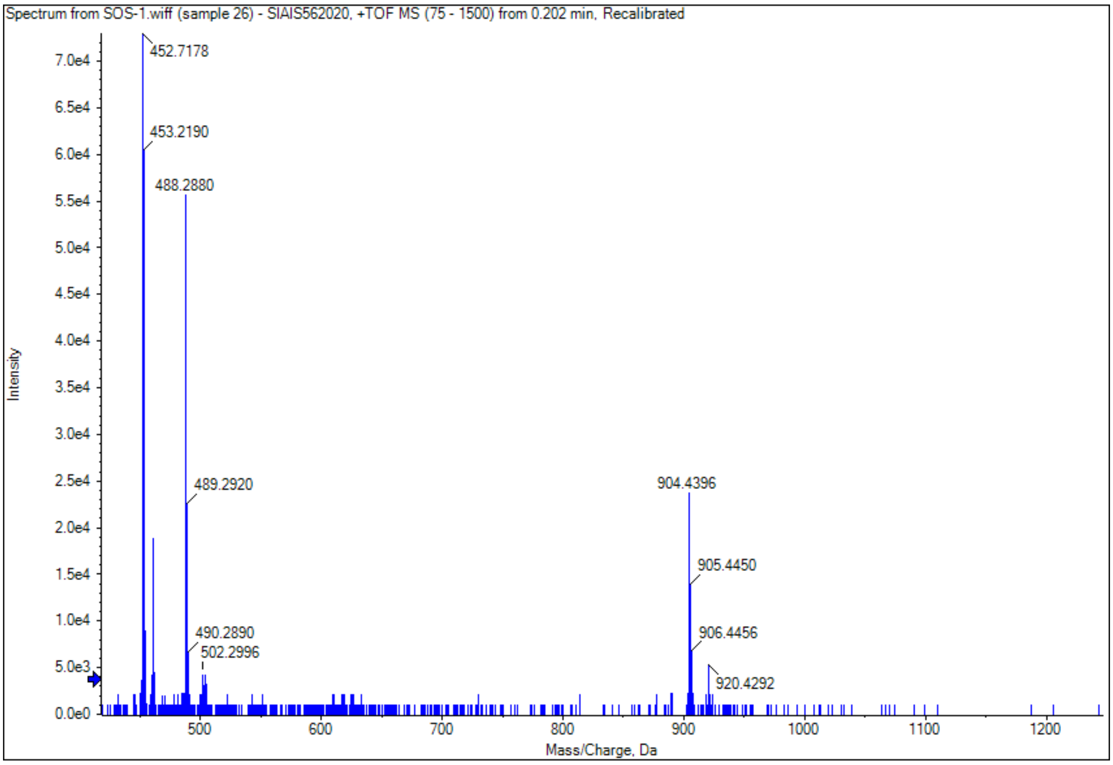

Figure S46. Purity spectrum and mass spectrum of compound **C11.**

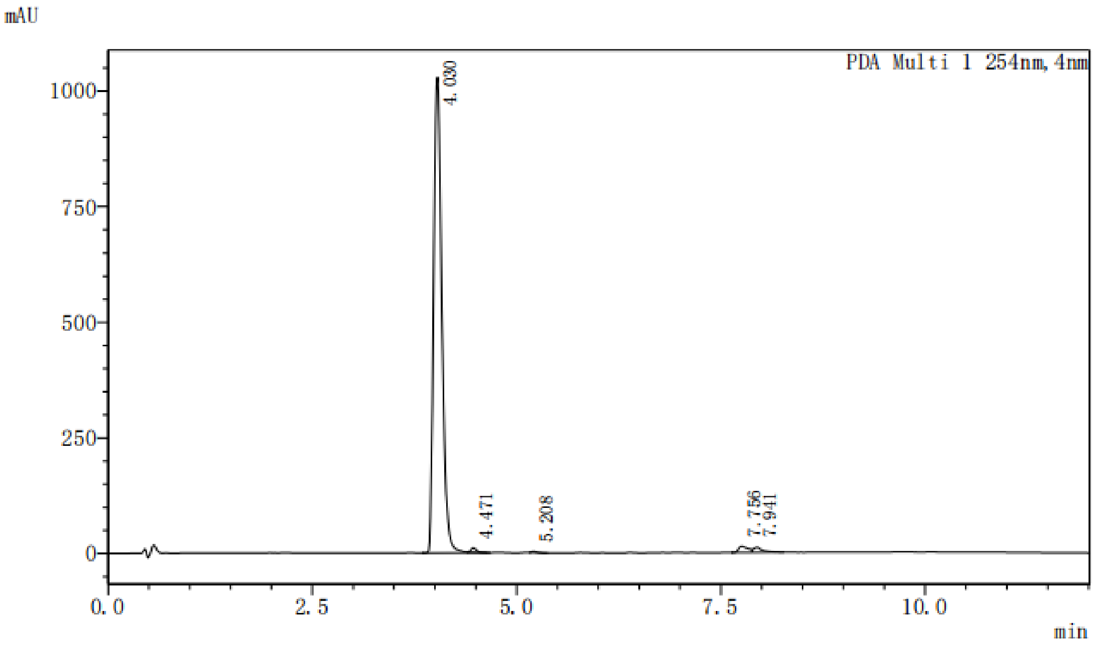

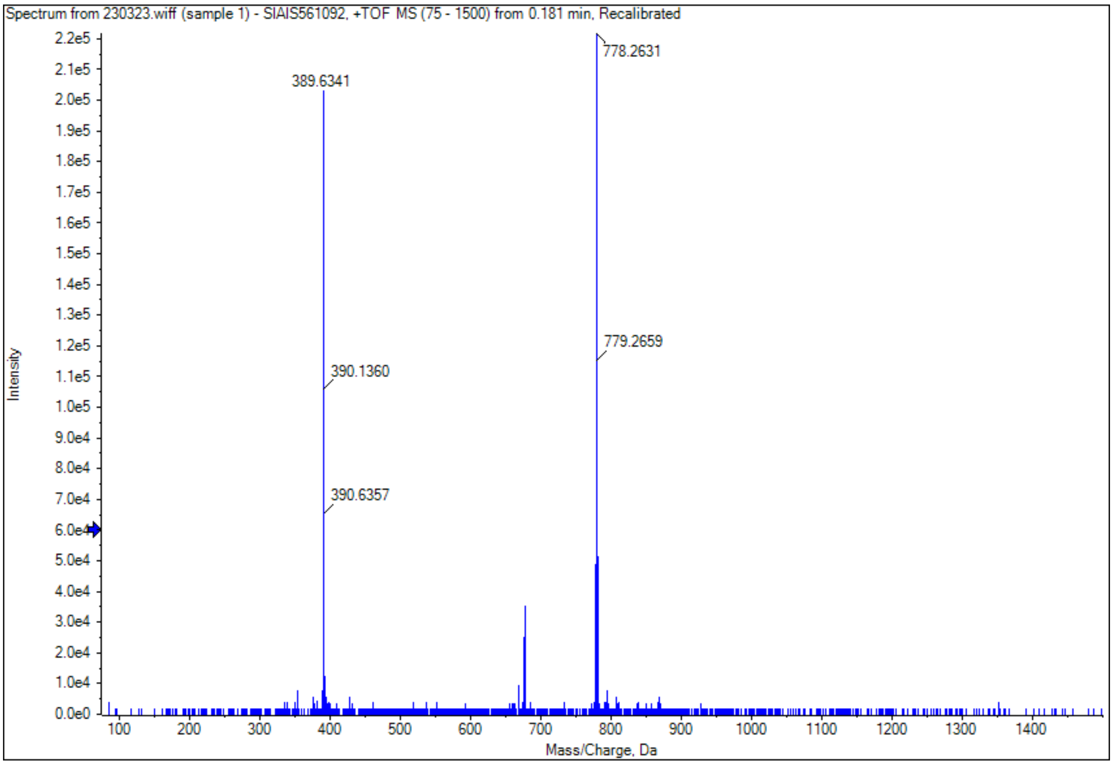

Figure S47. Purity spectrum and mass spectrum of compound **D1.**

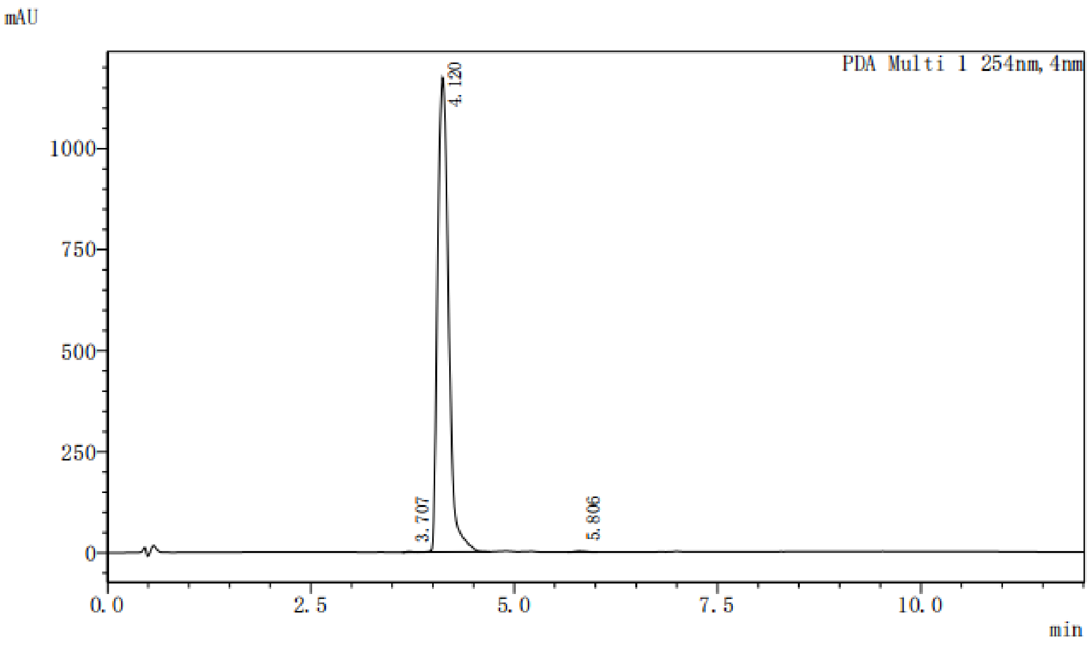

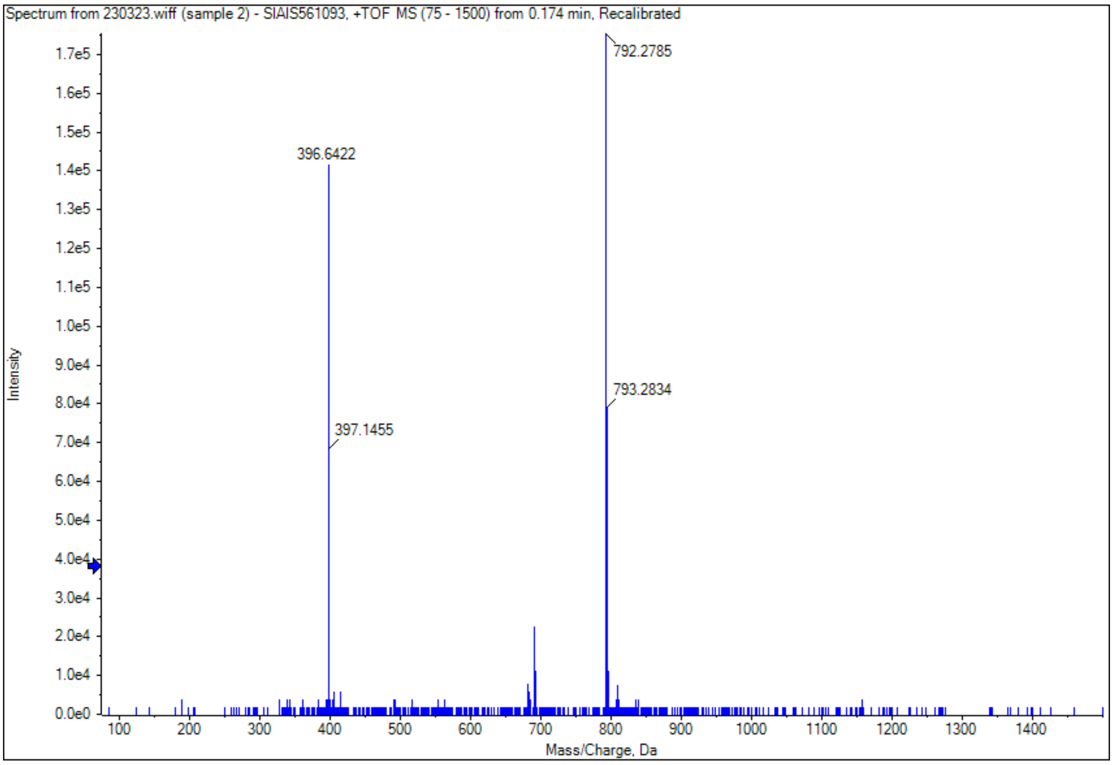

Figure S48. Purity spectrum and mass spectrum of compound **D2.**

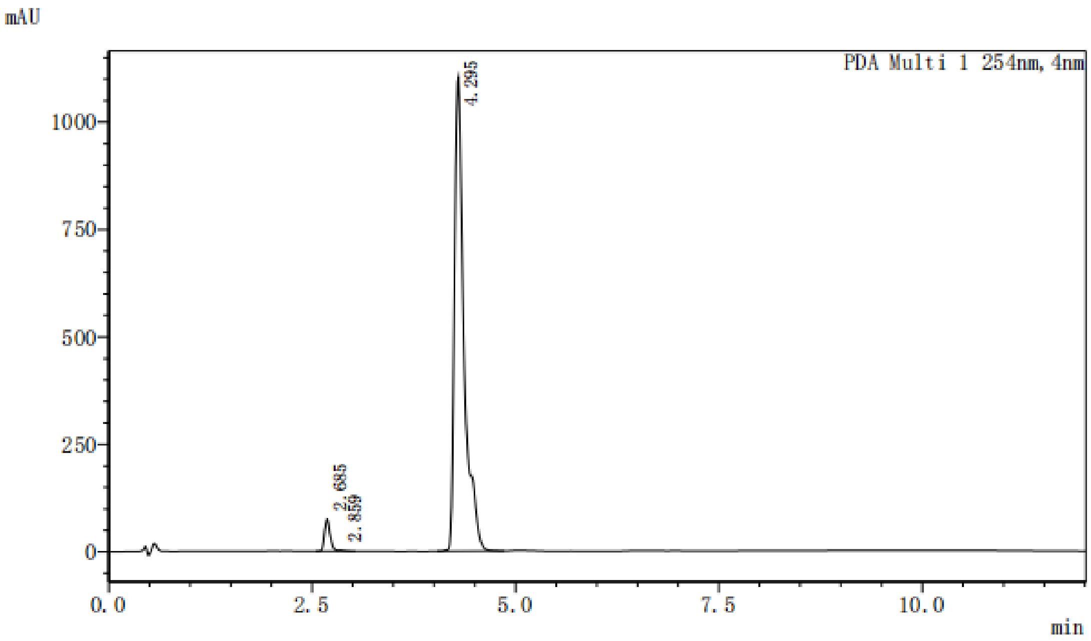

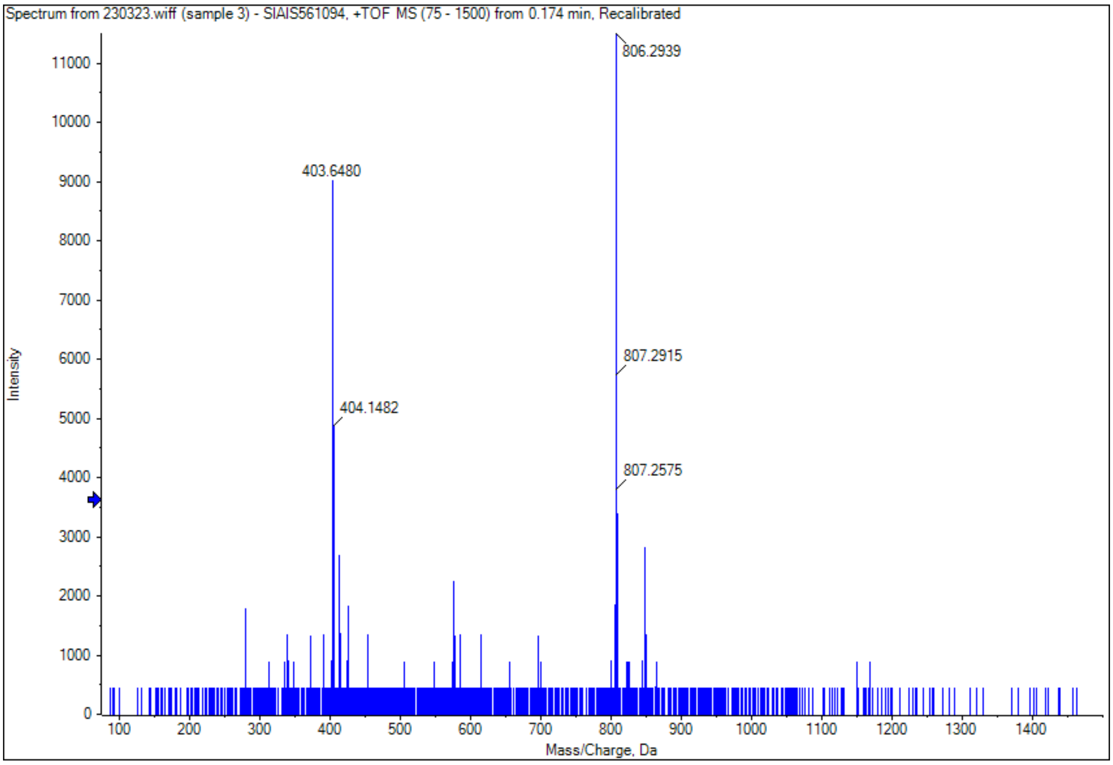

Figure S49. Purity spectrum and mass spectrum of compound **D3.**

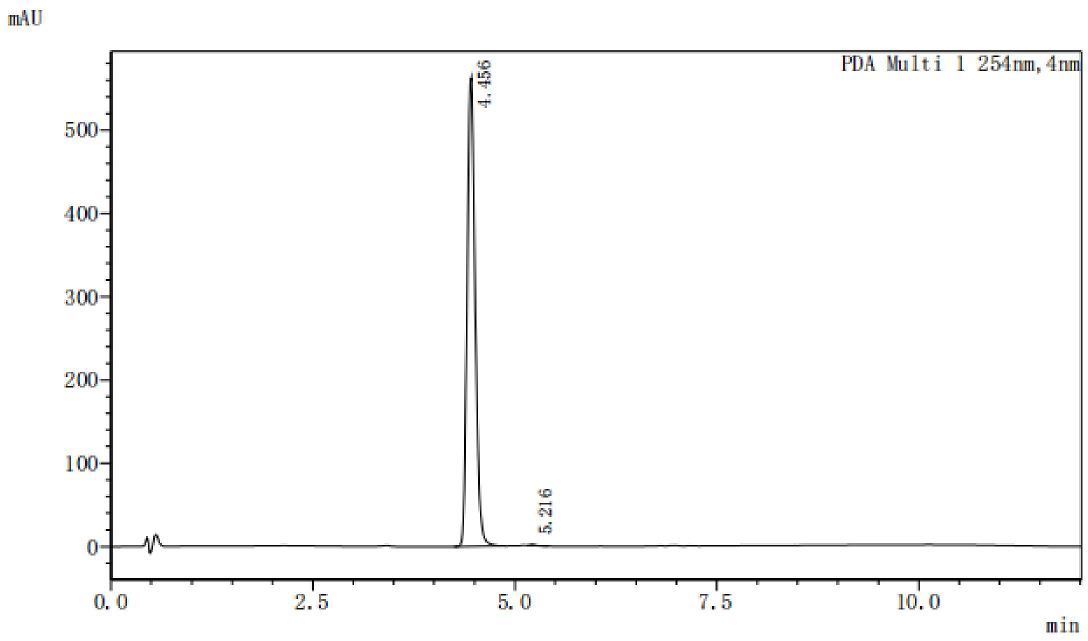

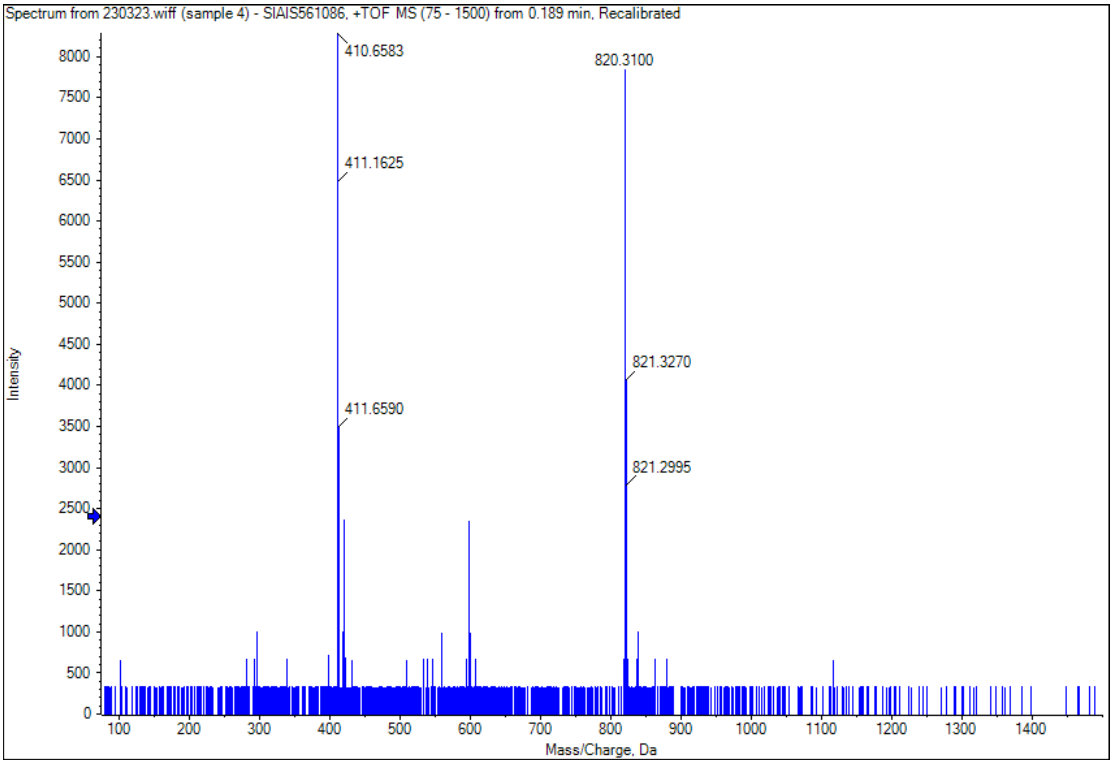

Figure S50. Purity spectrum and mass spectrum of compound **D4.**

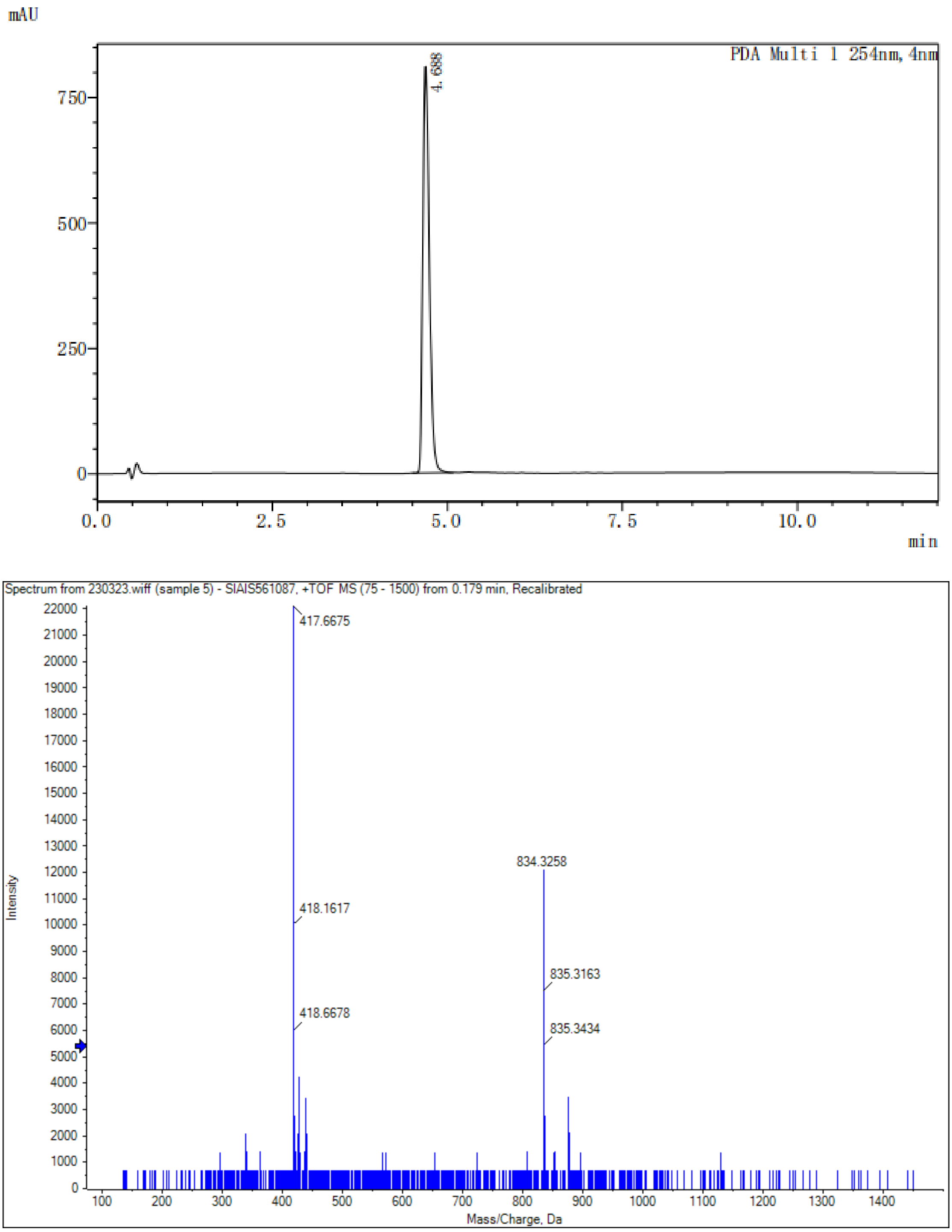

Figure S51. Purity spectrum and mass spectrum of compound **D5.**

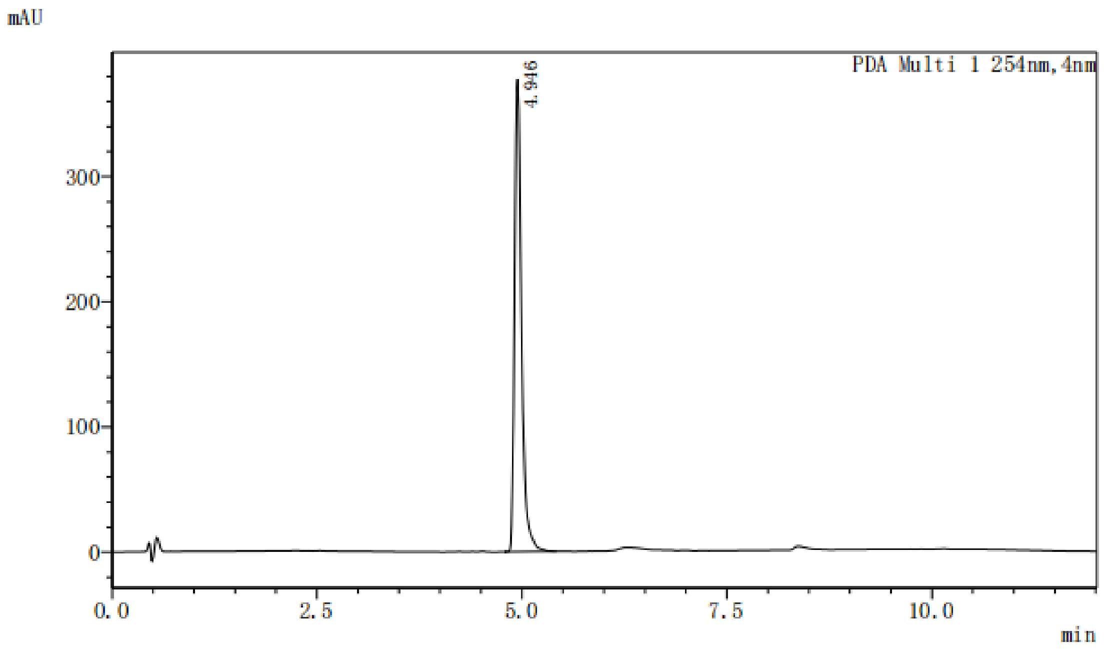

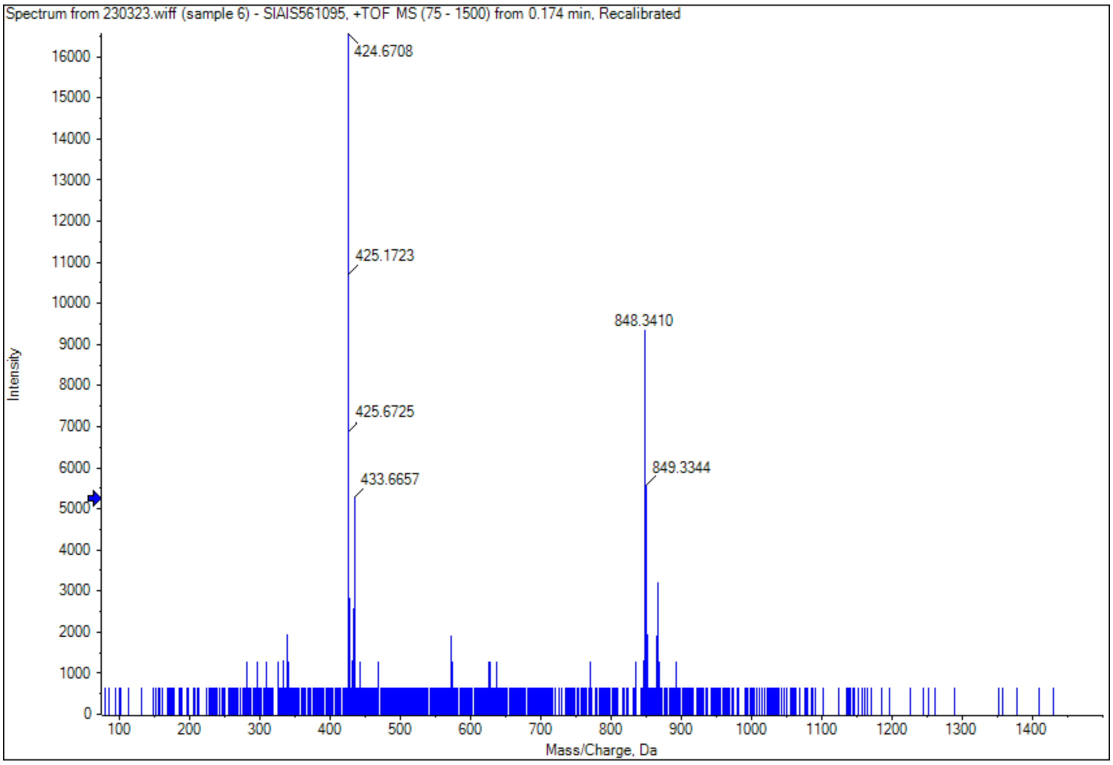

Figure S52. Purity spectrum and mass spectrum of compound **D6.**

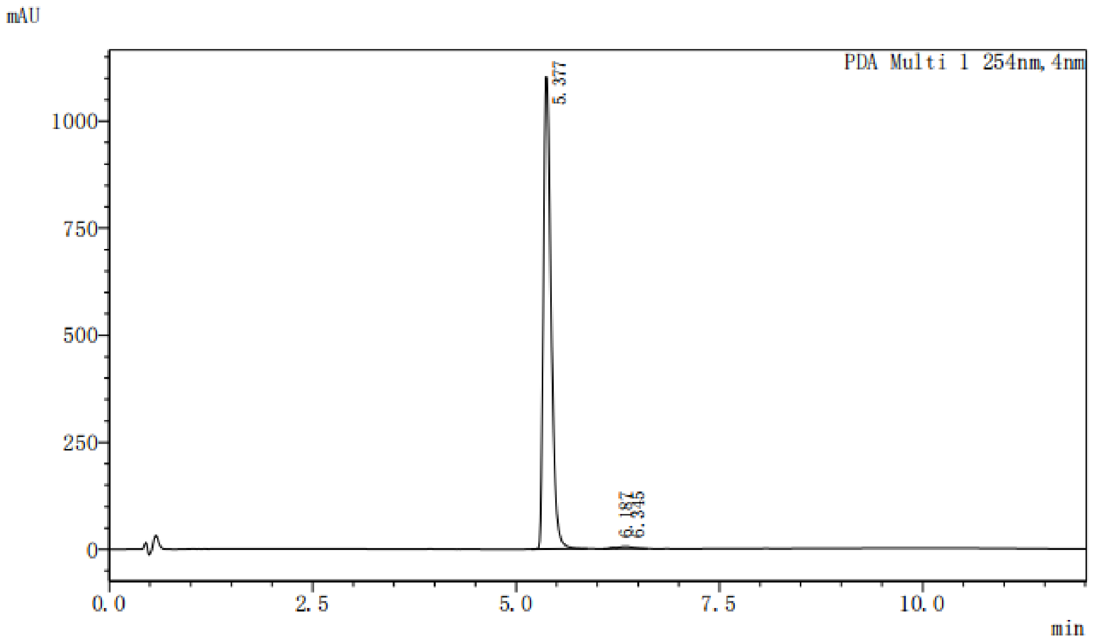

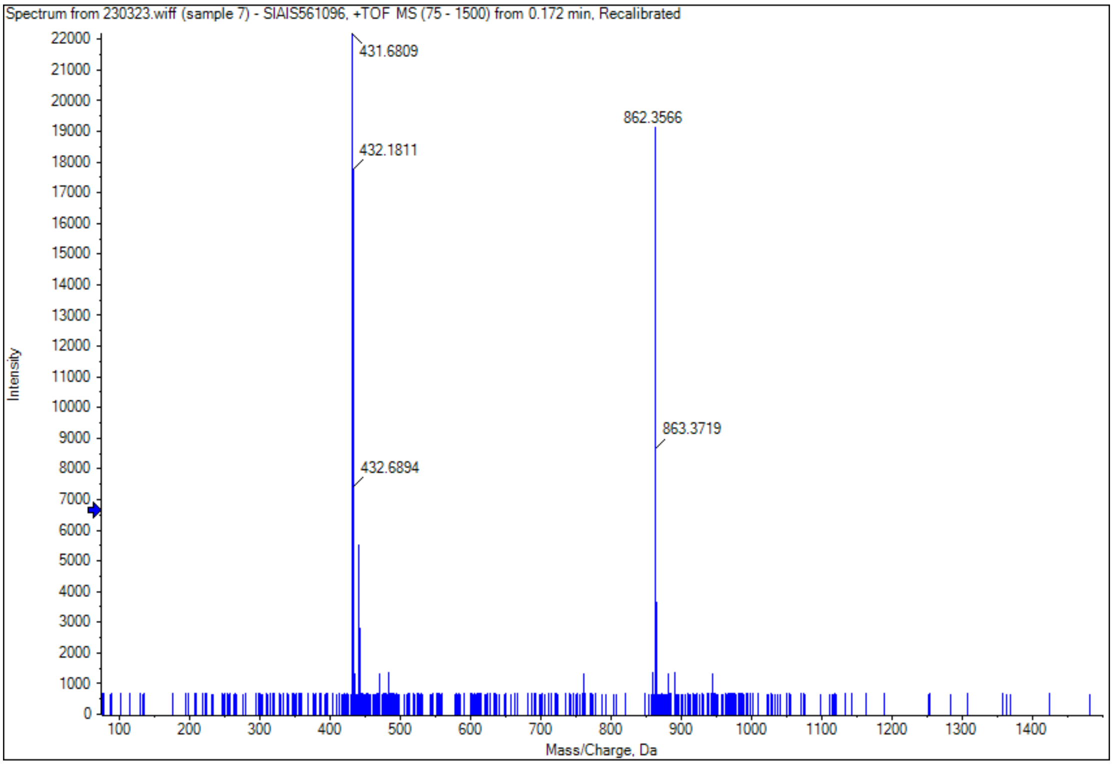

Figure S53. Purity spectrum and mass spectrum of compound **D7.**

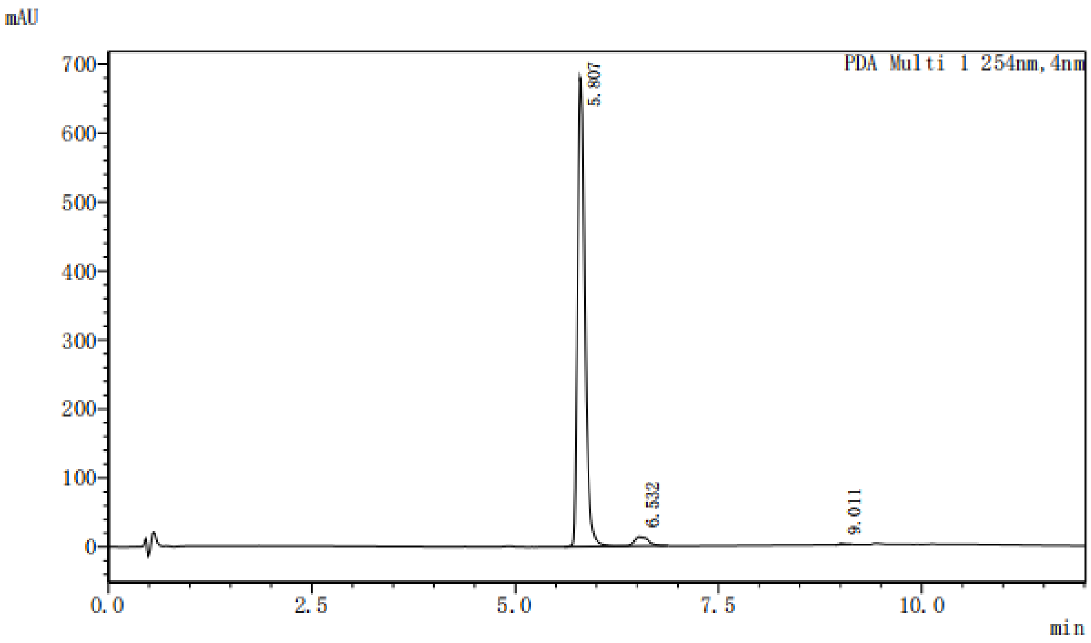

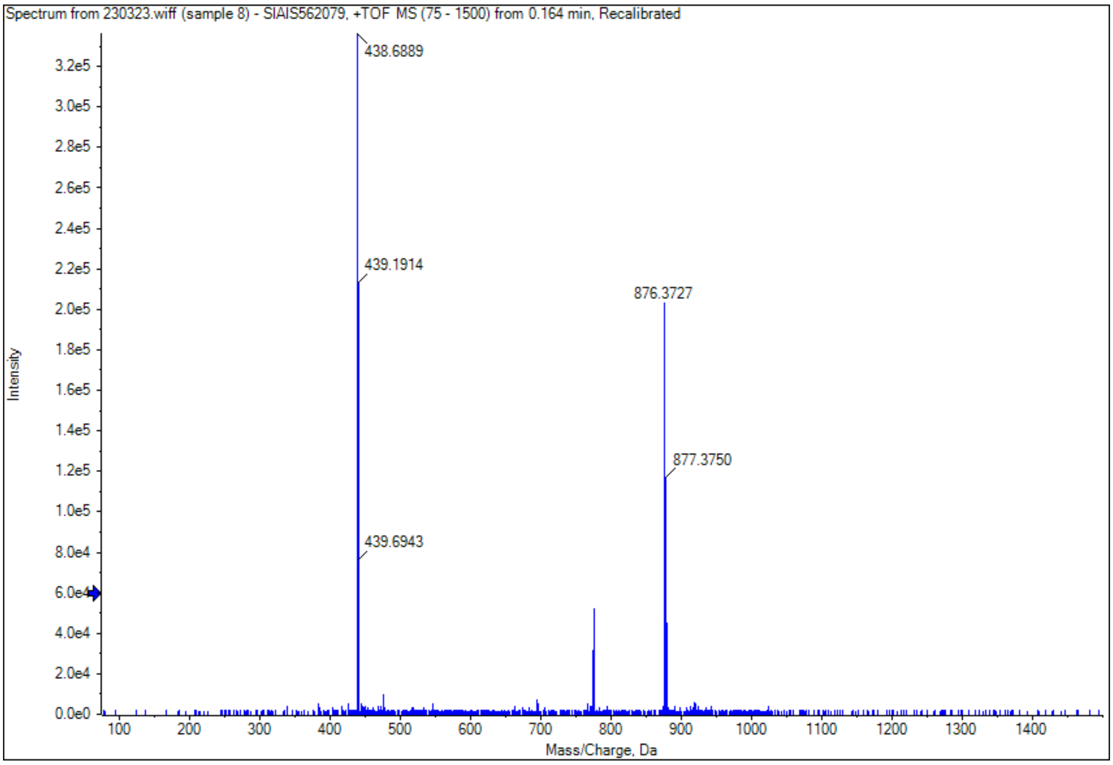

Figure S54. Purity spectrum and mass spectrum of compound **D8.**

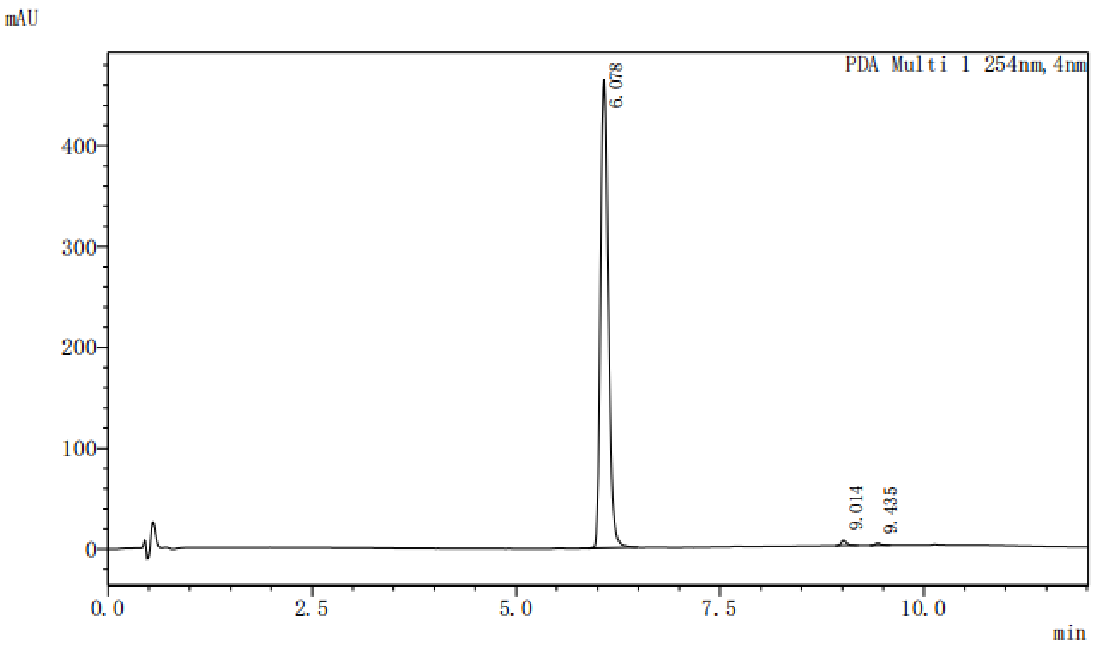

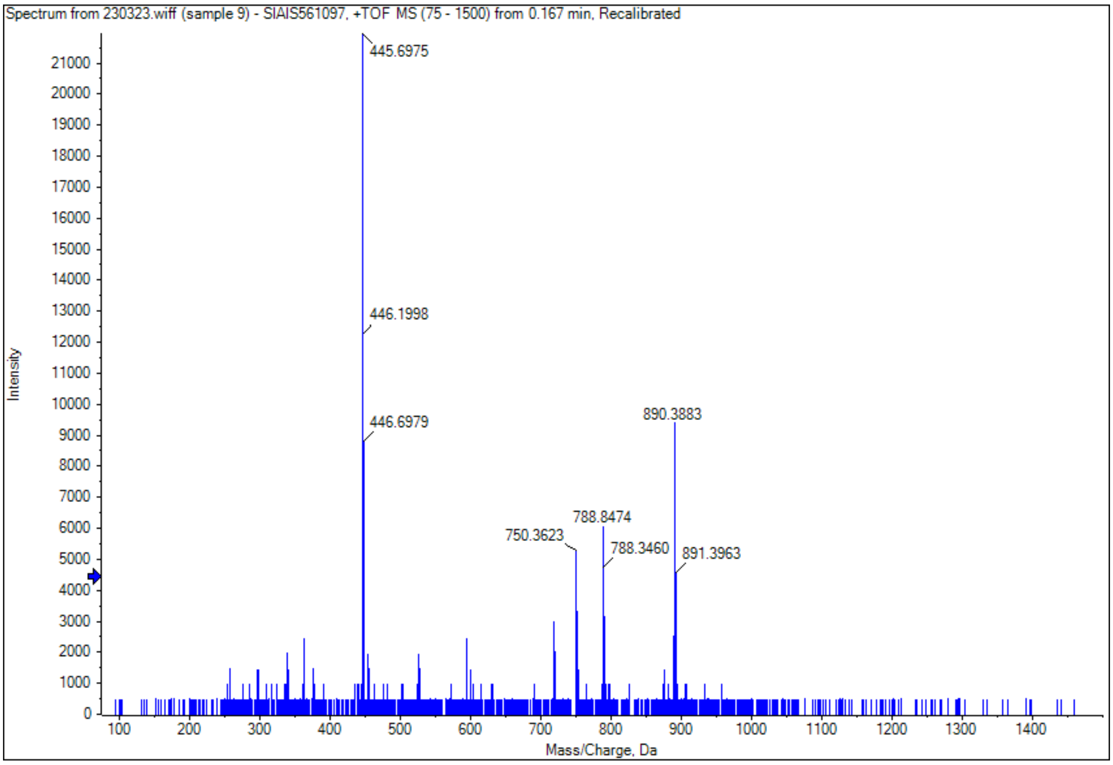

Figure S55. Purity spectrum and mass spectrum of compound **D9.**

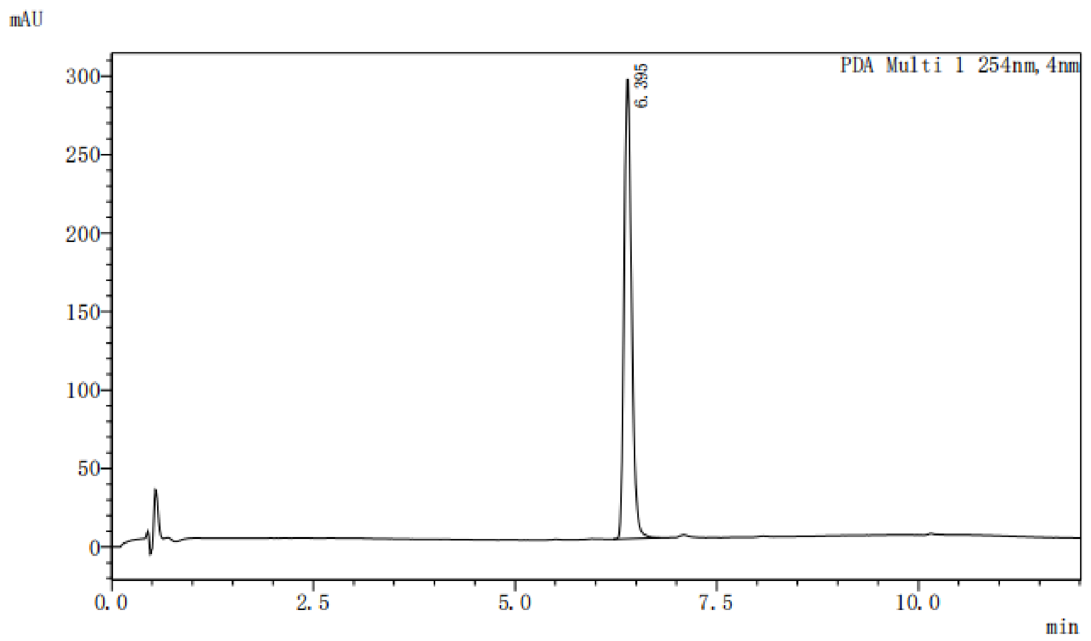

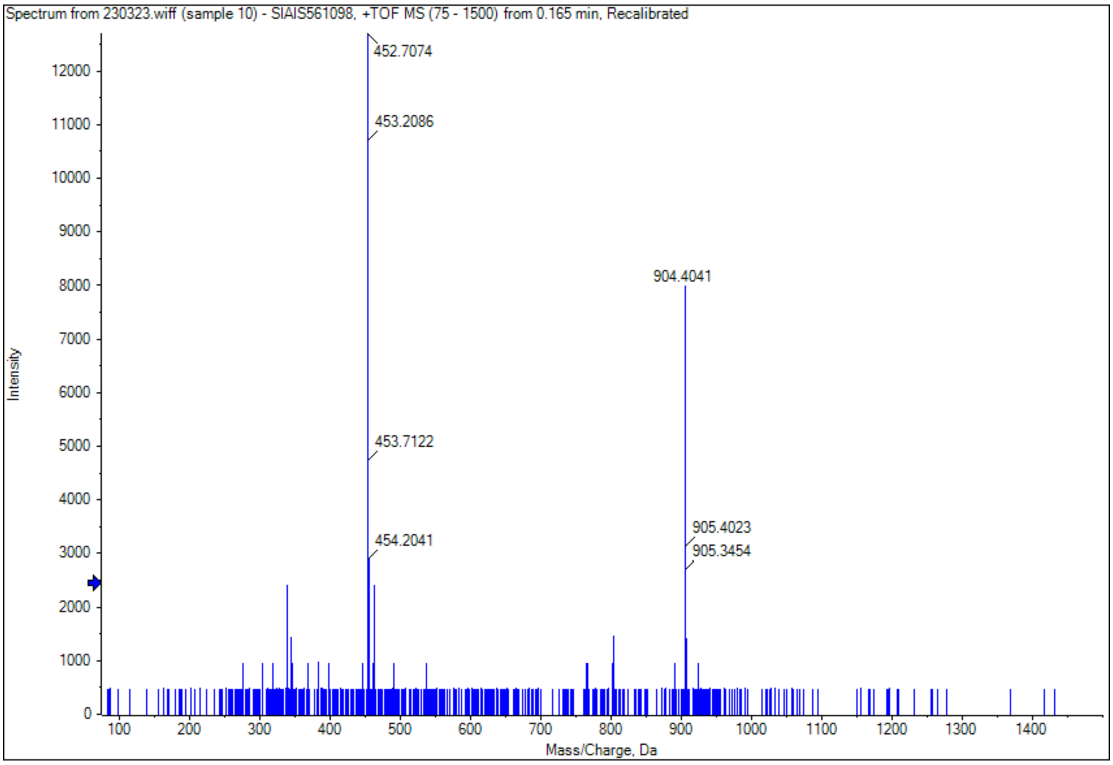

Figure S56. Purity spectrum and mass spectrum of compound **D10.**

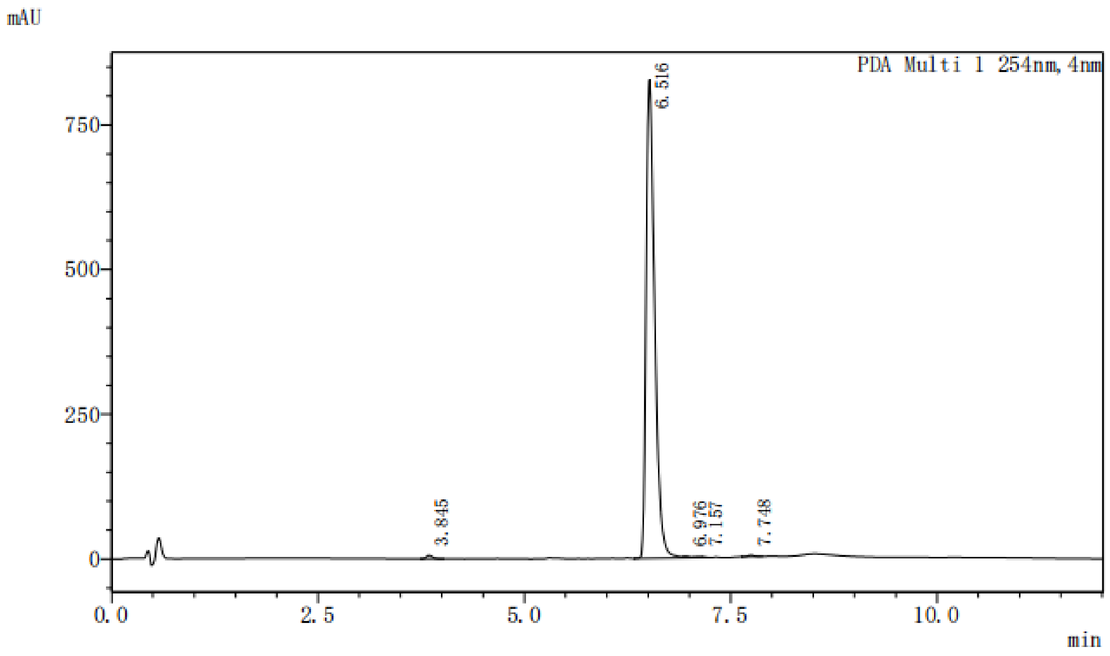

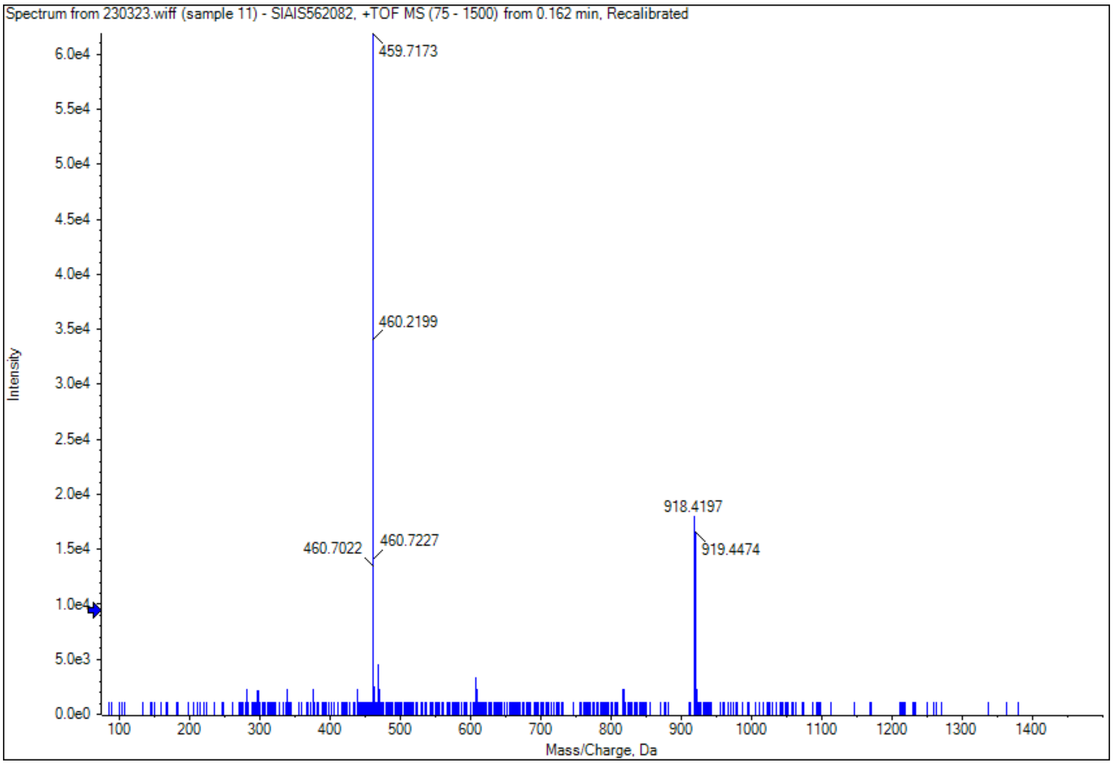

Figure S57. Purity spectrum and mass spectrum of compound **D11.**

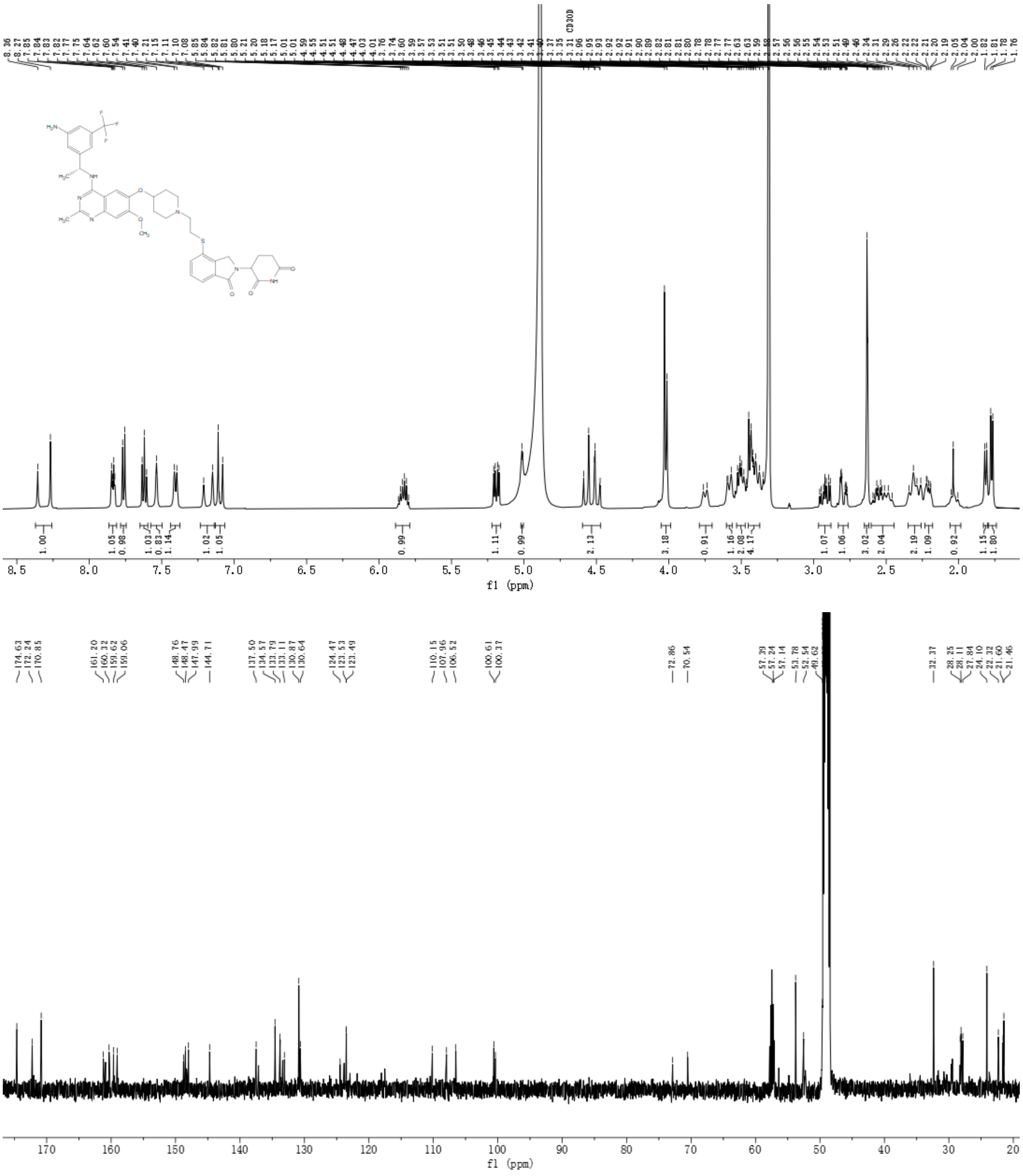

Figure S58. ^{1}H and ^{13}C spectra of **A1** in Methanol-D4.

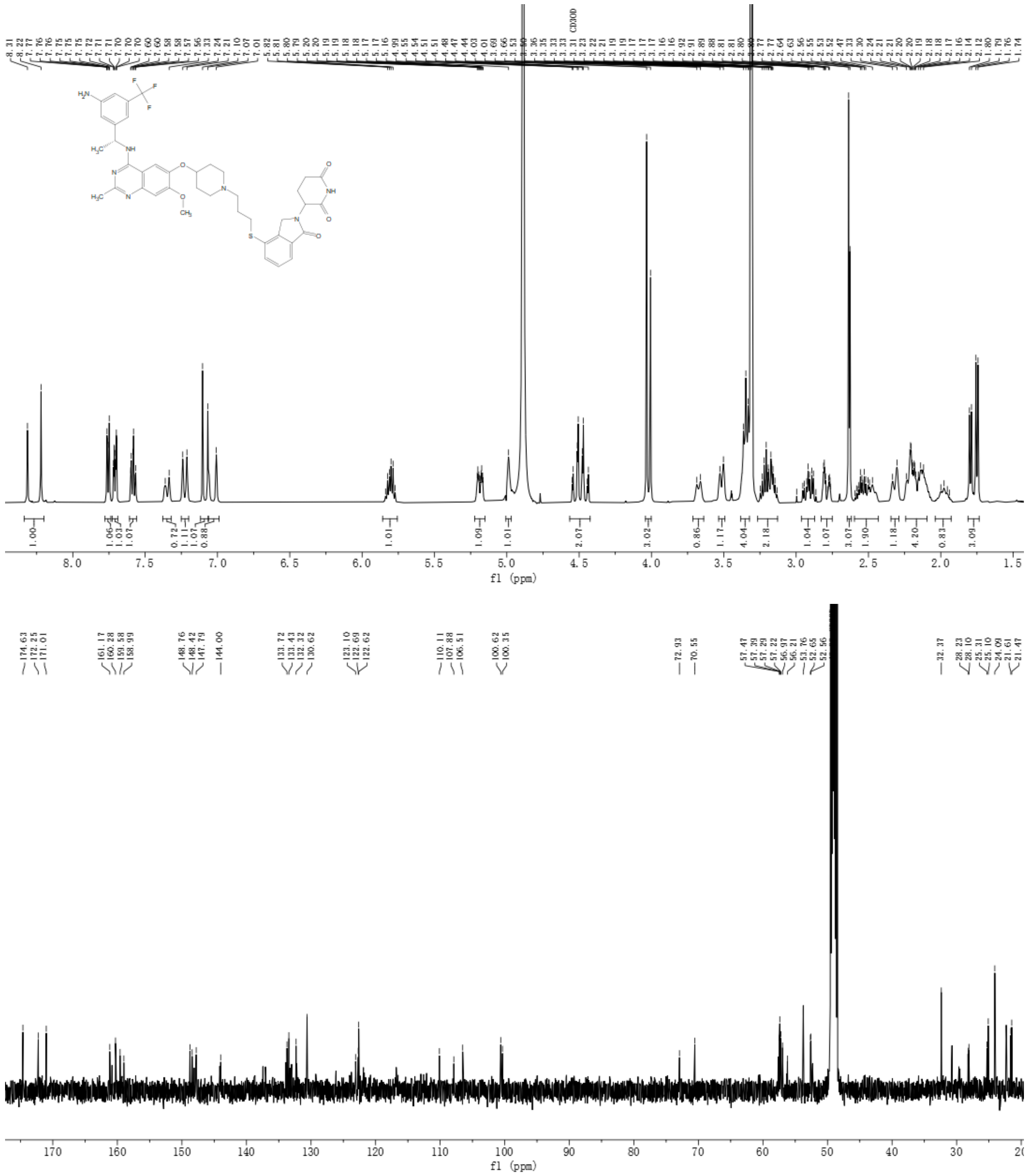

Figure S59. ^{1}H and ^{13}C spectra of **A2** in Methanol-D4.

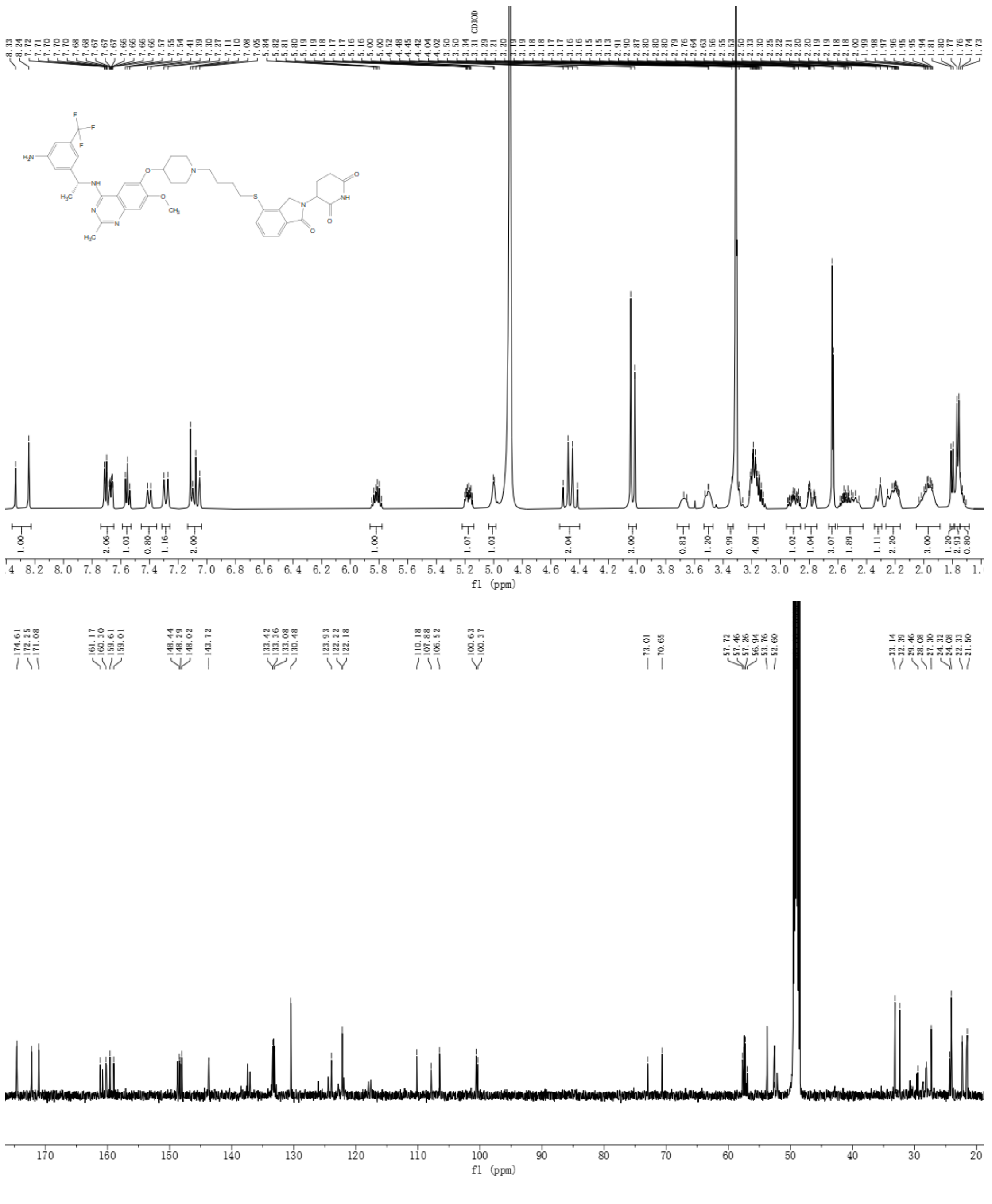

Figure S60. ^{1}H and ^{13}C spectra of **A3** in Methanol-D4.

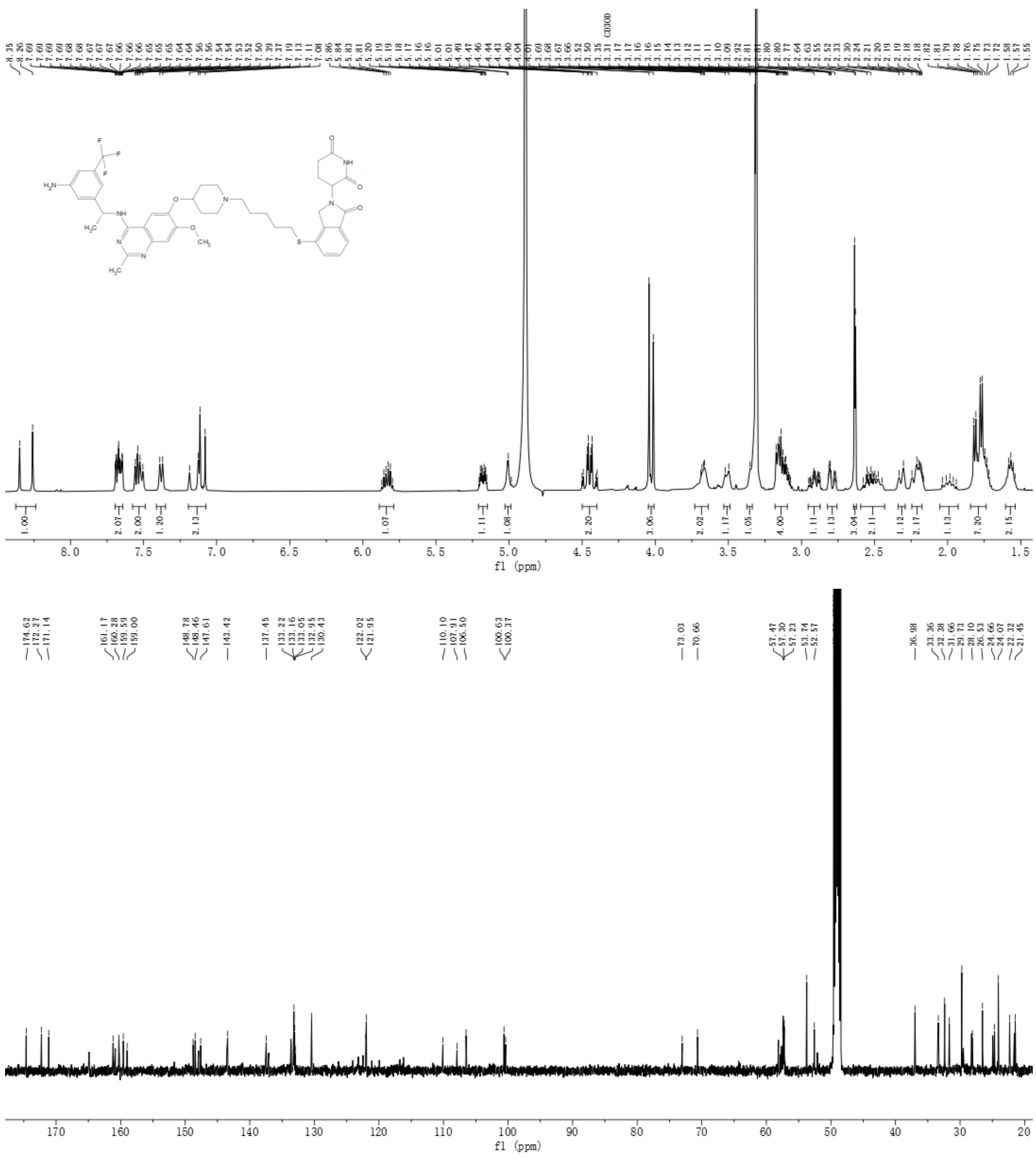

Figure S61. ^{1}H and ^{13}C spectra of **A4** in Methanol-D4.

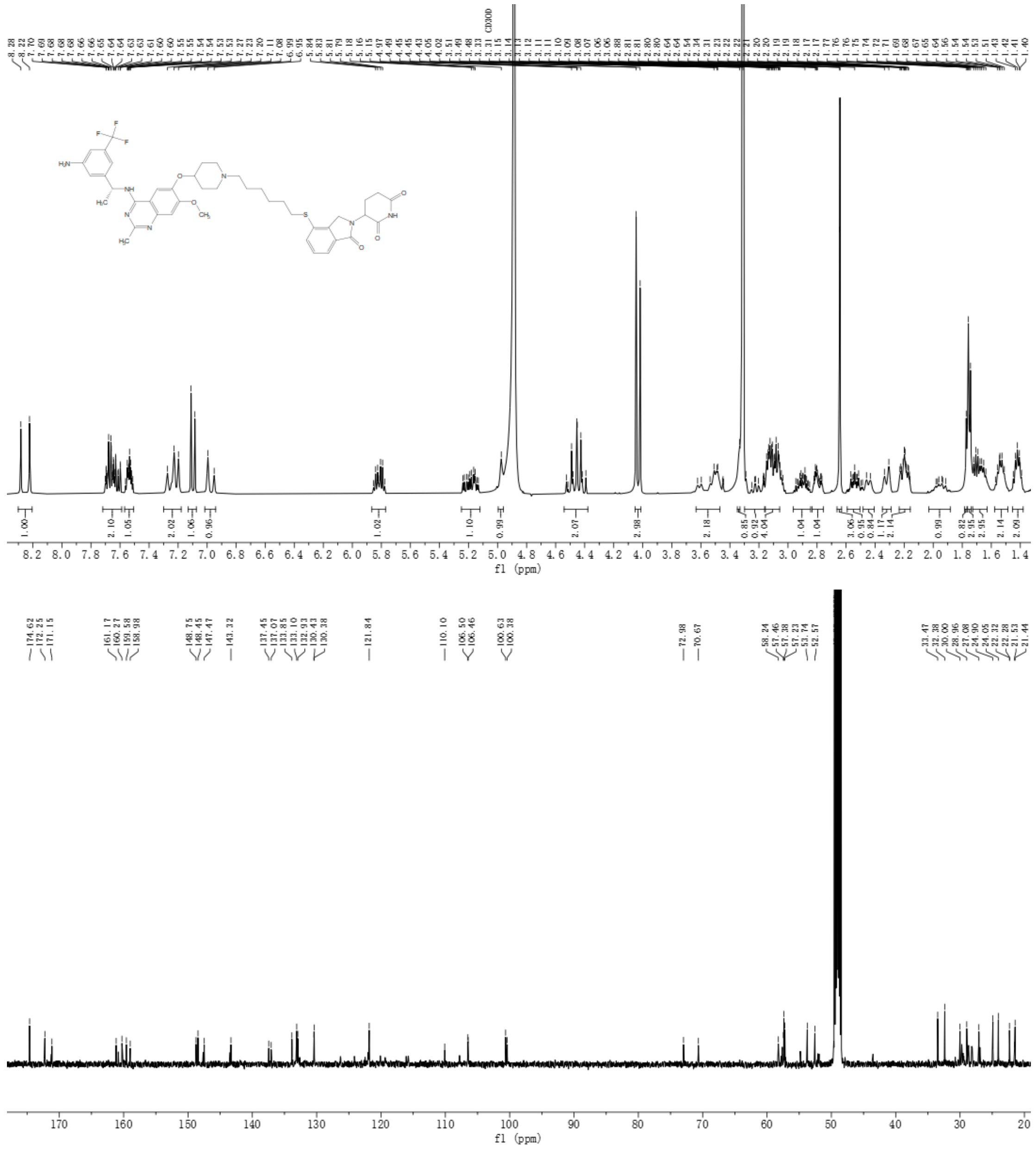

Figure S62. ^{1}H and ^{13}C spectra of **A5** in Methanol-D4.

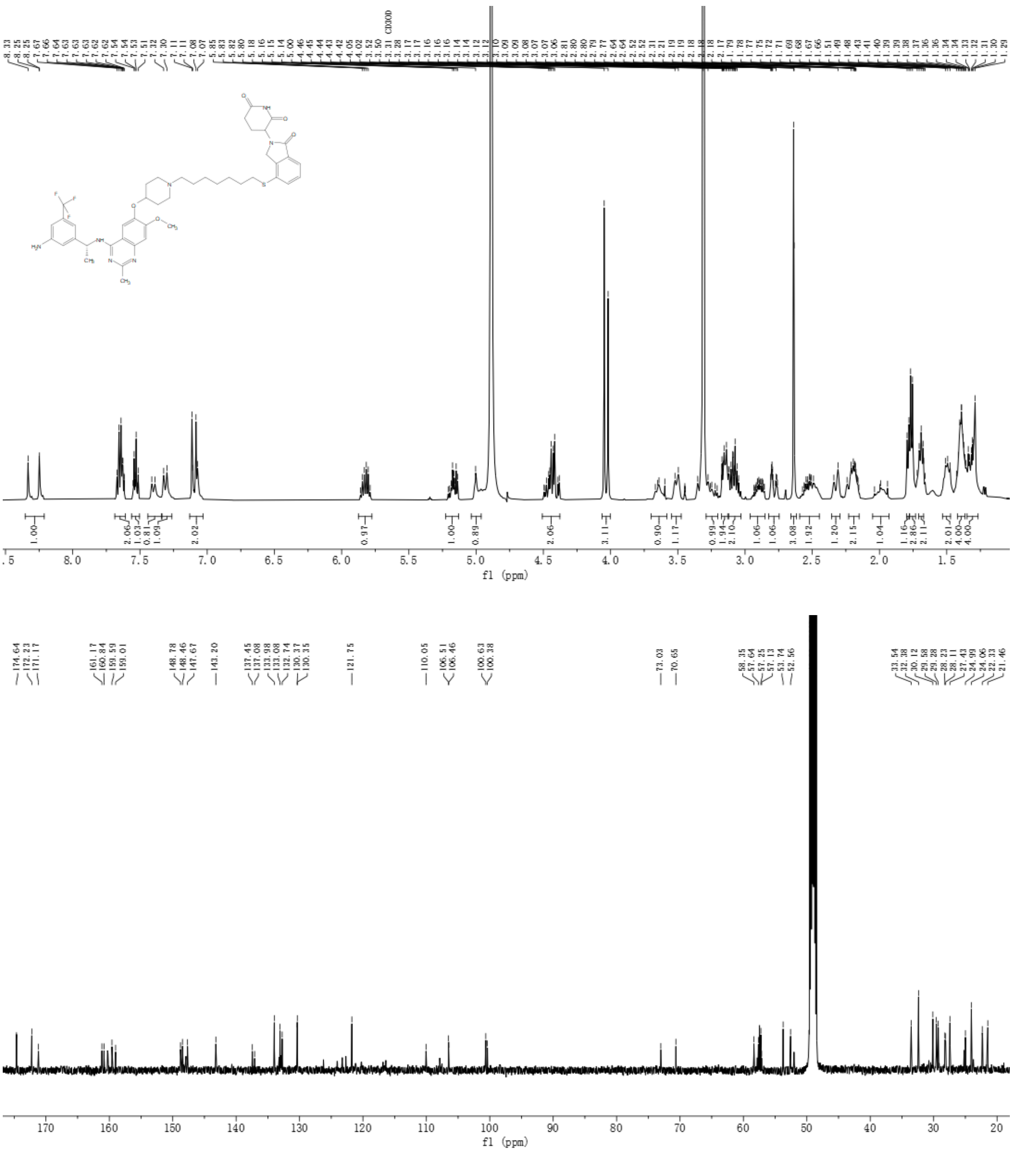

Figure S63. ^{1}H and ^{13}C spectra of **A6** in Methanol-D4.

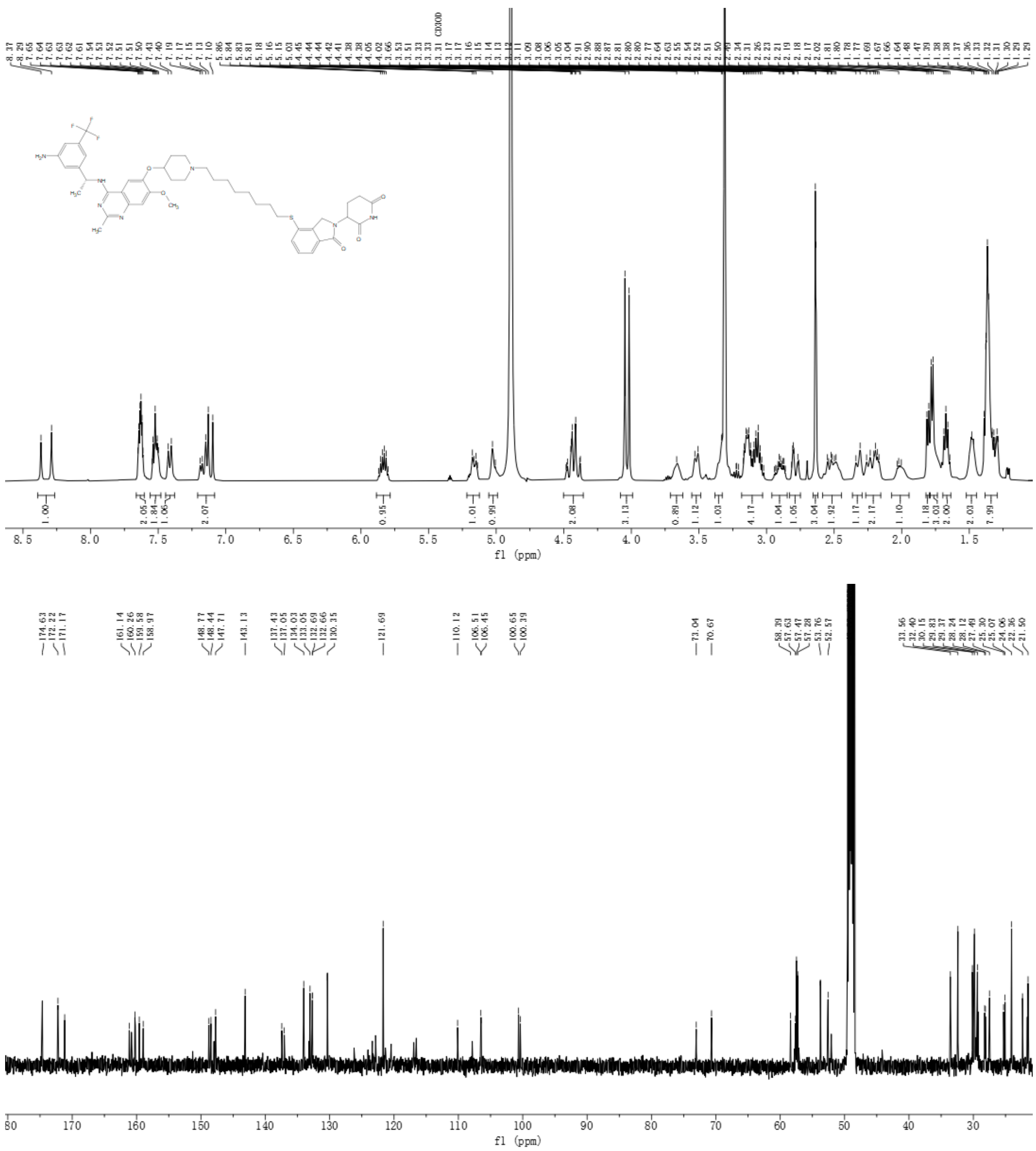

Figure S64. ^{1}H and ^{13}C spectra of **A7** in Methanol-D4.

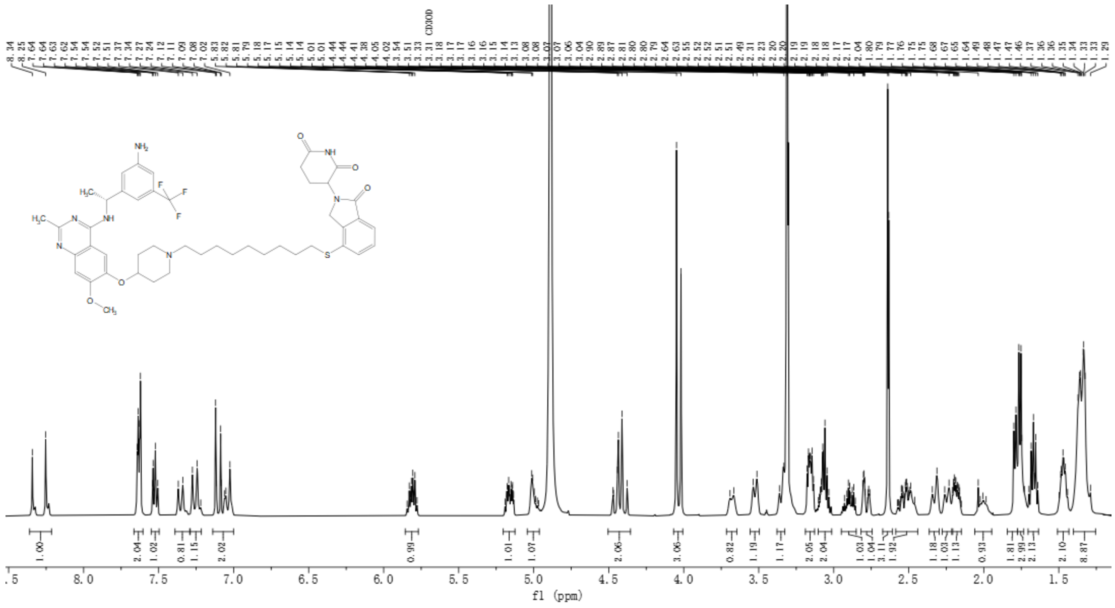

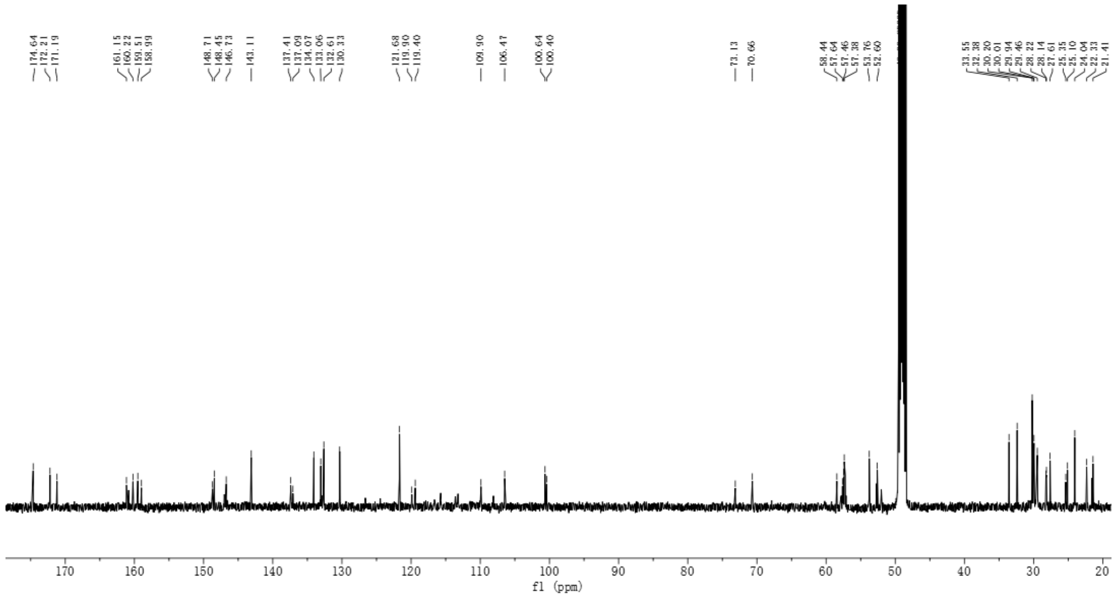

Figure S65. ^{1}H and ^{13}C spectra of **A8** in Methanol-D4.

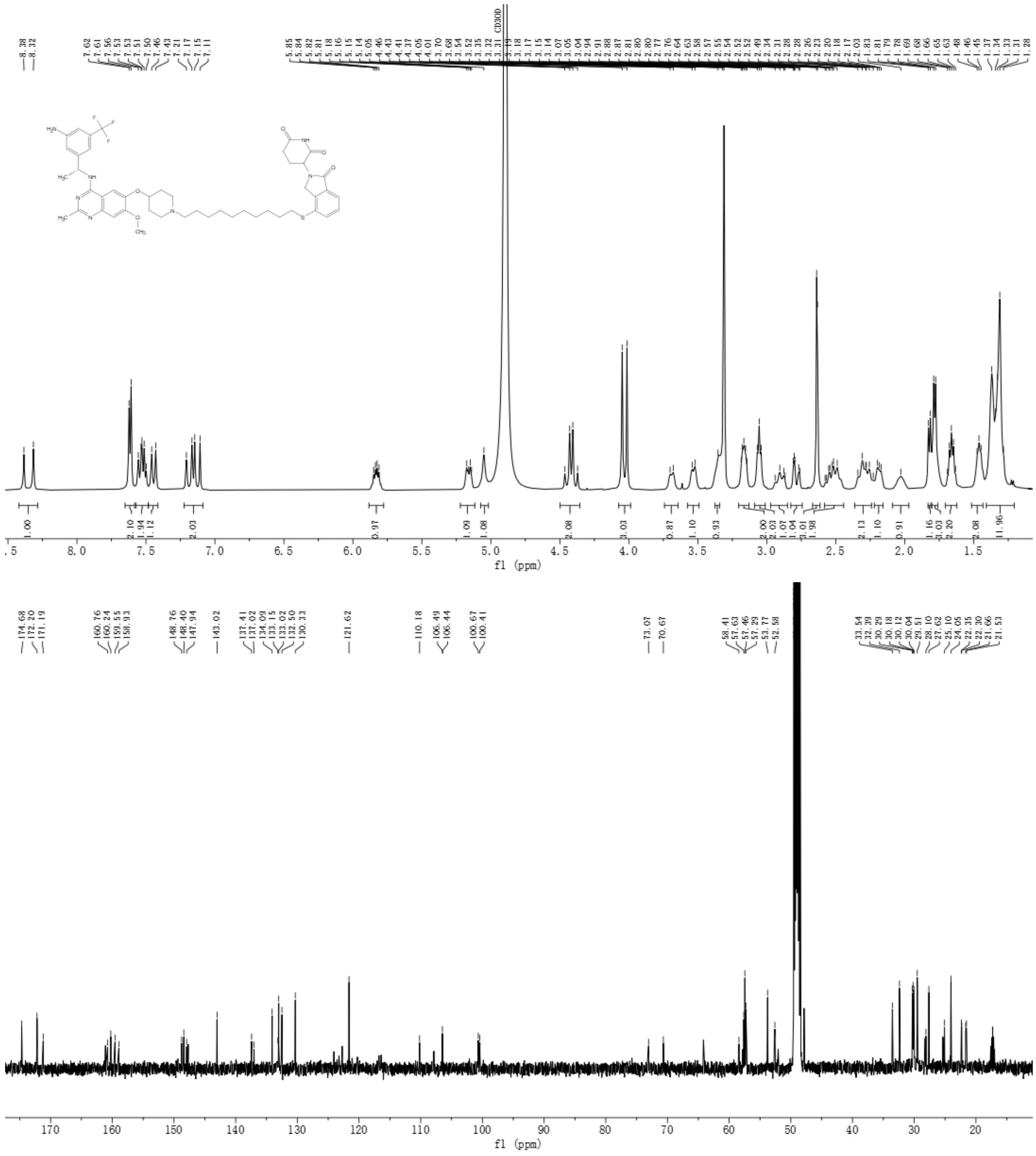

Figure S66. ^{1}H and ^{13}C spectra of **A9** in Methanol-D4.

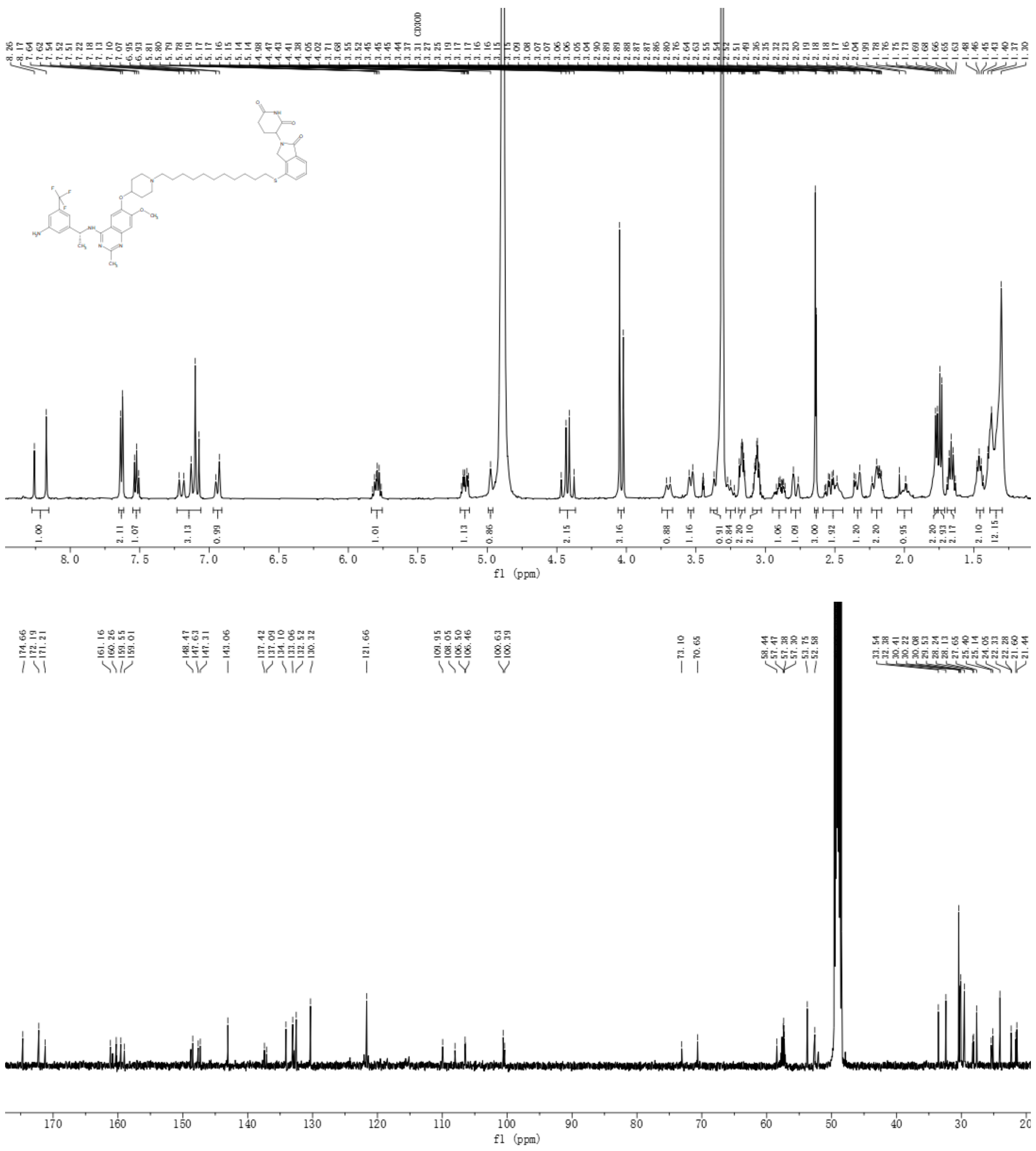

Figure S67. ^{1}H and ^{13}C spectra of **A10** in Methanol-D4.

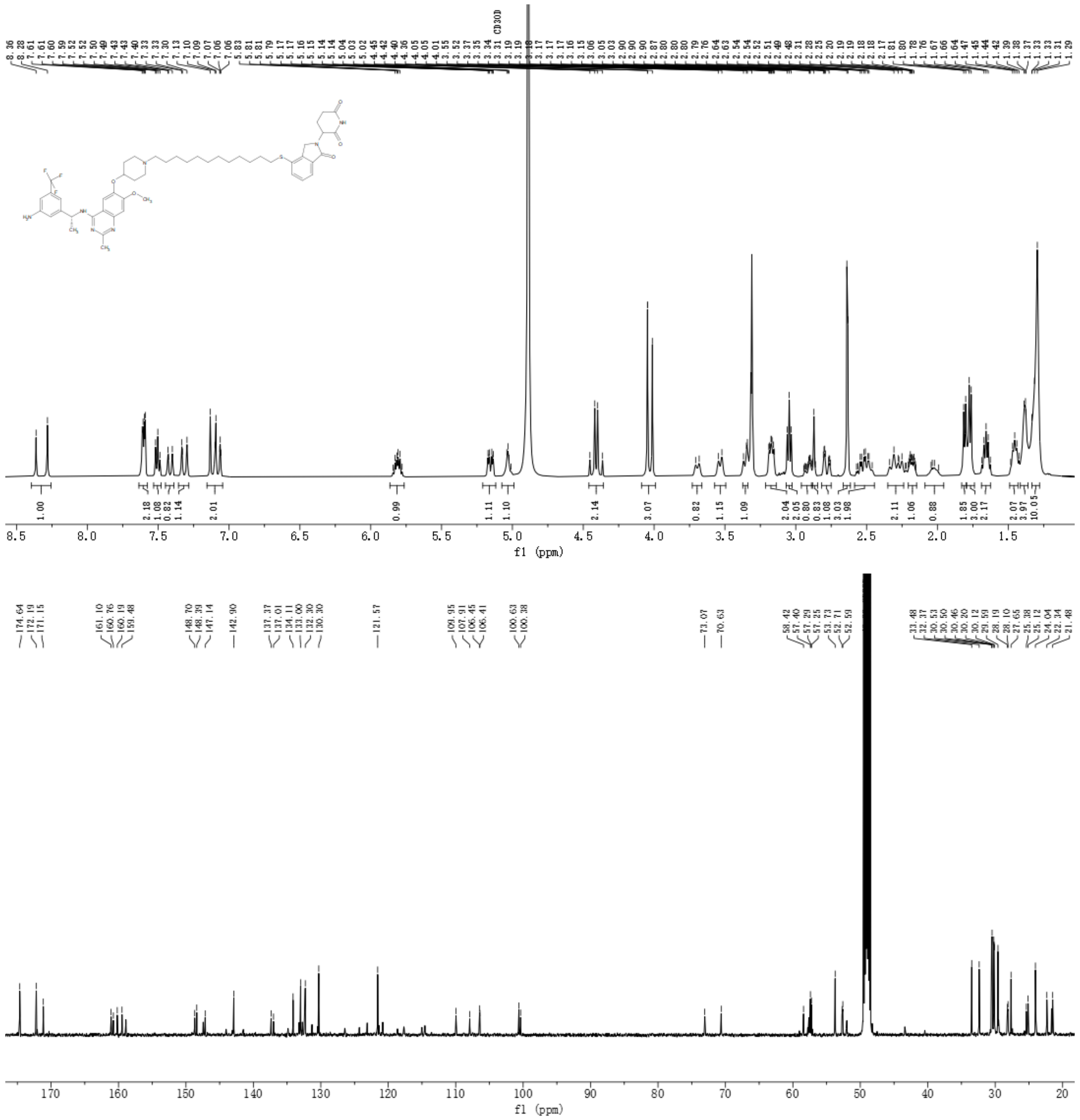

Figure S68. ^{1}H and ^{13}C spectra of **A11** in Methanol-D4.

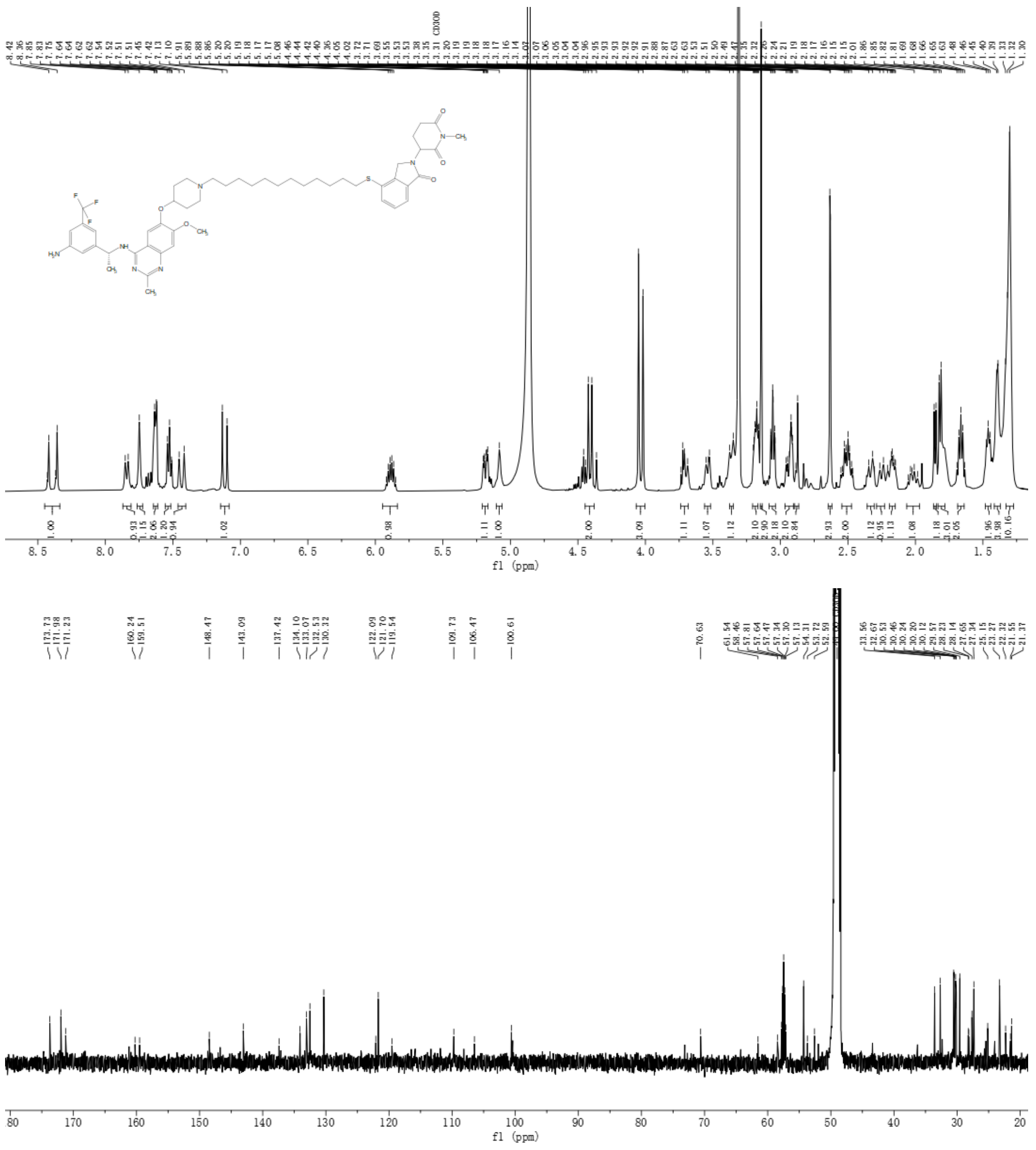

Figure S69. ^{1}H and ^{13}C spectra of **A11-NC** in Methanol-D4.

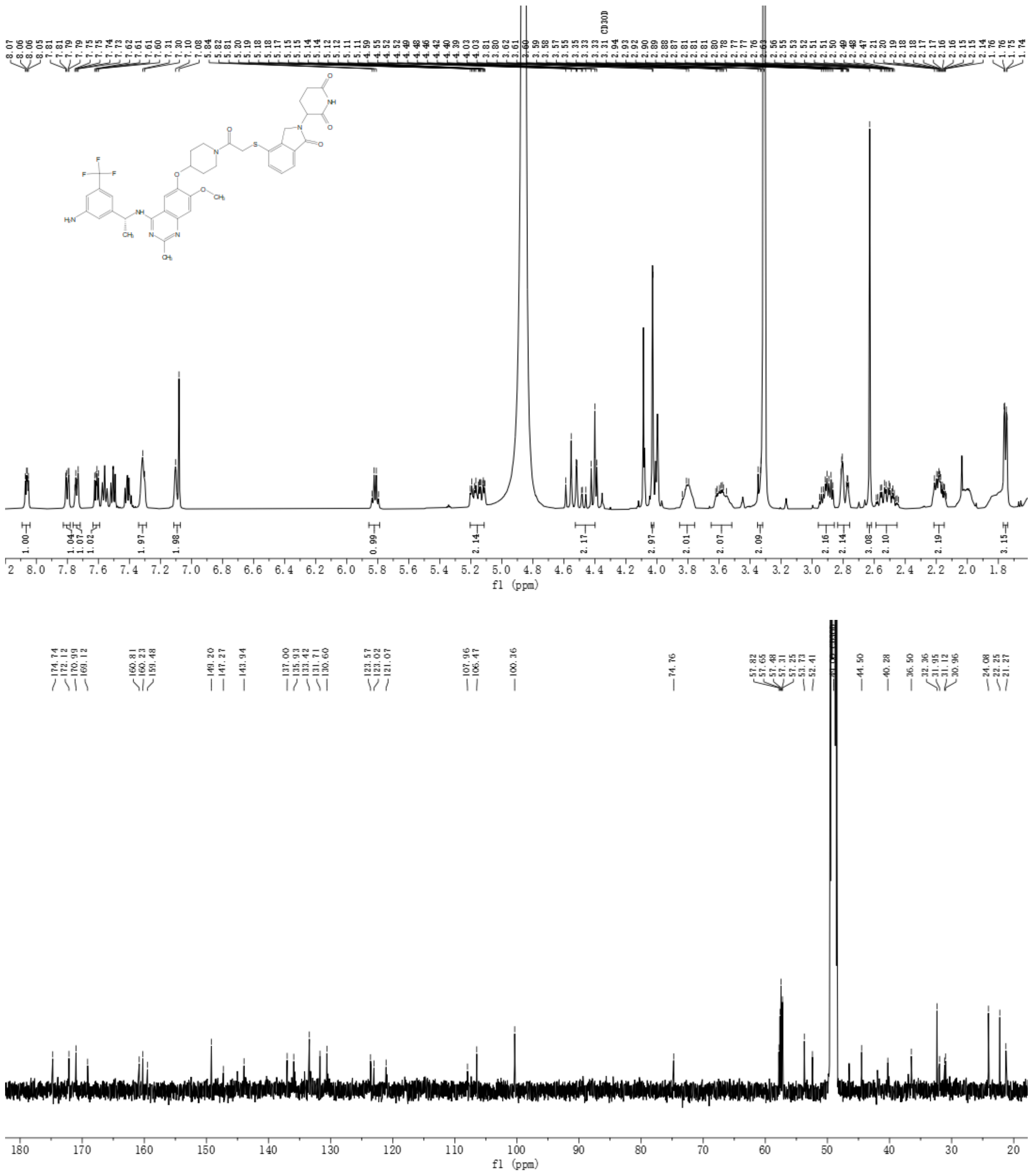

Figure S70. ^{1}H and ^{13}C spectra of **B1** in Methanol-D4.

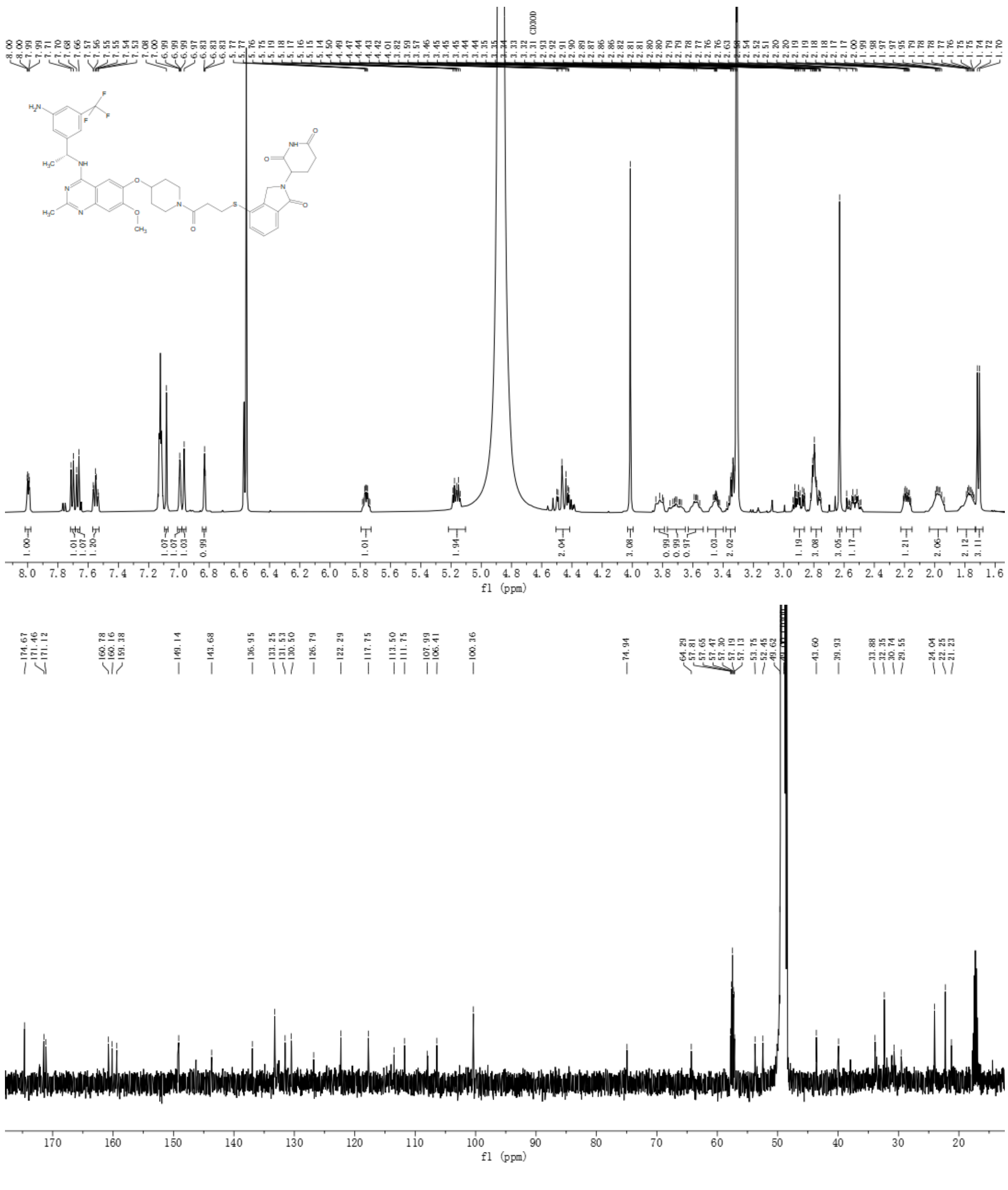

Figure S71. ^{1}H and ^{13}C spectra of **B2** in Methanol-D4.

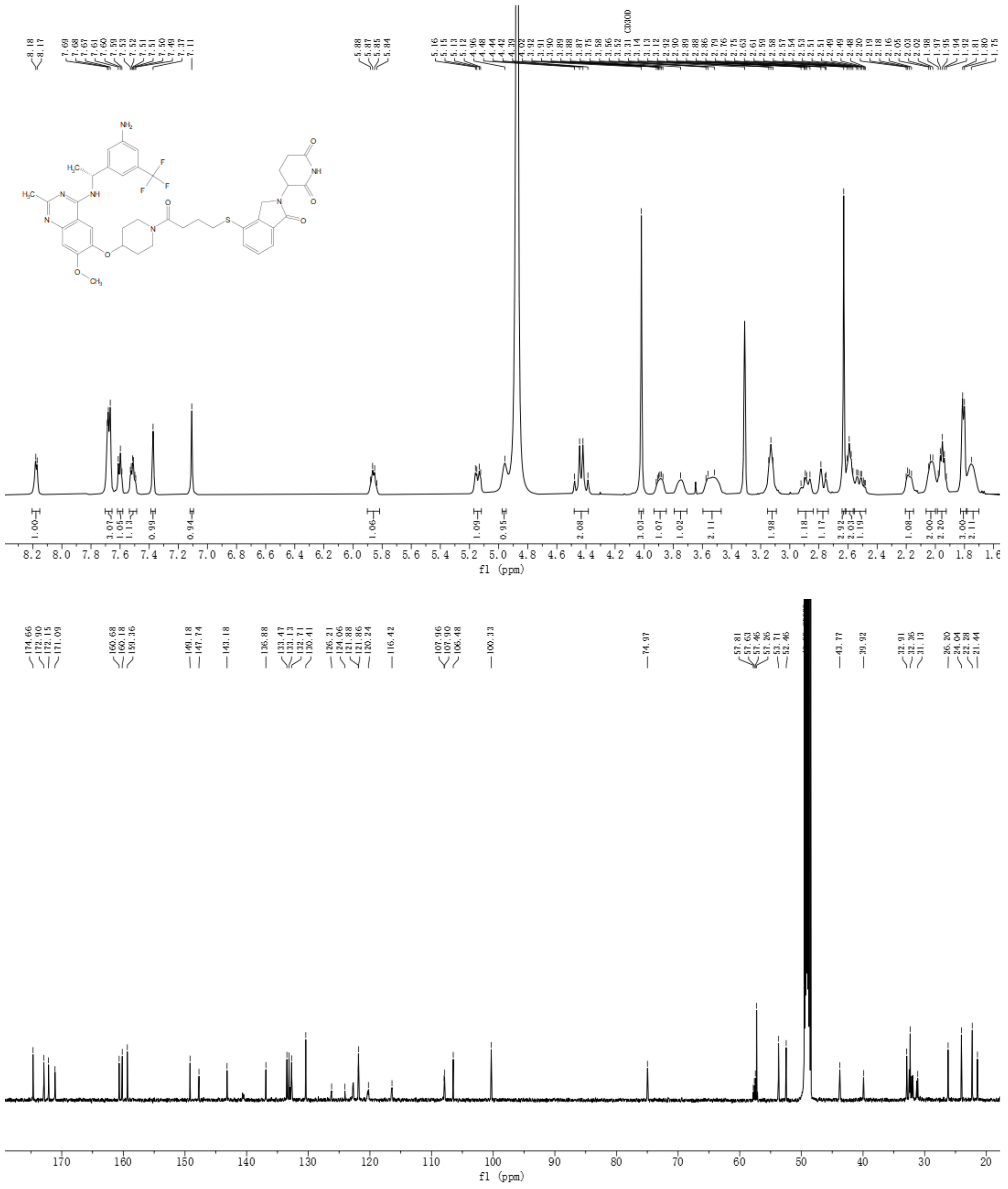

Figure S72. ^{1}H and ^{13}C spectra of **B3** in Methanol-D4.

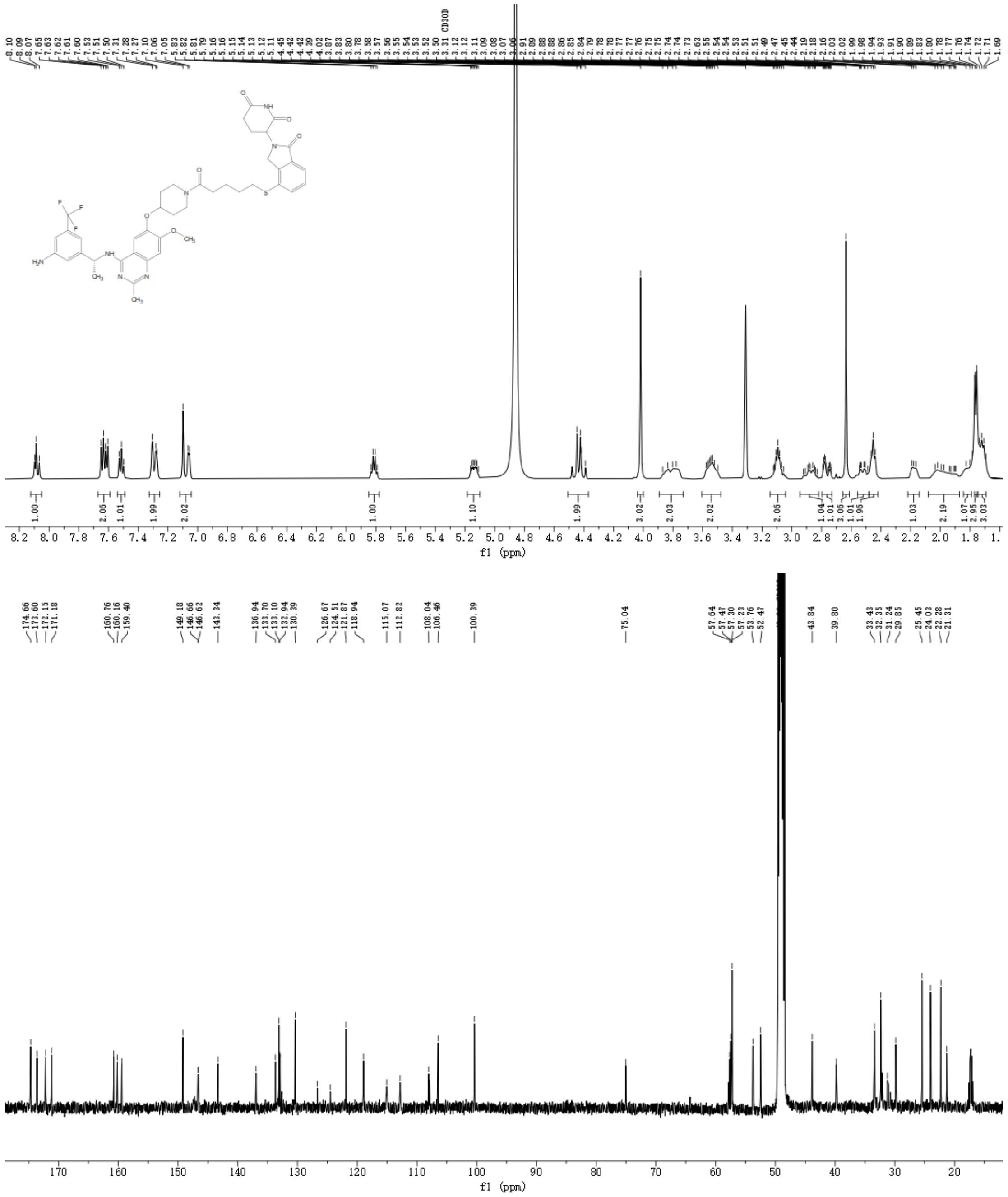

Figure S73. ^{1}H and ^{13}C spectra of **B4** in Methanol-D4.

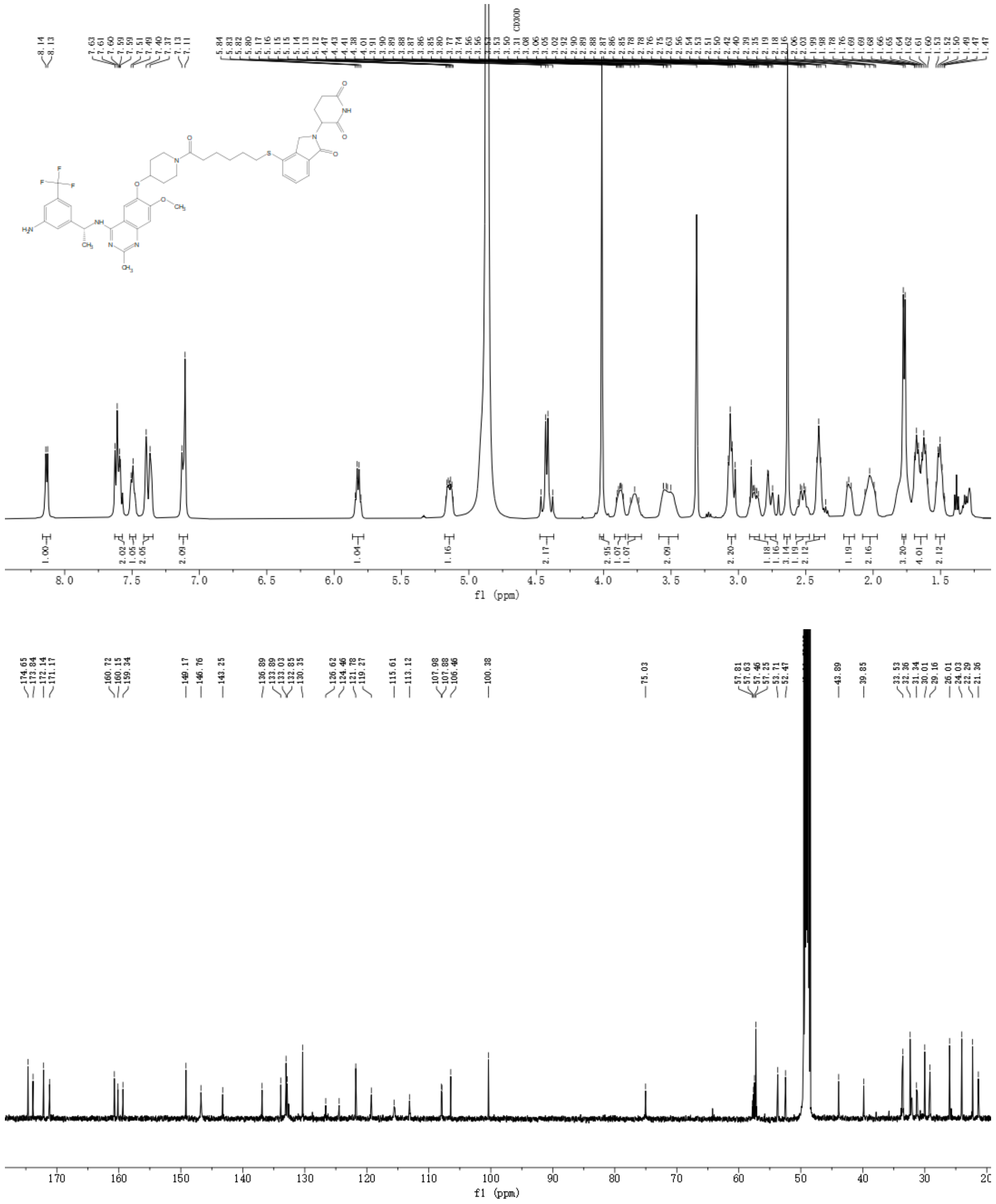

Figure S74. ^{1}H and ^{13}C spectra of **B5** in Methanol-D4.

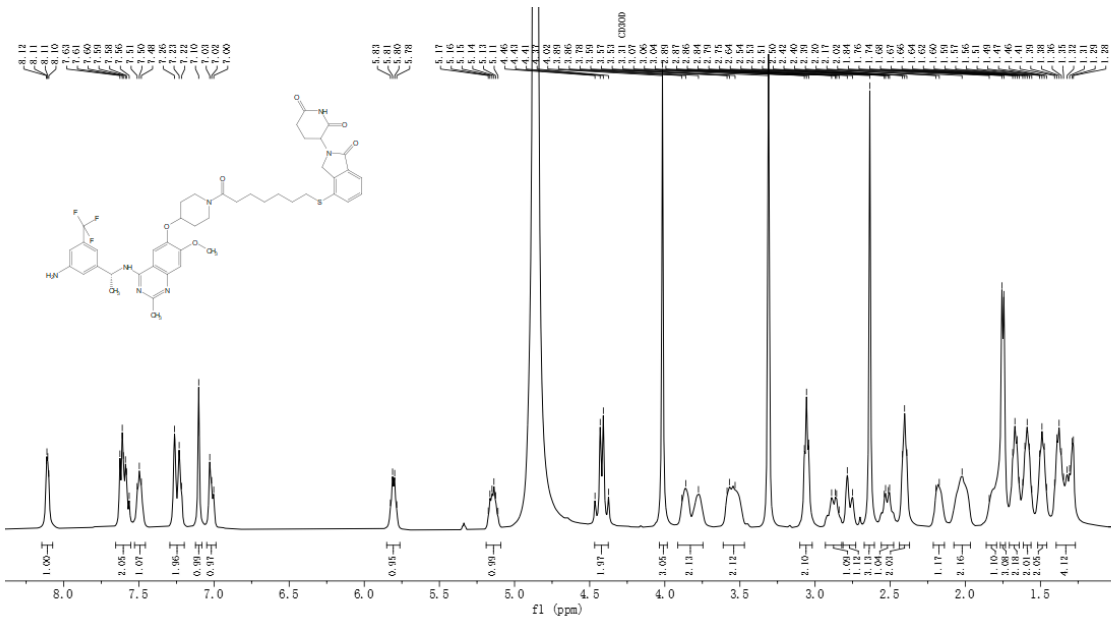

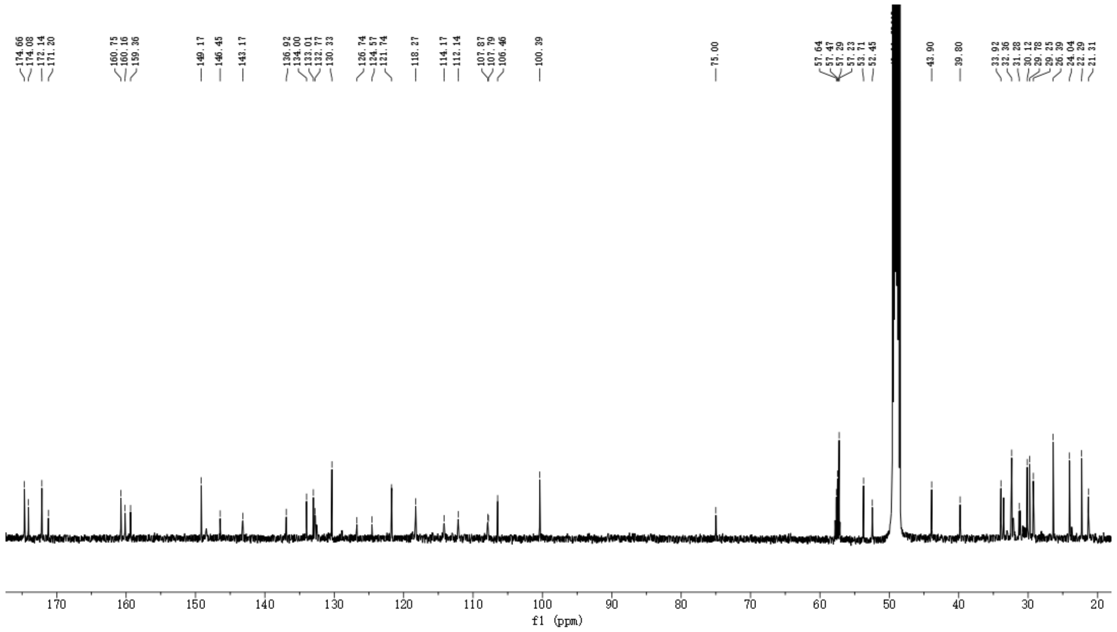

Figure S75. ^{1}H and ^{13}C spectra of **B6** in Methanol-D4.

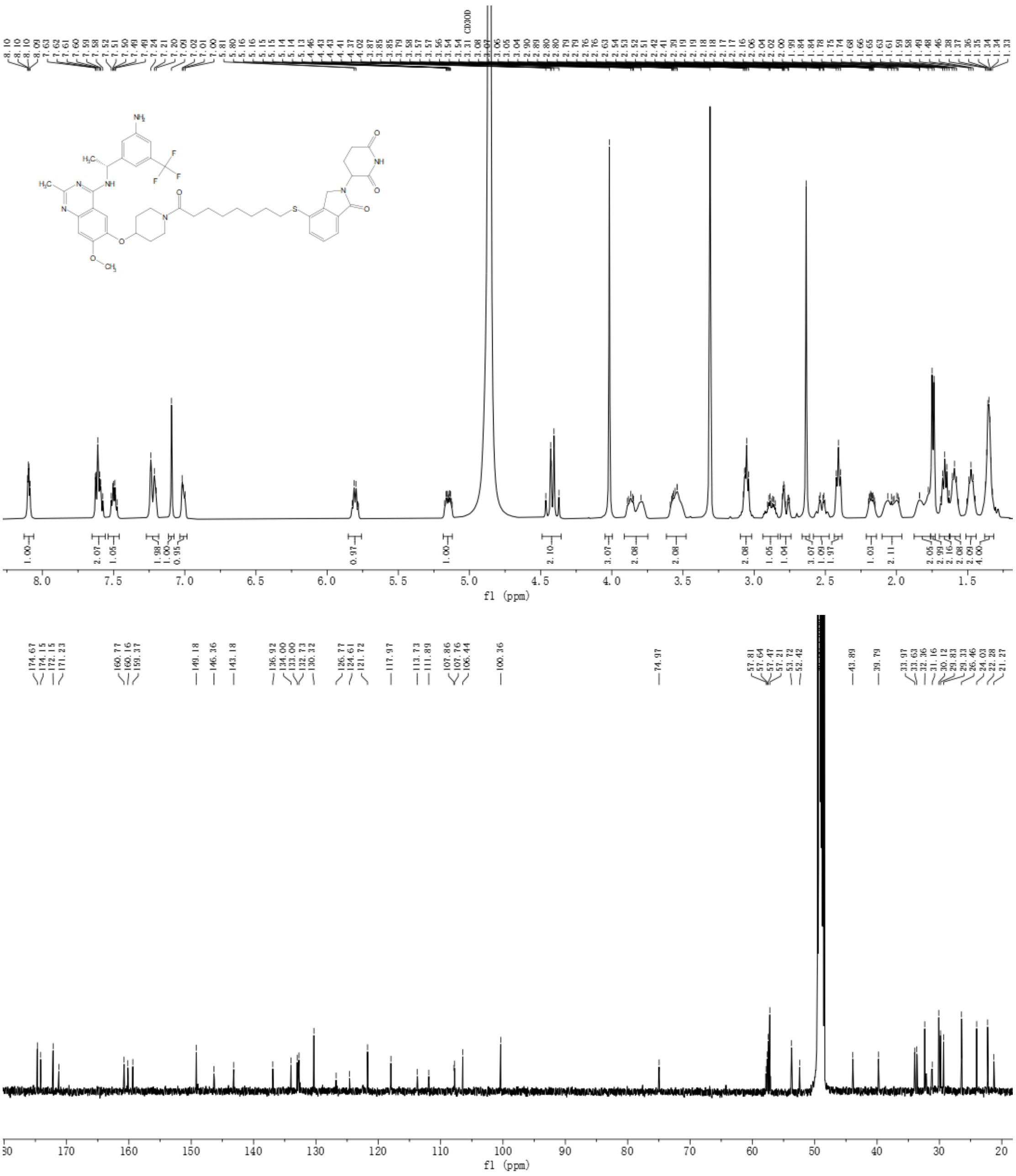

Figure S76. ^{1}H and ^{13}C spectra of **B7** in Methanol-D4.

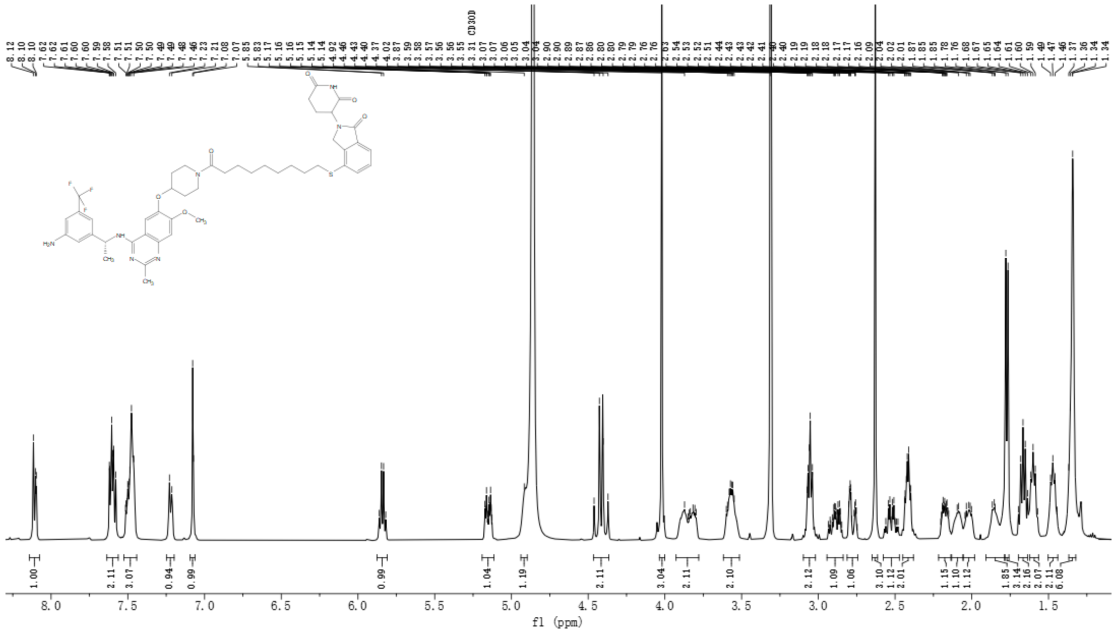

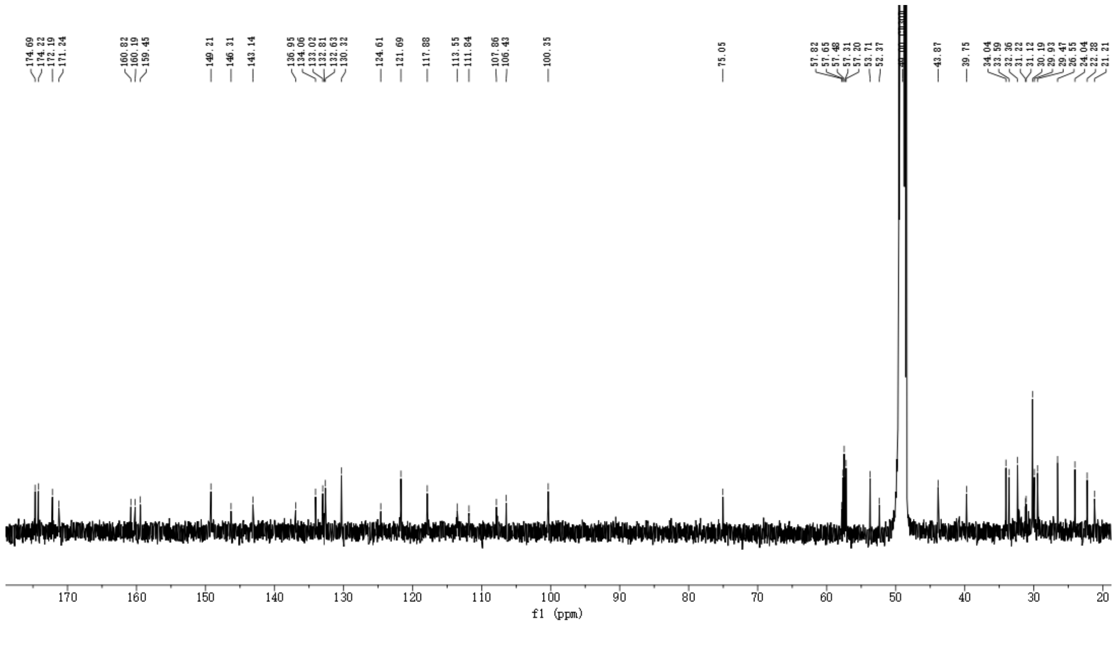

Figure S77. ^{1}H and ^{13}C spectra of **B8** in Methanol-D4.

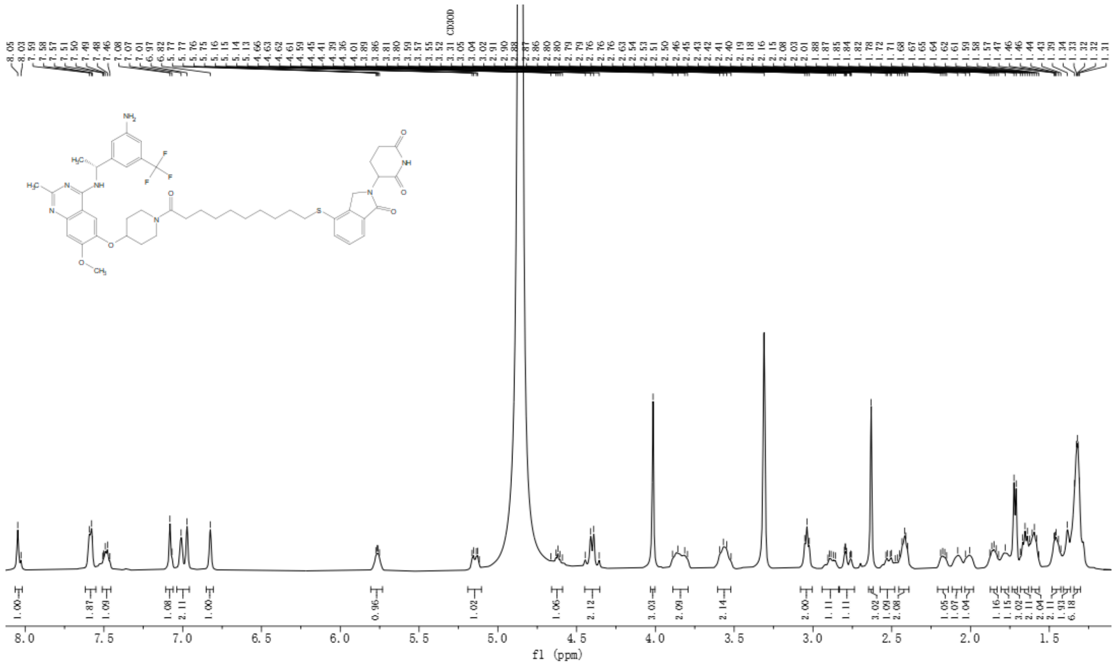

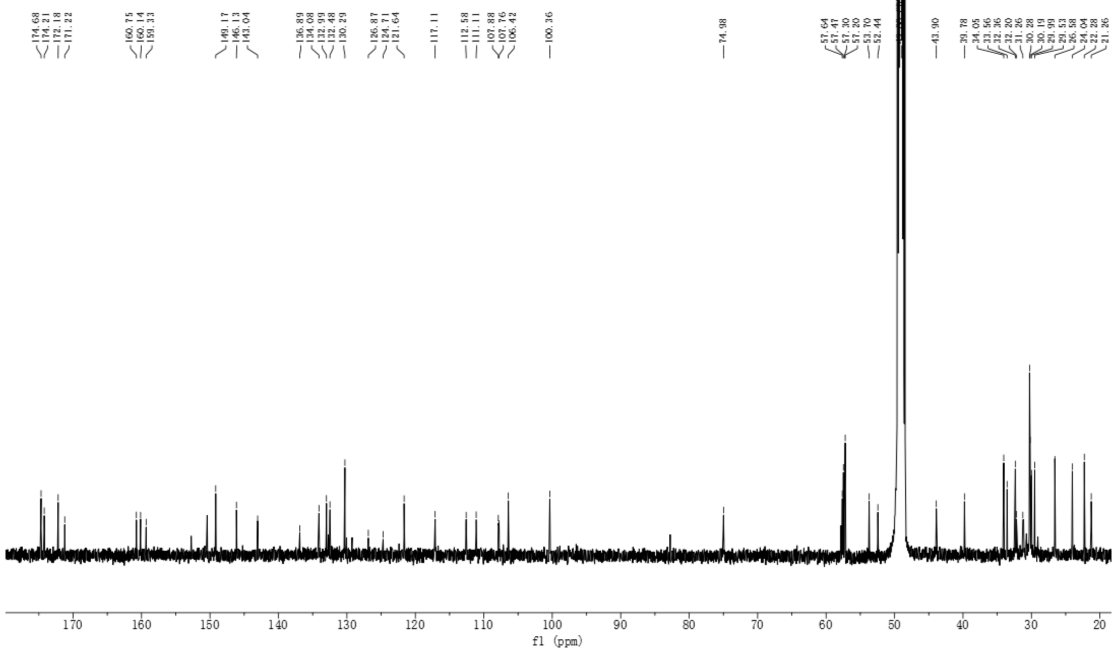

Figure S78. ^{1}H and ^{13}C spectra of **B9** in Methanol-D4.

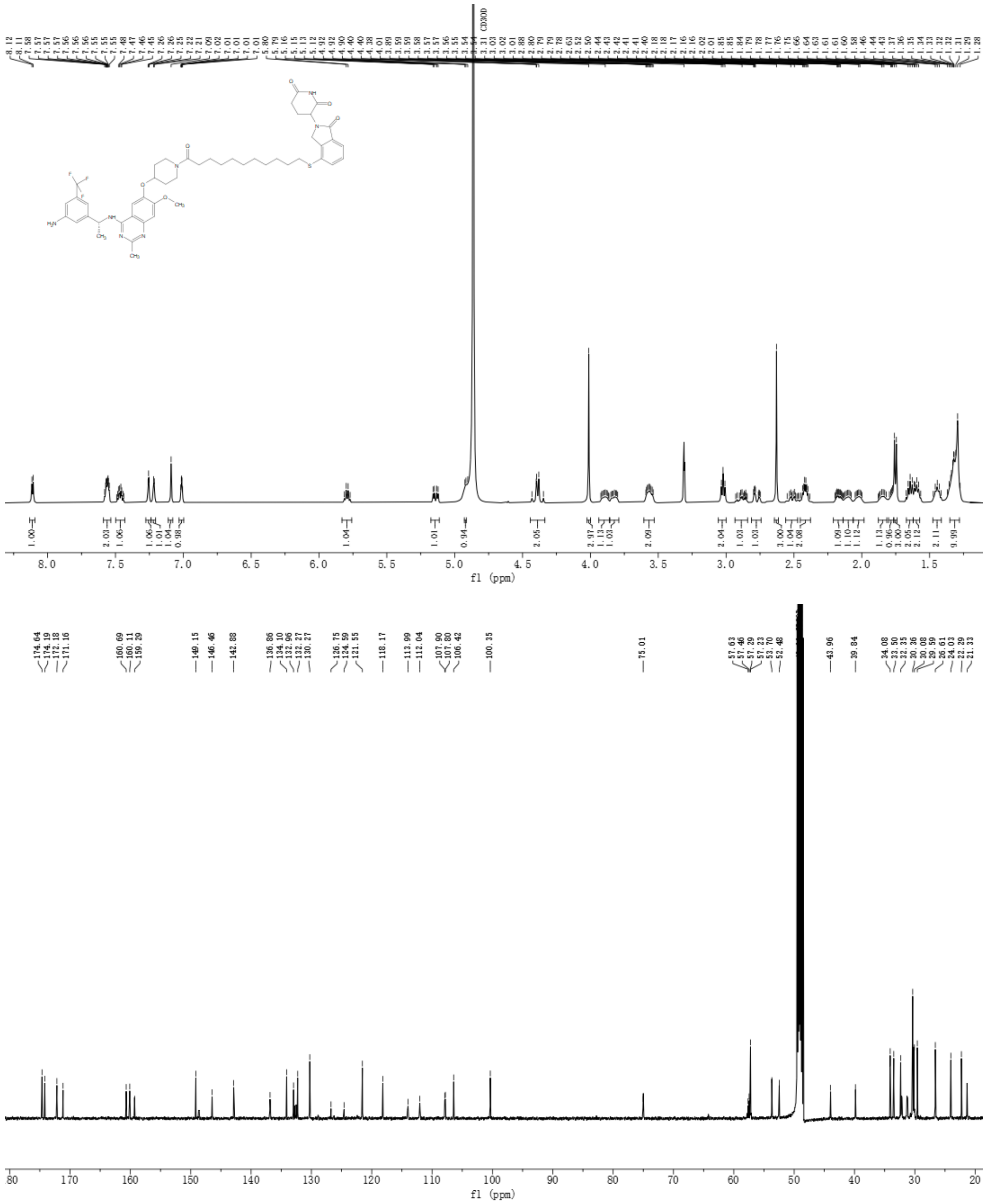

Figure S79. ^{1}H and ^{13}C spectra of **B10** in Methanol-D4.

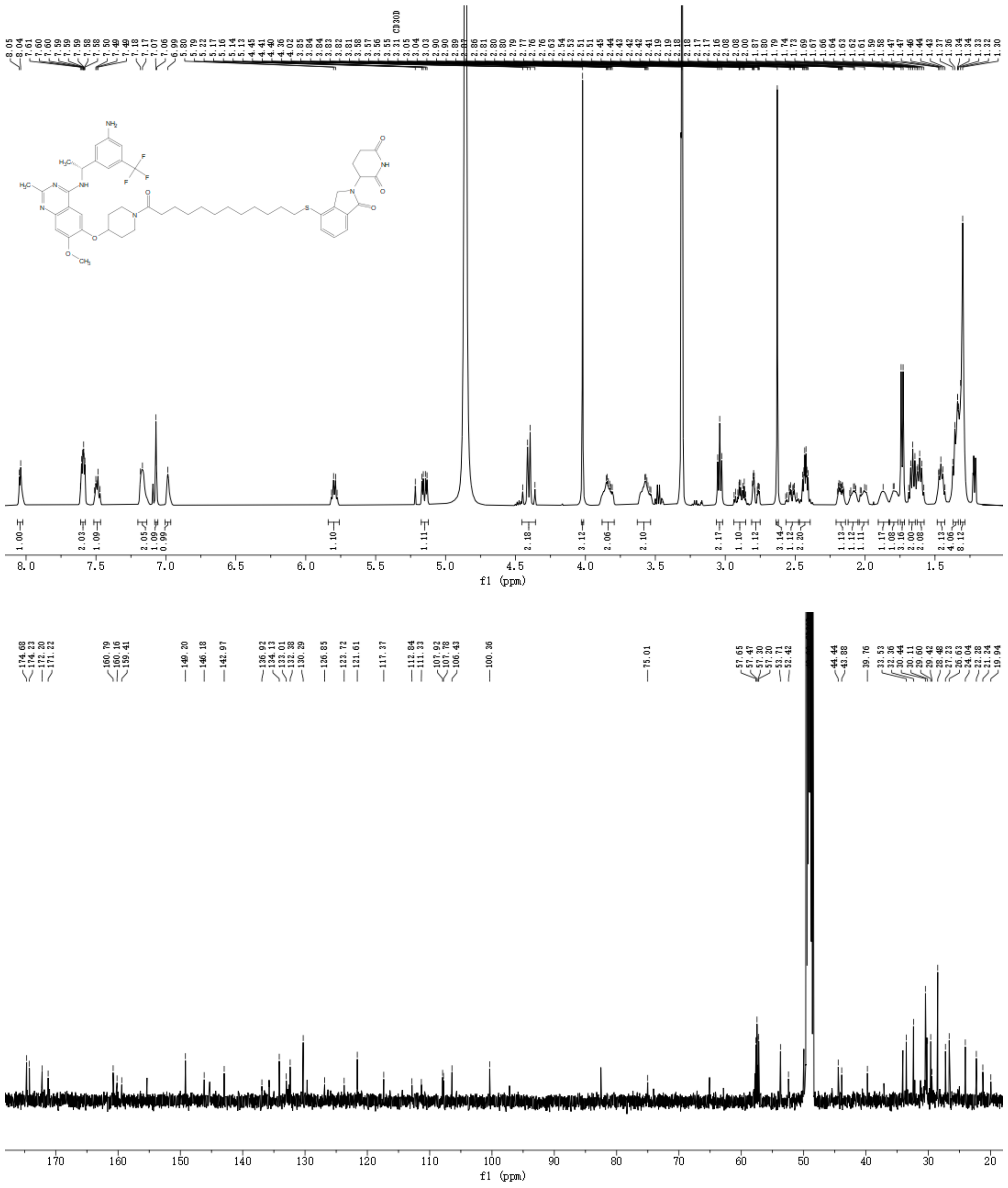

Figure S80. ^{1}H and ^{13}C spectra of **B11** in Methanol-D4.

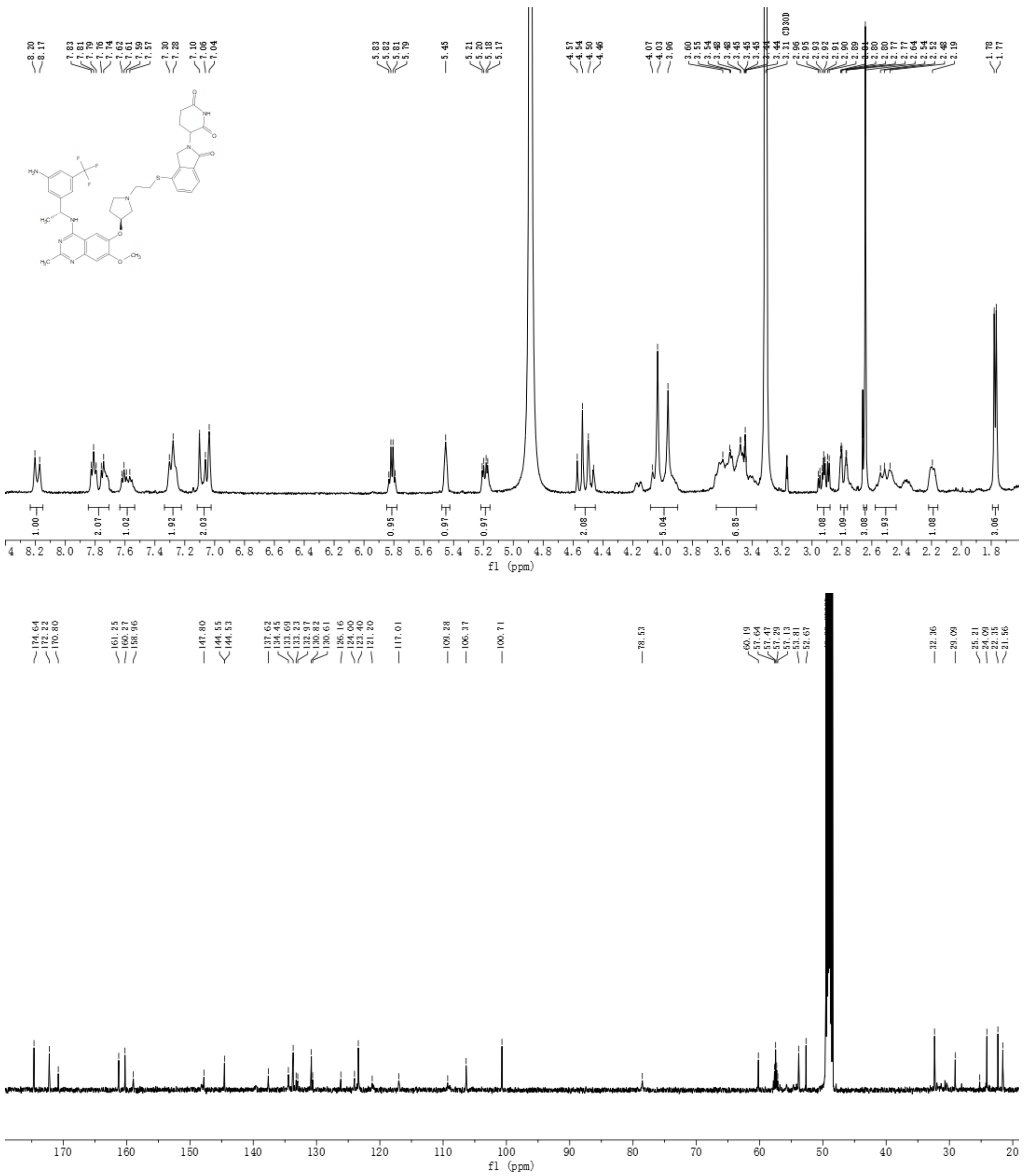

Figure S81. ^{1}H and ^{13}C spectra of **C1** in Methanol-D4.

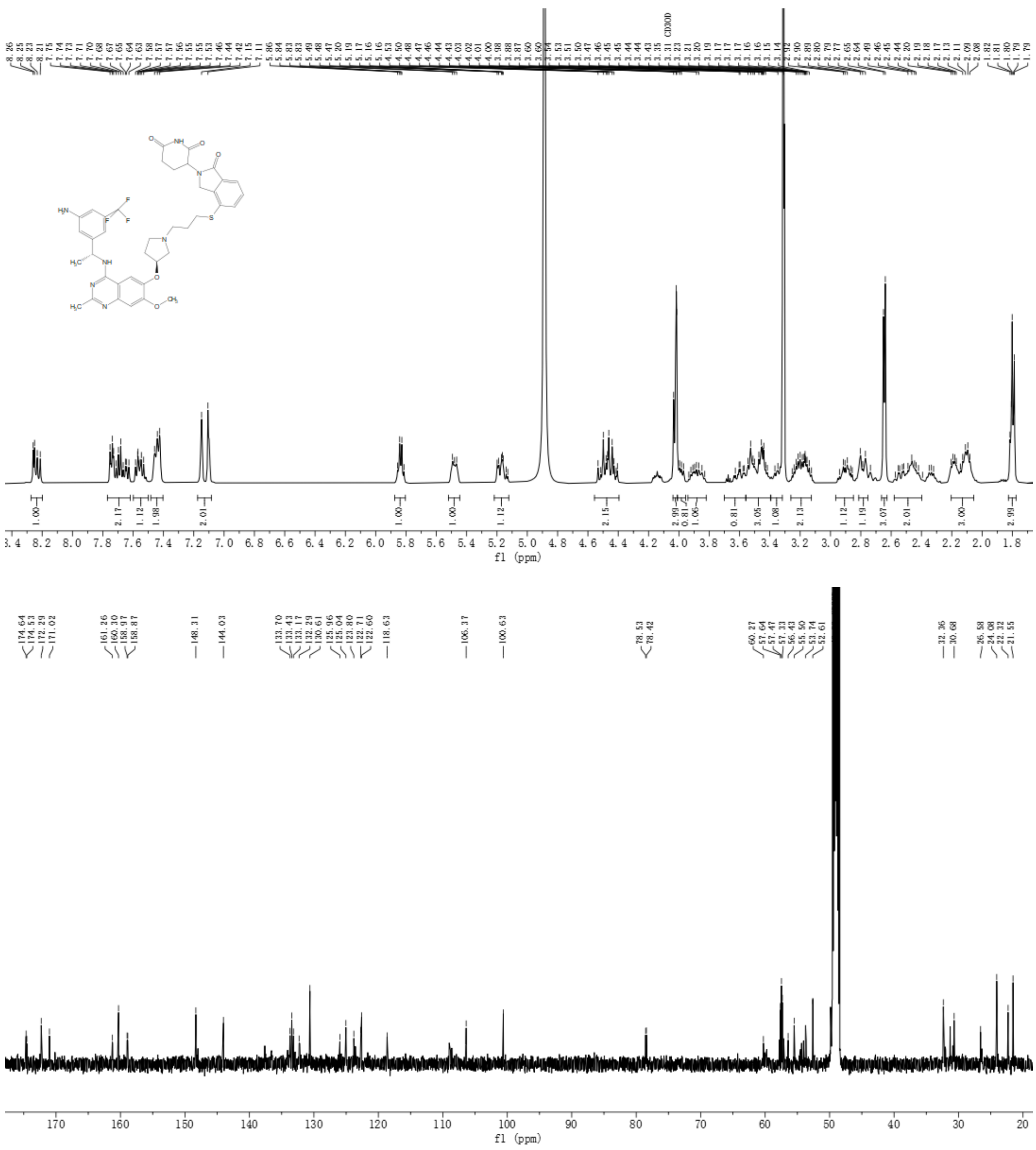

Figure S82. ^{1}H and ^{13}C spectra of **C2** in Methanol-D4.

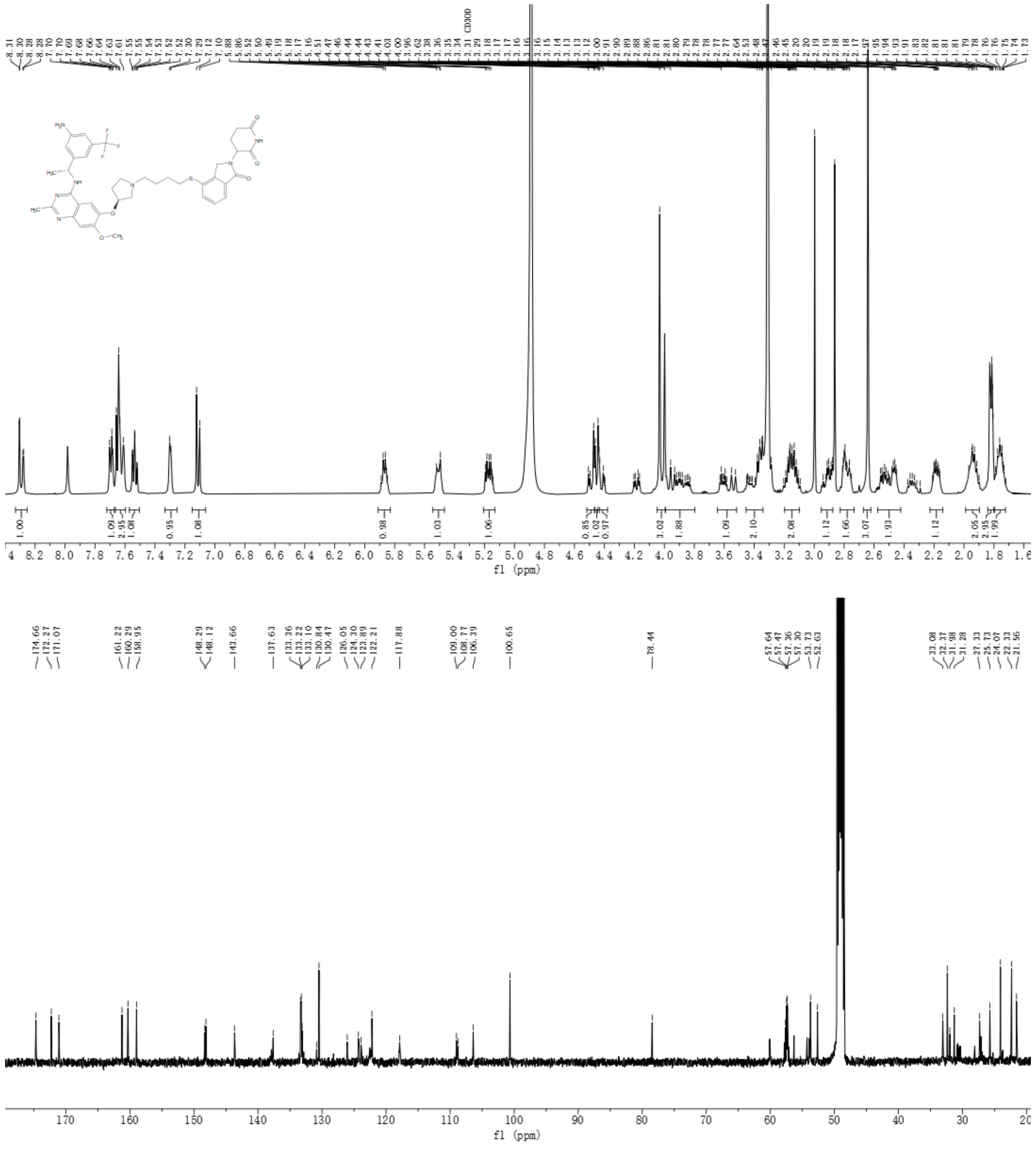

Figure S83. ^{1}H and ^{13}C spectra of **C3** in Methanol-D4.

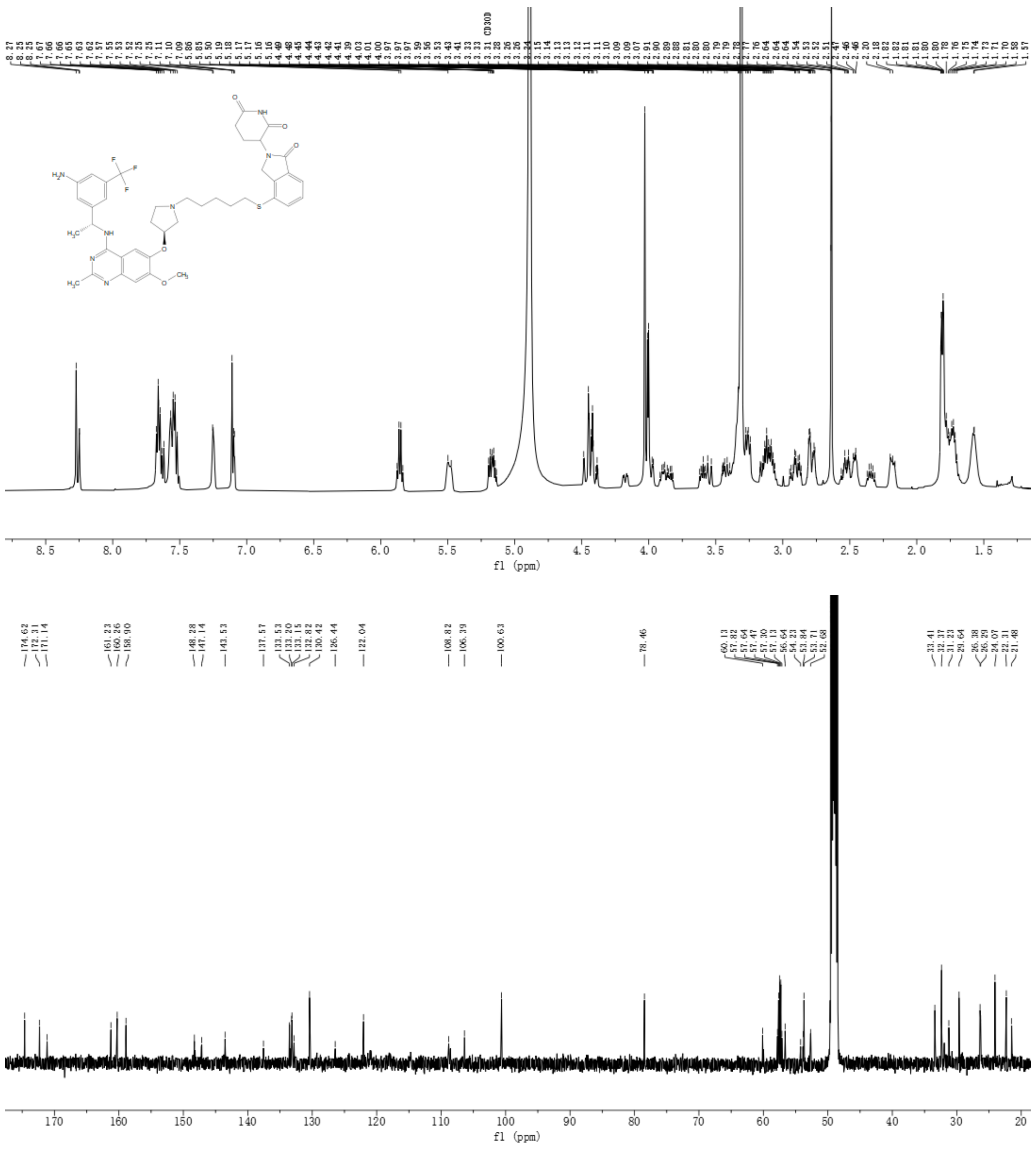

Figure S84. ^{1}H and ^{13}C spectra of **C4** in Methanol-D4.

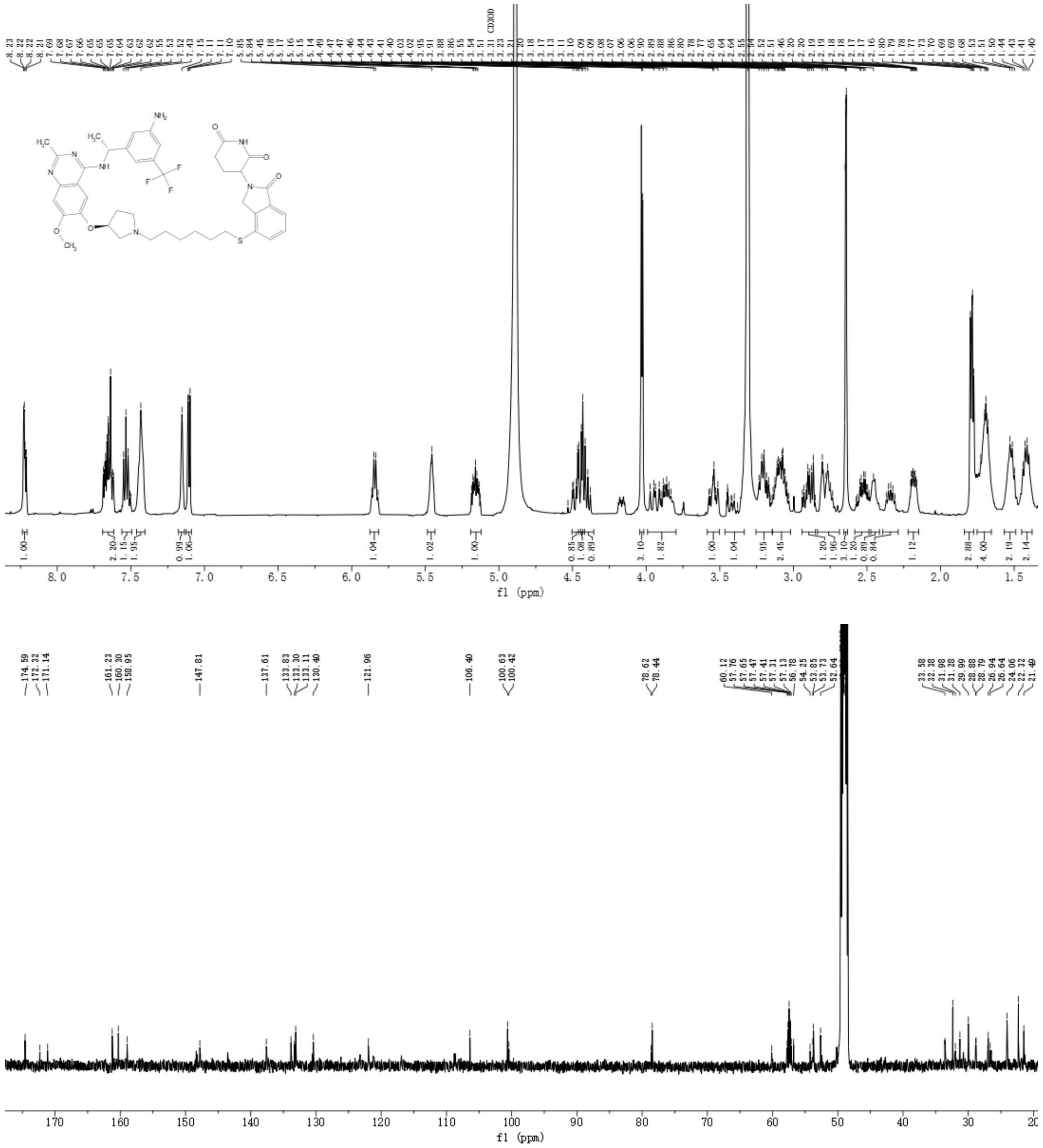

Figure S85. ^{1}H and ^{13}C spectra of **C5** in Methanol-D4.

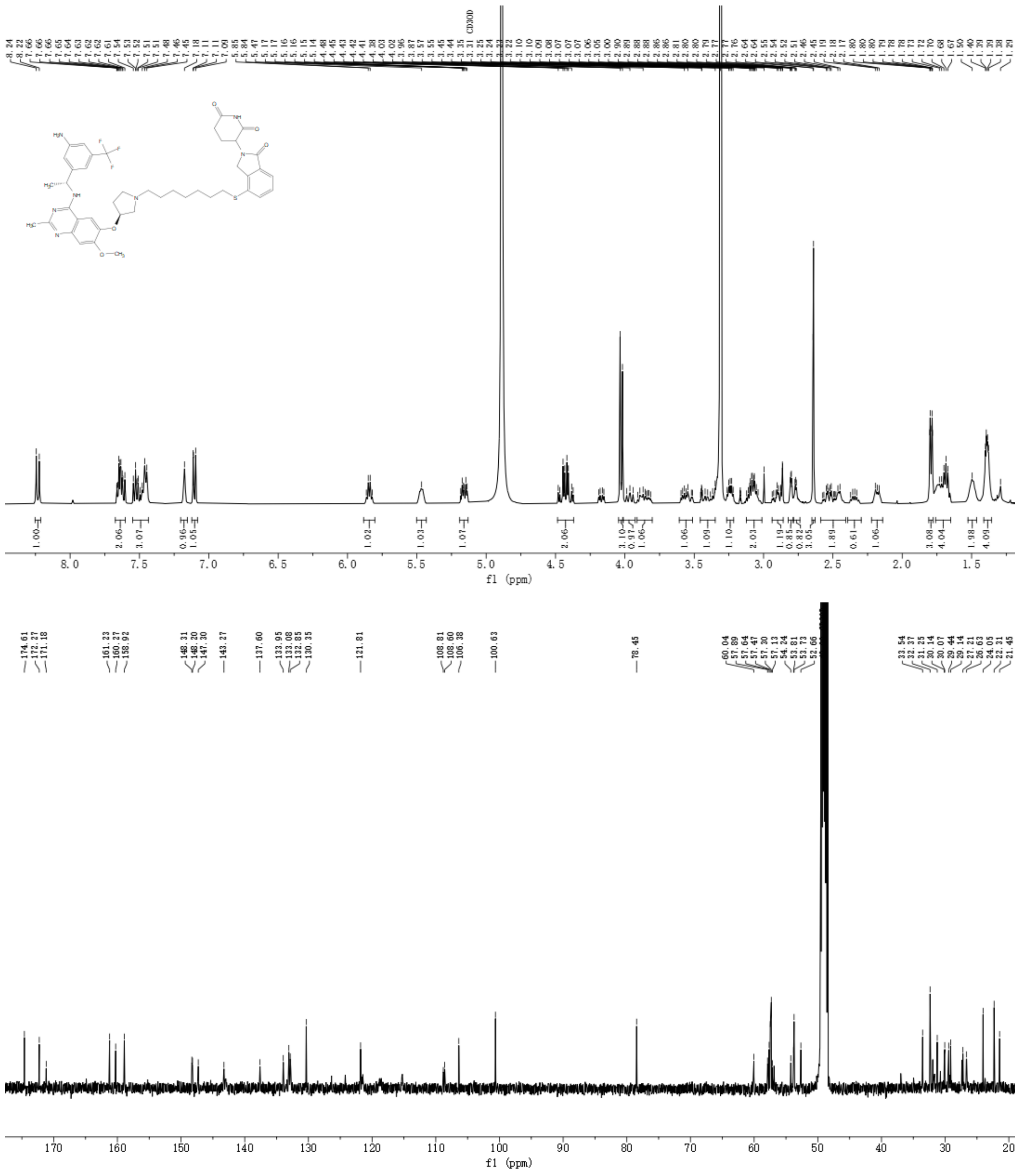

Figure S86. ^{1}H and ^{13}C spectra of **C6** in Methanol-D4.

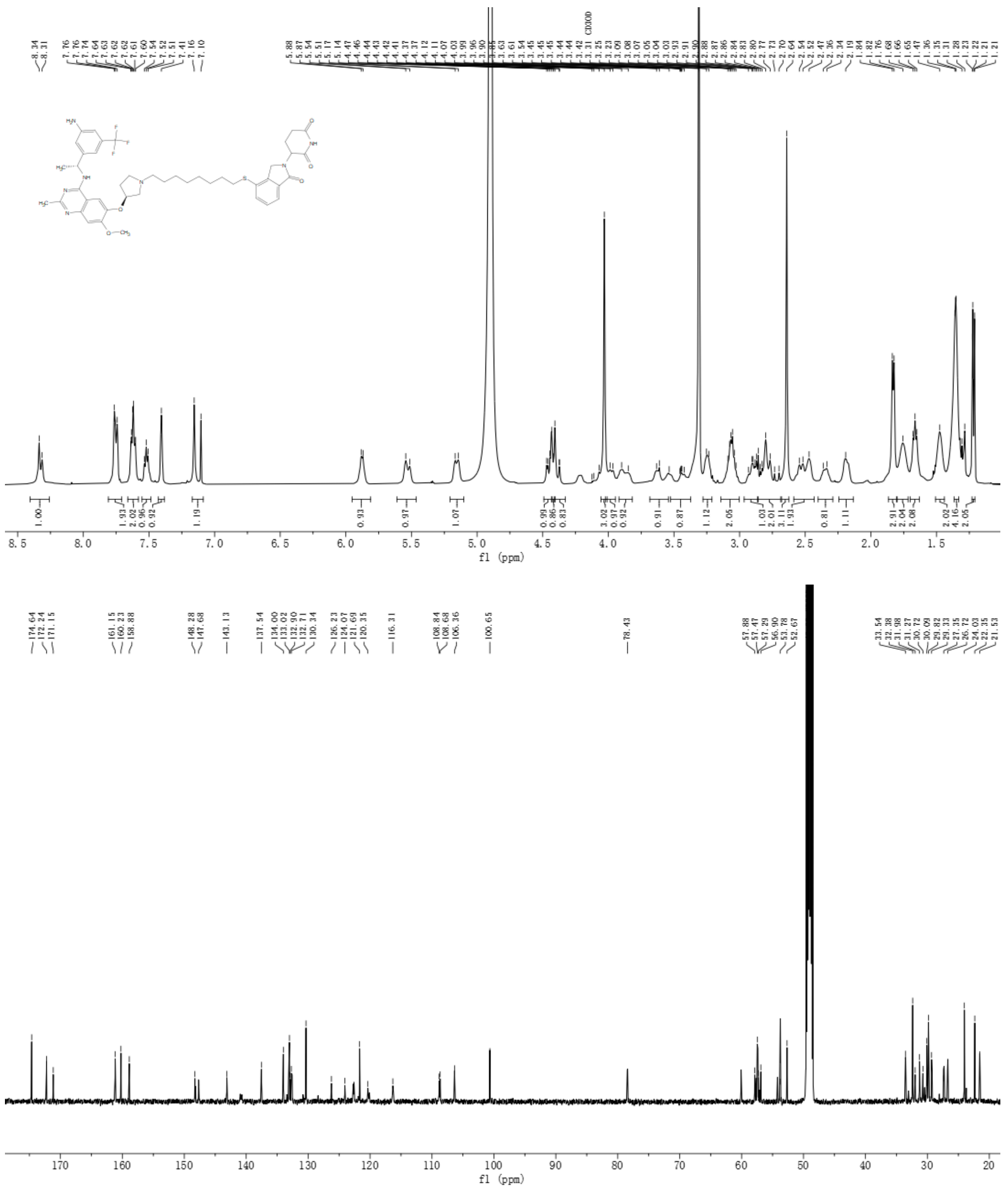

Figure S87. ^{1}H and ^{13}C spectra of **C7** in Methanol-D4.

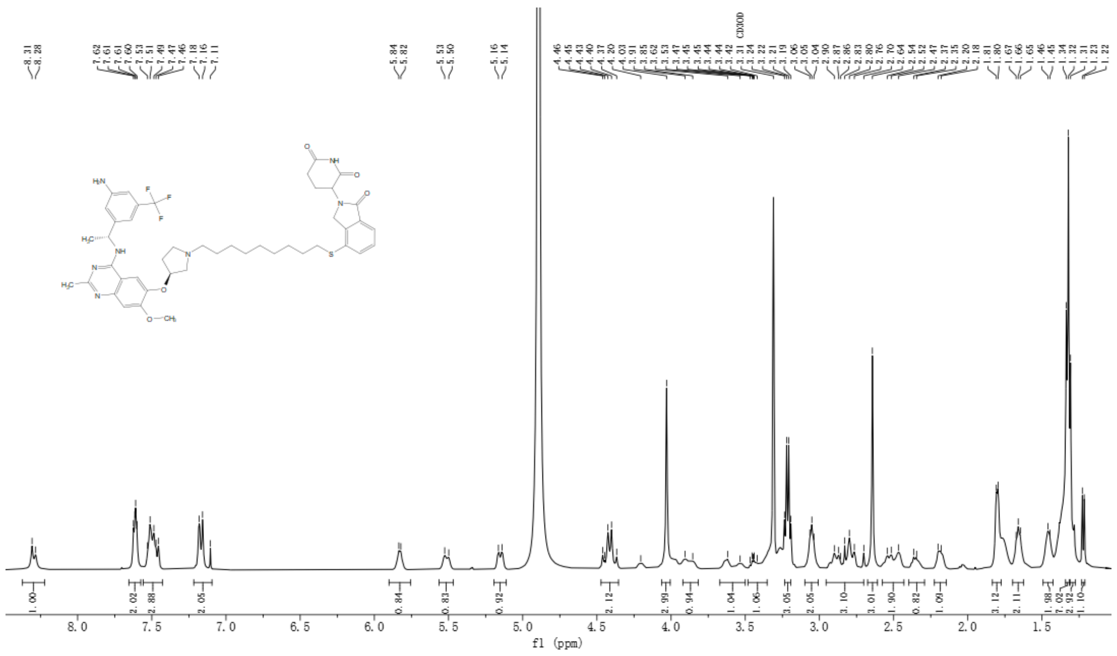

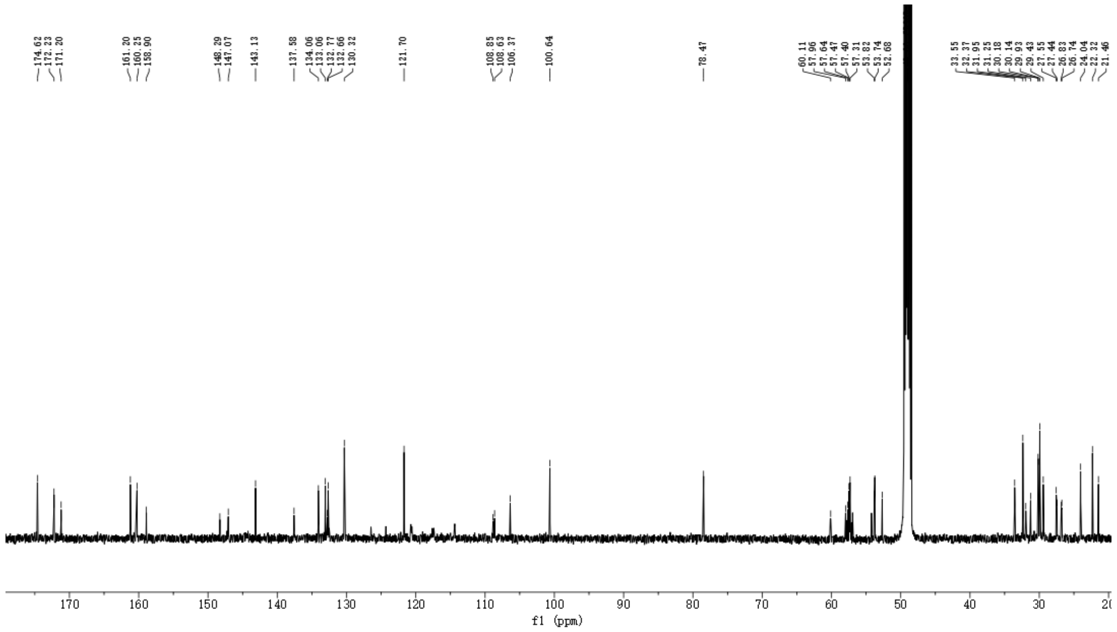

Figure S88. ^{1}H and ^{13}C spectra of **C8** in Methanol-D4.

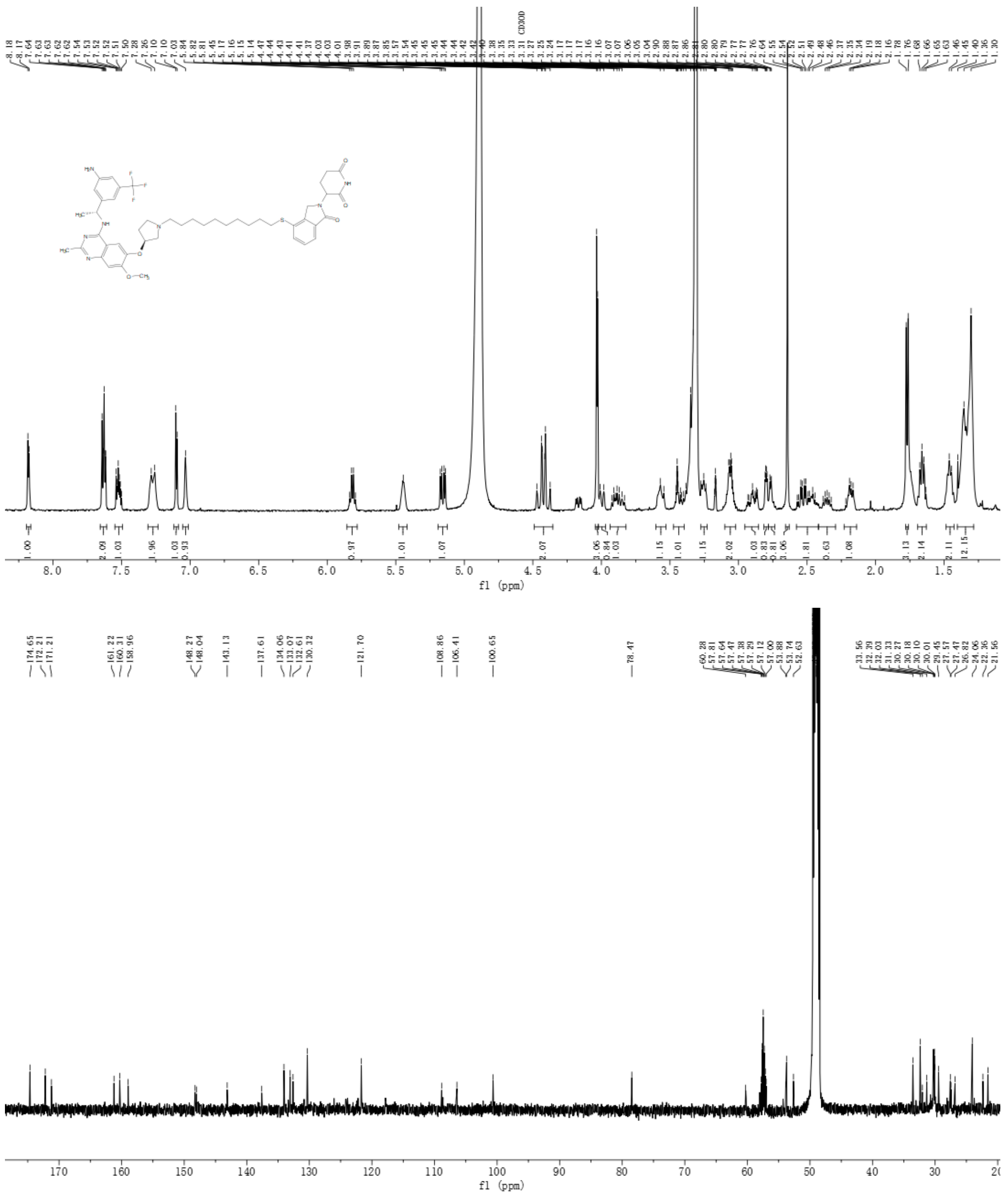

Figure S89. ^{1}H and ^{13}C spectra of **C9** in Methanol-D4.

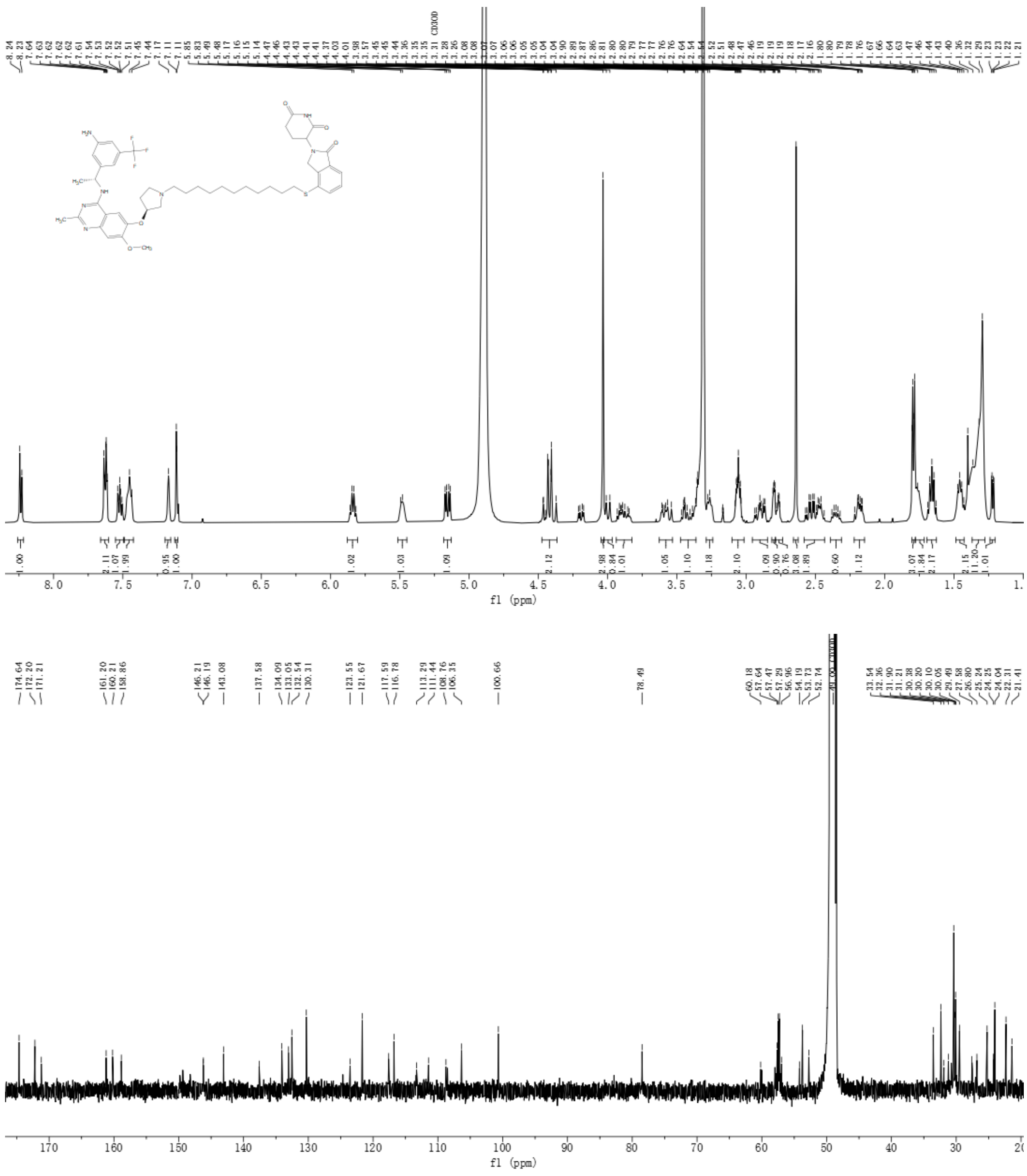

Figure S90. ^{1}H and ^{13}C spectra of **C10** in Methanol-D4.

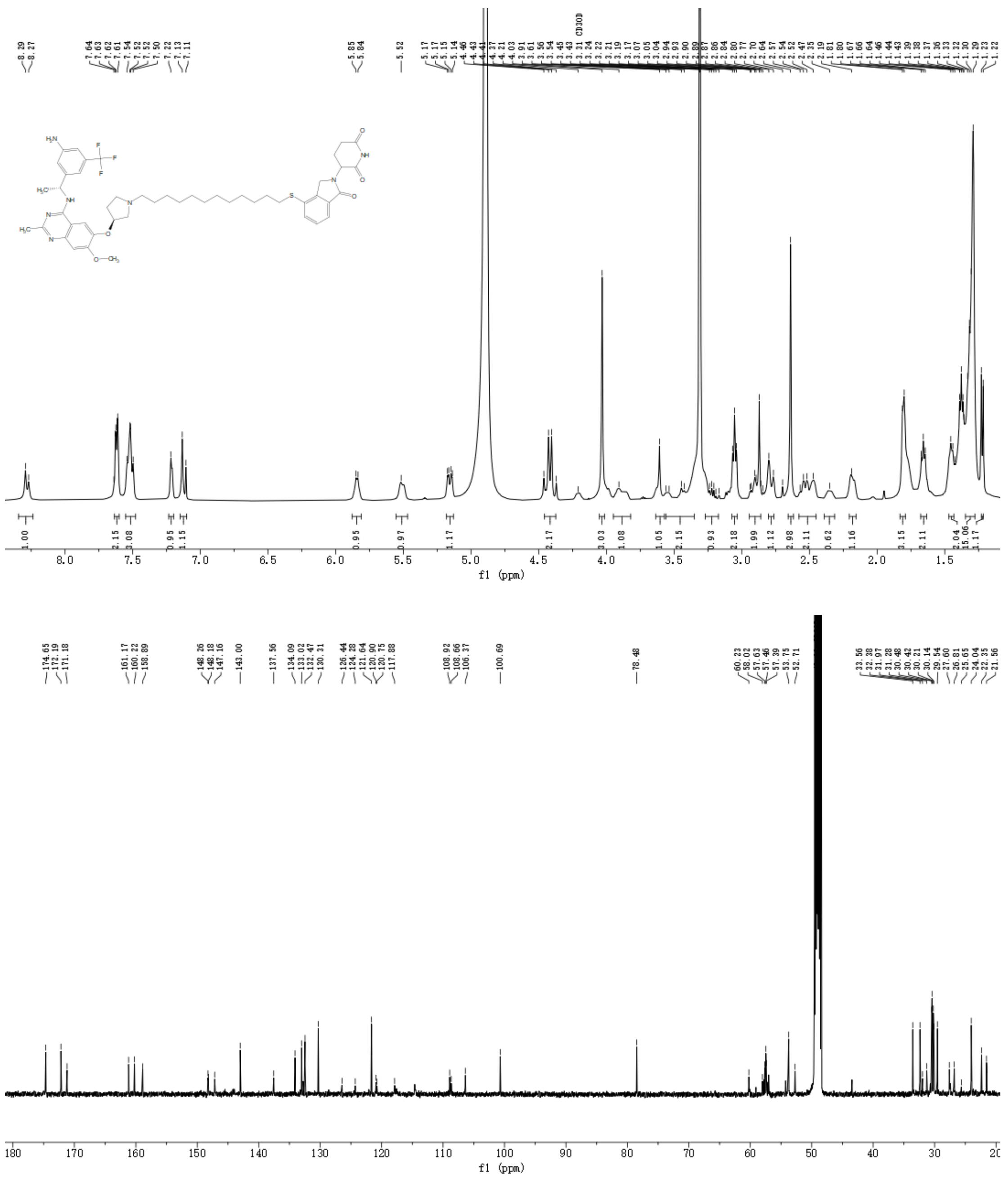

Figure S91. ^{1}H and ^{13}C spectra of **C11** in Methanol-D4.

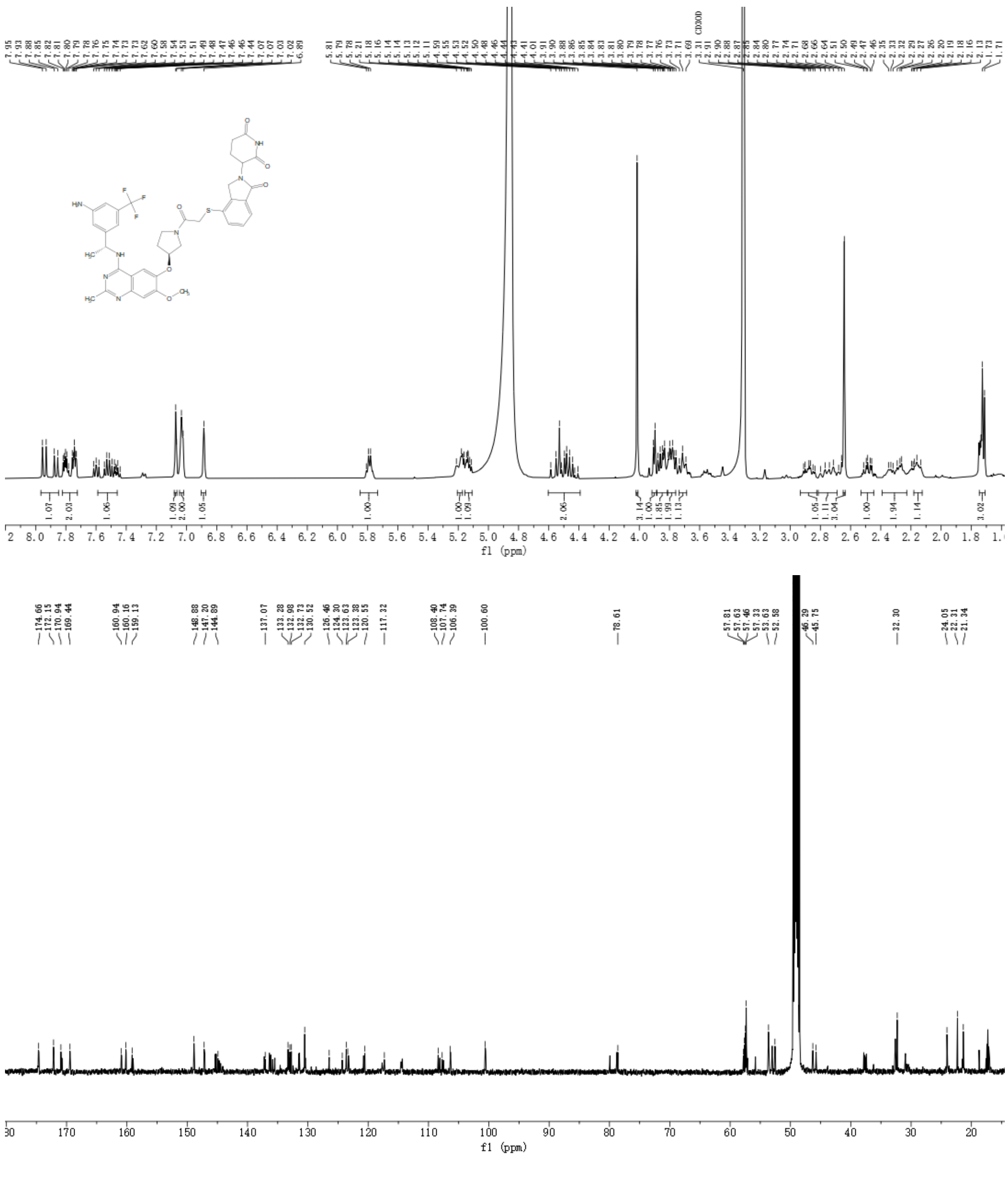

Figure S92. ^{1}H and ^{13}C spectra of **D1** in Methanol-D4.

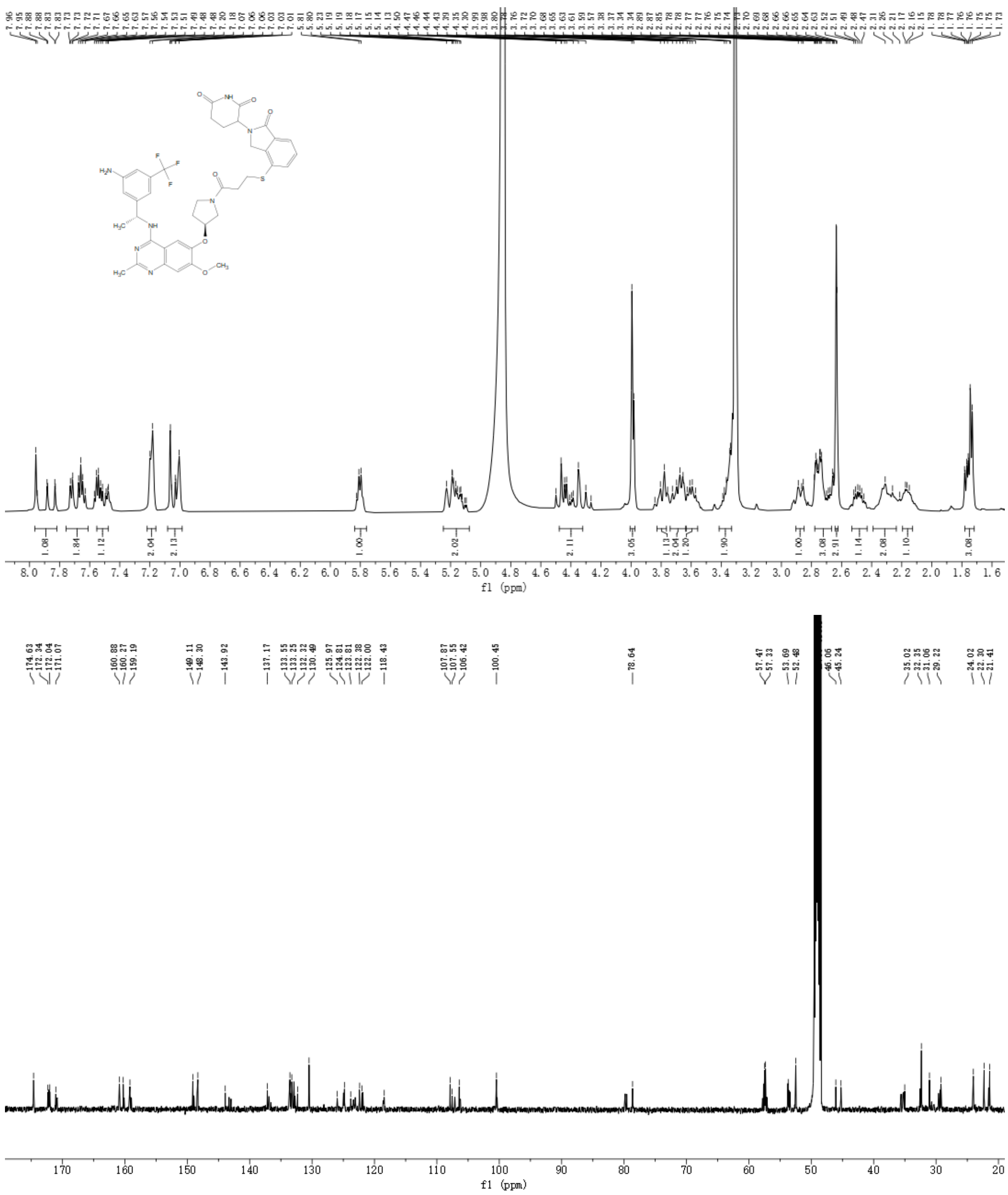

Figure S93. ^{1}H and ^{13}C spectra of **D2** in Methanol-D4.

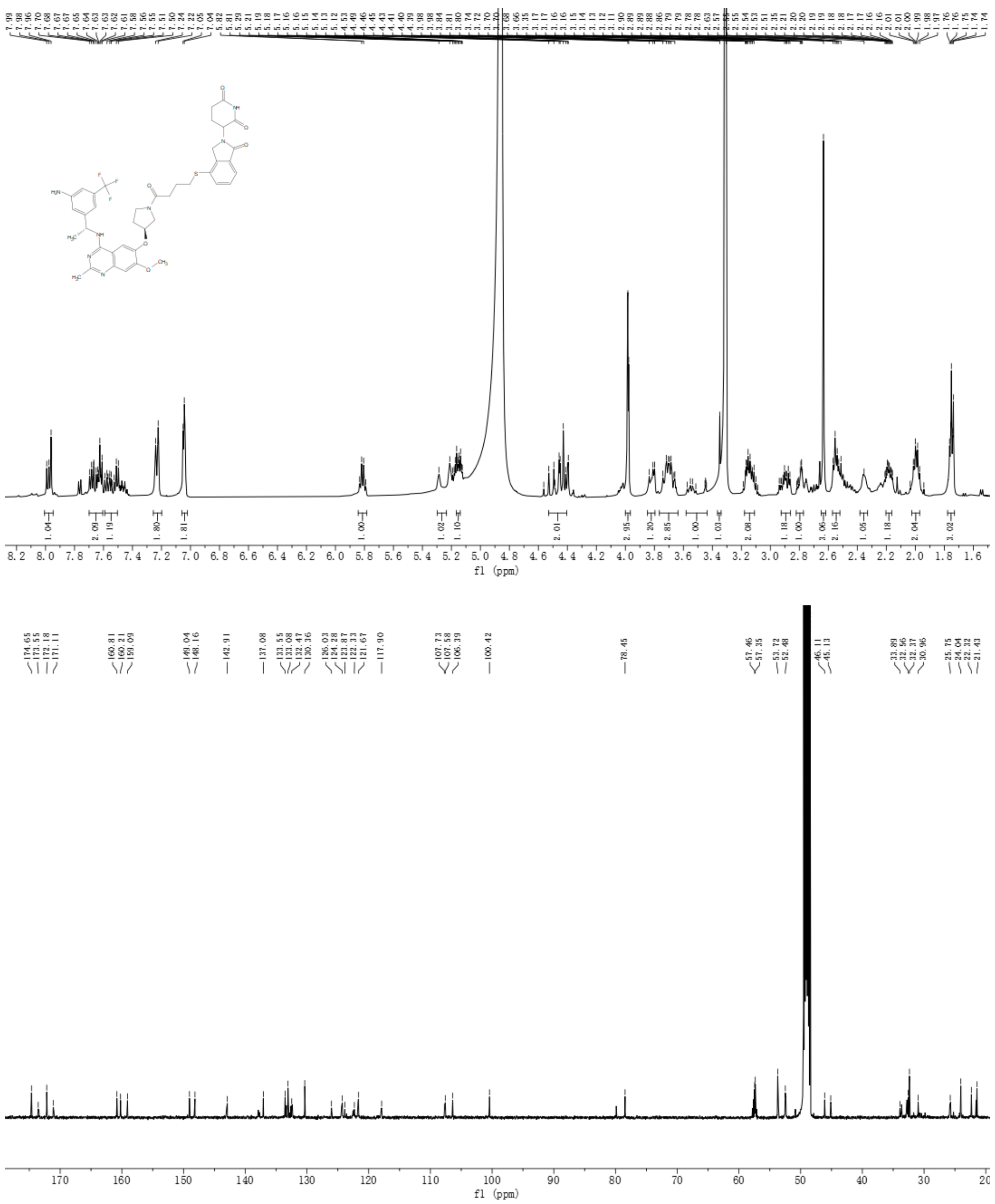

Figure S94. ^{1}H and ^{13}C spectra of **D3** in Methanol-D4.

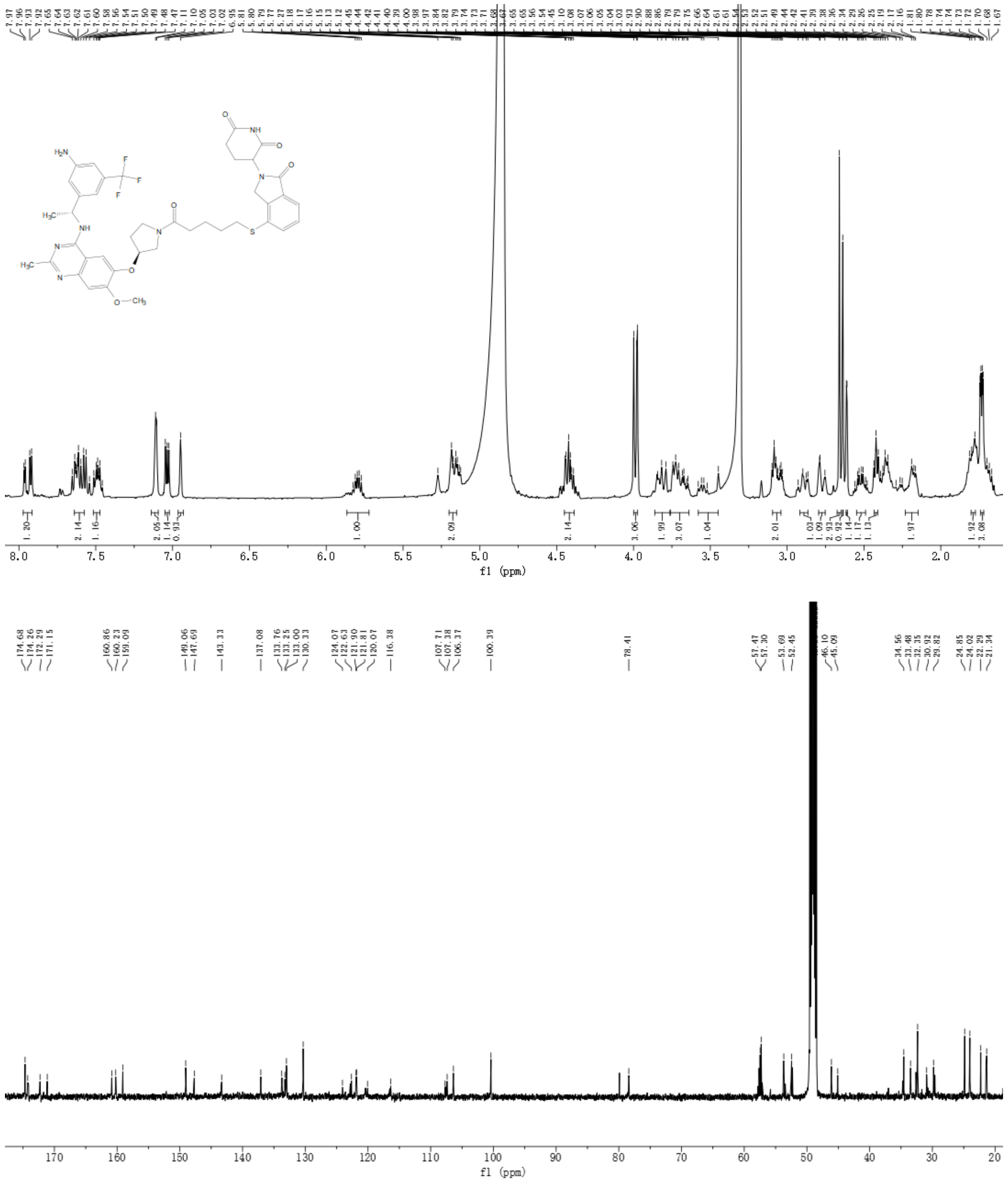

Figure S95. ^{1}H and ^{13}C spectra of **D4** in Methanol-D4.

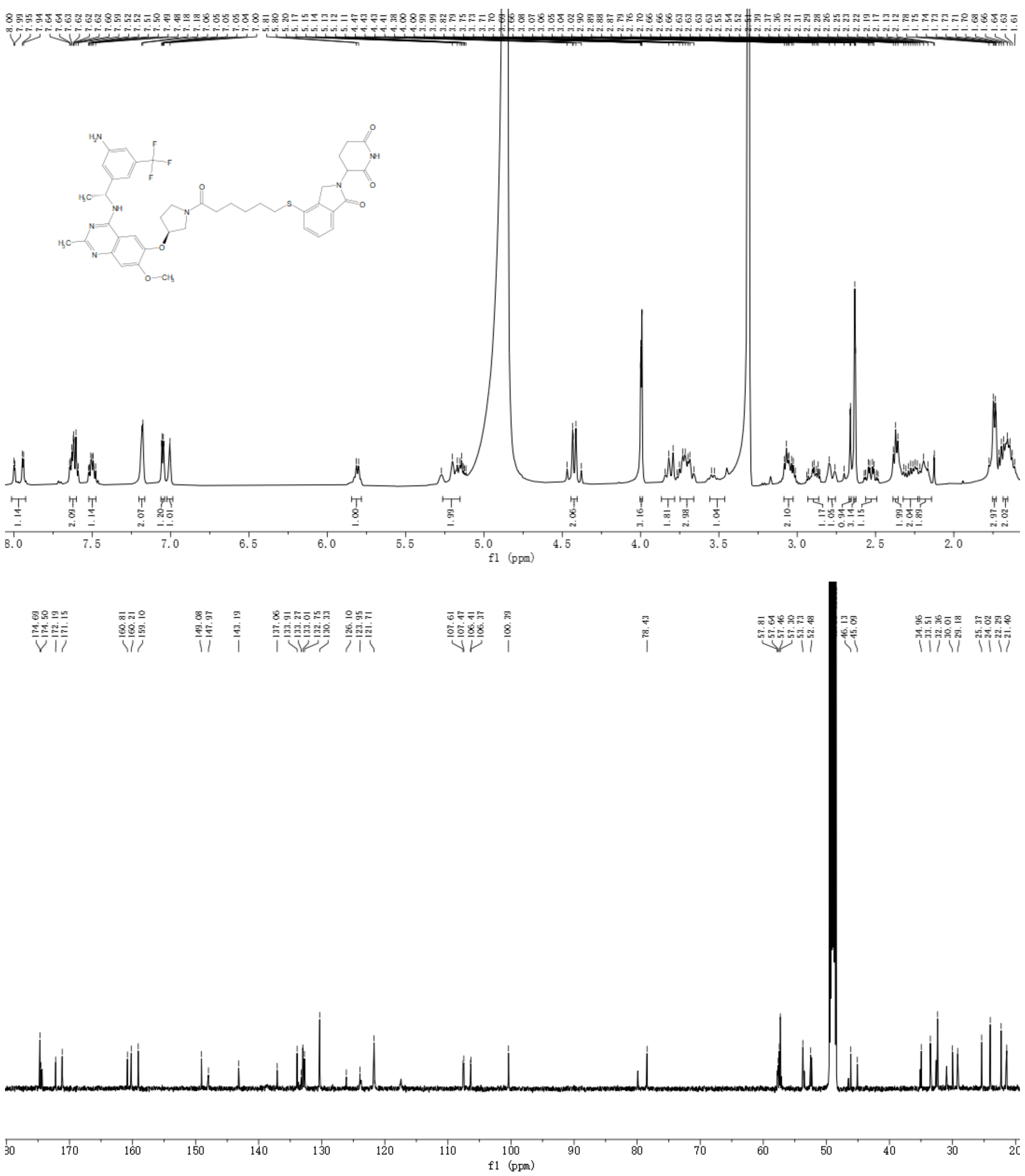

Figure S96. ^{1}H and ^{13}C spectra of **D5** in Methanol-D4.

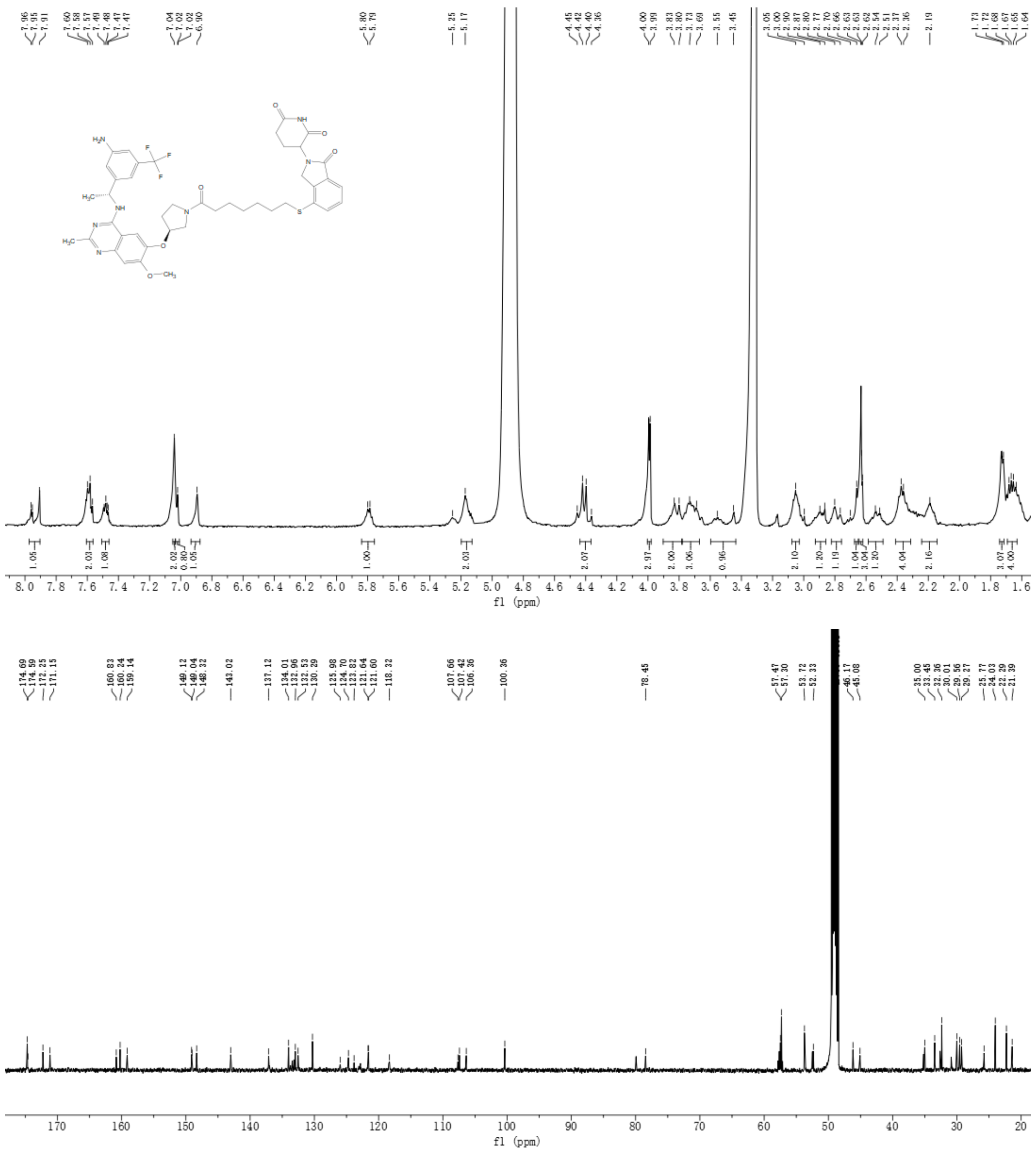

Figure S97. ^{1}H and ^{13}C spectra of **D6** in Methanol-D4.

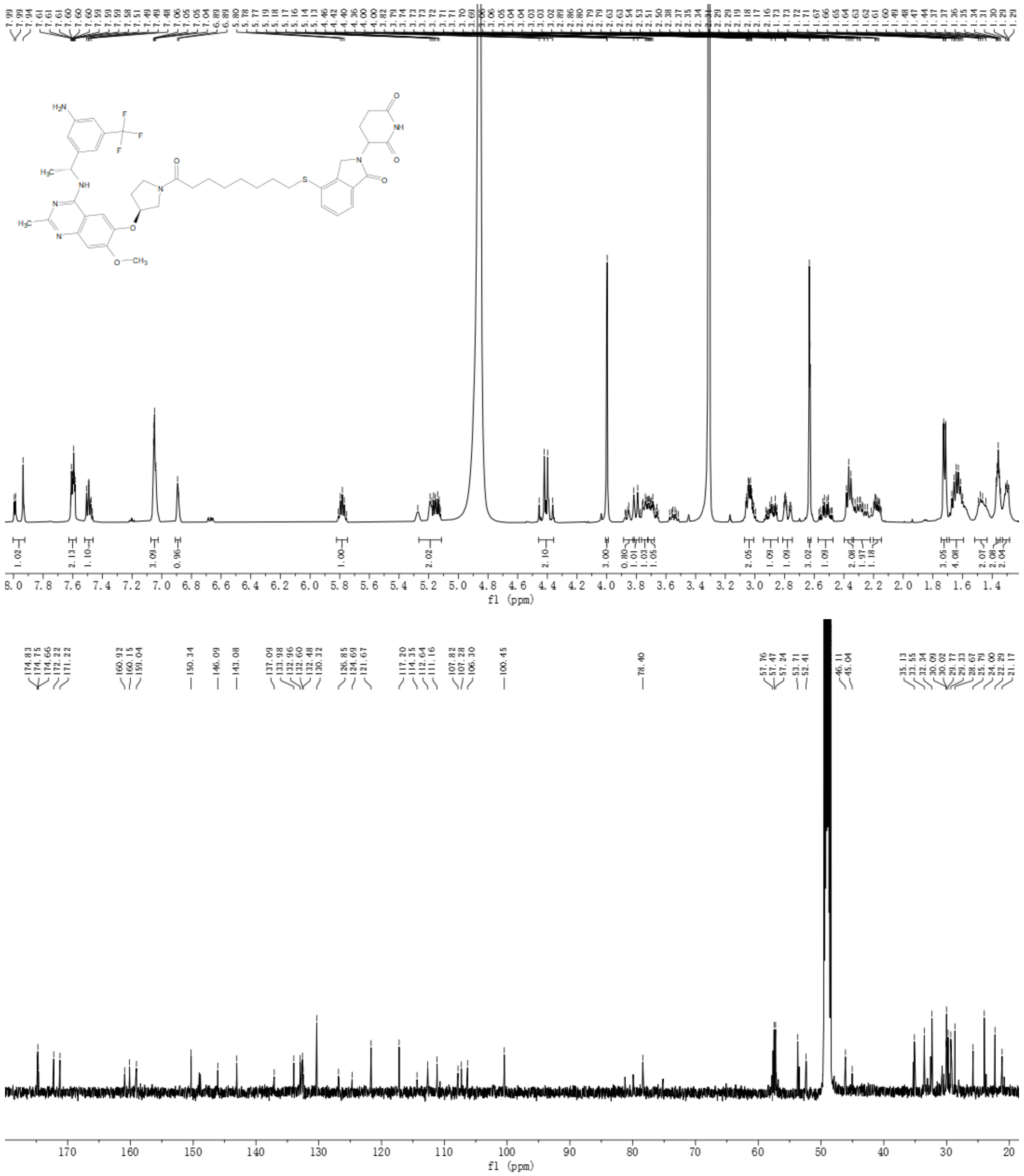

Figure S98. ^{1}H and ^{13}C spectra of **D7** in Methanol-D4.

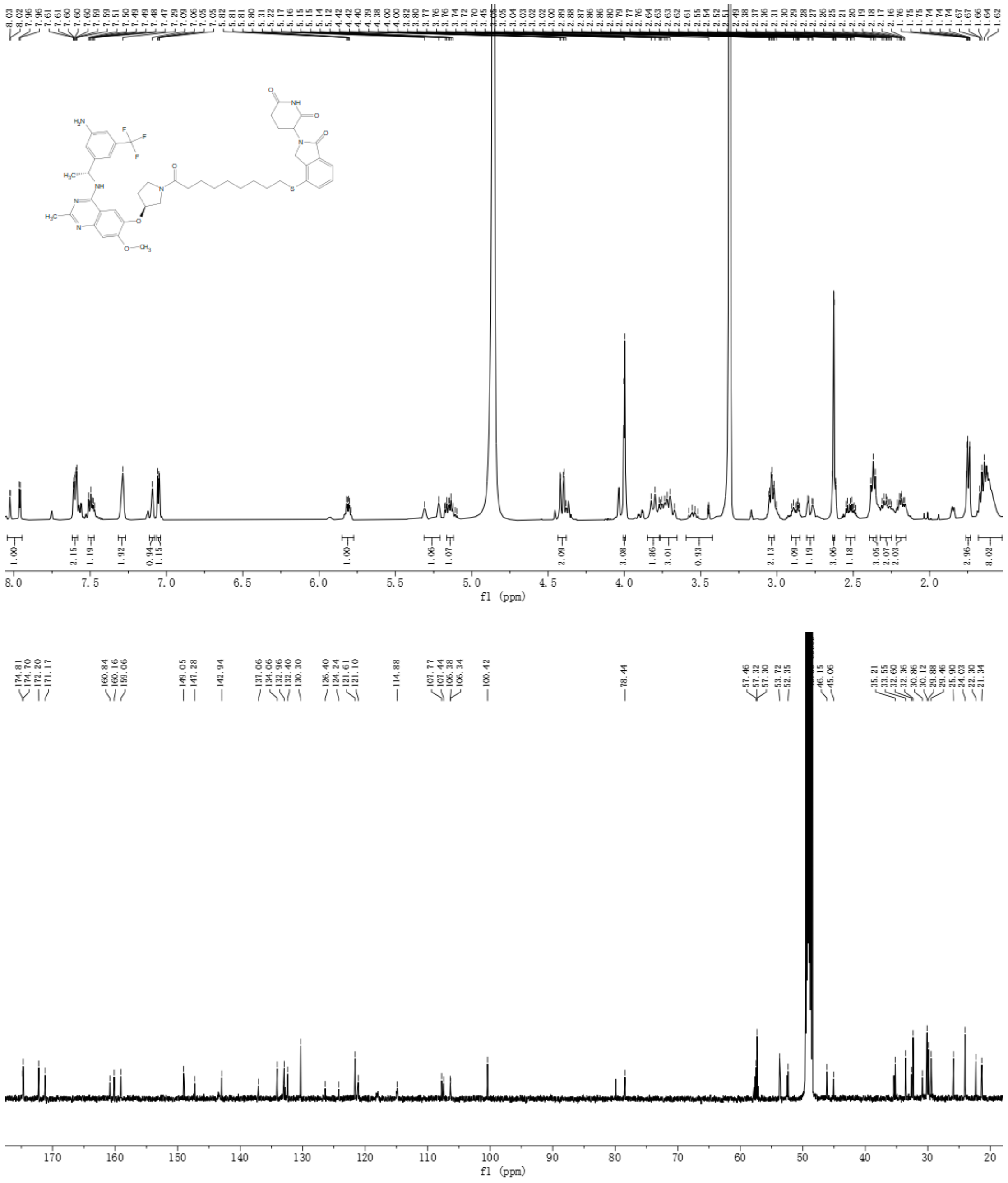

Figure S99. ^{1}H and ^{13}C spectra of **D8** in Methanol-D4.

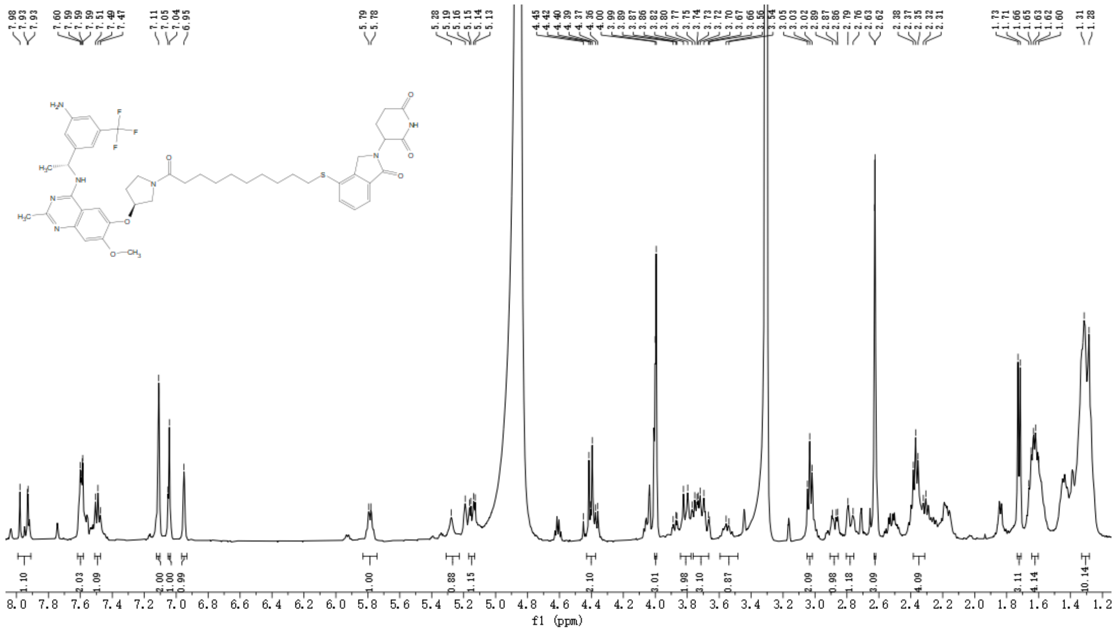

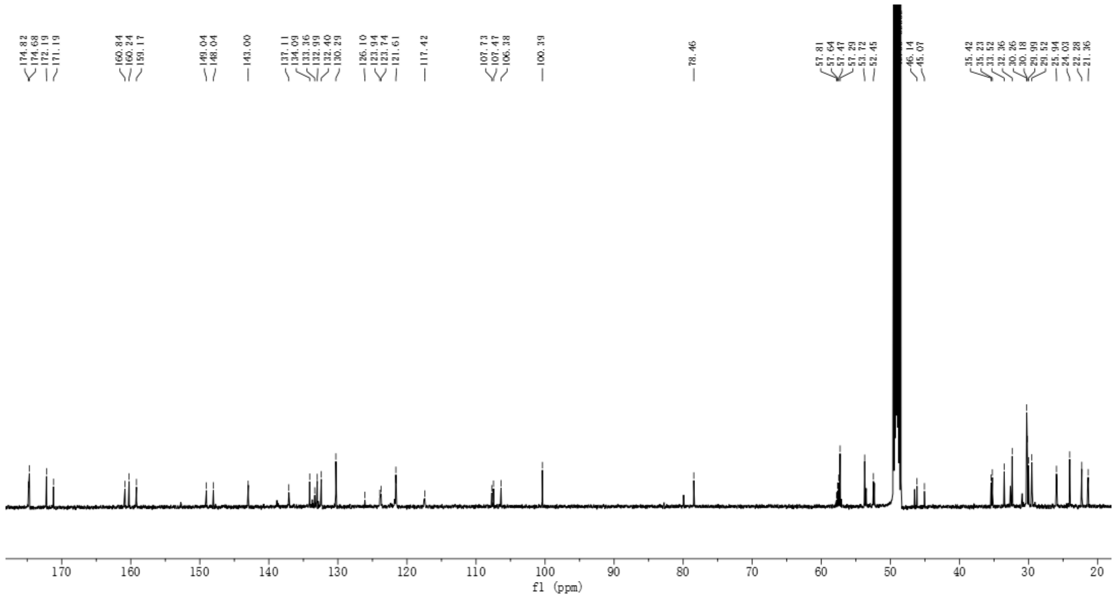

Figure S100. ^{1}H and ^{13}C spectra of **D9** in Methanol-D4.

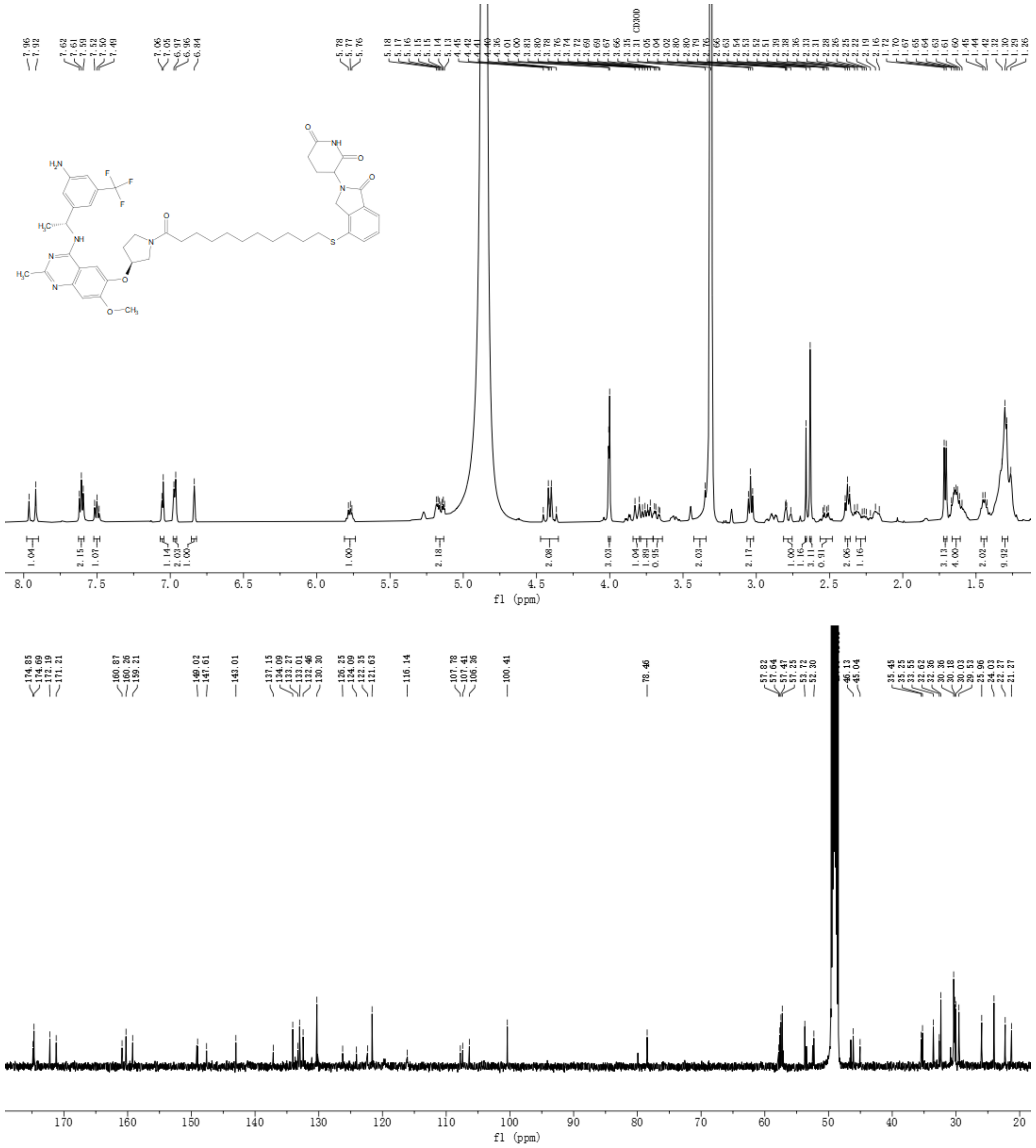

Figure S101. ^{1}H and ^{13}C spectra of **D10** in Methanol-D4.

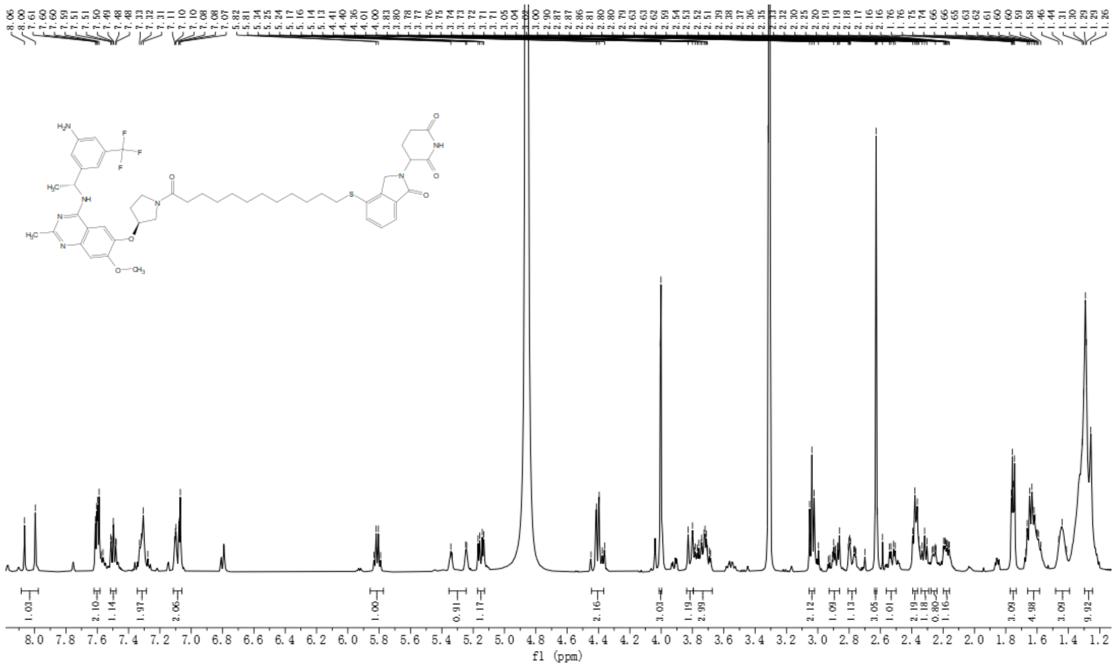

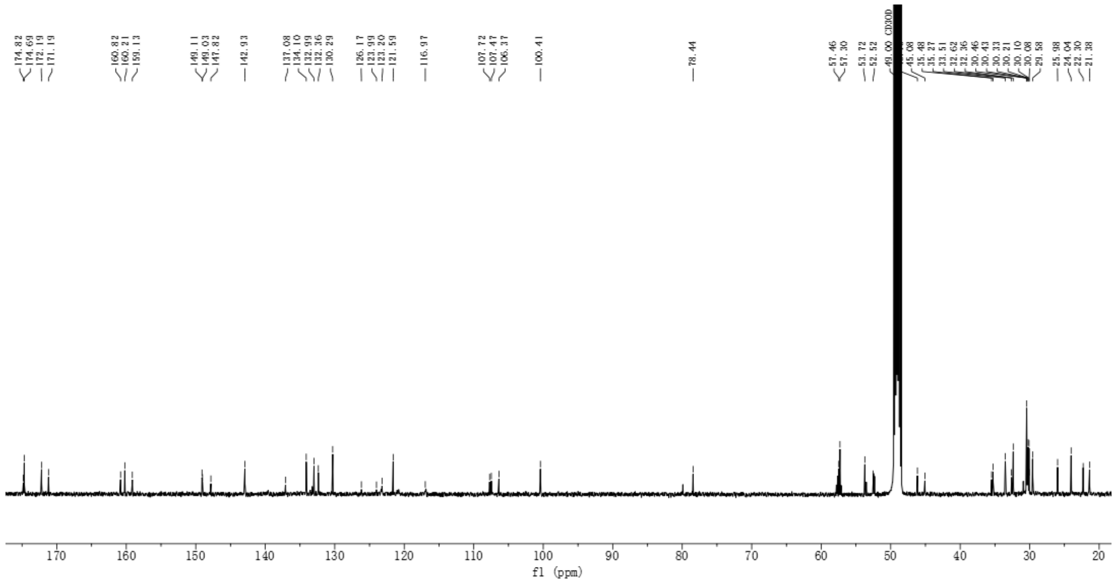

Figure S102. ^{1}H and ^{13}C spectra of **D11** in Methanol-D4.
